# Combining the catalytic enantioselective reaction of visible-light-generated radicals with a by-product utilization system†
†Electronic supplementary information (ESI) available: Characterization data and experimental procedures. CCDC 1547314–1547316. For ESI and crystallographic data in CIF or other electronic format see DOI: 10.1039/c7sc02621h



**DOI:** 10.1039/c7sc02621h

**Published:** 2017-09-01

**Authors:** Xiaoqiang Huang, Shipeng Luo, Olaf Burghaus, Richard D. Webster, Klaus Harms, Eric Meggers

**Affiliations:** a Fachbereich Chemie , Philipps-Universität Marburg , Hans-Meerwein-Strasse 4 , 35043 Marburg , Germany . Email: meggers@chemie.uni-marburg.de; b Division of Chemistry and Biological Chemistry , School of Physical and Mathematical Sciences , Nanyang Technological University , Singapore 637371 , Singapore

## Abstract

We report an unusual reaction design in which a chiral bis-cyclometalated rhodium(iii) complex enables the stereocontrolled chemistry of photo-generated carbon-centered radicals and at the same time catalyzes an enantioselective sulfonyl radical addition to an alkene. Specifically, employing inexpensive and readily available Hantzsch esters as the photoredox mediator, Rh-coordinated prochiral radicals generated by a selective photoinduced single electron reduction are trapped by allyl sulfones in a highly stereocontrolled fashion, providing radical allylation products with up to 97% ee. The hereby formed fragmented sulfonyl radicals are utilized *via* an enantioselective radical addition to form chiral sulfones, which minimizes waste generation.

## Abstract 2

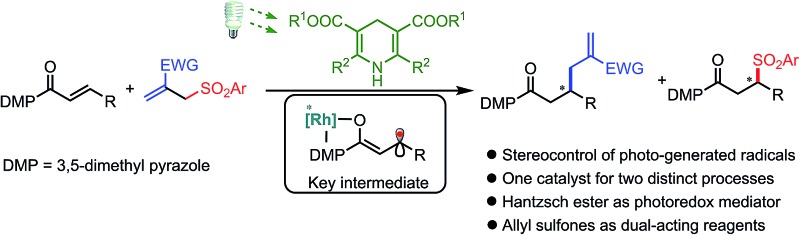
We report an unusual reaction design in which a chiral bis-cyclometalated rhodium(iii) complex enables the stereocontrolled chemistry of photo-generated carbon-centered radicals and at the same time catalyzes an enantioselective sulfonyl radical addition to an alkene.

## Introduction

**Fig. 1:**
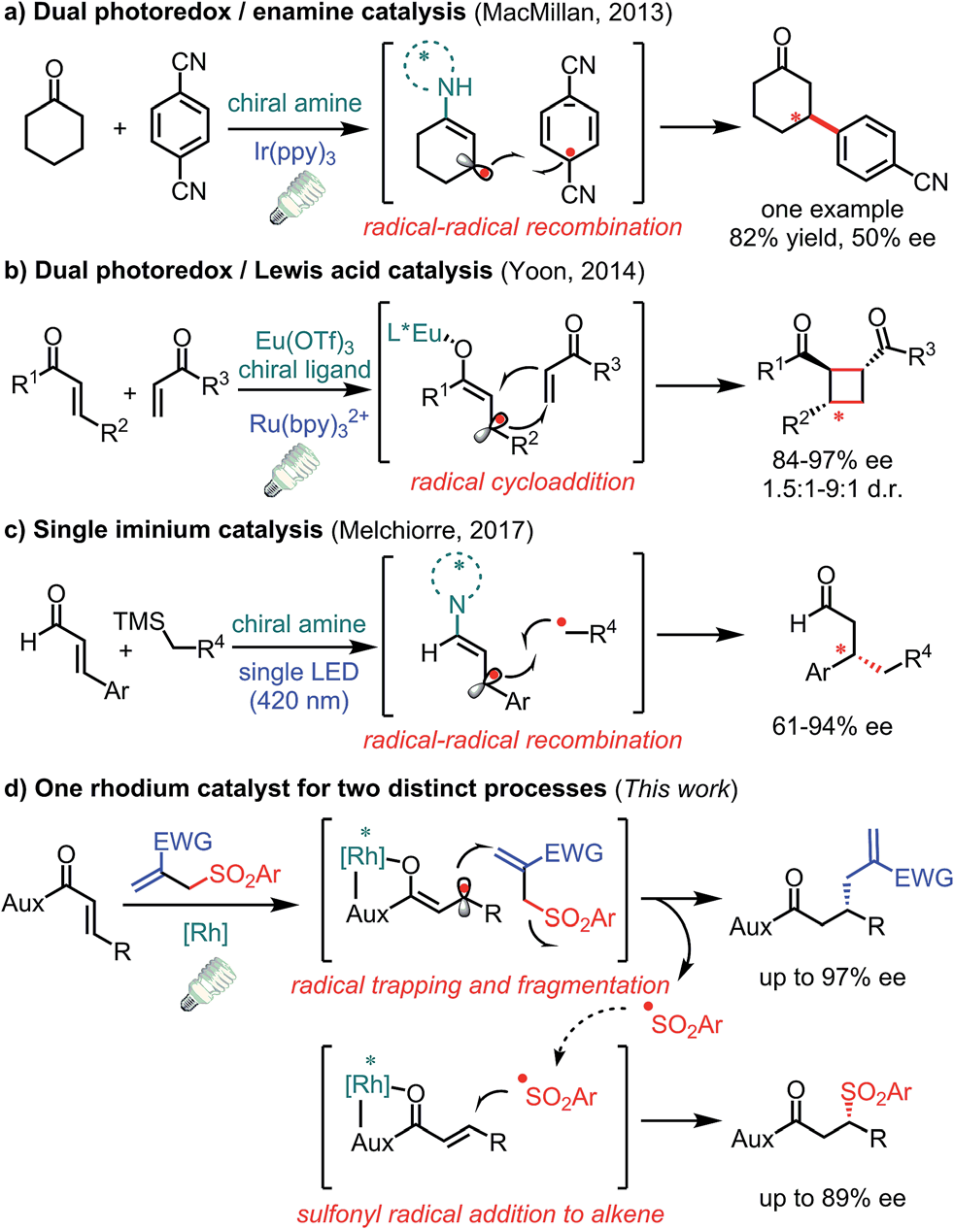
Visible-light-activated asymmetric transformations with allylic C(sp^2^) radical intermediates.

The conversion of prochiral carbon-centered radicals into stereocenters in a catalytic and enantioselective fashion is extremely challenging owing to the inherent high reactivity and conformational flexibility of such radical species.^1^ Over the past few years, photoinduced electron transfer (PET) has emerged as a powerful tool to access radical species in a mild and economic way,^2^ thus spurring on the discovery of novel asymmetric catalytic systems involving radical processes.^3^ However, the catalytic asymmetric PET-processes developed to date mainly deal with achiral radicals reacting with a prochiral C(sp^2^)-center bound to a chiral catalyst.^4^ In contrast, strategies for the direct stereocontrol of photo-generated carbon radical centers are underdeveloped.^5–7^ In this respect, some elegant protocols have been disclosed in enantioselective transformations of photo-generated allylic carbon radicals. In 2013, MacMillan and co-workers introduced a radical–radical recombination process by the combination of a chiral amine and photoredox catalyst in which only one example with moderate enantioselectivity was reported showing the challenge of stereocontrol over such radical intermediates (Fig. 1a).^8^ After that, Yoon and co-workers described a dual Lewis acid and photoredox catalyst for enantioselective radical [2 + 2] cycloadditions (Fig. 1b).^9^ Very recently, Melchiorre and co-workers reported that a single chiral iminium ion enabled the enantioselective coupling of β-enaminyl radicals with alkyl radicals (Fig. 1c).^10^ Despite these significant processes, novel transformations with new mechanisms are still highly desirable.

Herein, we demonstrate that a rhodium-based Lewis acid bearing exclusive metal-centered chirality can effectively control the stereochemistry of visible-light-generated prochiral radicals for the asymmetric radical allylation reaction with allyl sulfones as radical traps (Fig. 1d). Notably, the leaving sulfonyl radicals can be utilized providing β-sulfonyl carbonyl compounds which is without precedence in the chemistry of these well-developed sulfone-based radical trap reagents.^11,12^

## Reaction design

**Fig. 2:**
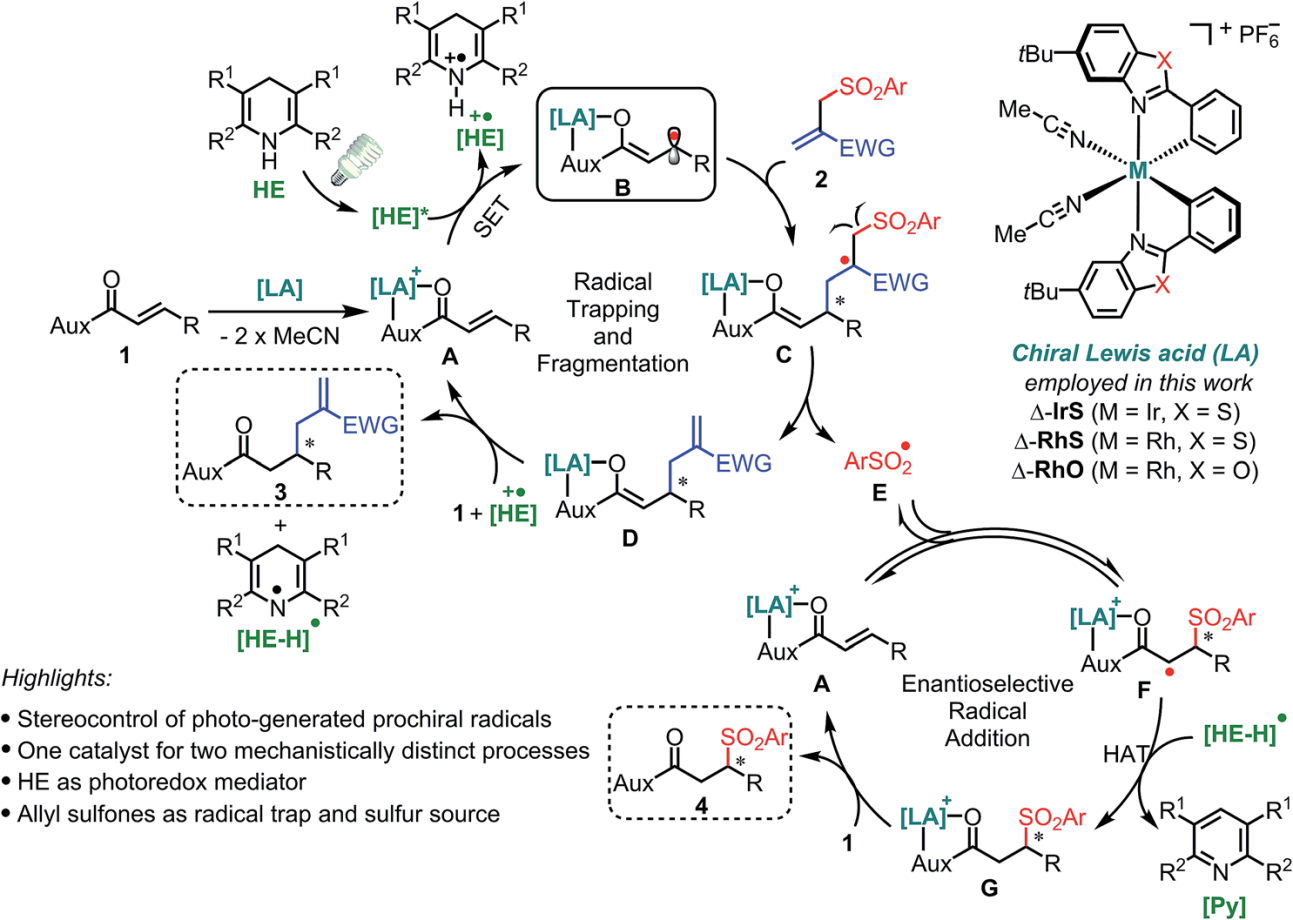
Reaction design and mechanistic proposal. One chiral rhodium catalyst for both enantioselective transformation of a carbon-centered radical and sulfonyl radical addition to an alkene. **HE** = Hantzsch ester, SET = single electron transfer, HAT = hydrogen atom transfer.

Our design is based on our recently introduced bis-cyclometalated chiral rhodium-based Lewis acids (LAs)^13,14^ for the electronic activation of substrates through two-point binding and employs Hantzsch esters (**HE**s)^15^ as a photoredox mediator for the generation of radical species under mild conditions (Fig. 2). Initially, the bidentate coordination of substrate **1** with the Lewis acid forms the LA/substrate complex **A** which is a much better electron acceptor than the free substrate **1**.^14*c*^ Therefore, the selective SET reduction of **A** by the visible-light-excited **HE** (HE*) generates the key Rh-coordinated radical intermediate **B**, which is trapped by an electron-deficient allyl sulfone **2** delivering the secondary radical intermediate **C**. The subsequent fragmentation^11^ of **C** provides the sulfonyl radical **E** and enolate intermediate **D**, the latter of which yields the C–C bond formation product **3** upon protonation. Meanwhile, the sulfonyl radical **E** undergoes a stereocontrolled radical addition^16^ to **A** in a reversible fashion^11^ and a subsequent HAT followed by ligand exchange provides the C–S bond formation product **4**.

Two key challenges are needed to be solved to achieve a high asymmetric induction. Firstly, a robust and effective chiral Lewis acid catalyst is required to control the stereochemistry of two mechanistically distinct processes as well as reduce the reduction potential of substrate **1** to ensure a highly chemoselective reduction. Secondly, the radical trapping and subsequent fragmentation process should be fast enough to compete with the protonation of intermediate **B** which would generate undesirable free β-carbonyl carbon radicals. The reaction of such free radicals with **2** would compromise the enantioselectivity of product **3**. Therefore, this design with the utilization of the leaving sulfonyl radicals, which otherwise would lead to by-products, is very attractive not only from the perspective of green and sustainable chemistry^17^ but also for suppressing side reactions of the sulfonyl radical and shifting the equilibrium of a potentially reversible radical fragmentation.^11^

## Results and discussion

We commenced this proposed process by the reaction of α,β-unsaturated *N*-acylpyrazole **1a** with allyl sulfone **2a** under visible light irradiation employing a stoichiometric amount of the Hantzsch ester **HE-1** as the photoredox mediator and reductant.^15^ Although our well-established iridium catalyst Δ-**IrS** ^18^ could not give any detectable product (Table 1, entry 1), the rhodium analog Δ-**RhS** ^14*b*^ enabled the transformation affording the expected allylation product **3a** and C–S formation product **4a** in excellent yields with moderate enantioselectivities (entry 2). Interestingly, the related Δ-**RhO** ^14a,19^ provided **3a** in much higher ee (96% ee, entry 3) indicating that the mechanism differs from our previous reports^14c,d^ about Giese-type radical reactions in which **RhS**, which features a higher steric congestion, works better than **RhO**. Notably, our recently developed chiral rhodium complexes show a unique reactivity for this reaction. Other Lewis acids such as Sc(OTf)_3_ gave very low efficiency while LiBF_4_ could not even catalyze the process (entries 4 and 5) and no conversion was observed without catalyst (entry 6). Other substituted **HE** species also worked very well (entries 7 and 8), whereas DIPEA, which is widely used as a sacrificial reductant in photoredox catalysis, could not accomplish the transformation (entry 9). These results highlight the multiple functions of the **HE** in our system acting as a photoredox mediator as well as an electron donor and proton source. Furthermore, on illumination with blue LEDs, which do not emit any UV light (see ESI for the emission spectra†), the reaction gave comparable results (entry 10). Together with the control experiments in darkness (entry 11), this confirms that the reaction is activated by visible light.

**Table 1:** Optimization of the reaction conditions^*a*^

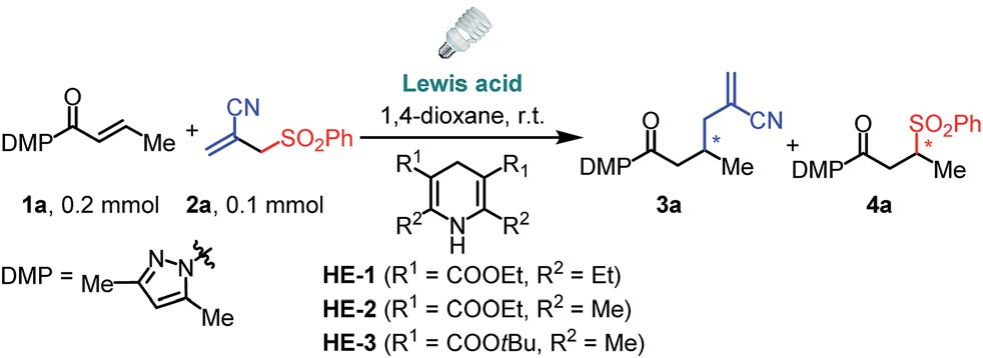						
Entry	Lewis acid^*b*^	**HE**	**3a**		**4a**	
			Yield^*c*^ (%)	ee^*d*^ (%)	Yield^*c*^ (%)	ee^*d*^ (%)
1	Δ-**IrS** (8.0)	**HE-1**	<5	n.a.	<5	n.a.
2	Δ-**RhS** (8.0)	**HE-1**	92	83	95	84
3	Δ-**RhO** (8.0)	**HE-1**	85	96	92	85
4	Sc(OTf)_3_ (20)	**HE-1**	10	n.a.	10	n.a.
5	LiBF_4_ (200)	**HE-1**	0	n.a.	0	n.a.
6	None	**HE-1**	0	n.a.	0	n.a.
7	Δ-**RhO** (8.0)	**HE-2**	78	96	82	80
8	Δ-**RhO** (8.0)	**HE-3**	80	94	85	85
9^*e*^	Δ-**RhO** (8.0)	None	0	n.a.	0	n.a.
10^*f*^	Δ-**RhO** (8.0)	**HE-1**	77	92	80	80
11^*g*^	Δ-**RhO** (8.0)	**HE-1**	0	n.a.	0	n.a.

^*a*^Reaction conditions: **1a** (0.20 mmol), **2a** (0.10 mmol), Lewis acid and Hantzsch ester (0.15 mmol) in 1,4-dioxane (1.0 mL) were stirred at room temperature for 24 h and irradiated with a 21 W CFL.

^*b*^Loadings in mol% provided in brackets.

^*c*^Isolated yields.

^*d*^Determined by HPLC on a chiral stationary phase.

^*e*^0.15 mmol of *N*,*N*-diisopropylethylamine (DIPEA) was employed.

^*f*^Blue LEDs (24 W) were used instead of a CFL (21 W).

^*g*^Performed in darkness. n.a. = not applicable.

With the optimized conditions at hand, we next investigated the substrate scope with respect to radical acceptors (Table 2). A wide range of allyl sulfones **2** with different leaving groups works well, delivering the radical allylation product **3a** in good yields and excellent ee (up to 97% ee) along with the recycled C–S formation products **4a–h** in good yields and ee (up to 89% ee) (entries 1–8). Intriguingly, a lower yield and slightly lower ee were observed for the radical functionalization product **3b** when allyl sulfone bearing a less electron deficient ester group was employed (compare entries 9 with 1). It is noteworthy that functional groups including a C≡C triple bond, a C=C double bond and an imide are well tolerated under these mild conditions (entries 11–13). As a limitation, substrates with a long chain at the β-position (**1b–d**) produced the radical allylation products **3f–h** with decreased ee (eqn (1)) and β-aryl α,β-unsaturated *N*-acylpyrazole could not afford any expected product. Furthermore, the alkenyl sulfone **5** was proven to be competent, providing the radical alkenylation product **6** in 54% yield with 93% ee (eqn (2)).

**Table 2:** Substrate scope with respect to allyl sulfones^*a*^

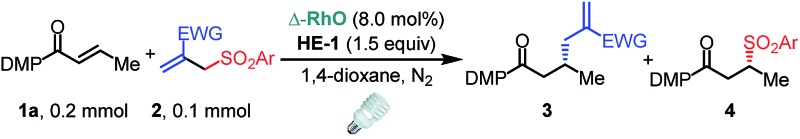				
Entry	EWG	Ar	**3**, Yield^*b*^, ee^*c*^	**4**, Yield^*b*^, ee^*c*^
1	CN	C_6_H_5_	**3a**, 85%, 96% ee	**4a**, 92%, 85% ee
2	CN	4-MeC_6_H_4_	**3a**, 68%, 96% ee	**4b**, 70%, 79% ee
3	CN	4-BrC_6_H_4_	**3a**, 81%, 97% ee	**4c**, 88%, 80% ee
4	CN	4-CF_3_C_6_H_4_	**3a**, 78%, 95% ee	**4d**, 78%, 76% ee
5	CN	2-MeC_6_H_4_	**3a**, 71%, 95% ee	**4e**, 72%, 86% ee
6^*d*^	CN	2,4,6-Me_3_C_6_H_2_	**3a**, 57%, 94% ee	**4f**, 60%, 89% ee
7^*d*^	CN	2-Naphthyl	**3a**, 82%, 94% ee	**4g**, 88%, 83% ee
8^*d*^	CN	1-Naphthyl	**3a**, 78%, 91% ee	**4h**, 84%, 80% ee
9	COOEt	C_6_H_5_	**3b**, 65%, 94% ee	**4a**, 68%, 84% ee
10^*d*^	COOEt	4-MeOC_6_H_4_	**3b**, 65%, 92% ee	**4i**, 63%, 81% ee
11^*d*^	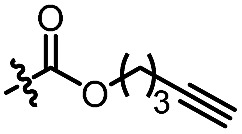	C_6_H_5_	**3c**, 60%, 92% ee	**4a**, 69%, 82% ee
12^*d*^	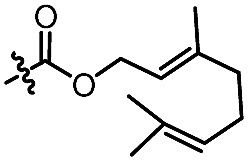	C_6_H_5_	**3d**, 62%, 92% ee	**4a**, 72%, 83% ee
13^*d*^	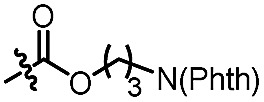	C_6_H_5_	**3e**, 73%, 92% ee	**4a**, 78%, 82% ee

^*a*^Reaction conditions: **1a** (0.20 mmol), **2** (0.10 mmol), Δ-**RhO** (0.008 mmol) and **HE-1** (0.15 mmol) in 1,4-dioxane (1.0 mL) were stirred at room temperature and irradiated with a 21 W CFL.

^*b*^Isolated yields.

^*c*^Determined by HPLC on a chiral stationary phase.

^*d*^35 °C.



1

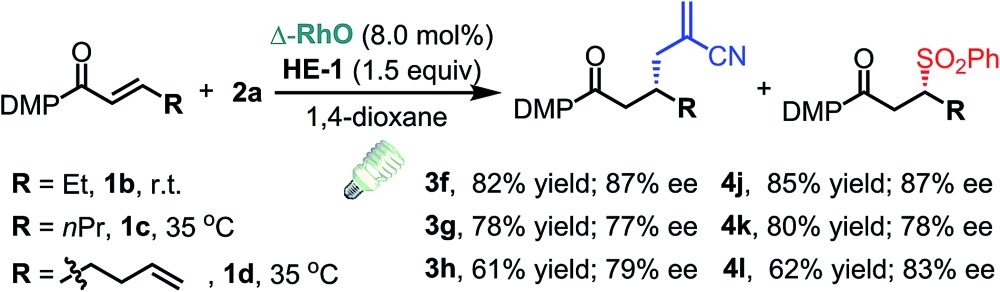



2

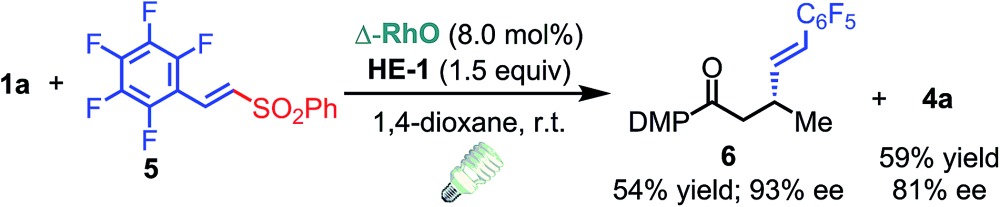




To further evaluate the functional group tolerance and robustness of this catalytic system, we conducted the reaction **1a** + **2a** → **3a** + **4a** in the presence of a series of common chemical functionalities (Table 3 and ESI†).^20^ To our satisfaction, this reaction shows a high chemo-selectivity towards the Lewis acid coordinated substrate (intermediate **A**) as additives containing azido, cyano, and carbonyl groups that are vulnerable to reductive conditions can be recovered in high yields under the standard conditions (entries 1–4). Importantly, several heterocycles which might competitively coordinate to the catalyst did not erode the enantiomeric excess of products (entries 3–9). Several natural products, including coumarin, caffeine, and (–)-citronellol, were found to have little influence on the reaction outcomes (entries 7–10). Furthermore, *N*-acylpyrazoles known as a useful and reactive synthetic building block can be easily converted into other compounds, such as alcohols or amides (eqn (3) and (4)). Overall, these results highlight the potential of this protocol for further applications in the synthesis of complex molecules.

**Table 3:** Reaction compatibility in the presence of additives^*a*^

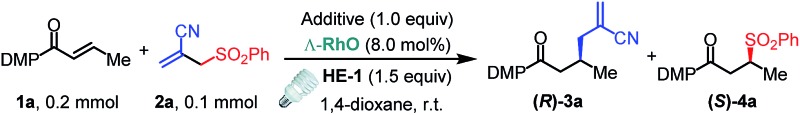						
Entry	Additive	Additive recovered^*b*^	**(*R*)-3a**		**(*S*)-4a**	
			Yield^*b*^	ee^*c*^	Yield^*b*^	ee^*c*^
1	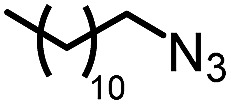	88%	86%	96% ee	85%	86% ee
2	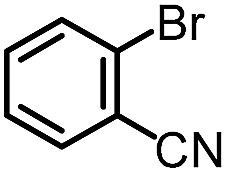	94%	86%	96% ee	85%	83% ee
3	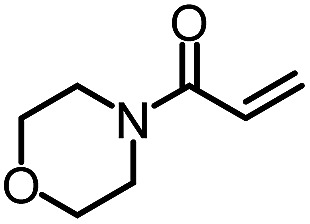	99%	86%	96% ee	92%	83% ee
4	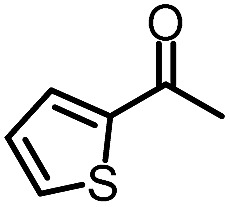	88%	82%	96% ee	88%	83% ee
5	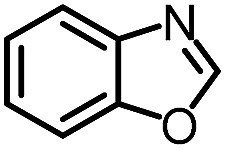	95%	86%	95% ee	92%	85% ee
6	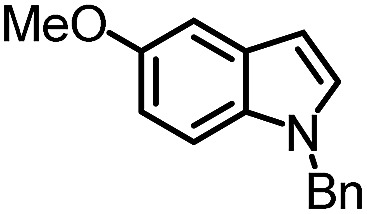	99%	86%	96% ee	85%	83% ee
7	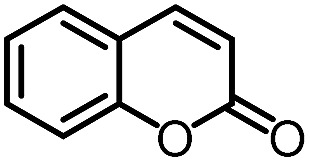	96%	82%	96% ee	92%	85% ee
8	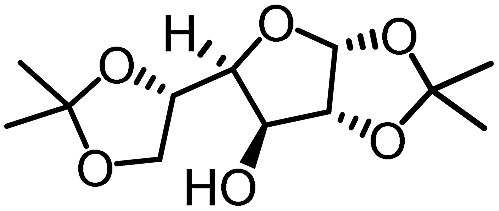	99%	82%	95% ee	92%	83% ee
9	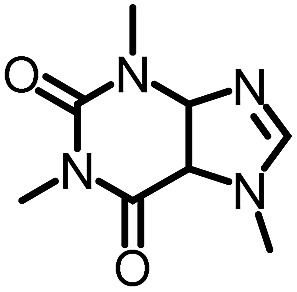	99%	86%	96% ee	92%	85% ee
10	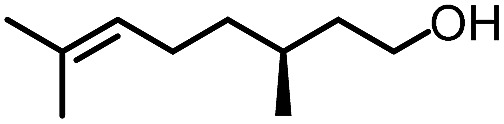	94%	86%	96% ee	92%	85% ee

^*a*^Reaction conditions: **1a** (0.20 mmol), **2a** (0.10 mmol), Λ-**RhO** (0.008 mmol), **HE-1** (0.15 mmol) and additives (0.10 mmol) in 1,4-dioxane (1.0 mL) were stirred at room temperature for 24 h and irradiated with a 21 W CFL.

^*b*^Isolated yields.

^*c*^Determined by HPLC on a chiral stationary phase.



3

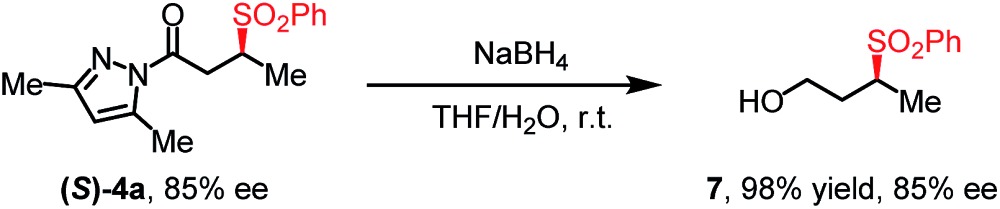



4

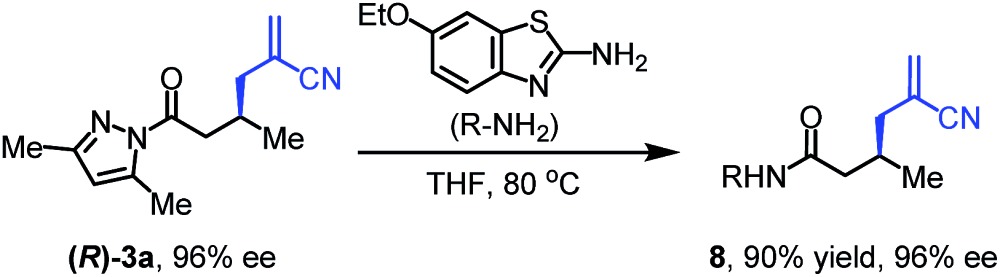




A number of experiments support the proposed mechanism (Fig. 2). Firstly, UV/vis spectra and luminescence spectra support the role of the **HE** as a visible light harvesting antenna being consistent with recent reports (Fig. S3 in ESI†).^15^ Secondly, Stern–Volmer quenching experiments demonstrate that the rhodium coordinated substrate **RhO-1a** ^21^ but not the free substrates **1** or **2** can effectively quench the luminescence of **HE-1**, suggesting that a selective SET process between the photoexcited HE* and intermediate **A** might occur (Fig. S4 and S5 in ESI†). This is further confirmed by cyclic voltammetry, in which a large difference in the reduction peak potential by almost 1 V was observed between **1a** (–2.59 V *vs.* Fc/Fc^+^) and **RhO-1a** (–1.62 V *vs.* Fc/Fc^+^), thus making highly selective SET between **RhO-1a** and the excited state of **HE-1** (*E* ^(HE˙^+^/HE*)^ = –2.23 V *vs.* Fc/Fc^+^) feasible (Fig. S6 in ESI†). Furthermore, the title reaction was greatly inhibited upon adding 2,6-di-*tert*-4-methylphenol (BHT) or 2,2,6,6-tetramethyl-piperidinooxy (TEMPO) as radical scavengers. When the reaction was monitored by electron paramagnetic resonance (EPR) using 5,5-dimethyl-1-pyrroline *N*-oxide (DMPO) as a free radical spin-trapping agent, mixed signals containing two radical species were observed, one of which was identified as a phenyl sulfonyl radical (*g* = 2.006, *α*_N_ = 9.5 G, *α*βH = 12.9 G) (Fig. S8 in ESI†).^22^ Finally, a quantum yield of 0.09 was determined for the reaction **1a** + **2a** → **3a** + **4a**, which is consistent with the proposed mechanism being devoid of any chain process (see ESI for details†).

## Conclusions

In conclusion, we here have introduced an unusual reaction scheme in which a chiral rhodium complex enables the catalytic enantioselective functionalization of a photo-generated carbon radical employing cheap and readily available Hantzsch esters as a photoredox mediator and reductant. Intriguingly, in this radical allylation reaction using allyl sulfones as reagents, the generated sulfonyl radical by-product can be trapped by electron deficient alkenes and transformed into valuable enantioenriched S-containing building blocks thereby minimizing waste generation. The simple reaction setup and the mild reaction conditions as well as the demonstrated compatibility with a wide range of functionalities render this robust catalytic system an appealing process. Further investigations on the stereocontrolled chemistry of prochiral radicals are ongoing in our laboratory.^23^

## Conflicts of interest

There are no conflicts to declare.
