# Supplementary material for: Combining the catalytic enantioselective reaction of visible-light-generated radicals with a by-product utilization system
Source: Chem Sci. 2017 Sep 1;8(10):7126–31. doi: 10.1039/c7sc02621h (PMC5637358; doi:10.1039/c7sc02621h)
Supplement: Supplementary file 1 [file SC-008-C7SC02621H-s001.pdf]

Electronic Supplementary Material (ESI) for Chemical Science.
This journal is © The Royal Society of Chemistry 2017

Electronic Supporting Information for:

Combining the Catalytic Enantioselective Reaction of Visible-Light-Generated Radicals with a By-Product Utilization System

Xiaoqiang Huang, Shipeng Luo, Olaf Burghaus, Richard D. Webster, Klaus Harms, and Eric Meggers*

*meggers@chemie.uni-marburg.de

1. General Information **S2**

Light sources and emission spectra of the lamps S2

2. Modifications for the Synthesis of Λ/Δ-RhO **S4**

3. Synthesis of Substrates **S6**

4. General Procedure **S15**

5. Screening of Reaction Conditions **S16**

6. Mechanistic Studies **S18**

6.1 Identification of **RhO-1a** Intermediate S18

6.2 Control Experiments S18

6.3 Stern-Volmer Quenching Experiments S19

6.4 Cyclic Voltammetry S22

6.5 Evidence for the Formation of Sulfonyl Radical S23

6.6 Determination of the Quantum Yield S25

7. Experimental and Analytical Data of Products **S26**

8. Transformations of the Products **S42**

9. HPLC Traces of the Products **S45**

10. HPLC Traces of the Robustness Screen **S77**

11. Single-Crystal X-Ray Diffraction Studies **S95**

12. NMR Spectra of New Compounds **S101**

13. References **S185**

1. General Information

All catalytic reactions were carried out under an atmosphere of nitrogen with magnetic stirring in a Schlenk tube (10 mL). The catalysts Δ-**IrS**[1] and Δ-**RhS**[2] were synthesized according to our published procedures. Δ/Λ-**RhO** were synthesized with some modifications (see Section 2).[3] Solvents were distilled under nitrogen from calcium hydride (CH_3CN, CH_2Cl_2), sodium/benzophenone (THF, Et_2O). HPLC grade of acetone, methanol, and ethanol was used without further purification. Dry 1,4-dioxane was bought from Alfa-Aesar. Reagents that were purchased from commercial suppliers were used without further purification. Flash column chromatography was performed with silica gel 60 M from Macherey-Nagel (irregular shaped, 230-400 mesh, pH 6.8, pore volume: 0.81 mL × g^{-1}, mean pore size: 66 Å, specific surface: 492 m^2 × g^{-1}, particle size distribution: 0.5% < 25 μm and 1.7% > 71 μm, water content: 1.6%). ^{1}H NMR, ^{19}F NMR and proton decoupled ^{13}C NMR spectra were recorded on Bruker Avance 300 (300 MHz), or Bruker AM (500 MHz) spectrometers at ambient temperature. NMR standards were used as follows: ^{1}H NMR spectroscopy: δ = 7.26 ppm ($CDCl_3$). ^{19}F NMR spectroscopy: δ = 0 ppm ($CFCl_3$). ^{13}C NMR spectroscopy: δ = 77.0 ppm ($CDCl_3$). IR spectra were recorded on a Bruker Alpha FT-IR spectrophotometer. High-resolution mass spectra were recorded on a Bruker En Apex Ultra 7.0 TFT-MS instrument using ESI technique. HPLC chromatography on chiral stationary phase was performed with an Agilent 1200 or Agilent 1260 HPLC system. Optical rotations were measured on a Krüss P8000-T polarimeter with $[\alpha]_D^{22}$ values reported in degrees with concentrations reported in g/100 mL. The EPR spectrometer is from Bruker (model esp300), with a modified Varian rectangular X-band cavity and the modulation frequency was set to 100 kHz, the modulation amplitude was 0.1 mT. The Stern-Volmer quenching experiments were recorded on a Spectra Max M5 microplate reader in a 10.0 mm quartz cuvette.

Light sources and emission spectra of the lamps

A 21 W compact fluorescent lamp (CFL, OSRAM DULUX® SUPERSTAR Micro Twist) or 24 W Blue LEDs (Hongchangzhaoming from Chinese Taobao, https://hongchang-led.taobao.com) served as light sources. Figures S1 and S2 display their emission spectra.

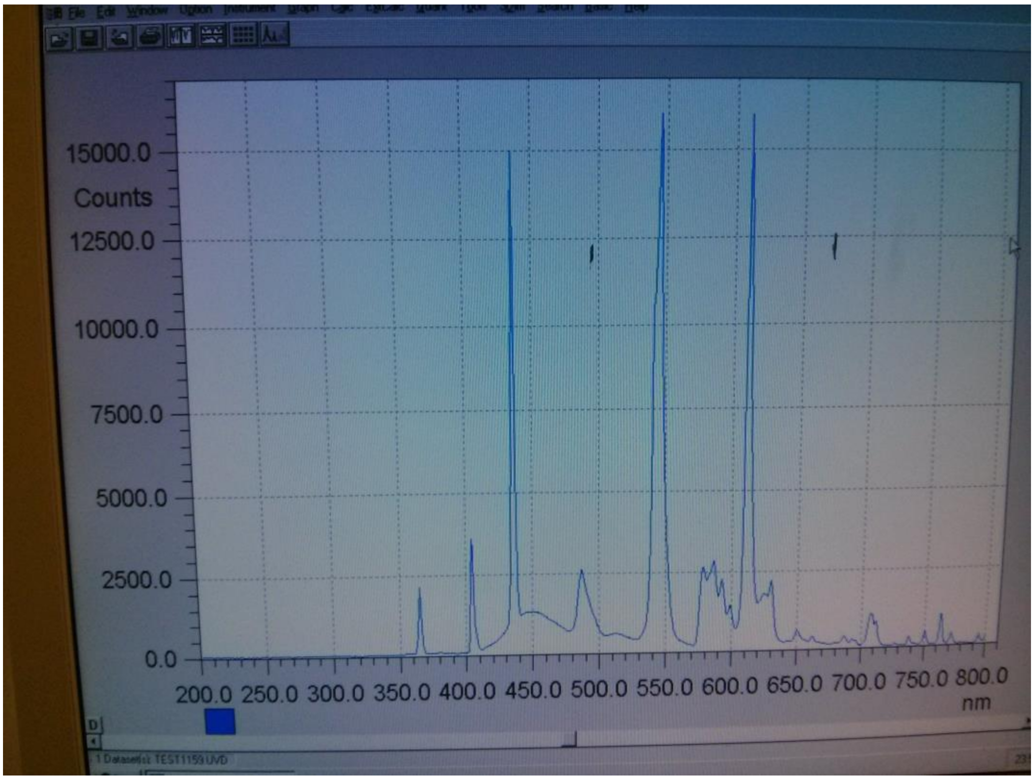

Figure S1. Emission spectrum of the 21 W CFL lamp.

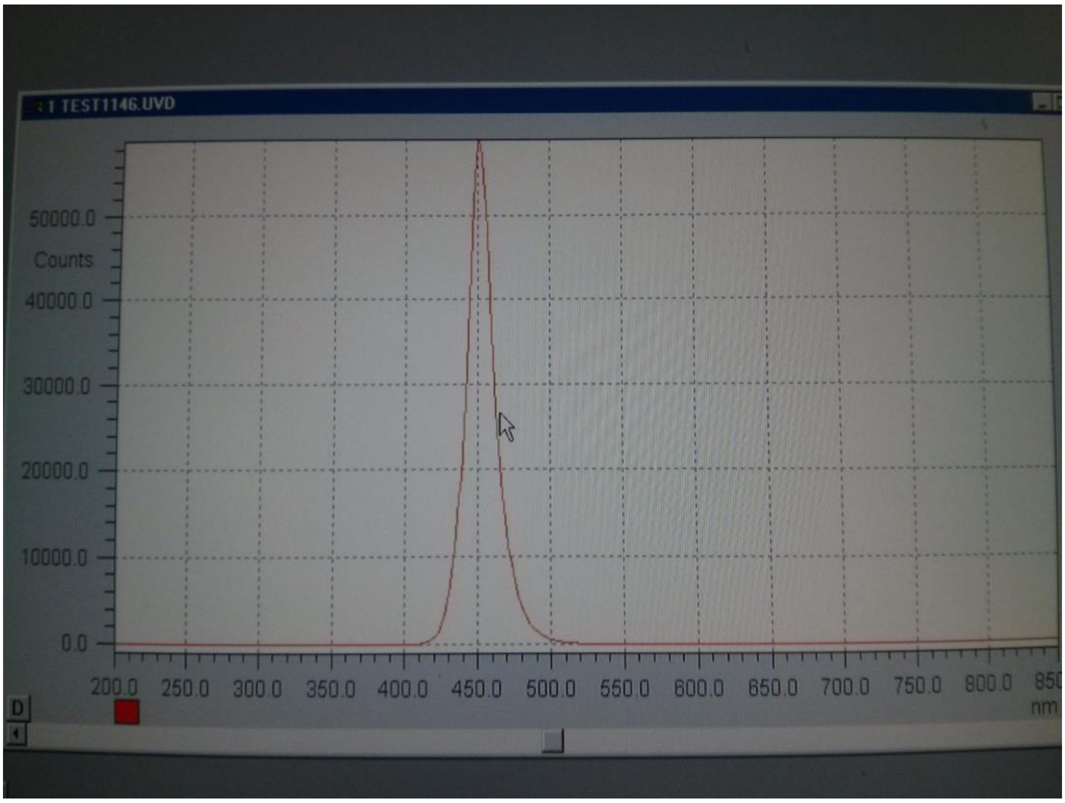

Figure S2. Emission spectrum of the 24 W blue LEDs.

2. Modifications for the Synthesis of Λ/Δ-RhO

Racemic **RhO** complex was synthesized according to our previous procedures,[3] in which the enantiopure **RhO** was obtained through a proline-mediated route resulting in a loss of at least 50% of rhodium complex. Herein, we modified the resolution process using a chiral auxiliary **(*R*)-Aux**, namely (*R*)-3-fluoro-2-(4-phenyl-4,5-dihydrooxazol-2-yl)phenol, instead of proline. The corresponding complexes **Λ/Δ-(*R*)-RhO** are stable and could be separated by flash chromatography, thus improving the atom economy of the catalyst synthesis.

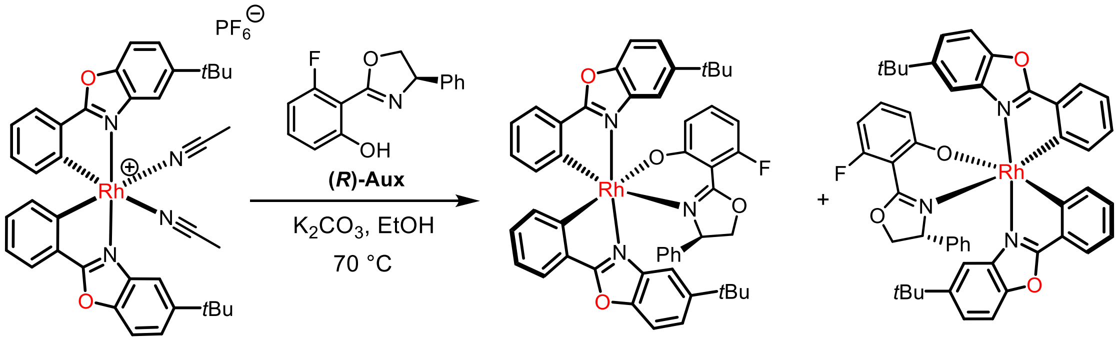

rac-**RhO** Λ-(*R*)-RhO Δ-(*R*)-RhO

To the mixture of *rac*-**RhO** (249 mg, 0.3 mmol) and K_2CO_3 (82.8 mg, 0.6 mmol) in absolute ethanol (6.0 mL) was added (*R*)-3-fluoro-2-(4-phenyl-4,5-dihydrooxazol-2-yl)phenol (**(*R*)-Aux**, 91 mg, 0.33 mmol) in one portion, After stirring at 70 °C overnight, the reaction mixture was cooled to room temperature and concentrated to dryness. The residue was directly subjected to a flash chromatography on silica gel (EtOAc/n-hexane = 1/100 to 1:10) giving **Δ-(*R*)-RhO** (117 mg, 45% yield) as a yellow solid and **Λ-(*R*)-RhO** (112 mg, 43% yield) as a yellow solid, respectively. The bis-acetonitrile catalysts could be obtained after removing of auxiliary following our previously reported procedures.[2]

Δ-(*R*)-RhO

^{1}H NMR (500 MHz, CD_2Cl_2) δ 8.02-7.98 (m, 1H), 7.72-7.68 (m, 1H), 7.62-7.58 (m, 1H), 7.55-7.51 (m, 2H), 7.46 (dd, J = 8.8, 2.0 Hz, 1H), 7.36 (d, J = 8.8 Hz, 1H), 7.31-7.27 (m, 1H), 7.02-6.94 (m, 3H), 6.92-6.84 (m, 3H), 6.67 (d, J = 7.6 Hz, 1H), 6.64-6.48 (m, 3H), 6.37-6.33 (m, 1H), 6.21 (d, J = 7.6 Hz, 2H), 5.96 (ddd, J = 12.8, 7.8, 0.8 Hz, 1H), 4.92 (dd, J = 9.7, 4.6 Hz, 1H), 4.83 (dd, J = 9.7, 9.0 Hz, 1H), 4.11 (dd, J = 9.0, 4.6 Hz, 1H), 1.44 (s, 9H), 1.32 (s, 9H).

^{13}C NMR (125 MHz, CD_2Cl_2) δ 175.23, 175.21, 172.0, 171.9, 170.54, 170.50, 169.3, 169.1, 167.1,

166.9, 165.44, 165.41, 164.1 (d, *J* = 257.5 Hz), 150.1, 150.0, 148.60, 148.56, 141.3, 139.1, 138.6, 134.5, 133.4, 133.3, 133.2, 131.9, 131.4, 131.11, 131.10, 130.8, 130.7, 127.9, 127.5 (2C), 125.7, 125.6, 123.7, 123.3, 123.1, 122.4, 120.60, 120.56, 115.3, 113.0, 111.3, 110.5, 100.2 (d, *J* = 6.4 Hz), 98.7 (d, *J* = 24.1 Hz), 75.0, 69.9, 35.5, 35.4, 31.81, 31.80.

IR (film): *v* (cm^{-1}) 2958, 2904, 2869, 1622, 1589, 1528, 1478, 1445, 1374, 1273, 1224, 1092, 1031, 930, 813, 782, 732, 698, 529, 455,

HRMS (ESI, *m*/*z*) calcd for $C_{49}H_{44}FN_3O_4Rh$ $[M+H]^+$: 860.2365, found: 860.2357.

Λ-(*R*)-RhO

^{1}H NMR (500 MHz, CD_2Cl_2) δ 8.15-8.11 (m, 1H), 7.74-7.44 (m, 7H), 7.00-6.94 (m, 9H), 6.46-6.28 (m, 3H), 6.04-5.90 (m, 2H), 4.37 (dd, *J* = 9.3, 7.8 Hz, 1H), 4.24 (dd, *J* = 16.8, 8.2 Hz, 1H), 4.21 (dd, *J* = 16.8, 8.2 Hz, 1H), 1.43 (s, 9H), 1.30 (s, 9H).

^{13}C NMR (125 MHz, CD_2Cl_2) δ 175.03, 175.00, 172.0, 171.9, 171.81, 171.79, 168.5, 168.2, 168.0, 167.7, 165.51, 165.50, 163.2 (d, *J* = 253.9 Hz), 150.6, 149.6, 149.0, 148.2, 141.3, 139.1, 138,6, 135.6, 133.3, 132.6, 132.5, 131.3, 131.2, 130.5, 130.2, 128.5, 127.4, 127.2, 125.53, 125.50, 123.9, 123.5, 123.0, 122.3, 119.62, 119.60, 115.2, 114.2, 110.9, 110.8, 102.6 (d, *J* = 8.3 Hz), 98.5 (d, *J* = 18.5 Hz), 75.6, 69.3, 35.44, 35.37, 31.8, 31.6.

IR (film): *v* (cm^{-1}) 2958, 2901, 2870, 1623, 1589, 1528, 1478, 1444, 1373, 1273, 1224, 1092, 1030, 979, 931, 814, 783, 733, 698, 529, 456.

HRMS (ESI, *m*/*z*) calcd for $C_{49}H_{44}FN_3O_4Rh$ $[M+H]^+$: 860.2365, found: 860.2357.

3. Synthesis of Substrates

3.1 General procedure A

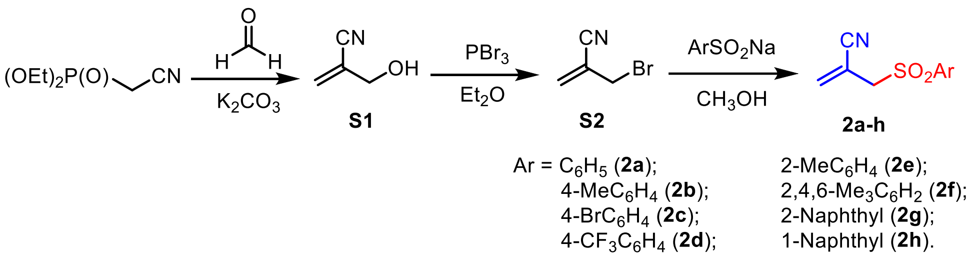

To a mixture of diethyl (cyanomethyl)phosphonate (20 mmol) and a 37% aqueous solution of formaldehyde (80 mmol), a saturated aqueous solution of potassium carbonate (37.5 mmol) was added at room temperature dropwise over 30 min. After stirring for an additional 2 h, the reaction was quenched with saturated aqueous ammonium chloride (20 mL). Afterwards, the reaction mixture was extracted with diethyl ether (3 × 12.5 mL). The organic layers were combined and dried over sodium sulfate. The solvent was evaporated using a rotary evaporator, and the remaining colorless oil was purified by flash chromatography using pentane/CH_2Cl_2 (2/1) giving the pure product **S1** as a colorless oil (70% yeild).

To a solution of **S1** (14 mmol) in dry ether (20 mL) was added phosphorus(III) bromide (5 mmol) at –10 °C. The temperature was allowed to rise to 20 °C and stirring was continued for 3 h. Water (10 mL) was then added and the mixture was extracted with diethyl ether (3 × 30 mL). The organic phase was washed with brine (20 mL), dried with sodium sulfate and concentrated under reduced pressure. The crude product was purified by column chromatography on silica gel (pentane/ CH_2Cl_2, 1/1) to give **S2** as a colorless oil (89% yield).

To a solution of **S2** (2.0 mmol) in methanol (5 mL) was added corresponding sodium aryl sulfinate (3.0 mmol). After 2.5 h of reflux, the mixture was concentrated under reduced pressure, the thereby obtained residue was dissolved in EtOAc and the mixture was washed with water, brine, dried with Na_2SO_4, filtered and the filtrate was evaporated and purified by chromatography (EtOAc/n-hexane, 1/1) to give corresponding products **2a-h**. The characteristic data of **2a** are in accord with literature.[4]

3.2 General procedure B

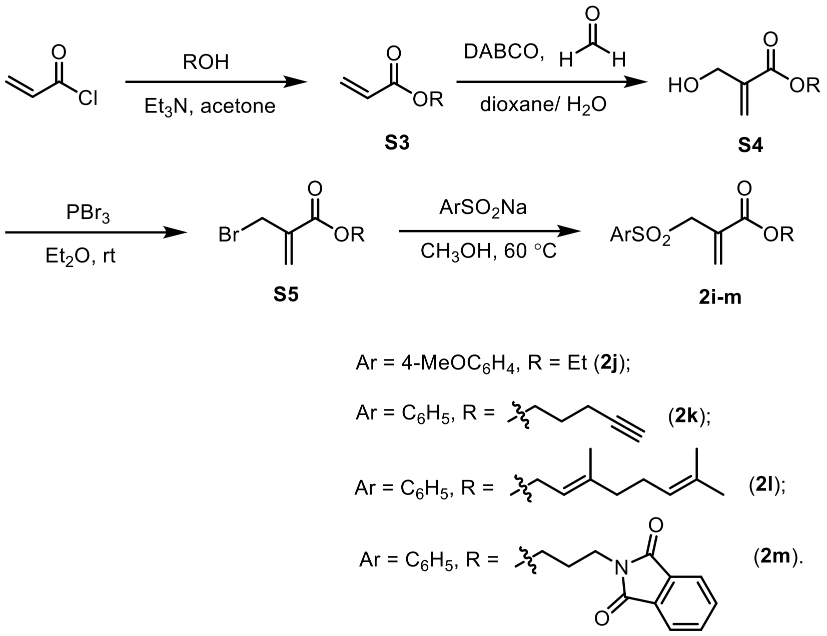

To a solution of corresponding alcohol (ROH, 10 mmol) and triethylamine (15 mmol) in acetone (15 mL) was added acryloyl chloride (13 mmol) dropwise at 0 °C. After stirring at 0 °C for 30 min, the reaction mixture was warmed to room temperature and stirred for additional 5 h. The resulting mixture was concentrated, then taken up in EtOAc (50 mL) and washed with brine (3 × 10 mL). The organic extracts were dried over anhydrous Na_2SO_4, concentrated by rotary evaporation. Purification by column chromatography (n-hexane/EtOAc, 9/1) afforded corresponding ester **S3**.

To a solution of a 37% aqueous solution of formaldehyde (7.0 mmol) and ester **S3** (5 mmol) in 5 mL 1,4-dioxane-water (1:1, v/v) was added DABCO (7.0 mmol) and the reaction progress was monitored by TLC. Upon completion, the reaction mixture was partitioned with EtOAc (50 mL) and water (20 mL). The organic layer was separated and washed with brine (5 mL), dried over anhydrous Na_2SO_4 and concentrated under reduced pressure. The crude product was purified by column chromatography on silica gel (EtOAc/n-hexane, 1/1) to afford corresponding alcohol ester **S4**.

To a solution of **S4** (5 mmol) in dry ether (10 mL) was added phosphorus(III) bromide (1.7 mmol) dropwise at –10 °C. The temperature was allowed to rise to 20 °C and stirring was continued for 3 h. Water (20 mL) was then added and the mixture was extracted with diethyl ether (3 × 10 mL). The organic phase was washed with saturated sodium chloride solution (5 mL), dried with sodium sulfate and concentrated under reduced pressure. The crude product was purified by column chromatography on silica gel (pentane/CH_2Cl_2, 1/1) to give corresponding brominated compound

S5.

To a solution of **S5** (2.0 mmol) in methanol (5 mL) was added corresponding sodium aryl sulfinate (3.0 mmol). After 2.5 h of reflux, the mixture was concentrated under reduced pressure, the thereby obtained residue was dissolved in EtOAc and the mixture was washed with water, brine, dried with Na_2SO_4, filtered and the filtrate was evaporated and purified by chromatography (EtOAc/n-hexane, 1/1) to give corresponding products **2j-m**.

3.3 General procedure C

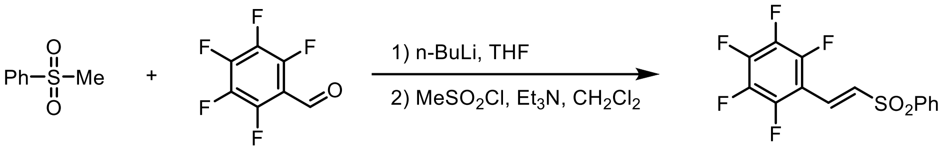

5

To a solution of methyl phenyl sulfone (1.25 g, 8.0 mmol) in THF (40 mL) cooled at –78 ºC, n-BuLi (1.6 M in n-hexane, 5.5 mL, 8.8 mmol) was added dropwise under argon atmosphere. The resulting solution was stirred at 0 ºC for 30 min, and then cooled back to –78 ºC. A solution of 2,3,4,5,6-pentafluorobenzaldehyde (1.72g, 8.8 mmol) in THF (2.0 mL) was added dropwise and the temperature was allowed to slowly raise to room temperature, and the solution was stirred until methylphenylsulfone disappeared by TLC. A saturated aqueous solution of NH_4Cl (20 mL) was added, the organic layer was separated and the aqueous layer was extracted with CH_2Cl_2 (3 × 10 mL). The combined organic layers were dried with Na_2SO_4 and evaporated under reduced pressure.

Without further purification, the resulting alcohol was dissolved in dry CH_2Cl_2 (25 mL) under argon atmosphere, cooled to 0 ºC, then Et_3N (11.2 mL, 80 mmol) and methanesulfonyl chloride (0.93 mL, 12 mmol) were added continuously. After stirring at room temperature for 90 min, a saturated aqueous solution of NH_4Cl (30 mL) was added, the organic layer was separated and the aqueous layer was extracted with CH_2Cl_2 (3 × 15 mL). The combined organic layers were dried (Na_2SO_4) and the solvent was evaporated. The residue was purified by flash chromatography (n-hexane/EtOAc, 5/1) to afford compound **5** (1.73g, 65%) as a white solid.

3.4 Analytical data of unreported starting materials

2-(Tosylmethyl)acrylonitrile (2b)

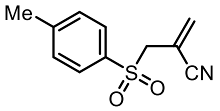

According to the general procedure **A**, compound **2b** was obtained as a white solid.

^{1}H NMR (300 MHz, $CDCl_3$) δ 7.83-7.76 (m, 2H), 7.43-7.36 (m, 2H), 6.20 (s, 1H), 5.98 (s, 1H), 3.91 (d, J = 0.8 Hz, 2H), 2.46 (s, 3H).

^{13}C NMR (75 MHz, $CDCl_3$) δ 145.9, 139.3, 134.4, 130.1, 128.7, 116.5, 111.6, 59.8, 21.7.

IR (film): ν (cm^{-1}) 2985, 2226, 1315, 1288, 1144, 1084, 975, 813, 772, 676, 608, 580, 524.

HRMS (ESI, *m*/*z*) calcd for $C_{11}H_{11}NO_2SNa$ [M+Na]$^+$: 244.0403, found: 244.0398.

2-(((4-Bromophenyl)sulfonyl)methyl)acrylonitrile (2c)

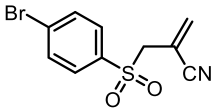

According to the general procedure **A**, compound **2c** was obtained as a white solid.

^{1}H NMR (300 MHz, $CDCl_3$) δ 8.13-7.85 (m, 4H), 6.26 (s, 1H), 6.05 (s, 1H), 3.98 (d, J = 0.8 Hz, 2H).

^{13}C NMR (75 MHz, $CDCl_3$) δ 139.8, 139.7, 136.3, 132.9, 130.2, 116.4, 111.2, 59.8.

IR (film): ν (cm^{-1}) 2929, 2222, 1569, 1385, 1314, 1281, 1241, 1151, 1130, 1073, 1006, 968, 902, 819, 790, 709, 617, 579.

HRMS (ESI, *m*/*z*) calcd for $C_{10}H_8NO_2SNa$ [M+Na]$^+$: 307.9351, found: 307.9347.

2-(((4-(Trifluoromethyl)phenyl)sulfonyl)methyl)acrylonitrile (2d)

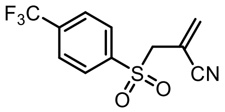

According to the general procedure **A**, compound **2d** was obtained as a white solid.

^{1}H NMR (300 MHz, $CDCl_3$) δ 8.08 (d, J = 8.2 Hz, 2H), 7.89 (d, J = 8.2 Hz, 2H), 6.26 (s, 1H), 6.05 (t, J = 0.8 Hz, 1H), 3.98 (d, J = 0.8 Hz, 2H).

^{13}C NMR (75 MHz, $CDCl_3$) δ 140.9, 140.0, 136.4 (q, J = 33.2 Hz), 129.4, 126.7 (q, J = 3.6 Hz), 122.9 (q, J = 273.2 Hz), 116.2, 111.0, 59.8.

^{19}F NMR (282 MHz, $CDCl_3$) δ –63.30 (s, 3F).

IR (film): ν (cm^{-1}) 2986, 2926, 2232, 1406, 1320, 1247, 1161, 1141, 1086, 1060, 1015, 972, 905, 844, 799, 698, 582.

HRMS (ESI, *m*/*z*) calcd for $C_{11}H_8F_3NO_2SNa$ $[M+Na]^+$: 298.0120, found: 298.0114.

2-((*o*-Tolylsulfonyl)methyl)acrylonitrile (2e)

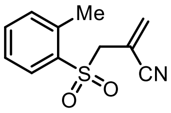

According to the general procedure **A**, compound **2e** was obtained as a white solid.

^1H NMR (300 MHz, $CDCl_3$) δ 7.97 (d, J = 8.0 Hz, 1H), 7.62-7.53 (m, 1H), 7.44-7.36 (m, 2H), 6.18 (s, 1H), 6.00 (s, 1H), 3.96 (s, 2H), 2.73 (s, 3H).

^{13}C NMR (75 MHz, $CDCl_3$) δ 139.3, 138.4, 135.5, 134.6, 133.0, 131.1, 126.8, 116.4, 111.3, 58.9, 20.4.

IR (film): ν (cm^{-1}) 2991, 2936, 2229, 1594, 1454, 1404, 1314, 1285, 1243, 1199, 1153, 1122, 1058, 960, 897, 806, 761, 698, 579.

HRMS (ESI, *m*/*z*) calcd for $C_{11}H_{11}NO_2SNa$ $[M+Na]^+$: 244.0403, found: 244.0398.

2-((Mesitylsulfonyl)methyl)acrylonitrile (2f)

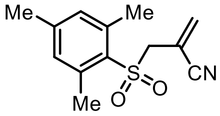

According to the general procedure **A**, compound **2f** was obtained as a white solid.

^1H NMR (300 MHz, $CDCl_3$) δ 7.00 (s, 2H), 6.20 (s, 1H), 6.00 (d, J = 0.8 Hz, 1H), 3.92 (d, J = 0.8 Hz, 2H), 2.66 (s, 6H), 2.33 (s, 3H).

^{13}C NMR (75 MHz, $CDCl_3$) δ 144.5, 140.5, 139.0, 132.5, 131.5, 116.6, 111.4, 59.5, 23.0, 21.1.

IR (film): ν (cm^{-1}) 2985, 2220, 1593, 1454, 1308, 3183, 1272, 1143, 971, 670, 605, 572.

HRMS (ESI, *m*/*z*) calcd for $C_{13}H_{15}NO_2SNa$ $[M+Na]^+$: 272.0716, found: 272.0710.

2-((Naphthalen-2-ylsulfonyl)methyl)acrylonitrile (2g)

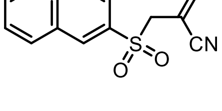

According to the general procedure **A**, compound **2g** was obtained as a white solid.

^{1}H NMR (300 MHz, $CDCl_3$) δ 8.75 (d, J = 8.4 Hz, 1H), 8.34-8.26 (m, 1H), 8.19 (d, J = 8.2 Hz, 1H), 8.01 (d, J = 8.0 Hz, 1H), 7.82-7.56 (m, 3H), 6.11 (s, 1H), 5.84 (s, 1H), 4.12 (s, 2H).

^{13}C NMR (75 MHz, $CDCl_3$) δ 139.1, 136.2, 134.2, 132.1, 132.0, 129.5, 129.2, 128.8, 127.2, 124.3, 123.4, 116.2, 111.3, 59.2.

IR (film): ν (cm^{-1}) 2929, 2234, 2186, 1505, 1311, 1256, 1164, 1114, 977, 905, 800, 770, 737, 640, 573, 538, 474.

HRMS (ESI, m/z) calcd for $C_{14}H_{11}NO_2SNa$ $[M+Na]^+$: 280.0403, found: 280.0396.

2-((Naphthalen-1-ylsulfonyl)methyl)acrylonitrile (2h)

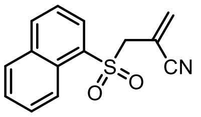

According to the general procedure **A**, compound **2h** was obtained as a white solid.

^{1}H NMR (300 MHz, $CDCl_3$) δ 8.51 (s, 1H), 8.10-7.84 (m, 4H), 7.77-7.59 (m, 2H), 6.20 (s, 1H), 5.97 (s, 1H), 4.01 (s, 2H).

^{13}C NMR (75 MHz, $CDCl_3$) δ 139.4, 135.7, 134.2, 132.0, 131.0, 129.9, 129.8, 129.6, 128.1, 128.0, 122.7, 116.5, 111.5, 59.8.

IR (film): ν (cm^{-1}) 2992, 2937, 2229, 1592, 1454, 1403, 1314, 1286, 1242, 1200, 1153, 1123, 1063, 959, 897, 761, 679, 578.

HRMS (ESI, m/z) calcd for $C_{14}H_{11}NO_2SNa$ $[M+Na]^+$: 280.0403, found: 280.0398.

Ethyl 2-(((4-methoxyphenyl)sulfonyl)methyl)acrylate (2j)

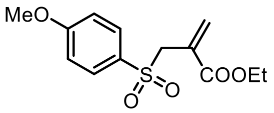

According to the general procedure **B**, compound **2j** was obtained as a colorless oil.

^{1}H NMR (300 MHz, $CDCl_3$) δ 7.78-7.72 (m, 2H), 7.00-6.94 (m, 2H), 6.47 (s, 1H), 5.88 (s, 1H), 4.12 (s, 2H), 4.03 (q, J = 7.1 Hz, 2H), 3.86 (s, 3H), 1.17 (t, J = 7.1 Hz, 3H).

^{13}C NMR (75 MHz, $CDCl_3$) δ 164.8, 163.8, 133.0, 130.9, 130.0, 129.4, 114.2, 61.4, 57.7, 55.6, 14.0.

IR (film): ν (cm^{-1}) 2984, 1716, 1591, 1496, 1463, 1413, 1308, 1252, 1187, 1139, 1086, 1020, 962,

899, 837, 778, 670, 526.

HRMS (ESI, *m*/*z*) calcd for $C_{13}H_{16}O_5SNa$ $[M+Na]^+$: 307.0611, found: 307.0604.

Pent-4-yn-1-yl 2-((phenylsulfonyl)methyl)acrylate (2k)

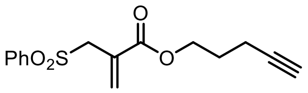

According to the general procedure **B**, compound **2k** was obtained as a white solid.

^{1}H NMR (300 MHz, $CDCl_3$) δ 7.90-7.84 (m, 2H), 7.68-7.62 (m, 1H), 7.59-7.51 (m, 2H), 6.51 (s, 1H), 5.92 (s, 1H), 4.16 (s, 2H), 4.09 (t, J = 6.3 Hz, 2H), 2.24 (td, J = 7.0, 2.6 Hz, 2H), 1.98 (t, 2.6 Hz, J = 1H), 1.84-1.74 (m, 2H).

^{13}C NMR (75 MHz, $CDCl_3$) δ 164.7, 138.4, 133.9, 133.5, 129.1, 129.0, 128.7, 82.8, 69.1, 64.0, 57.5, 27.4, 15.2.

IR (film): *v* (cm^{-1}) 3262, 1705, 1626, 1292, 1245, 1142, 1080,749, 684, 523, 480.

HRMS (ESI, *m*/*z*) calcd for $C_{15}H_{16}O_4SNa$ $[M+Na]^+$: 315.0662, found: 315.0655.

(*E*)-3,7-Dimethylocta-2,6-dien-1-yl 2-((phenylsulfonyl)methyl)acrylate (2l)

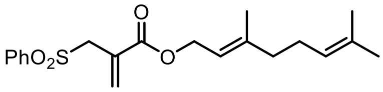

According to the general procedure **B**, compound **2l** was obtained as a colorless oil (*contains about 5% of Z-isomer derived from geraniol*).

^{1}H NMR (300 MHz, $CDCl_3$) δ 7.90-7.84 (m, 2H), 7.67-7.59 (m, 1H), 7.57-7.49 (m, 2H), 6.51 (s, 1H), 5.94 (s, 1H), 5.25-5.17 (m, 1H), 5.12-5.04 (m, 1H), 4.46 (d, J = 7.1 Hz, 2H), 4.16 (s, 2H), 2.13-1.95 (m, 4H), 1.69 (s, 3H), 1.66 (s, 3H), 1.61 (s, 3H).

^{13}C NMR (75 MHz, $CDCl_3$) δ 164.8, 142.6, 138.5, 133.8, 133.3, 131.9, 129.2, 129.0, 128.8, 123.6, 117.8, 62.4, 57.5, 39.5, 26.3, 25.7, 17.7, 16.5.

IR (film): *v* (cm^{-1}) 2926, 1718, 1446, 1385, 1313, 1245, 1182, 1145, 1084, 965, 892, 809, 754, 660, 523.

HRMS (ESI, *m*/*z*) calcd for $C_{20}H_{26}O_4SNa$ $[M+Na]^+$: 385.1444, found: 385.1427.

3-(1,3-Dioxoisoindolin-2-yl)propyl 2-((phenylsulfonyl)methyl)acrylate (2m)

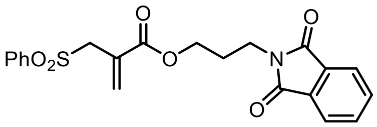

According to the general procedure **B**, compound **2m** was obtained as a white solid.

^{1}H NMR (300 MHz, $CDCl_3$) δ 7.90-7.82 (m, 4H), 7.77-7.69 (m, 2H), 7.66-7.49 (m, 3H), 6.49 (s, 1H), 5.89 (s, 1H), 4.14 (s, 2H), 4.02 (t, *J* = 6.2 Hz, 2H), 3.76 (t, *J* = 6.9 Hz, 2H), 2.02-1.92 (m, 2H).

^{13}C NMR (75 MHz, $CDCl_3$) δ 168.2, 164.6, 138.5, 134.1, 133.8, 133.7, 132.0, 129.1, 128.8, 123.3, 62.7, 57.5, 34.8, 27.5.

IR (film): *v* (cm^{-1}) 2959, 1707, 1445, 1403, 1375, 1307, 1199, 1161, 1083, 1054, 969, 921, 791, 755, 681, 607, 558.

HRMS (ESI, *m*/*z*) calcd for $C_{21}H_{19}NO_6SNa$ $[M+Na]^+$: 436.0825, found: 436.0828.

(*E*)-1-(3,5-Dimethyl-1*H*-pyrazol-1-yl)hepta-2,6-dien-1-one (1d)

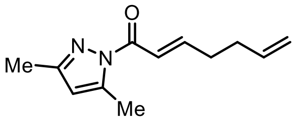

According to the reported precedures,[5] compound **1d** was obtained as a colorless liquid.

^{1}H NMR (300 MHz, $CDCl_3$) δ 7.40-7.16 (m, 2H), 6.00 (s, 1H), 5,95-5.75 (m, 1H), 5.14-5.00 (m, 2H), 2.60 (s, 3H), 2.51-2.41 (m, 2H), 2.36-2.24 (m, 2H), 2.28 (s, 3H).

^{13}C NMR (75 MHz, $CDCl_3$) δ 165.1, 151.8, 150.4, 144.4, 137.1, 121.5, 115.5, 111.2, 32.2, 32.0, 14.6, 13.8.

IR (film): *v* (cm^{-1}) 2927, 1705, 1639, 1581, 1476, 1411, 1376, 1344, 1294, 1243, 1139, 960, 914, 805, 753, 700, 626, 586, 407.

HRMS (ESI, *m*/*z*) calcd for $C_{12}H_{16}N_2ONa$ $[M+Na]^+$: 227.1155, found: 227.1155.

(*E*)-1,2,3,4,5-Pentafluoro-6-(2-(phenylsulfonyl)vinyl)benzene (5)

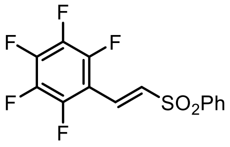

According to the general procedure C, compound **5** was obtained as a white solid.

^{1}H NMR (500 MHz, $CDCl_3$) δ 7.98-7.92 (m, 2H), 7.73-7.65 (m, 2H), 7.63-7.55 (m, 2H), 7.22 (d, *J*

= 16.8 Hz, 1H).

^{13}C NMR (125 MHz, $CDCl_3$) δ 147.6-147.2 (m), 144.2-143.8 (m), 140.8-140.2 (m), 139.5, 136.4-135.8 (m), 135.5-135.1 (m), 134.0, 129.6, 128.0, 125.9-125.7 (m), 108.4-107.8 (m).

^{19}F NMR (282 MHz, $CDCl_3$) δ –138.10 - –138.32 (m, 2F), –148.58 - –148.82 (m, 1F), –160.56 - –160.84 (m, 2F).

IR (film): v (cm^{-1}) 1650, 1495, 1419, 1301, 1146, 1082, 1001, 963, 845, 816, 749, 685, 550.

HRMS (ESI, *m*/*z*) calcd for $C_{14}H_7F_5O_2SNa$ $[M+Na]^+$: 356.9979, found: 356.9970.

4. General Procedure

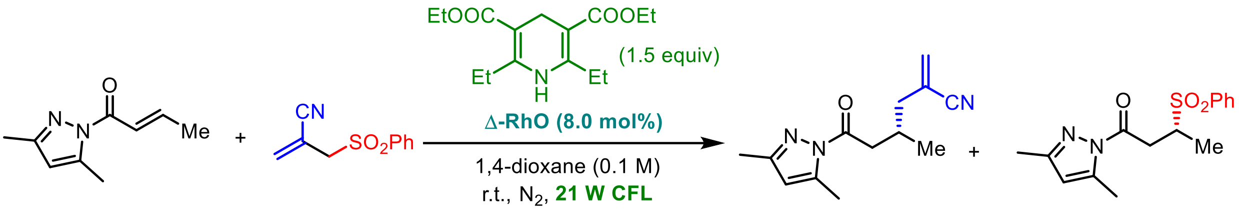

A dried 10 mL Schlenk tube was charged with **2a** (20.7 mg, 0.10 mmol), **Δ-RhO** (6.6 mg, 8.0 mol%) and **HE-1** (42.2 mg, 0.15 mmol, synthesized following a reported procedure[6]). The tube was purged with nitrogen for three times. Then, 1,4-dioxane (1.0 mL, 0.10 M, bubbling with nitrogen gas for five minutes before addition) was added via syringe followed by addition of **1a** (32.8 mg, 0.2 mmol) under nitrogen atmosphere. The tube was sealed and positioned approximately 5 cm away from a 21 W compact fluorescent lamp. The reaction was stirred at room temperature for the indicated time (monitored by TLC) under nitrogen atmosphere. Afterwards, the mixture was diluted with CH_2Cl_2. The combined organic layers were concentrated under reduced pressure. The residue was purified by flash chromatography on silica gel (n-hexane/EtOAc) to afford the products **3a** and **4a**. Racemic samples were obtained by carrying out the reactions with *rac*-**RhO**. The enantiomeric excess was determined by HPLC analysis on a chiral stationary phase.

5. Screening of Reaction Conditions

Table S1. Effect of Lewis acid catalysts[a]

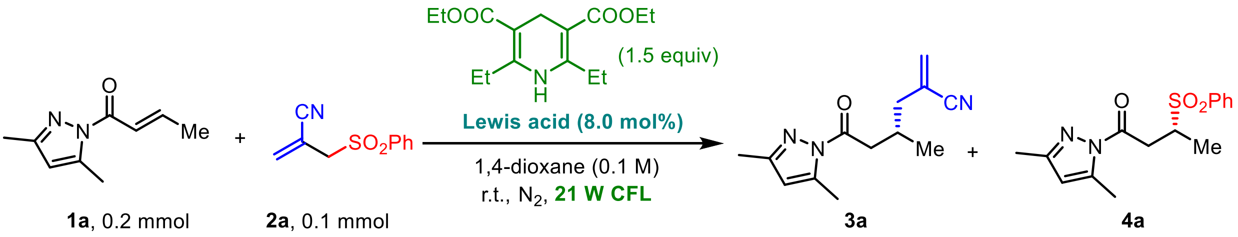

Entry	Lewis acid	3a	4a
1	Δ-**IrS**	< 5% yield	< 5% yield
2	Δ-**RhS**	92% yield; 83% ee	95% yield; 84% ee
**3**	**Δ-RhO**	**85% yield; 96% ee**	**92% yield; 85% ee**
4	Δ-**RhPP**[b]	74% yield; 40% ee	77% yield; 10% ee
5	$Sc(OTf)_3$[c]	10% yield	10% yield
6	$LiBF_4$[d]	0% yield	0% yield
7	none	0% yield	0% yield

[a] Reaction conditions: **1a** (0.20 mmol), **2a** (0.10 mmol), Lewis acid (8.0 mol%) and **HE-1** (0.15 mmol) in 1,4-dioxane (0.1 M) were stirred at room temperature for 24 h with a 21 W CFL. [b] **Δ-RhPP** was synthesized according to our previous report.[7] [c] (20 mol%) of $Sc(OTf)_3$ was employed. [d] (200 mol%) of $LiBF_4$ was employed.

Table S2. Variation of Hantzsch ester[a]

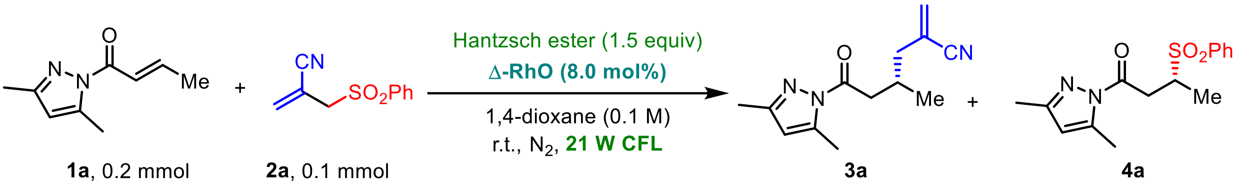

Entry	Hantzsch ester		3a	4a
1	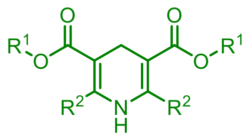	R^1 = Et; R^2 = Et (**HE-1**)	**85% yield; 96% ee**	**92% yield; 85% ee**
2		R^1 = Et; R^2 = Me (**HE-2**)	78% yield; 96% ee	82% yield; 80% ee
**3**		R^1 = *t*Bu; R^2 = Me (**HE-3**)	80% yield; 94% ee	85% yield; 85% ee

[a] Reaction conditions: **1a** (0.20 mmol), **2a** (0.10 mmol), **Δ-RhO** (8.0 mol%) and indicated Hantzsch ester (0.15 mmol) in 1,4-dioxane (0.1 M) were stirred at room temperature for 24 h with a 21 W CFL.

Table S3. Effect of different solvents[a]

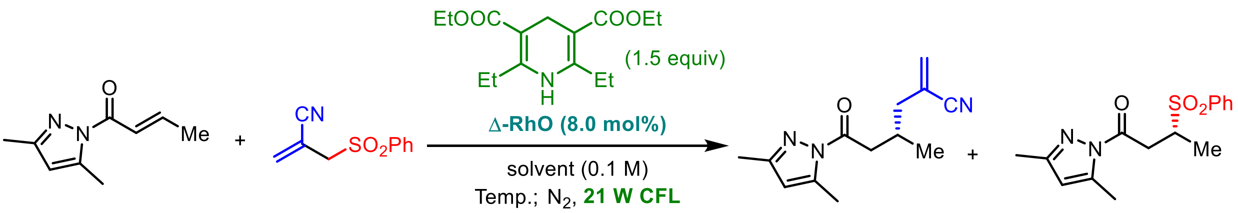

Entry	Solvent	Temp.	3a	4a
1	1,4-dioxane	r.t.	**85% yield; 96% ee**	**92% yield; 85% ee**
2	1,4-dioxane	35 °C	82% yield; 95% ee	90% yield; 86% ee
3	acetone	35 °C	81% yield; 89% ee	85% yield; 87% ee
4[b]	CH_2Cl_2	35 °C	81% yield; 92% ee	86% yield; 87% ee
5	THF	35 °C	82% yield; 94% ee	90% yield; 86% ee

[a] Reaction conditions: **1a** (0.10 mmol), **2a** (0.05 mmol), **Δ-RhO** (8.0 mol%) and **HE-1** (0.075 mmol) in indicated solvent (0.1 M) were stirred at room temperature for 24 h or 35 ºC for 15 h with a 21 W CFL. [b] The reduced product, 1-(3,5-dimethyl-1*H*-pyrazol-1-yl)butan-1-one[8], was detected in less than 5% yield.

Table S4. Effect of light source[a]

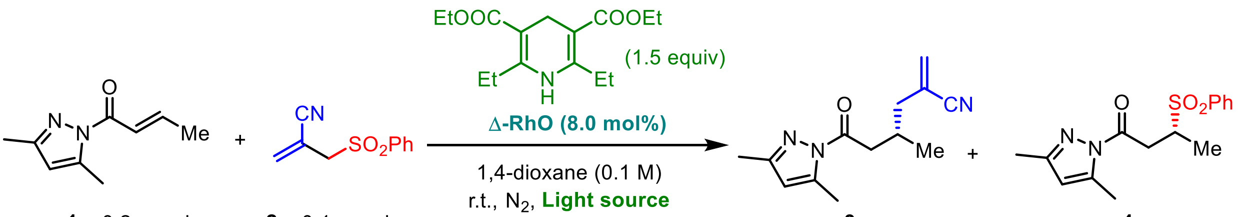

Entry	Light source	3a	4a
1	21 W CFL[b]	85% yield; 96% ee	92% yield; 85% ee
2	24 W Blue LEDs[c]	77% yield; 92% ee	80% yield; 80% ee

[a] Reaction conditions: **1a** (0.20 mmol), **2a** (0.10 mmol), **Δ-RhO** (8.0 mol%) and **HE-1** (0.15 mmol) in 1,4-dioxane (1.0 mL, 0.1 M) were stirred at room temperature for 24 h with the indicated light source. [b] See Figure S1 for emission spectrum. [c] See Figure S2 for emission spectrum.

6. Mechanistic Studies

6.1 Identification of RhO-1a Intermediate

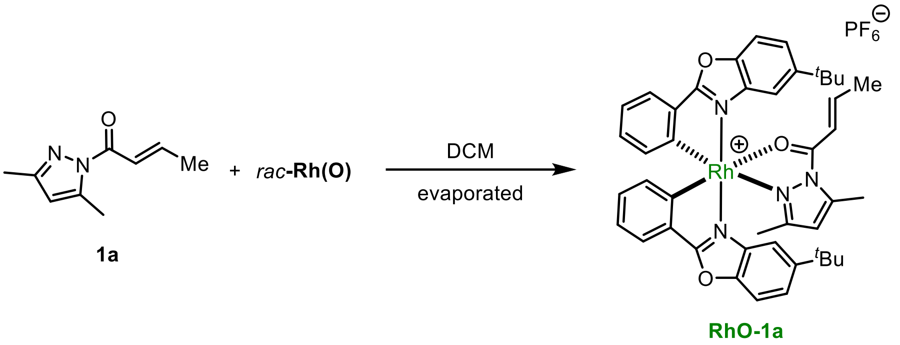

To a solution of *rac*-**RhO** (83.1 mg, 0.1 mmol) in CH_2Cl_2 (2 mL) was added α,β-unsaturated *N*-acylpyrazole **1a** (16.4 mg, 0.1 mmol). The mixture was stirred at room temperature for 1 minute and then the solvent was removed in vacuum. The procedure was repeated for another 3 times until the ligand exchange finished completely (detected by ^{1}H NMR). The resulting solid was recrystallized from CH_2Cl_2/Et_2O giving pure **RhO-1a**, which was characterized by single crystal X-ray diffraction (see Section 11).

6.2 Control Experiments

6.2.1 Using BHT as a radical trap

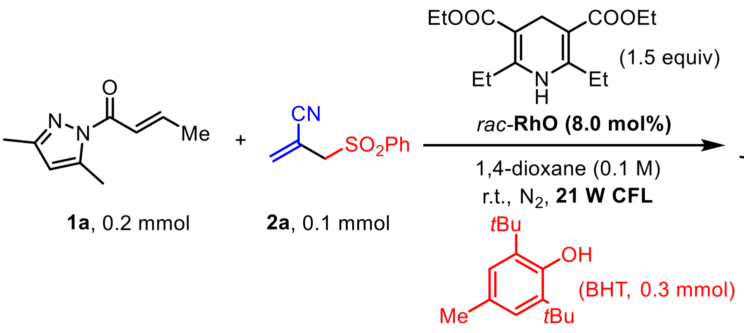

As shown above, when BHT (3.0 equiv) was added to the reaction **1a**+**2a**→**3a**+**4a** under standard conditions, the reaction was significantly inhibited delivering **3a** and **4a** in decreased yields.

6.2.2 Using TEMPO as a radical trap

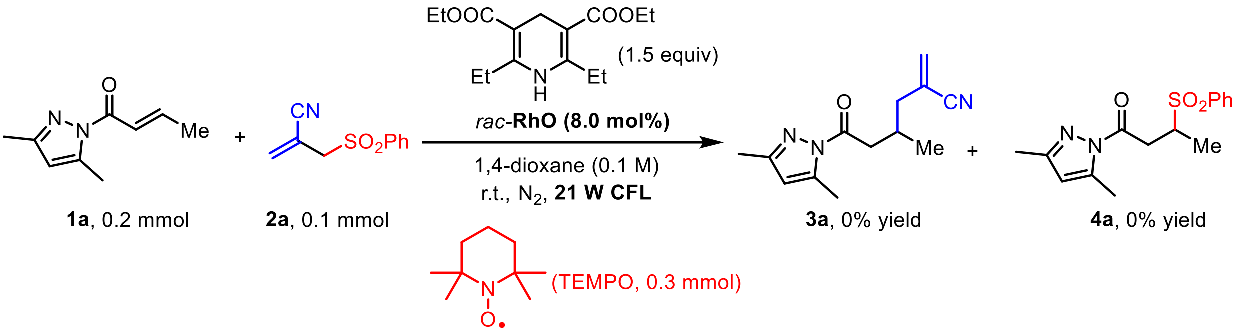

When TEMPO (3.0 equiv) was added to the reaction **1a**+**2a**→**3a**+**4a** under standard conditions, the reaction was completely inhibited.

6.2.3 Using 1,1-diphenyl ethylene a radical trap

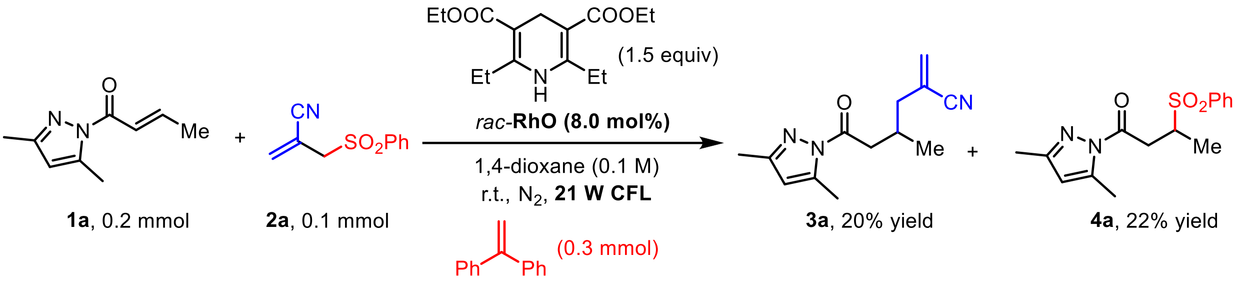

When the 1,1-diphenylethylene (3.0 equiv) was added to the reaction **1a**+**2a**→**3a**+**4a** under standard conditions, the reaction was partly inhibited and the yields of the products were decreased to 20% for **3a** and 22% for **4a**.

All these control experiments indicate that radical processes might be involved in the present transformation.

6.3 Stern-Volmer Quenching Experiments

6.3.1 UV-Vis absorption spectra and luminescence emission spectra

As shown in Figure S3, both **HE-1** and **RhO-1a** absorb visible light with wavelength < 425 nm. In order to simulate the reaction conditions, the luminescence quenching experiments were performed with the photoredox mediator Hantzsch ester alone (see section 6.3.2) and with the mixture of Hantzsch ester and **RhO** in a molar ratio of 2 : 1 (see section 6.3.3), respectively.

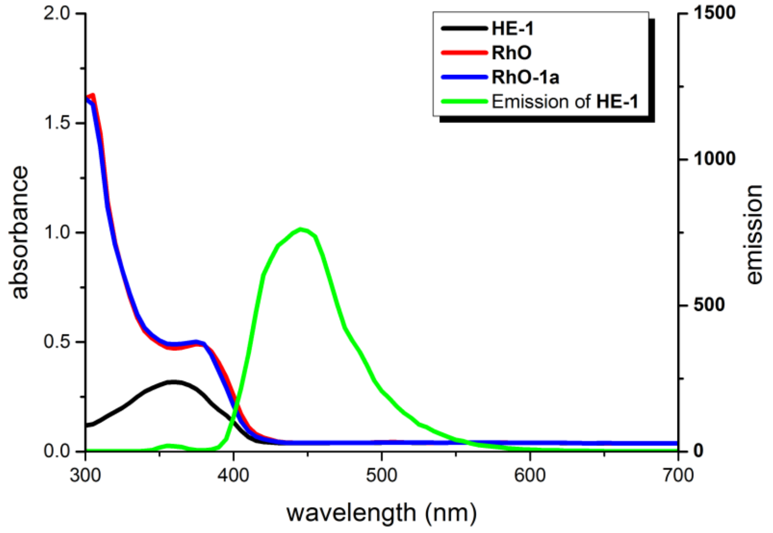

Figure S3. UV-Vis absorption spectra and luminescence emission spectra. Concentration for absorption spectra in 1,4-dioxane: **HE-1** = 0.05 mM, **RhO** = 0.05 mM, **RhO-1a** = 0.05 mM. Concentration for emission spectra of **HE-1** in 1,4-dioxane = 0.5 mM.

6.3.2 Quenching experiments with the Hantzsch ester alone

The solutions of **HE-1** (0.5 mM in 1,4-dioxane) were excited at λ = 360 nm and the emission was measured at 455 nm (emission maximum). For each quenching experiment, after degassed with a nitrogen stream for 5 minutes, the emission intensity of the solution (1 mL) of **HE-1** with different concentration of quencher (0, 0.05, 0.10, 0.15, 0.20, 0.25 mM) in a screw-top 10.0 mm quartz cuvette was collected.

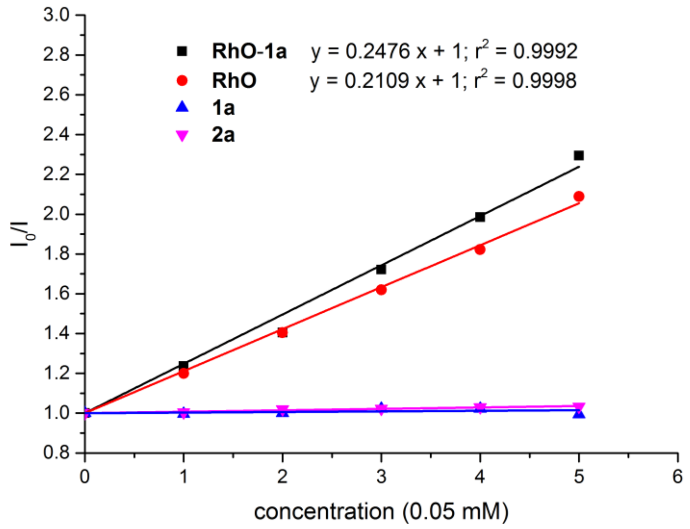

Figure S4. Stern-Volmer plots. I_0 and I are respective luminescence intensities in the absence and presence of the indicated concentrations of the corresponding quencher. [**HE-1**] = 0.5 mM, Ex = 360 nm, Em = 455 nm.

6.3.3 Quenching experiments with Hantzsch ester and RhO.

The solutions of **HE-1** (0.5 mM) and **RhO** (0.25 mM) in 1,4-dioxane were excited at λ = 360 nm and the emission was measured at 455 nm (emission maximum). For each quenching experiment, after degassed with a nitrogen stream for 5 minutes, the emission intensity of the solution (1 mL) of **HE-1** and **RhO** with different concentration of quencher (0, 0.05, 0.10, 0.15, 0.20, 0.25 mM) in a screw-top 10.0 mm quartz cuvette was collected. A very rapid coordination of **1a** with **RhO** is expected based on our previous work.[9]

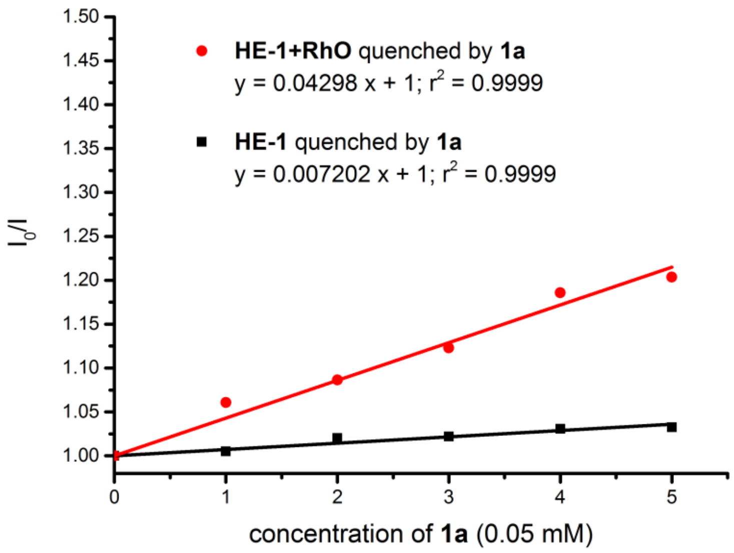

Figure S5. Stern-Volmer plots. I_0 and I are respective luminescence intensities of the solution of **HE-1** (black one) or the mixture of **HE-1** and **RhO** (red one) in the absence and presence of the indicated concentrations of the quencher **1a**. Ex = 360 nm, Em = 455 nm.

Results: **RhO-1a** and **RhO** can quench the luminescence while substrate **1a** and substrate **2a** are not capable of quenching (Figure S4). **RhO** might quench the luminescence of **HE-1** via competitive absorption (inner filter effect).[10] Considering the similar absorption of **RhO-1a** and **RhO** (Figure S3), the in situ generated **RhO-1a** can quench the luminescence of the mixture of **HE-1** and **RhO** slightly (Figure S5), indicating **RhO-1a** might undergoes a photoinduced electron transfer with **HE-1**. Furthermore, **RhO-1a** as the major existing species of rhodium complexes, is most likely responsible for oxidative quenching of photoexcited **HE-1**, which is further supported by cyclic voltammetry studies (see Section 6.4).

6.4 Cyclic Voltammetry

All cyclic voltammetry experiments were carried out using analytical grade CH_2Cl_2 as the solvent containing 0.1 M Bu_4NPF_6 as the electrolyte and 1 mM of the analyte. Cyclic voltammetry experiments were conducted with a computer controlled Eco Chemie Autolab PGSTAT302N potentiostat in a Metrohm electrochemical cell containing a 1 mm diameter planar glassy carbon (GC) disk electrode (eDAQ), a platinum wire auxiliary electrode (Metrohm) and a silver wire miniature reference electrode (eDAQ) that was connected to the test solution via a salt bridge containing 0.5 M *n*Bu_4NPF_6 in CH_3CN. Accurate potentials were referenced to the ferrocene/ferrocenium (Fc/Fc^+) redox couple, which was used as an internal standard. All solutions used for the voltammetric experiments were deoxygenated by purging with high purity argon gas and measurements were performed in a Faraday cage at room temperature (22 ± 2 °C).

Substrate **1a** showed one chemically irreversible reduction process with a cathodic peak potential (E_p^{red}) at –2.59 V *vs.* Fc/Fc^+ (Figure S6, red curve). **RhO-1a** could be reduced with an E_p^{red} at approximately –1.62 V *vs.* Fc/Fc^+ and oxidised with an E_p^{ox} at approximately +1.32 V *vs.* Fc/Fc^+, both in chemically irreversible processes (Figure S6, blue curve). It is noteworthy that coordination of the cyclometalated rhodium catalyst could significantly decrease reductive potential of **1a**.

Besides, **HE-1** could be oxidised in a chemically irreversible process with an anodic peak potential (E_p^{ox}) at approximately 0.50 V *vs.* Fc/Fc^+ (Figure S7). According to luminescence emission spectra (Figure S3, maximum wavelength = 455 nm, corresponding to 2.73 eV), the redox potential of photoexcited **HE-1** is estimated as –2.23 V *vs.* Fc/Fc^+, which is feasible to selectively reduce **RhO-1a** instead of free **1a**.

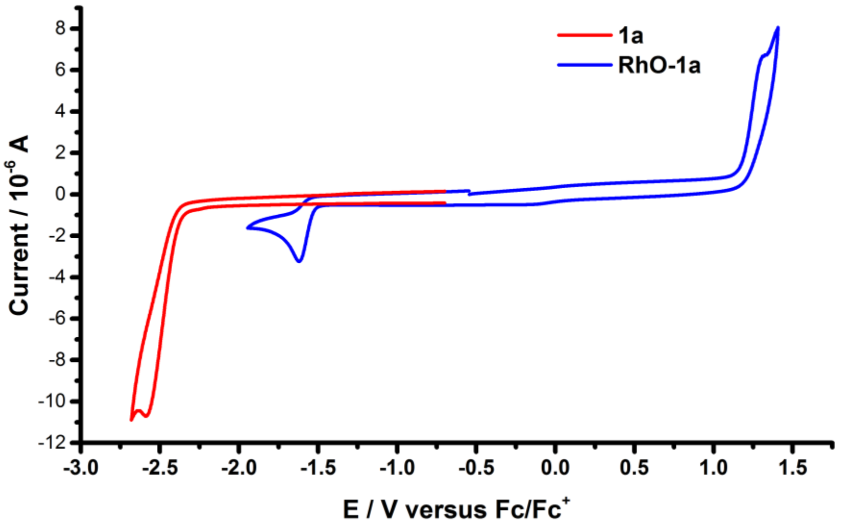

Figure S6. CV of compound **1a** and **RhO-1a**.

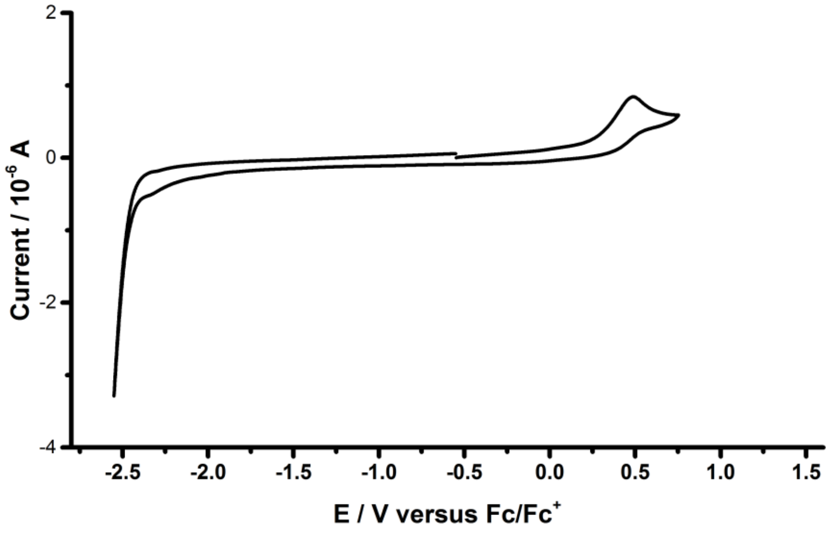

Figure S7. CV of compound **HE-1**.

6.5 Evidence for the Formation of Sulfonyl Radical

6.5.1 Trapping experiments with but-3-en-2-one

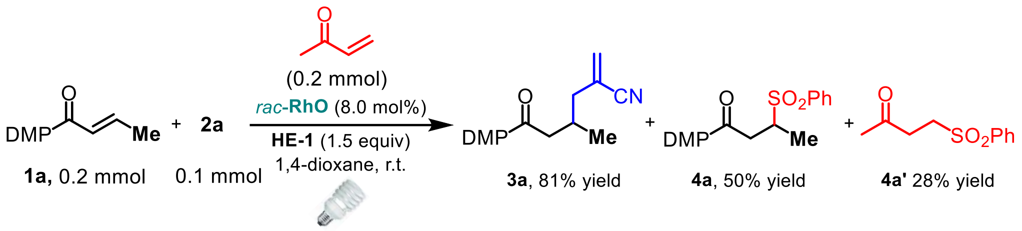

To trap the sulfonyl radical, but-3-en-2-one which is not able to bind the Rh catalyst was added to act as a sulphonyl trap. As shown, the expected radical trapping product **4a'** could be obtained in

28% yield, along with the formation of **3a** and **4a**, indicating the involvement of sulfonyl radical.

4-(Phenylsulfonyl)butan-2-one (4a')[11]

^{1}H NMR (300 MHz, $CDCl_3$) δ 7.95-7.87 (m, 2H), 7.71-7.53 (m, 3H), 3.41-3.33 (m, 2H), 2.96-2.88 (m, 2H), 2.17 (s, 3H).

^{13}C NMR (75 MHz, $CDCl_3$) δ 139.0, 133.9, 129.4, 127.9, 50.5, 35.8, 29.8.

All characteristic data are consistent with the literature report.[11]

6.5.2 EPR experiments

EPR spectra were recorded at room temperature using DMPO (5,5-dimethyl-1-pyrroline *N*-oxide) as free radical spin trapping agent. According to general procedure, the reaction of **1a** and **2a** under standard conditions with the addition of 10 μL DMPO solution (1M in H_2O) was stirred with 21 W CFL for 30 min. Then, a portion of the reaction mixture was taken out to an EPR tube and measured by EPR (9.18142 GHz; Mod. Frequency = 100 kHz; Mod. Ampl. = 0.08 mT).

As shown in Figure S8, two sets of signals were observed, one of which is simulated as signals 1 with 6 lines (g = 2.006; α_N = 9.5 G, $\alpha_H{}^{\beta}$ = 12.9 G) and further signed as EPR signals of sulfonyl radical adducts.[12] These results suggest more than one radical species including sulfonyl radicals are involved in this transformation.

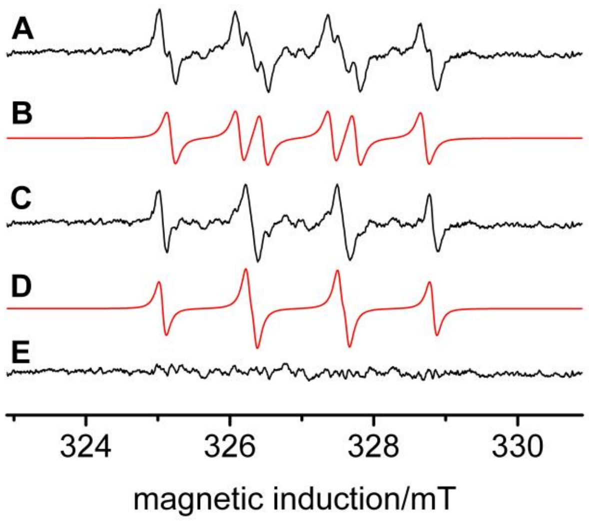

Figure S8. EPR experiments with the addition of DMPO (X band, r.t.). **A**: experimental signals; **B**: simulated signal 1 (g = 2.006, α_N = 9.5 G, $\alpha_H{}^{\beta}$ = 12.9 G); **C**: residual of signals (A-B); **D**: simulated signals 2 (g = 2.006, α_N = 1.200 mT, $\alpha_H{}^{\beta}$ = 1.280 mT); **E**: residual of signals (C-D).

6.6 Determination of the Quantum Yield

The quantum yield of the title reaction **1a**+**2a**→**3a**+**4a** was determined by a method and setups developed by Prof. Dr. Eberhard Riedle's Group.[13] As light source 420 nm LEDs were employed. A powermeter was used as detector. The measurement was accomplished in a dark room with a 1.1 W red LEDs.

Step 1: The radiant power of light transmitted by the cuvette with a blank solution was measured as P_{blank} = 46.25 mW.

Step 2: The reaction mixture of **1a** (65.6 mg, 0.40 mmol), **2a** (41.6 mg, 0.20 mmol), *rac*-**RhO** (13.2 mg, 8.0 mol%), **HE-1** (84.4 mg, 1.5 equiv) in 1,4-dioxane (2.0 mL, 0.1 M) was filled into a fluorescence cuvette with a stirring bar and septum and degassed by bubbling with nitrogen (10 min). Then, the cuvette was put into the setups and illuminated with the 420 nm LEDs. The transmitted radiant power P_{sample} = 3.6 mW was noted. The transmitted radiant power was monitored during the irradiation and remained constant.

Step 3: After illumination for 2 hours (t = 2 × 3600 s), the amount of the formed **3a** was determined as 9.257×10^{-5} mol ($n_{product}$) by ^{1}H NMR.

Step 4: The overall quantum yield can be calculated as following:

$$\text{Quantum Yield} = \frac{N_{product}}{N_{photon}} = \frac{N_A \times n_{product}}{\frac{P_{absorbed} \times \mathrm{t}}{\frac{h \times c}{\lambda}}} = \frac{h \times \mathrm{c} \times N_A \times n_{product}}{(P_{blank} - P_{sample}) \times \mathrm{t} \times \lambda}$$

$$= \frac{6.626 \times 10^{-34}\mathrm{Js} \times 2.998 \times 10^{8}\mathrm{ms}^{-1} \times 6.022 \times 10^{23}\mathrm{mol}^{-1} \times 9.257 \times 10^{-5}\mathrm{mol}}{(46.25 - 3.6) \times 10^{-3}\mathrm{Js}^{-1} \times 2 \times 3600\mathrm{s} \times 420 \times 10^{-9}\mathrm{m}} = 0.09$$

where $N_{product}$ is the number of product **3a** formed; N_{photon} is the number of photons absorbed; N_A is Avogadro's constant; $n_{product}$ is the molar amount of product **3a** formed; $P_{absorbed}$ is the radiant power absorbed; t is the irradiation time; h is the Planck's constant; c is the speed of light; λ is the wavelength of light source, P_{blank} is the radiant power transmitted by the cuvette with a blank solution; P_{sample} is the radiant power transmitted by the cuvette with reaction mixture.

7. Experimental and Analytical Data of Products

(01) Table 2, entry 1

According to the general procedure, the reaction of (*E*)-1-(3,5-dimethyl-1*H*-pyrazol-1-yl) but-2-en-1-one **1a** (32.8 mg, 0.20 mmol), 2-((phenylsulfonyl)methyl)acrylonitrile **2a** (20.8 mg, 0.10 mmol), Δ-**RhO** (6.6 mg, 8.0 mol%), diethyl 2,6-diethyl-1,4-dihydropyridine-3,5-dicarboxylate (42.2 mg, 1.5 equiv) in 1,4-dioxane (1.0 mL, 0.1 M) under nitrogen atmosphere with visible light for 24 hours at room temperature, afforded 19.6 mg (85%) of **3a** as a colorless oil and **4a** (28.2mg, 92%) as a yellow oil.

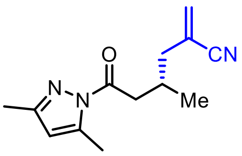

(*S*)-6-(3,5-Dimethyl-1*H*-pyrazol-1-yl)-4-methyl-2-methylene-6-oxon-hexanenitrile (3a)

Enantiomeric excess of **3a** was established by HPLC analysis using a Daicel Chiralpak OD-H column, ee = 96% (HPLC: 254 nm, n-hexane/isopropanol = 99:1, flow rate 1.0 mL/min, 40 °C, t_r (major) = 6.8 min, t_r (minor) = 6.4 min).

$[\alpha]_D^{22}$ = +21.1° (*c* 1.0, CH_2Cl_2).

^{1}H NMR (300 MHz, $CDCl_3$) δ 5.95 (s, 1H), 5.91 (s, 1H), 5.78-5.75 (m, 1H), 3.13 (dd, *J* = 16.5, 5.8 Hz, 1H), 2.99 (dd, *J* = 16.5, 7.3 Hz, 1H), 2.54 (s, 3H), 2.52-2.38 (m, 2H), 2.23 (s, 3H), 2.23-2.09 (m, 1H), 1.07 (d, *J* = 6.6 Hz, 3H).

^{13}C NMR (75 MHz, $CDCl_3$) δ 172.4, 152.0, 144.0, 132.0, 121.7, 118.6, 111.2, 41.5, 41.2, 28.5, 19.3, 14.5, 13.8.

IR (film): ν (cm^{-1}) 2965, 2929, 2221, 1722, 1583, 1437, 1379, 1333, 1240, 1171, 1137, 996, 960, 904, 805, 745, 642, 559.

HRMS (ESI, *m/z*) calcd for $C_{13}H_{17}N_3ONa$ $[M+Na]^+$: 254.1264, found: 254.1259.

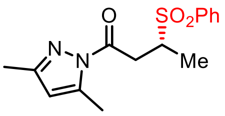

(*R*)-1-(3,5-Dimethyl-1*H*-pyrazol-1-yl)-3-(phenylsulfonyl)butan-1-one (4a)

Enantiomeric excess of **4a** was established by HPLC analysis using a Daicel Chiralpak OD-H column, ee = 85% (HPLC: 254 nm, n-hexane/isopropanol = 95:5, flow rate 1.0 mL/min, 40 °C, t_r

(major) = 12.0 min, t_r (minor) = 10.8 min).

$[\alpha]_D^{22}$ = +47.6° (*c* 1.0, CH_2Cl_2).

^{1}H NMR (500 MHz, $CDCl_3$) δ 7.95-7.91 (m, 2H), 7.68-7.63 (m, 1H), 7.59-7.53 (m, 2H), 5.94 (s, 1H), 3.89-3.82 (m, 1H), 3.76 (dd, *J* = 17.4, 4.4 Hz, 1H), 3.27 (dd, *J* = 17.4, 8.8 Hz, 1H), 2.45 (s, 3H), 2.21 (s, 3H), 1.38 (d, *J* = 6.8 Hz, 3H).

^{13}C NMR (125 MHz, $CDCl_3$) δ 170.0, 152.6, 144.1, 136.9, 133.8, 129.14, 129.12, 111.5, 56.1, 35.8, 14.3, 14.0, 13.8.

IR (film): *v* (cm^{-1}) 2929, 1721, 1585, 1446, 1382, 1304, 1141, 1081, 1030, 986, 961, 911, 814, 757, 728, 688, 582, 547.

HRMS (ESI, *m*/*z*) calcd for $C_{15}H_{19}N_2O_3S$ $[M+H]^+$: 307.1111, found: 307.1107.

(02) Table 2, entry 2

According to the general procedure, the reaction of (*E*)-1-(3,5-dimethyl-1*H*-pyrazol-1-yl) but-2-en-1-one **1a** (32.8 mg, 0.20 mmol), 2-(tosylmethyl)acrylonitrile **2b** (22.1 mg, 0.10 mmol), Δ-**RhO** (6.6 mg, 8.0 mol%), diethyl 2,6-diethyl-1,4-dihydropyridine-3,5-dicarboxylate (42.2 mg, 1.5 equiv) in 1,4-dioxane (1.0 mL, 0.1 M) under nitrogen atmosphere with visible light for 24 hours at room temperature, afforded 16.1 mg (68%) of **3a** as a colorless oil and **4b** (21.4mg, 70%) as a yellow oil. Enantiomeric excess of **3a** was determined as 96% ee.

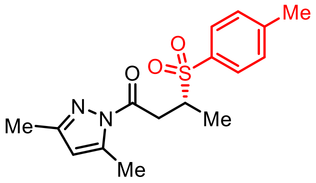

(*R*)-1-(3,5-Dimethyl-1*H*-pyrazol-1-yl)-3-tosylbutan-1-one (4b)

Enantiomeric excess of **4b** was established by HPLC analysis using a Daicel Chiralpak OD-H column, ee = 79% (HPLC: 254 nm, n-hexane/ isopropanol = 95:5, flow rate 1.0 mL/min, 40 °C, t_r (major) = 14.8 min, t_r (minor) = 11.0 min). $[\alpha]_D^{22}$ = +10.8° (*c* 1.0, CH_2Cl_2).

^{1}H NMR (500 MHz, $CDCl_3$) δ 7.82-7.78 (m, 2H), 7.37-7.33 (m, 2H), 5.94 (s, 1H), 3.86-3.78 (m, 1H), 3.74 (dd, *J* = 17.4, 4.4 Hz, 1H), 3.24 (dd, *J* = 17.4, 8.8 Hz, 1H), 2.45 (s, 3H), 2.44 (s, 3H), 2.21 (s, 3H), 1.37 (d, *J* = 6.8 Hz, 3H).

^{13}C NMR (125 MHz, $CDCl_3$) δ 170.1, 152.5, 144.8, 144.1, 133.9, 129.8, 129.1, 111.5, 56.1, 35.9, 21.6, 14.3, 14.0, 13.8.

IR (film): ν (cm^{-1}) 2929, 1721, 1589, 1396, 1330, 1293,1261, 1139, 1082, 1026, 987, 962, 908, 870, 758, 721, 640, 555.

HRMS (ESI, *m*/*z*) calcd for $C_{16}H_{20}N_2O_3SNa$ $[M+Na]^+$: 343.1087, found: 343.1080.

(03) Table 2, entry 3

According to the general procedure, the reaction of (*E*)-1-(3,5-dimethyl-1*H*-pyrazol-1-yl) but-2-en-1-one **1a** (32.8 mg, 0.20 mmol), 2-(((4-bromophenyl)sulfonyl)methyl)acrylonitrile **2c** (28.5 mg, 0.10 mmol), Δ-**RhO** (6.6 mg, 8.0 mol%), diethyl 2,6-diethyl-1,4-dihydropyridine-3,5-dicarboxylate (42.2 mg, 1.5 equiv) in 1,4-dioxane (1.0 mL, 0.1 M) under nitrogen atmosphere with visible light for 24 hours at room temperature, afforded 18.9 mg (81%) of **3a** as a colorless oil, and afforded **4c** (33.8 mg, 88%) as a white solid. Enantiomeric excess of **3a** was determined as 97% ee.

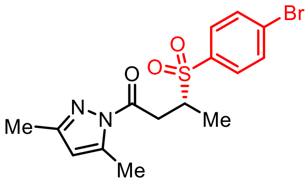

(*R*)-3-((4-bromophenyl)sulfonyl)-1-(3,5-dimethyl-1*H*-pyrazol-1-yl)butan-1-one (4c)

Enantiomeric excess of **4c** was established by HPLC analysis using a Daicel Chiralpak OD-H column, ee = 80% (HPLC: 254 nm, n-hexane/isopropanol = 95:5, flow rate 1.0 mL/min, 40 °C, t_r (major) = 12.9 min, t_r (minor) = 11.2 min). $[\alpha]_D^{22}$ = +13.1° (*c* 1.0, CH_2Cl_2).

^1H NMR (500 MHz, $CDCl_3$) δ 7.80-7.76 (m, 2H), 7.71-7.67 (m, 2H), 5.96 (s, 1H), 3.89-3.81 (m, 1H), 3.76 (dd, *J* = 17.4, 4.8 Hz, 1H), 3.24 (dd, *J* = 17.4, 8.4 Hz, 1H), 2.45 (s, 3H), 2.21 (s, 3H), 1.40 (d, *J* = 6.8 Hz, 3H).

^{13}C NMR (125 MHz, $CDCl_3$) δ 169.7, 152.7, 144.1, 135.9, 132.5, 130.7, 129.3, 111.6, 56.2, 35.8, 14.3, 13.9, 13.8.

IR (film): ν (cm^{-1}) 2981, 1727, 1575, 1446, 1384, 112, 1279, 1134, 1075, 988, 961, 914, 824, 761, 684, 576.

HRMS (ESI, *m*/*z*) calcd for $C_{15}H_{17}BrN_2O_3SNa$ $[M+Na]^+$: 407.0035, found: 407.0024.

(04) Table 2, entry 4

According to the general procedure, the reaction of (*E*)-1-(3,5-dimethyl-1*H*-pyrazol-1-yl)but-2-

en-1-one **1a** (32.8 mg, 0.20 mmol), 2-(((4-(trifluoromethyl)phenyl)sulfonyl)methyl) acrylonitrile **2d** (22.1 mg, 0.10 mmol), Δ-**RhO** (6.6 mg, 8.0 mol%), diethyl 2,6-diethyl-1,4-dihydropyridine-3,5-dicarboxylate (42.2 mg, 1.5 equiv) in 1,4-dioxane (1.0 mL, 0.1 M) under nitrogen atmosphere with visible light for 24 hours at room temperature, afforded 18.4 mg (78%) of **3a** as a colorless oil, and afforded 29.2 mg (78%) of **4d** as a white solid. Enantiomeric excess of **3a** was determined as 95% ee.

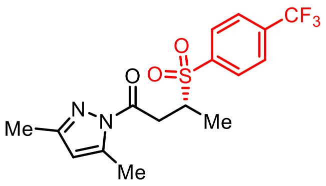

(*R*)-1-(3,5-Dimethyl-1*H*-pyrazol-1-yl)-3-((4-(trifluoromethyl)phenyl)sulfonyl)butan-1-one (4d)

Enantiomeric excess of **4d** was established by HPLC analysis using a Daicel Chiralpak OD-H column, ee = 76% (HPLC: 254 nm, n-hexane/isopropanol = 95:5, flow rate 1.0 mL/min, 40 °C, t_r (major) = 10.3 min, t_r (minor) = 9.2 min). A white solid. $[\alpha]_D^{22}$ = +19.9° (*c* 1.0, CH_2Cl_2).

^{1}H NMR (500 MHz, $CDCl_3$) δ 8.07 (d, *J* = 8.2 Hz, 2H), 7.82 (d, *J* = 8.2 Hz, 2H), 5.95 (s, 1H), 3.94-3.86 (m, 1H), 3.78 (dd, *J* = 17.4, 4.9 Hz, 1H), 3.24 (dd, *J* = 17.4, 8.3 Hz, 1H), 2.43 (s, 3H), 2.20 (s, 3H), 1.42 (d, *J* = 6.9 Hz, 3H).

^{13}C NMR (125 MHz, $CDCl_3$) δ 169.6, 152.7, 144.2, 140.6, 135.5 (q, *J* = 33.2 Hz), 129.8, 126.2 (q, *J* = 3.6 Hz), 124.2, 123.0 (q, *J* = 273.2 Hz), 111.7, 56.2, 35.8, 14.2, 13.9, 13.8.

^{19}F NMR (282 MHz, $CDCl_3$) δ –63.3 (s, 3F).

IR (film): *v* (cm^{-1}) 2965, 1725, 1584, 1451, 1378, 1317, 1166, 1060, 1036, 984, 960, 890, 844, 743, 677, 545.

HRMS (ESI, *m*/*z*) calcd for $C_{16}H_{17}F_3N_2O_3SNa$ $[M+Na]^+$: 397.0804, found: 397.0794.

(05) Table 2, entry 5

According to the general procedure, the reaction of (*E*)-1-(3,5-dimethyl-1*H*-pyrazol-1-yl) but-2-en-1-one **1a** (32.8 mg, 0.20 mmol), 2-((*o*-tolylsulfonyl)methyl)acrylonitrile **2e** (22.1 mg, 0.10 mmol), Δ-**RhO** (6.6 mg, 8.0 mol%), diethyl 2,6-diethyl-1,4-dihydropyridine-3,5-dicarboxylate (42.2 mg, 1.5 equiv) in 1,4-dioxane (1.0 mL, 0.1 M) under nitrogen atmosphere with visible light for 24 hours at room temperature, afforded 16.8 mg (71%) of **3a** as a colorless oil, and afforded 23.0 mg (72%) of **4e** as a yellow oil. Enantiomeric excess of **3a** was determined as 95% ee.

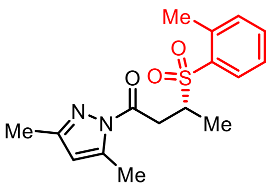

(R)-1-(3,5-Mimethyl-1H-pyrazol-1-yl)-3-(o-tolylsulfonyl)butan-1-one (4e)

Enantiomeric excess of **4e** was established by HPLC analysis using a Daicel Chiralpak OD-H column, ee = 86% (HPLC: 254 nm, n-hexane/isopropanol = 95:5, flow rate 1.0 mL/min, 40 °C, t_r (major) = 12.2 min, t_r (minor) = 10.8 min). $[\alpha]_D^{22}$ = +15.4° (c 1.0, CH_2Cl_2).

^{1}H NMR (500 MHz, $CDCl_3$) δ 7.99 (d, J = 7.9 Hz, 1H), 7.51-7.47 (m, 1H), 7.35-7.29 (m, 2H), 5.93 (s, 1H), 3.99-3.91 (m, 1H), 3.75 (dd, J = 17.4, 4.4 Hz, 1H), 3.27 (dd, J = 17.4, 8.6 Hz, 1H), 2.73 (s, 3H), 2.43 (s, 3H), 2.20 (s, 3H), 1.40 (d, J = 6.8 Hz, 3H).

^{13}C NMR (125 MHz, $CDCl_3$) δ 170.1, 152.5, 144.0, 138.9, 135.2, 133.7, 132.8, 131.3, 126.3, 111.5, 54.8, 35.7, 20.5, 14.3, 13.7, 13.4.

IR (film): ν (cm^{-1}) 2981, 1722, 1586, 1452, 1382, 1308, 1144, 1031, 986, 961, 807, 758, 662, 554.

HRMS (ESI, m/z) calcd for $C_{16}H_{20}N_2O_3SNa$ $[M+Na]^+$: 343.1087, found: 343.1080.

(06) Table 2, entry 6

According to the general procedure, the reaction of (E)-1-(3,5-dimethyl-1H-pyrazol-1-yl) but-2-en-1-one **1a** (32.8 mg, 0.20 mmol), 2-((mesitylsulfonyl)methyl)acrylonitrile **2f** (24.9 mg, 0.10 mmol), Δ-**RhO** (6.6 mg, 8.0 mol%), diethyl 2,6-diethyl-1,4-dihydropyridine-3,5-dicarboxylate (42.2 mg, 1.5 equiv) in 1,4-dioxane (1.0 mL, 0.1 M) under nitrogen atmosphere with visible light for 24 hours at 35 °C, afforded 13.5 mg (57%) of **3a** as a colorless oil, and afforded 20.9 mg (60%) of **4f** as a yellow oil. Enantiomeric excess of **3a** was determined as 94% ee.

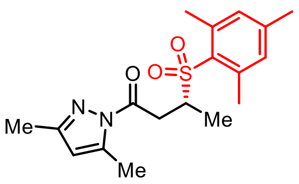

(R)-1-(3,5-Dimethyl-1H-pyrazol-1-yl)-3-(mesitylsulfonyl)butan-1-one (4f)

Enantiomeric excess of **4f** was established by HPLC analysis using a Daicel Chiralpak IA column, ee = 89% (HPLC: 254 nm, n-hexane/isopropanol = 95:5, flow rate 1.0 mL/min, 40 °C, t_r (major) = 9.6 min, t_r (minor) = 10.9 min). $[\alpha]_D^{22}$ = –28.4° (c 0.10, CH_2Cl_2).

^{1}H NMR (500 MHz, $CDCl_3$) δ 6.93 (s, 2H), 5.93 (s, 1H), 3.97-3.89 (m, 1H), 3.78 (dd, J = 17.0, 4.6

Hz, 1H), 3.27 (dd, J = 17.0, 8.6 Hz, 1H), 2.68 (s, 6H), 2.43 (s, 3H), 2.28 (s, 3H), 2.19 (s, 3H), 1.41 (d, J = 6.9 Hz, 3H).

^{13}C NMR (125 MHz, $CDCl_3$) δ 170.3, 152.4, 144.0, 143.3, 140.8, 132.3, 131.1, 111.4, 55.4, 35.4, 23.0, 21.0, 14.3, 13.7, 13.2.

IR (film): ν (cm^{-1}) 2959, 1718, 1591, 1449, 1416, 1378, 1306, 1262, 1134, 1101, 1023, 962, 799, 749, 728, 681, 642, 569.

HRMS (ESI, m/z) calcd for $C_{18}H_{25}N_2O_3S$ [M+H]$^+$: 349.1580, found: 349.1573.

(07) Table 2, entry 7

According to the general procedure, the reaction of (*E*)-1-(3,5-dimethyl-1*H*-pyrazol-1-yl) but-2-en-1-one **1a** (32.8 mg, 0.20 mmol), ethyl 2-((naphthalen-2-ylsulfonyl)methyl)acrylonitrile **2g** (25.7 mg, 0.10 mmol), Δ-**RhO** (6.6 mg, 8.0 mol%), diethyl 2,6-diethyl-1,4-dihydropyridine-3,5-dicarboxylate (42.2 mg, 1.5 equiv) in 1,4-dioxane (1.0 mL, 0.1 M) under nitrogen atmosphere with visible light for 24 hours at 35 °C, afforded 19.4 mg (82%) of **3a** as a colorless oil, and afforded 31.3 mg (88%) of **4g** as a yellow oil. Enantiomeric excess of **3a** was determined as 94% ee.

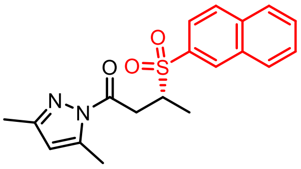

(*R*)-1-(3,5-Dimethyl-1*H*-pyrazol-1-yl)-3-(naphthalen-2-ylsulfonyl)butan-1-one (4g)

Enantiomeric excess of **4g** was established by HPLC analysis using a Daicel Chiralpak OD-H column, ee = 83% (HPLC: 254 nm, n-hexane/isopropanol = 95:5, flow rate 1.0 mL/min, 40 °C, t_r (major) = 13.5 min, t_r (minor) = 14.3 min). $[\alpha]_D^{22}$ = +39.2° (c 1.0, CH_2Cl_2)

^{1}H NMR (300 MHz, $CDCl_3$) δ 8.80 (d, J = 8.6 Hz, 1H), 8.31 (d, J = 7.3 Hz, 1H), 8.11 (d, J = 8.2 Hz, 1H), 7.95 (d, J = 8.2 Hz, 1H), 7.75-7.53 (m, 3H), 5.89 (s, 1H), 4.22-4.08 (m, 1H), 3.82 (dd, J = 17.2, 4.4 Hz, 1H), 3.34 (dd, J = 17.2, 8.8 Hz, 1H), 2.39 (s, 3H), 2.14 (s, 3H), 1.39 (d, J = 6.8 Hz, 3H).

^{13}C NMR (75 MHz, $CDCl_3$) δ 170.0, 152.4, 144.0, 135.3, 134.3, 132.2, 131.8, 129.3, 129.1, 128.7, 127.0, 124.5, 124.2, 111.4, 55.6, 35.8, 14.2, 13.8, 13.6.

IR (film): ν (cm^{-1}) 2937, 1722, 1586, 1385, 1313, 1269, 1188, 1151, 1122, 964, 886, 805, 771, 742, 707, 663, 592.

HRMS (ESI, m/z) calcd for $C_{19}H_{20}N_2O_3SNa$ [M+Na]$^+$: 379.1087, found: 379.1078.

(08) Table 2, entry 8

According to the general procedure, the reaction of (*E*)-1-(3,5-dimethyl-1*H*-pyrazol-1-yl) but-2-en-1-one **1a** (32.8 mg, 0.20 mmol), ethyl 1-((naphthalen-2-ylsulfonyl)methyl)acrylonitrile **2h** (25.7 mg, 0.10 mmol), Δ-**RhO** (6.6 mg, 8.0 mol%), diethyl 2,6-diethyl-1,4-dihydropyridine-3,5-dicarboxylate (42.2 mg, 1.5 equiv) in 1,4-dioxane (1.0 mL, 0.1 M) under nitrogen atmosphere with visible light for 24 hours at 35 °C, afforded 18.4 mg (78%) of **3a** as a colorless oil, and afforded 29.9 mg (84%) of **4h** as a yellow oil. Enantiomeric excess of **3a** was determined as 91% ee.

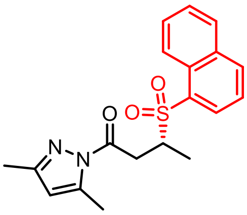

(*R*)-1-(3,5-Dimethyl-1*H*-pyrazol-1-yl)-3-(naphthalen-1-ylsulfonyl)butan-1-one (4h)

Enantiomeric excess of **4h** was established by HPLC analysis using a Daicel Chiralpak OD-H column, ee = 80% (HPLC: 254 nm, n-hexane/isopropanol = 95:5, flow rate 1.0 mL/min, 40 °C, t_r (major) = 18.7 min, t_r (minor) = 16.5 min. $[\alpha]_D^{22}$ = +15.6° (*c* 1.0, CH_2Cl_2).

^{1}H NMR (300 MHz, $CDCl_3$) δ 8.50 (s, 1H), 8.03-7.87 (m, 4H), 7.71-7.57 (m, 2H), 5.88 (s, 1H), 4.02-3.88 (m, 1H), 3.84 (dd, *J* = 17.4, 4.6 Hz, 1H), 3.27 (dd, *J* = 17.4, 8.4 Hz, 1H), 2.36 (s, 3H), 2.19 (s, 3H), 1.43 (d, *J* = 6.8 Hz, 3H).

^{13}C NMR (75 MHz, $CDCl_3$) δ 170.0, 152.5, 144.0, 135.4, 134.0, 132.1, 131.1, 129.4, 129.34, 129.30, 127.9, 127.6, 123.6, 111.5, 56.2, 36.0, 14.1, 14.0, 13.7.

IR (film): ν (cm^{-1}) 2924, 1721, 1586, 1451, 1382, 1305, 1122, 1073, 1029, 985, 961, 910, 862, 815, 752, 703, 644, 553, 474.

HRMS (ESI, *m*/*z*) calcd for $C_{19}H_{21}N_2O_3S$ $[M+H]^+$: 357.1267, found: 357.1259.

(09) Table 2, entry 9

According to the general procedure, the reaction of (*E*)-1-(3,5-dimethyl-1*H*-pyrazol-1-yl) but-2-en-1-one **1a** (32.8 mg, 0.20 mmol), ethyl ethyl 2-((phenylsulfonyl)methyl)acrylate **2i**[14] (25.4 mg, 0.10 mmol), Δ-**RhO** (6.6 mg, 8.0 mol%), diethyl 2,6-diethyl-1,4-dihydropyridine-3,5-dicarboxylate (42.2 mg, 1.5 equiv) in 1,4-dioxane (1.0 mL, 0.1 M) under nitrogen atmosphere with

visible light for 24 hours at room temperature, afforded 18.3 mg (65%) of **3b** as a colorless oil, and afforded 20.8 mg (68%) of **4a** as a yellow oil. Enantiomeric excess of **4a** was determined as 84% ee.

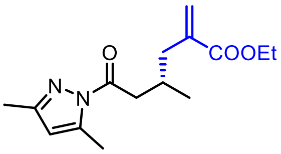

Ethyl (*S*)-6-(3,5-dimethyl-1*H*-pyrazol-1-yl)-4-methyl-2-methylene-6-oxohexanoate (3b)

Enantiomeric excess of **3b** was established by HPLC analysis using a Daicel Chiralcel OJ-H column, ee = 94% (HPLC: 254 nm, n-hexane/isopropanol = 99:1, flow rate 1.0 mL/min, 25 °C, t_r (major) = 6.9 min, t_r (minor) = 7.6 min). $[\alpha]_D^{22}$ = +14.2° (*c* 1.0, CH_2Cl_2).

^{1}H NMR (300 MHz, $CDCl_3$) δ 6.19 (d, *J* = 1.6 Hz, 1H), 5.93 (s, 1H), 5.57-5.53 (m, 1H), 4.19 (q, *J* = 7.2 Hz, 2H), 3.11 (dd, *J* = 16.4, 5.2 Hz, 1H), 2.93 (dd, *J* = 16.4, 7.4 Hz, 1H), 2.53 (s, 3H), 2.47-2.29 (m, 3H), 2.22 (s, 3H), 1.29 (t, *J* = 7.2 Hz, 3H),1.00 (d, *J* = 6.4 Hz, 3H).

^{13}C NMR (75 MHz, $CDCl_3$) δ 173.2, 167.2, 151.6, 144.0, 139.2, 126.2, 110.9, 60.6, 41.7, 39.1, 28.8, 19.8, 14.6, 14.2, 13.8.

IR (film): ν (cm^{-1}) 2967, 1718, 1630, 1583, 1440, 1409, 1377, 1329, 1242, 1185, 1148, 1025, 994, 959, 807, 746, 684, 553.

HRMS (ESI, *m*/*z*) calcd for $C_{15}H_{22}N_2O_3Na$ $[M+Na]^+$: 301.1523, found: 301.1527.

(10) Table 2, entry 10

According to the general procedure, the reaction of (*E*)-1-(3,5-dimethyl-1*H*-pyrazol-1-yl) but-2-en-1-one **1a** (32.8 mg, 0.20 mmol), ethyl 2-(((4-methoxyphenyl)sulfonyl)methyl)acrylate **2j** (28.4 mg, 0.10 mmol), Δ-**RhO** (6.6 mg, 8.0 mol%), diethyl 2,6-diethyl-1,4-dihydropyridine-3,5-dicarboxylate (42.2 mg, 1.5 equiv) in 1,4-dioxane (1.0 mL, 0.1 M) under nitrogen atmosphere with visible light for 24 hours at 35 °C, afforded 18.3 mg (65%) of **3b** as a colorless oil, and afforded 21.2 mg (63%) of **4i** as a yellow oil. Enantiomeric excess of **3b** was determined as 92% ee.

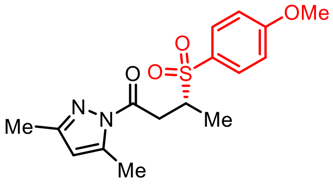

(*R*)-1-(3,5-Dimethyl-1*H*-pyrazol-1-yl)-3-((4-methoxyphenyl)sulfonyl)butan-1-one (4i)

Enantiomeric excess of **4i** was established by HPLC analysis using a Daicel Chiralpak OD-H column, ee = 81% (HPLC: 254 nm, n-hexane/isopropanol = 90:10, flow rate 1.0 mL/min, 40 °C, t_r (major) = 14.4 min, t_r (minor) = 11.9 min). $[\alpha]_D^{22}$ = +10.0° (*c* 1.0, CH_2Cl_2).

^{1}H NMR (500 MHz, $CDCl_3$) δ 7.87-7.81 (m, 2H), 7.03-7.67 (m, 2H), 5.94 (s, 1H), 3.88 (s, 3H), 3.85-3.71 (m, 2H), 3.27 (dd, *J* = 17.4, 8.8 Hz, 1H), 2.46 (s, 3H), 2.21 (s, 3H), 1.37 (d, *J* = 6.8 Hz, 3H).

^{13}C NMR (125 MHz, $CDCl_3$) δ 170.1, 163.8, 152.5, 144.1, 131.3, 128.3, 114.3, 111.5, 56.3, 55.7, 36.0, 14.3, 14.1, 13.8.

IR (film): ν (cm^{-1}) 2922, 1716, 1587, 1457, 1372, 1310, 1264, 1130, 1080, 1020, 960, 842, 411, 758, 562.

HRMS (ESI, *m*/*z*) calcd for $C_{16}H_{20}N_2O_4SNa$ $[M+Na]^+$: 359.1036, found: 359.1027.

(11) Table 2, entry 11

According to the general procedure, the reaction of (*E*)-1-(3,5-dimethyl-1H-pyrazol-1-yl) but-2-en-1-one **1a** (32.8 mg, 0.20 mmol), ethyl hex-4-yn-1-yl 2-((phenylsulfonyl)methyl)acrylate **2k** (30.6 mg, 0.10 mmol), Δ-**RhO** (6.6 mg, 8.0 mol%), diethyl 2,6-diethyl-1,4-dihydropyridine-3,5-dicarboxylate (42.2 mg, 1.5 equiv) in 1,4-dioxane (1.0 mL, 0.1 M) under nitrogen atmosphere with visible light for 24 hours at 35 °C, afforded 19.0 mg (60%) of **3c** as a colorless oil, and afforded 21.1 mg (69%) of **4a** as a yellow oil. Enantiomeric excess of **4a** was determined as 82% ee.

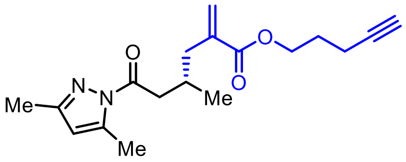

(*S*)-Pent-4-yn-1-yl-6-(3,5-dimethyl-1*H*-pyrazol-1-yl)-4-methyl-2-methylene-6-oxohexanoate (3c)

Enantiomeric excess of **3c** was established by HPLC analysis using a Daicel Chiralcel OJ-H column, ee = 92% (HPLC: 254 nm, n-hexane/isopropanol = 98:2, flow rate 1.0 mL/min, 25 °C, t_r (major) = 10.0 min, t_r (minor) = 11.8 min). $[\alpha]_D^{22}$ = +10.4° (*c* 1.0, CH_2Cl_2).

^{1}H NMR (500 MHz, $CDCl_3$) δ 6.20 (d, *J* = 1.4 Hz, 1H), 5.94 (s, 1H), 5.59-5.56 (m, 1H), 4.24 (t, *J* = 6.3 Hz, 2H), 3.11 (dd, *J* = 16.4, 5.4 Hz, 1H), 2.94 (dd, *J* = 16.4, 7.4 Hz, 1H), 2.53 (s, 3H), 2.47-2.36 (m, 2H), 2.31 (td, *J* = 6.8, 2.6 Hz, 3H), 2.23 (s, 3H), 1.97 (t, *J* = 2.6 Hz, 1H), 1.96-1.87 (m, 2H), 1.00 (d, *J* = 6.4 Hz, 3H).

^{13}C NMR (125 MHz, $CDCl_3$) δ 173.1, 167.0, 151.7, 144.0, 138.9, 126.6, 111.0, 83.1, 69.0, 63.2, 41.6, 39.1, 28.7, 27.5, 19.8, 15.3, 14.6, 13.8.

IR (film): ν (cm^{-1}) 3296, 2960, 2928, 1718, 1629, 1583, 1438, 1409, 1379, 1330, 1244, 1182, 1149, 1077, 996, 959, 809, 726, 637, 523.

HRMS (ESI, *m*/*z*) calcd for $C_{18}H_{24}N_2O_3Na$ $[M+Na]^+$: 339.1679, found: 339.1673.

(12) Table 2, entry 12

According to the general procedure, the reaction of (*E*)-1-(3,5-dimethyl-1*H*-pyrazol-1-yl)but-2-en-1-one **1a** (32.8 mg, 0.20 mmol), (*E*)-3,7-dimethylocta-2,6-dien-1-yl 2-((phenylsulfonyl)methyl) acrylate **2l** (*contains about 5% of Z-isomer derived from geraniol*, 36.2 mg, 0.10 mmol), **Δ-RhO** (6.6 mg, 8.0 mol%), diethyl 2,6-diethyl-1,4- dihydropyridine-3,5-dicarboxylate (42.2 mg, 1.5 equiv) in 1,4-dioxane (1.0 mL, 0.1 M) under nitrogen atmosphere with visible light for 24 hours at 35 °C, afforded 23.9 mg (62%) of **3d** as a colorless oil, and afforded 22.0 mg (72%) of **4a** as a yellow oil. Enantiomeric excess of **4a** was determined as 83% ee.

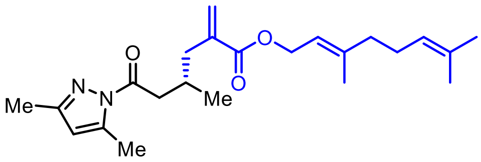

(*E*)-3,7-Dimethylocta-2,6-dien-1-yl (*S*)-6-(3,5-dimethyl-1*H*-pyrazol-1-yl)-4-methyl-2-methylene -6-oxohexanoate (3d)

Enantiomeric excess of **3d** was established by HPLC analysis using a Daicel Chiralpak IG column, ee = 92% (HPLC: 254 nm, n-hexane/isopropanol = 99.1:0.9, flow rate 1.0 mL/min, 25 °C, t_r (major) = 12.4 min, t_r (minor) = 13.3 min). $[\alpha]_D^{22}$ = +15.7° (*c* 1.0, CH_2Cl_2).

^1H NMR (500 MHz, $CDCl_3$) δ 6.20 (d, *J* = 1.4 Hz, 1H), 5.94 (s, 1H), 5.58-5.55 (m, 1H), 5.40-5.34 (m, 1H), 5.11-5.05 (m, 1H), 4.66 (d, 7.0 Hz, 2H), 3.11 (dd, *J* = 16.4, 5.4 Hz, 1H), 2.94 (dd, *J* = 16.4, 7.8 Hz, 1H), 2.53 (s, 3H), 2.47-2.29 (m, 3H), 2.22 (s, 3H), 2.13-2.01 (m, 4H), 1.71 (s, 3H), 1.67 (s, 3H), 1.60 (s, 3H), 1.00 (d, *J* = 6.4 Hz, 3H).

^{13}C NMR (125 MHz, $CDCl_3$) δ 173.2, 167.2, 151.6, 144.0, 142.0, 139.1, 131.8, 126.3, 123.8, 118.4, 111.0, 61.7, 41.6, 39.5, 39.1, 28.7, 26.3, 25.7, 19.9, 17.7, 16.5, 14.6, 13.8.

IR (film): ν (cm^{-1}) 3298, 3172, 2963, 2925, 2873, 1633, 1598, 1519, 1462, 1381, 1316, 1244, 1158, 1079, 1005, 956, 870, 770, 661, 544.

HRMS (ESI, *m*/*z*) calcd for $C_{23}H_{34}N_2O_3Na$ $[M+Na]^+$: 409.2462, found: 409.2454.

(13) Table 2, entry 13

According to the general procedure, the reaction of (*E*)-1-(3,5-dimethyl-1*H*-pyrazol-1-yl) but-2-en-1-one **1a** (32.8 mg, 0.20 mmol), 3-(1,3-dioxoisoindolin-2-yl)propyl 2-((phenylsulfonyl) methyl)acrylate **2m** (41.3 mg, 0.10 mmol), Δ-**RhO** (6.6 mg, 8.0 mol%), diethyl 2,6-diethyl-1,4-dihydropyridine-3,5-dicarboxylate (42.2 mg, 1.5 equiv) in 1,4-dioxane (1.0 mL, 0.1 M) under nitrogen atmosphere with visible light for 24 hours at 35 °C, afforded 31.9 mg (73%) of **3e** as a colorless oil, and afforded 23.9 mg (78%) of **4a** as a yellow oil. Enantiomeric excess of **4a** was determined as 82% ee

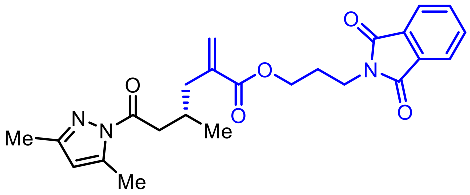

(*S*)-3-(1,3-Dioxoisoindolin-2-yl)propyl 6-(3,5-dimethyl-1*H*-pyrazol-1-yl)-4-methyl-2-methylene -6-oxohexanoate (3e)

Enantiomeric excess of **3e** was established by HPLC analysis using a Daicel Chiralcel OJ-H column, ee = 92% (HPLC: 254 nm, n-hexane/isopropanol = 80:20, flow rate 1.0 mL/min, 25 °C, t_r (major) = 17.4 min, t_r (minor) = 20.5 min). $[\alpha]_D^{22}$ = +13.7° (*c* 1.0, CH_2Cl_2).

^{1}H NMR (500 MHz, $CDCl_3$) *δ* 7.84-7.80 (m, 2H), 7.72-7.68 (m, 2H), 6.17 (s, 1H), 5.92 (s, 1H), 5.55 (s, 1H), 4.17 (t, *J* = 6.2 Hz, 2H), 3.82 (t, *J* = 6.9 Hz, 2H), 3.10 (dd, *J* = 16.4, 5.4 Hz, 1H), 2.92 (dd, *J* = 16.4, 7.7 Hz, 1H), 2.51 (s, 3H), 2.45-2.33 (m, 2H), 2.32-2.26 (m, 1H), 2.21 (s, 3H), 2.11-2.04 (m, 2H), 0.99 (d, *J* = 6.4 Hz, 3H).

^{13}C NMR (125 MHz, $CDCl_3$) *δ* 173.1, 168.2, 166.9, 151.6, 143.9, 138.7, 133.9, 132.0, 126.7, 123.2, 110.9, 62.0, 41.6, 39.0, 35.1, 28.7, 27.6, 19.8, 14.6, 13.8.

IR (film): *v* (cm^{-1}) 2959, 2928, 1772, 1709, 1583, 1439, 1377, 1330, 1244, 1182, 1148, 1080, 1046, 959, 805, 747, 528.

HRMS (ESI, *m/z*) calcd for $C_{24}H_{27}N_3O_5Na$ $[M+Na]^+$: 460.1843, found: 460.1832.

(14) Eq 1, reaction of 1b

According to the general procedure, the reaction of (*E*)-1-(3,5-dimethyl-1*H*-pyrazol-1-yl) pent-2-en-1-one **1b** (35.6 mg, 0.20 mmol), 2-((phenylsulfonyl)methyl)acrylonitrile **2a** (20.8 mg,

0.10 mmol), Δ-**RhO** (6.6 mg, 8.0 mol%), diethyl 2,6-diethyl-1,4-dihydropyridine-3,5-dicarboxylate (42.2 mg, 1.5 equiv) in 1,4-dioxane (1.0 mL, 0.1 M) under nitrogen atmosphere with visible light for 48 hours at room temperature, afforded 20.1 mg (82%) of **3f** as a colorless oil, and afforded 27.2 mg (85%) of **4j** as a yellow oil.

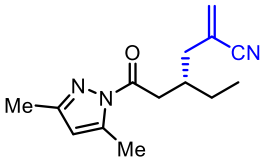

(*S*)-6-(3,5-Dimethyl-1*H*-pyrazol-1-yl)-4-ethyl-2-methylene-6-oxohexanenitrile (3f)

Enantiomeric excess of **3f** was established by HPLC analysis using a Daicel Chiralpak OD-H column, ee = 87% (HPLC: 254 nm, n-hexane/isopropanol = 99:1, flow rate 1.0 mL/min, 40 °C, t_r (major) = 6.8 min, t_r (minor) = 8.5 min). $[\alpha]_D^{22}$ = +4.0° (*c* 1.0, CH_2Cl_2).

^1H NMR (500 MHz, $CDCl_3$) δ 5.96 (s, 1H), 5.90 (s, 1H), 5.77-5.75 (m, 1H), 3.14-3.04 (m, 2H), 2.53 (s, 3H), 2.38-2.31 (m, 3H), 2.23 (s, 3H), 1.55-1.43 (m, 2H), 0.95 (t, *J* = 7.4 Hz, 3H).

^{13}C NMR (125 MHz, $CDCl_3$) δ 172.7, 151.9, 144.0, 132.1, 121.7, 118.7, 111.2, 38.7, 38.1, 34.6, 25.9, 14.6, 13.8, 10.8.

IR (film): *v* (cm^{-1}) 2925, 2221, 1721, 1643, 1583, 1380, 1341, 1271, 1017, 959, 806, 745, 686, 578.

HRMS (ESI, *m*/*z*) calcd for $C_{14}H_{19}N_3ONa$ $[M+Na]^+$: 268.1420, found: 268.1417.

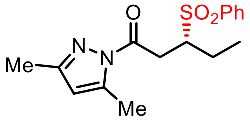

(*R*)-1-(3,5-Dimethyl-1*H*-pyrazol-1-yl)-3-(phenylsulfonyl)pentan-1-one (4j)

Enantiomeric excess of **4j** was established by HPLC analysis using a Daicel Chiralpak OD-H column, ee = 87% (HPLC: 254 nm, n-hexane/isopropanol = 95:5, flow rate 1.0 mL/min, 40 °C, t_r (major) = 12.6 min, t_r (minor) = 11.8 min). $[\alpha]_D^{22}$ = –4.2° (*c* 1.0, CH_2Cl_2).

^1H NMR (500 MHz, $CDCl_3$) δ 7.94-7.90 (m, 2H), 7.65-7.61 (m, 1H), 7.56-7.50 (m, 2H), 5.94 (s, 1H), 3.85-3.79 (m, 1H), 3.77 (dd, *J* = 17.6, 6.3 Hz, 1H), 3.28 (dd, *J* = 17.6, 5.8 Hz, 1H), 2.43 (s, 3H), 2.22 (s, 3H), 2.11-2.01 (m, 1H), 1.72-1.62 (m, 1H), 1.02 (t, *J* = 7.4 Hz, 3H).

^{13}C NMR (125 MHz, $CDCl_3$) δ 170.2, 152.4, 144.2, 137.6, 133.7, 129.1, 129.0, 111.5, 61.6, 33.7, 21.8, 14.3, 13.8, 11.2.

IR (film): *v* (cm^{-1}) 2969, 2931, 1720, 1586, 1447, 1409, 1379, 1298, 1262, 1141, 1081, 959, 629, 693, 595, 563.

HRMS (ESI, *m*/*z*) calcd for $C_{16}H_{20}N_2O_3SNa$ $[M+Na]^+$: 343.1087, found: 343.1079.

(15) Eq 1, reaction of 1c

According to the general procedure, the reaction of (*E*)-1-(3,5-dimethyl-1*H*-pyrazol-1-yl) hex-2-en-1-one **1c** (38.5 mg, 0.20 mmol), 2-((phenylsulfonyl)methyl)acrylonitrile **2a** (20.8 mg, 0.10 mmol), Δ-**RhO** (6.6 mg, 8.0 mol%), diethyl 2,6-diethyl-1,4-dihydropyridine-3,5-dicarboxylate (42.2 mg, 1.5 equiv) in 1,4-dioxane (1.0 mL, 0.1 M) under nitrogen atmosphere with visible light for 40 hours at 35 °C, afforded 20.3 mg (78%) of **3g** as a colorless oil, and afforded 26.7 mg (80%) of **4k** as a yellow oil.

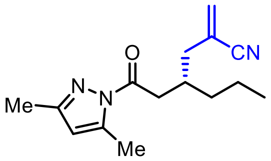

(*S*)-6-(3,5-Dimethyl-1*H*-pyrazol-1-yl)-2-oxoethyl)-2-methyleneheptanenitrile (3g)

Enantiomeric excess of **3g** was established by HPLC analysis using a Daicel Chiralpak OD-H column, ee = 77% (HPLC: OD-H, 254 nm, n-hexane/isopropanol = 99:1, flow rate 1.0 mL/min, 40 °C, t_r (major) = 6.2 min, t_r (minor) = 5.5 min). $[\alpha]_D^{22}$ = +11.8° (*c* 1.0, CH_2Cl_2).

^{1}H NMR (300 MHz, $CDCl_3$) δ 5.95 (s, 1H), 5.89 (s, 1H), 5.74 (s, 1H), 3.16-3.00 (m, 2H), 2.53 (s, 3H), 2.46-2.30 (m, 3H), 2.23 (s, 3H), 1.49-1.35 (m, 4H), 0.91 (t, *J* = 7.2 Hz, 3H).

^{13}C NMR (75 MHz, $CDCl_3$) δ 172.7, 151.9, 144.0, 132.0, 121.8, 118.7, 111.2, 39.1, 38.6, 35.6, 33.0, 19.7, 14.5, 14.1, 13.8.

IR (film): ν (cm^{-1}) 2961, 2930, 2222, 1721, 1620, 1585, 1548, 1440, 1409, 1380, 1337, 1239, 1173, 1108, 958, 867, 805, 742, 648, 578, 412.

HRMS (ESI, *m*/*z*) calcd for $C_{15}H_{21}N_3ONa$ $[M+Na]^+$: 282.1577, found: 282.1579.

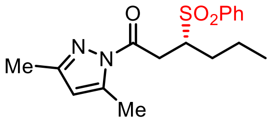

(*R*)-1-(3,5-Dimethyl-1*H*-pyrazol-1-yl)-3-(phenylsulfonyl)hexan-1-one (4k)

Enantiomeric excess of **4k** was established by HPLC analysis using a Daicel Chiralpak AD-H column, ee = 78% (HPLC: 254 nm, n-hexane/isopropanol = 95:5, flow rate 1.0 mL/min, 40 °C, t_r (major) = 13.0 min, t_r (minor) = 16.3 min). $[\alpha]_D^{22}$ = –7.0° (*c* 1.0, CH_2Cl_2).

^{1}H NMR (300 MHz, $CDCl_3$) δ 7.96-7.88 (m, 2H), 7.68-7.44 (m, 3H), 5.94 (s, 1H), 3.96-3.84 (m, 1H), 3.76 (dd, *J* = 17.6, 6.4 Hz, 1H), 3.24 (dd, *J* = 17.6, 5.8 Hz, 1H), 2.41 (s, 3H), 2.21 (s, 3H), 2.08-1.90 (m, 1H), 1.67-1.29 (m, 3H), 0.89 (t, *J* = 7.4 Hz, 3H).

^{13}C NMR (75 MHz, $CDCl_3$) δ 170.2, 152.4, 144.1, 137.6, 133.6, 129.1, 129.0, 111.4, 60.2, 34.4, 30.5, 19.9, 14.2, 13.8. 13.7.

IR (film): ν (cm^{-1}) 2963, 2931, 2874, 1723, 1586, 1445, 1381, 1303, 1176, 1141, 1082, 1030, 999, 961, 933, 808, 730, 691, 591, 563, 410.

HRMS (ESI, *m*/*z*) calcd for $C_{17}H_{22}N_2O_3SNa$ $[M+Na]^+$: 357.1243, found: 357.1247.

(16) Eq 1, reaction of 1d

According to the general procedure, the reaction of (*E*)-1-(3,5-dimethyl-1*H*-pyrazol-1-yl) hepta-2,6-dien-1-one **1d** (40.9 mg, 0.20 mmol), 2-((phenylsulfonyl)methyl)acrylonitrile **2a** (20.8 mg, 0.10 mmol), **Δ-RhO** (6.6 mg, 8.0 mol%), diethyl 2,6-diethyl-1,4-dihydropyridine-3,5-dicarboxylate (42.2 mg, 1.5 equiv) in 1,4-dioxane (1.0 mL, 0.1 M) under nitrogen atmosphere with visible light for 40 hours at 35 °C, afforded 16.5 mg (61%) of **3h** as a colorless oil, and afforded 21.5 mg (62%) of **4l** as a yellow oil.

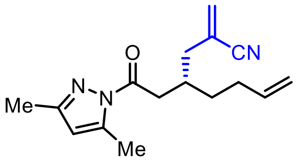

(*S*)-4-(2-(3,5-Dimethyl-1*H*-pyrazol-1-yl)-2-oxoethyl)-2-methyleneoct-7-enenitrile (3h)

Enantiomeric excess of **3h** was established by HPLC analysis using a Daicel Chiralpak OD-H column, ee = 79% (HPLC: 254 nm, n-hexane/isopropanol = 99:1, flow rate 1.0 mL/min, 40 °C, t_r (major) = 6.9 min, t_r (minor) = 5.7 min). $[\alpha]_D^{22}$ = +6.0° (*c* 1.0, CH_2Cl_2).

^{1}H NMR (300 MHz, $CDCl_3$) δ 5.95 (s, 1H), 5.90 (s, 1H), 5.87-5.71 (m, 2H), 5.07-4.93 (m, 2H), 3.08-3.02 (m, 2H), 2.53 (s, 3H), 2.48-2.32 (m, 3H), 2.23 (s, 3H), 2.18-2.08 (m, 2H), 1.58-1.48 (m, 2H).

^{13}C NMR (75 MHz, $CDCl_3$) δ 172.5, 152.0, 144.0, 137.9, 132.1, 121.6, 118.6, 115.1, 111.2, 39.0, 38.4, 32.8, 32.6, 30.7, 14.5, 13.8.

IR (film): ν (cm^{-1}) 2925, 2223, 1722, 1640, 1584, 1409, 1380, 1338, 1245, 1171, 1032, 959, 915, 806, 745, 644, 562, 410.

HRMS (ESI, *m*/*z*) calcd for $C_{16}H_{21}N_3ONa$ $[M+Na]^+$: 294.1577, found: 294.1580.

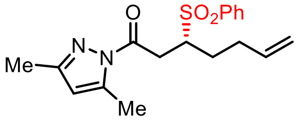

(*R*)-1-(3,5-Dimethyl-1*H*-pyrazol-1-yl)-3-(phenylsulfonyl)hept-6-en-1-one (4l)

Enantiomeric excess of **4l** was established by HPLC analysis using a Daicel Chiralpak AD-H column, ee = 83% (HPLC: 254 nm, n-hexane/isopropanol = 95:5, flow rate 1.0 mL/min, 40 °C, t_r (major) = 14.1 min, t_r (minor) = 17.2 min). $[\alpha]_D^{22}$ = –1.2° (*c* 1.0, CH_2Cl_2).

^{1}H NMR (300 MHz, $CDCl_3$) δ 7.95-7.89 (m, 2H), 7.66-7.48 (m, 3H), 5.94 (s, 1H), 5.78-5.62 (m, 1H), 5.03-4.93 (m, 2H), 3.98-3.86 (m, 1H), 3.78 (dd, *J* = 17.6, 6.4 Hz, 1H), 3.28 (dd, *J* = 17.6, 5.8 Hz, 1H), 2.41 (s, 3H), 2.22 (s, 3H), 2.26-2.04 (m, 3H), 1.76-1.64 (m, 1H).

^{13}C NMR (75 MHz, $CDCl_3$) δ 170.0, 152.4, 144.1, 137.6, 136.5, 133.7, 129.1, 129.0, 116.1, 111.4, 59.7, 34.4, 30.5, 27.7, 14.2, 13.7.

IR (film): ν (cm^{-1}) 2928, 1722, 1585, 1479, 1444, 1381, 1311, 1177, 1142, 1083, 1029, 916, 808, 732, 691, 588, 562, 410.

HRMS (ESI, *m*/*z*) calcd for $C_{18}H_{22}N_2O_3SNa$ $[M+Na]^+$: 369.1243, found: 369.1244.

(17) Eq 2

According to the general procedure, the reaction of (*E*)-1-(3,5-dimethyl-1*H*-pyrazol-1-yl) but-2-en-1-one **1a** (32.8 mg, 0.20 mmol), (*E*)-1,2,3,4,5-pentafluoro-6-(2-(phenylsulfonyl)vinyl) benzene **5** (25.7 mg, 0.10 mmol), Δ-**RhO** (6.6 mg, 8.0 mol%), diethyl 2,6-diethyl-1,4-dihydropyridine-3,5-dicarboxylate (42.2 mg, 1.5 equiv) in 1,4-dioxane (1.0 mL, 0.1 M) under nitrogen atmosphere with visible light for 48 hours at room temperature, afforded 21.1 mg (54%) of **6** as a colorless oil, and afforded 21.1 mg (59%) of **4a** as a yellow oil. Enantiomeric excess of **4a** was determined as 81% ee.

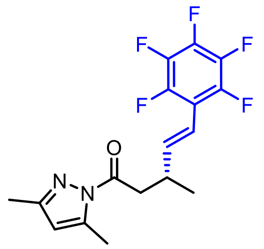

(*R*,*E*)-1-(3,5-Dimethyl-1*H*-pyrazol-1-yl)-3-methyl-5-(perfluorophenyl)pent-4-en-1-one (6)

Enantiomeric excess of **6** was established by HPLC analysis using a Daicel Chiralpak IG column, ee = 93% (HPLC: 254 nm, n-hexane/isopropanol = 99:1, flow rate 1.0 mL/min, 25 °C, t_r (major) = 11.4 min, t_r (minor) = 10.4 min). $[\alpha]_D^{22}$ =–12.0° (*c* 0.5, CH_2Cl_2).

^{1}H NMR (500 MHz, $CDCl_3$) δ 6.56 (dd, *J* = 16.4, 7.6 Hz, 1H), 6.33 (dd, *J* = 16.4, 1.0 Hz, 1H), 5.96 (s, 1H), 3.28 (dd, *J* = 16.4, 7.6 Hz, 1H), 3.17 (dd, *J* = 16.4, 6.8 Hz, 1H), 3.12-3.04 (m, 1H), 2.53 (s,

3H), 2.24 (s, 3H), 1.23 (d, J = 6.8 Hz, 3H).

^{13}C NMR (125 MHz, $CDCl_3$) δ 172.2, 152.0, 144.3 (br), 144.1, 113.4, 111.2, 41.6, 34.6, 20.0, 14.6, 13.8.

^{19}F NMR (282 MHz, $CDCl_3$) δ –143.40 - –143.60 (m, 2F), –157.51 - –157.71 (m, 1F), –163.40 - –163.64 (m, 2F).

IR (film): ν (cm^{-1}) 2923, 1728, 1583, 1520, 1494, 1459, 1412, 1378, 1353, 1317, 1248, 1136, 997, 967, 802, 746.

HRMS (ESI, *m/z*) calcd for $C_{17}H_{15}F_5N_2ONa$ [M+Na]$^+$: 381.0997, found: 381.0987.

8. Transformations of the Products

8.1 Reduction of (*S*)-4a

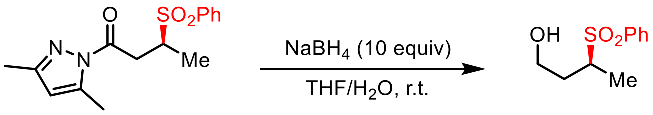

To a solution of (*S*)-1-(3,5-dimethyl-1*H*-pyrazol-1-yl)-3-(phenylsulfonyl)butan-1-one **(*S*)-4a** (*obtained by the reactions catalyzed by Λ-**RhO**,* 85% ee, 30.6 mg, 0.10 mmol) in THF/H_2O (4/1, 1.0 mL, 0.1 M) at 0 °C was added $NaBH_4$ (38.0 mg, 1.0 mmol). The reaction mixture was stirred at room temperature for 6 hours. The reaction was quenched with aqueous 2 N HCl (1.0 mL) at room temperature and extracted with CH_2Cl_2 (4×10 mL). The combined organic layers were dried over anhydrous Na_2SO_4, filtered, and concentrated under reduced pressure. The residue was purified by flash chromatography on silica gel (EtOAc/n-hexane = 1:1) to afford **7** (20.9 mg, 0.098 mmol, yield: 98%) as a white solid.

(*S*)-3-(Phenylsulfonyl)butan-1-ol (7)

Enantiomeric excess of **7** was established by HPLC analysis using a Daicel Chiralpak OD-H column, ee = 85% (HPLC: 254 nm, n-hexane/isopropanol = 90:10, flow rate 1.0 mL/min, 40 °C, t_r (major) = 19.1 min, t_r (minor) = 18.1 min). $[\alpha]_D^{22}$ = –15.8° (*c* 1.0, CH_2Cl_2).

^{1}H NMR (300 MHz, $CDCl_3$) δ 7.93-7.85 (m, 2H), 7.70-7.54 (m, 3H), 3.86 (dt, *J* = 11.0, 5.6 Hz, 1H), 3.69 (ddd, *J* = 11.0, 8.4, 5.0 Hz, 1H), 3.41-3.28 (m, 1H), 2.31-2.17 (m, 2H), 1.69 (ddt, *J* = 14.1, 8.8, 5.2 Hz, 1H), 1.27 (d, *J* = 6.9 Hz, 3H).

^{13}C NMR (75 MHz, $CDCl_3$) δ 137.2, 133.7, 129.1, 129.0, 59.3, 57.1, 32.2, 13.7.

IR (film): ν (cm^{-1}) 3341, 3208, 3143, 2928, 2876, 1581, 1447, 1380, 1294, 1140, 1060, 1005, 796, 766, 731, 691, 663, 589, 547.

HRMS (ESI, *m*/*z*) calcd for $C_{10}H_{14}O_3SNa$ $[M+Na]^+$: 237.0564, found: 237.0556.

8.2 Transamidation to 8 for Single Crystal X-ray Diffraction

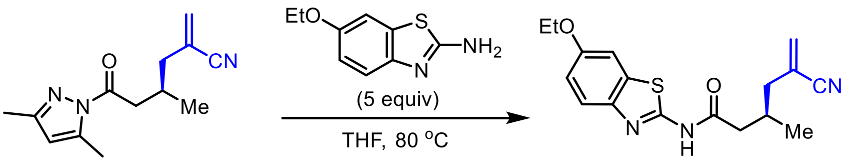

To a solution of (*R*)-6-(3,5-dimethyl-1*H*-pyrazol-1-yl)-4-methyl-2-methylene-6-oxohexanenitrile

(*R*)-3a (<u>*obtained by the reactions catalyzed by Λ-**RhO**,*</u> 96% ee, 23.1 mg, 0.10 mmol) in THF (0.1 mL, 1 M) was added 6-ethoxybenzo[*d*]thiazol-2-amine (97.0 mg, 0.50 mmol). The reaction mixture was stirred at 80 °C for 40 hours. After the start material was converted completely, the reaction mixture was cooled to room temperature and concentrated under reduced pressure. The residue was purified by flash chromatography on silica gel (EtOAc/n-hexane = 1:1) to afford **8** (29.6 mg, 90% yield) as a white solid.

(*R*)-5-Cyano-*N*-(6-ethoxybenzo[*d*]thiazol-2-yl)-3-methylhex-5-enamide (8)

Enantiomeric excess of **8** was established by HPLC analysis using a Daicel Chiralpak OD-H column, ee = 96% (HPLC: 254 nm, n-hexane/isopropanol = 85:15, flow rate 1.0 mL/min, 40 °C, t_r (major) = 30.6 min, t_r (minor) = 22.9 min). $[\alpha]_D^{22}$ = –8.0° (*c* 1.0, CH_2Cl_2).

^{1}H NMR (300 MHz, $CDCl_3$) δ 11.24 (brs, 1H), 7.64 (d, *J* = 8.8 Hz, 1H), 7.31 (d, *J* = 2.5 Hz, 1H), 7.06 (dd, *J* =8.8, 2.5 Hz, 1H), 5.85 (s, 1H), 5.66 (s, 1H), 4.10 (q, *J* = 7.0, 2H), 2.55-2.27 (m, 4H), 2.12 (dd, *J* = 13.6, 7.0, 1H), 1.45 (t, *J* = 7.0, 3H), 0.98 (d, *J* = 6.1 Hz, 3H).

^{13}C NMR (75 MHz, $CDCl_3$) δ 169.9, 157.3, 156.3, 141.8, 133.2, 132.4, 121.1, 121.0, 118.4, 115.9, 105.3, 64.2, 41.2, 41.0, 29.2, 19.0, 14.8.

IR (film): *v* (cm^{-1}) 3182, 2975, 2921, 2218, 2047, 1688, 1605, 1551, 1460, 1259, 1223, 1114, 1060, 942, 822, 747, 666.

HRMS (ESI, *m*/*z*) calcd for $C_{17}H_{19}N_3O_2SNa$ $[M+Na]^+$: 330.1271, found: 330.1264.

8.3 Transamidation of (*R*)-3a with 4-Methoxyaniline

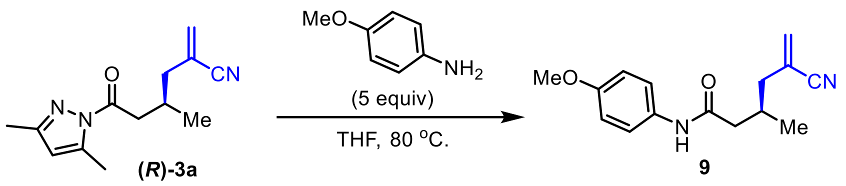

To a solution of (*R*)-6-(3,5-dimethyl-1*H*-pyrazol-1-yl)-4-methyl-2-methylene-6-oxohexanenitrile **(*R*)-3a** (<u>*obtained by the reactions catalyzed by Λ-**RhO**,*</u> 96% ee, 23.1 mg, 0.10 mmol) in THF (0.1 mL, 1 M) was added 4-methoxyaniline (62.0 mg, 0.50 mmol). The reaction mixture was stirred at 80 °C for 24 hours. After the starting material was consumed completely, the reaction mixture was cooled to room temperature and concentrated under reduced pressure. The residue was purified by flash chromatography on silica gel (EtOAc/n-hexane = 1:2) to afford **9** (24.3 mg, 94% yield) as a white solid.

(*R*)-5-Cyano-*N*-(4-methoxyphenyl)-3-methylhex-5-enamide (9)

Enantiomeric excess of **9** was established by HPLC analysis using a Daicel Chiralpak OD-H column, ee = 96% (HPLC: 254 nm, n-hexane/isopropanol = 90:10, flow rate 1.0 mL/min, 40 °C, t_r (major) = 40.1 min, t_r (minor) = 36.2 min). $[\alpha]_D^{22}$ = –2.4° (*c* 1.0, CH_2Cl_2).

^{1}H NMR (300 MHz, $CDCl_3$) δ 7.44-7.36 (m, 2H), 7.24 (brs, 1H), 6.89-6.83 (m, 2H), 5.94 (s, 1H), 5.77 (s, 1H), 3.78 (s, 3H), 2.47-2.33 (m, 3H), 2.28-2.16 (m, 2H), 1.07 (d, *J* = 6.2 Hz, 3H).

^{13}C NMR (75 MHz, $CDCl_3$) δ 169.4, 156.5, 132.5, 130.8, 121.9, 121.2, 119.0, 114.2, 55.5, 43,4, 41.0, 29.8, 19.1.

IR (film): ν (cm^{-1}) 3299, 2960, 2837, 2226, 2180, 1655, 1605, 1536, 1509, 1460, 1236, 1171, 1032, 939, 829, 638, 524.

HRMS (ESI, *m*/*z*) calcd for $C_{15}H_{18}N_2O_2Na$ $[M+Na]^+$: 281.1260, found: 281.1254.

9. HPLC Traces of the Products

Enantiomeric purities of the reaction products were determined with a Daicel Chiralpak OD-H, IA, IG or Chiralcel OJ-H column (250 × 4.6 mm) on an Agilent 1200 or 1260 Series HPLC System using n-hexane/isopropanol as a mobile phase. The column temperature was 25 °C or 40 °C and UV-absorption was measured at 254 nm.

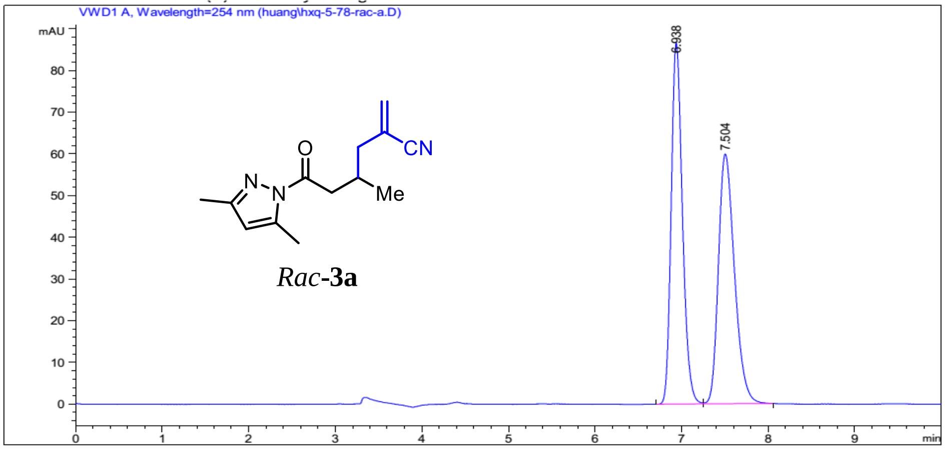

Peak #	RetTime [min]	Type	Width [min]	Area [mAU*s]	Height [mAU]	Area %
1	6.938	BV	0.1386	779.92273	86.56280	50.2042
2	7.504	VV R	0.1946	773.57709	59.91864	49.7958
Totals :				1553.49982	146.48144	

Figure S9. HPLC traces of *rac*-**3a**.

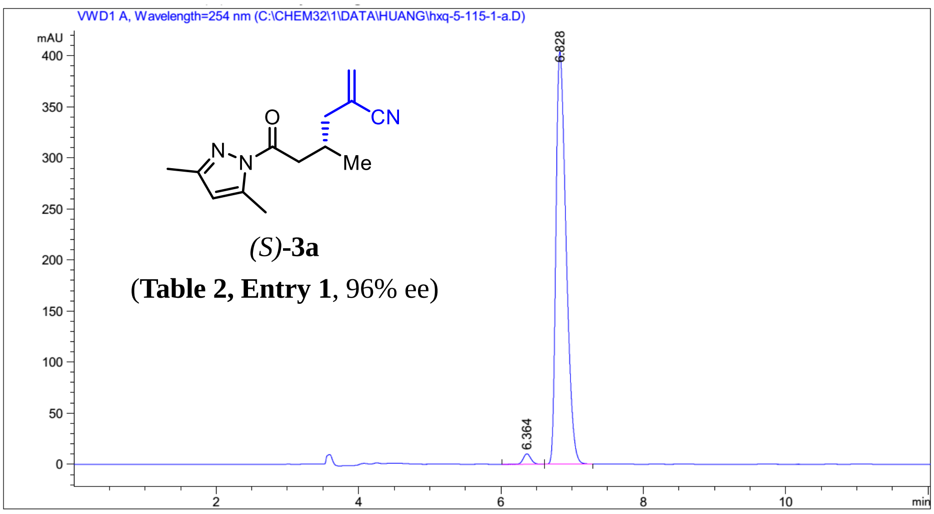

Peak #	RetTime [min]	Type	Width [min]	Area [mAU*s]	Height [mAU]	Area %
1	6.364	VB R	0.1138	77.47672	10.23726	1.9041
2	6.828	BB	0.1532	3991.37061	404.04858	98.0959

Figure S10. HPLC traces of (*S*)-**3a** (**Table 2, Entry 1**).

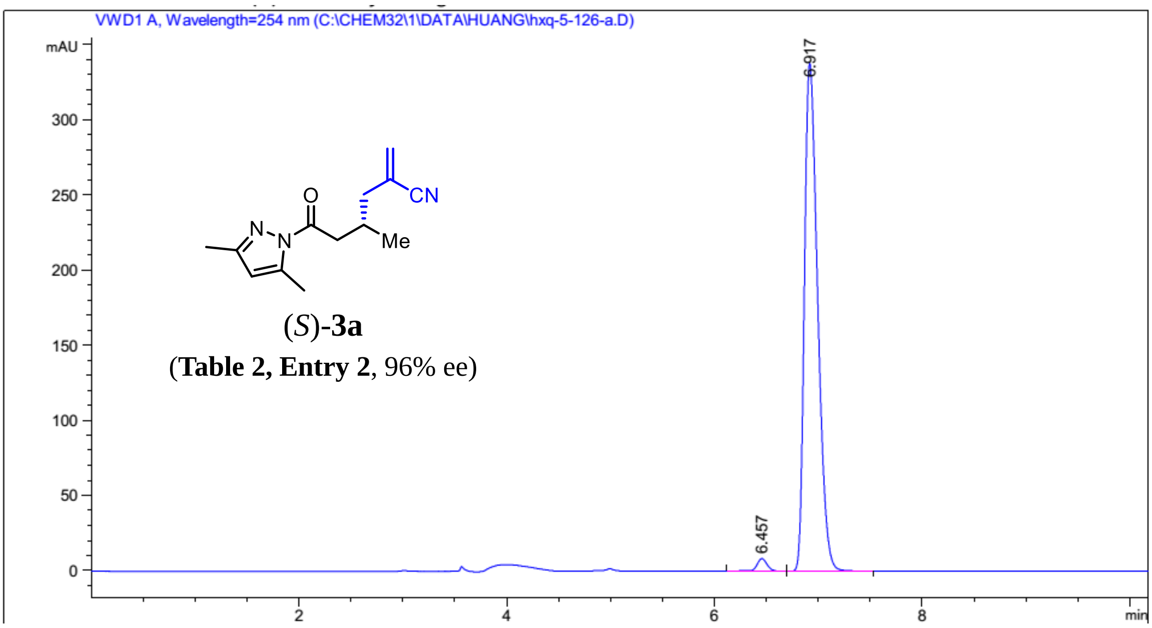

Peak #	RetTime [min]	Type	Width [min]	Area [mAU*s]	Height [mAU]	Area %
1	6.457	VB R	0.1029	58.44178	8.43045	1.9182
2	6.917	BB	0.1377	2988.19922	337.81195	98.0818

Figure S11. HPLC traces of (*S*)-**3a** (**Table 2, Entry 2**).

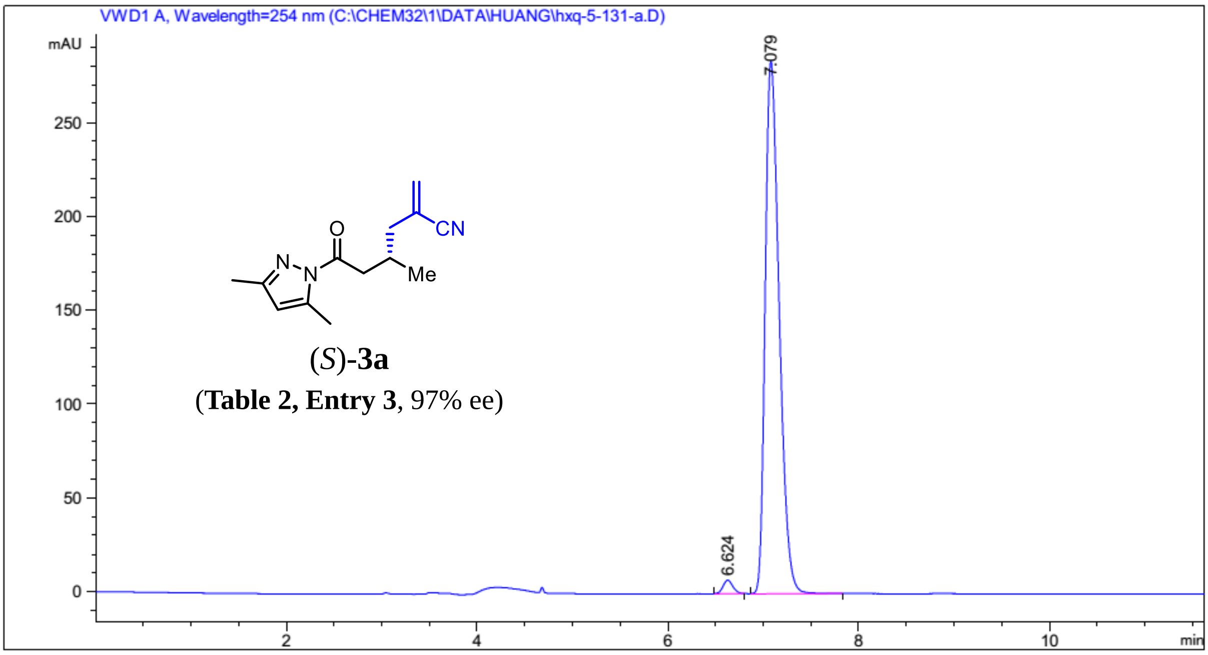

Peak #	RetTime [min]	Type	Width [min]	Area [mAU*s]	Height [mAU]	Area %
1	6.624	MM R	0.1191	50.91529	7.12533	1.7310
2	7.079	FM R	0.1699	2890.52344	283.61243	98.2690

Figure S12. HPLC traces of (*S*)-**3a** (**Table 2, Entry 3**).

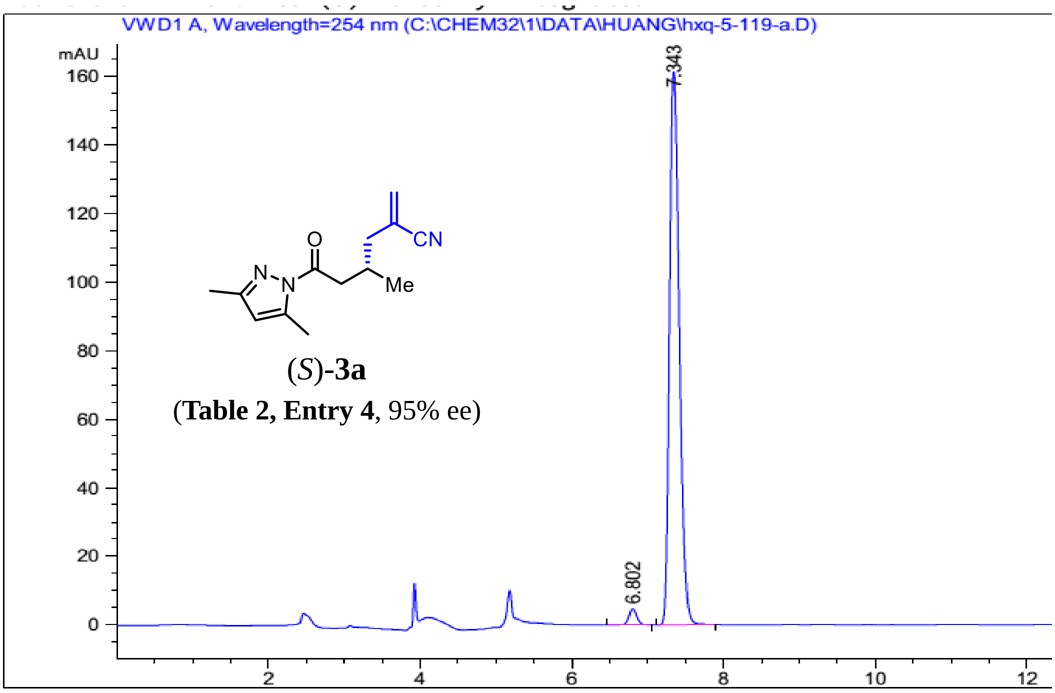

Peak #	RetTime [min]	Type	Width [min]	Area [mAU*s]	Height [mAU]	Area %
1	6.802	VV R	0.1039	33.69920	4.68674	2.3032
2	7.343	BB	0.1375	1429.41956	161.12549	97.6968

Figure S13. HPLC traces of (*S*)-**3a** (**Table 2, Entry 4**).

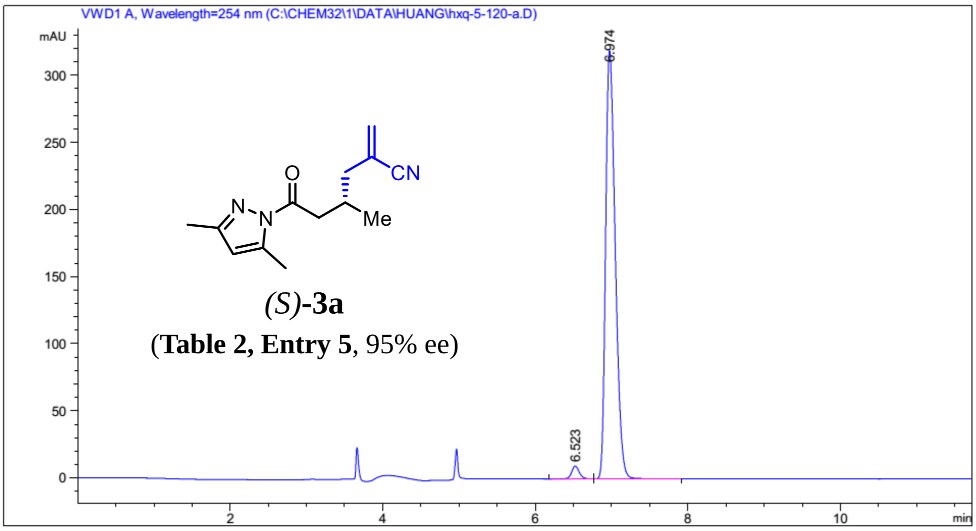

Peak #	RetTime [min]	Sig	Type	Area [mAU*s]	Height [mAU]	Area %
1	6.523	1	BV	68.42435	9.57870	2.3923
2	6.974	1	VB	2791.80713	318.67108	97.6077
Totals :				2860.23148	328.24978	

Figure S14. HPLC traces of (*S*)-**3a** (**Table 2, Entry 5**).

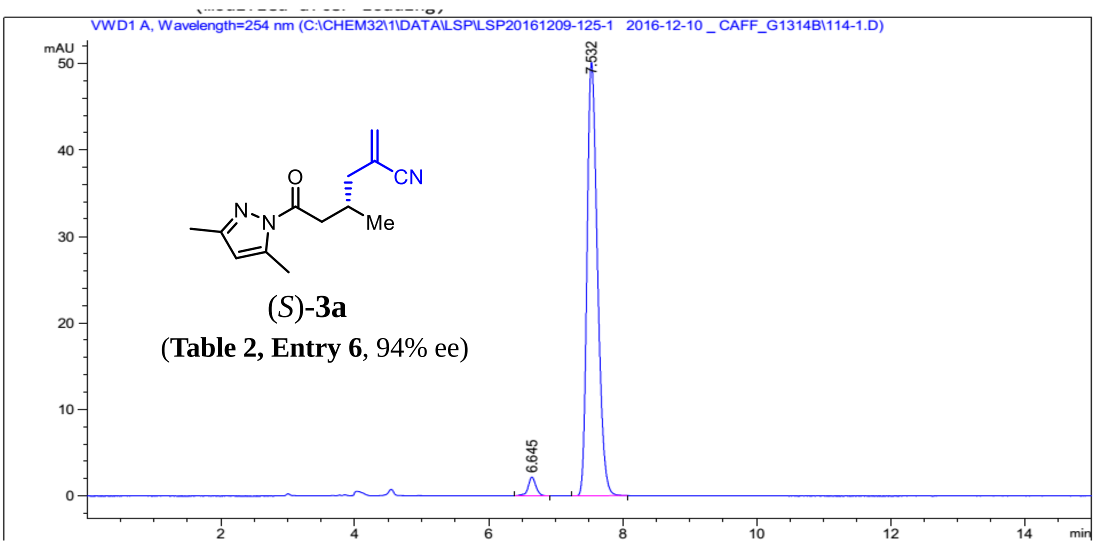

Peak #	RetTime [min]	Type	Width [min]	Area [mAU*s]	Height [mAU]	Area %
1	6.645	BB	0.1088	18.09816	2.20478	3.2282
2	7.532	BB	0.1648	542.52759	50.12337	96.7718
Totals :				560.62574	52.32815	

Figure S15. HPLC traces of (*S*)-**3a** (**Table 2, Entry 6**).

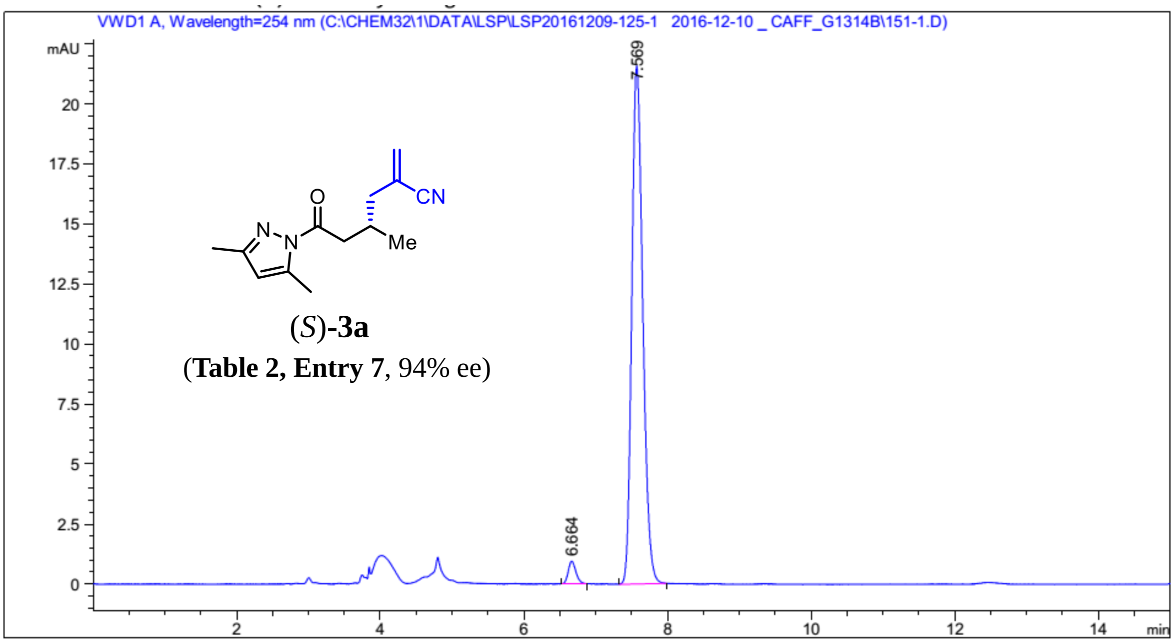

Peak #	RetTime [min]	Type	Width [min]	Area [mAU*s]	Height [mAU]	Area %
1	6.664	MM	0.1242	7.07298	9.49085e-1	2.9934
2	7.569	BB	0.1554	229.21523	21.57634	97.0066
Totals :				236.28821	22.52542	

Figure S16. HPLC traces of (*S*)-**3a** (**Table 2, Entry 7**).

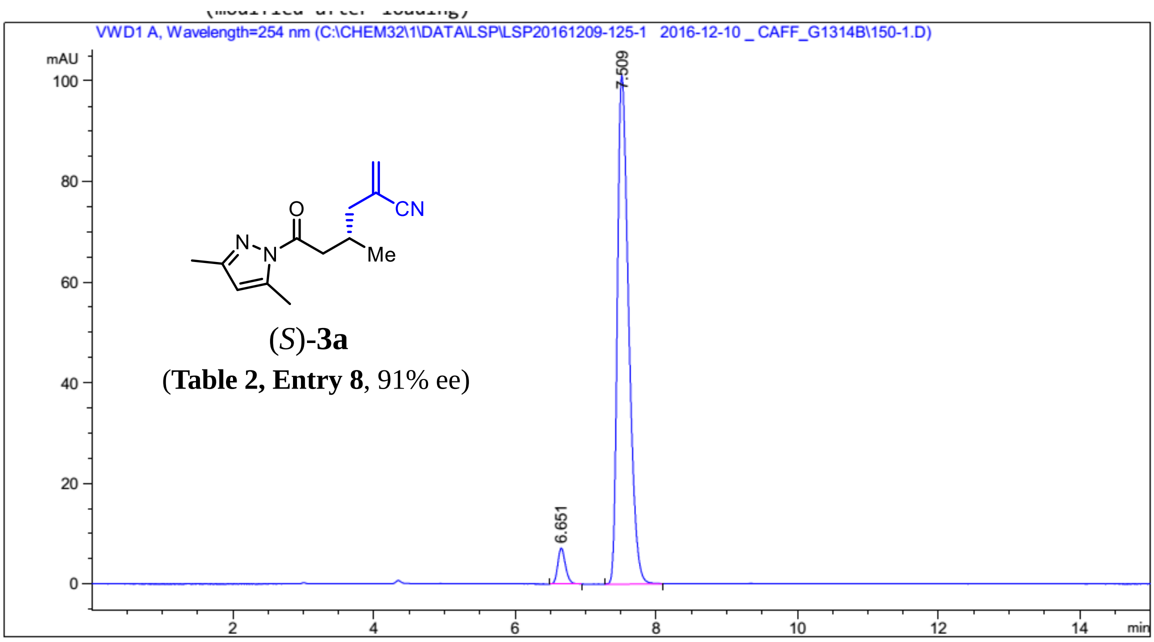

Peak #	RetTime [min]	Type	Width [min]	Area [mAU*s]	Height [mAU]	Area %
1	6.651	BB	0.1152	54.52230	7.17371	4.6467
2	7.509	BB	0.1686	1118.82678	101.14517	95.3533
Totals :				1173.34908	108.31888	

Figure S17. HPLC traces of (*S*)-**3a** (**Table 2, Entry 8**).

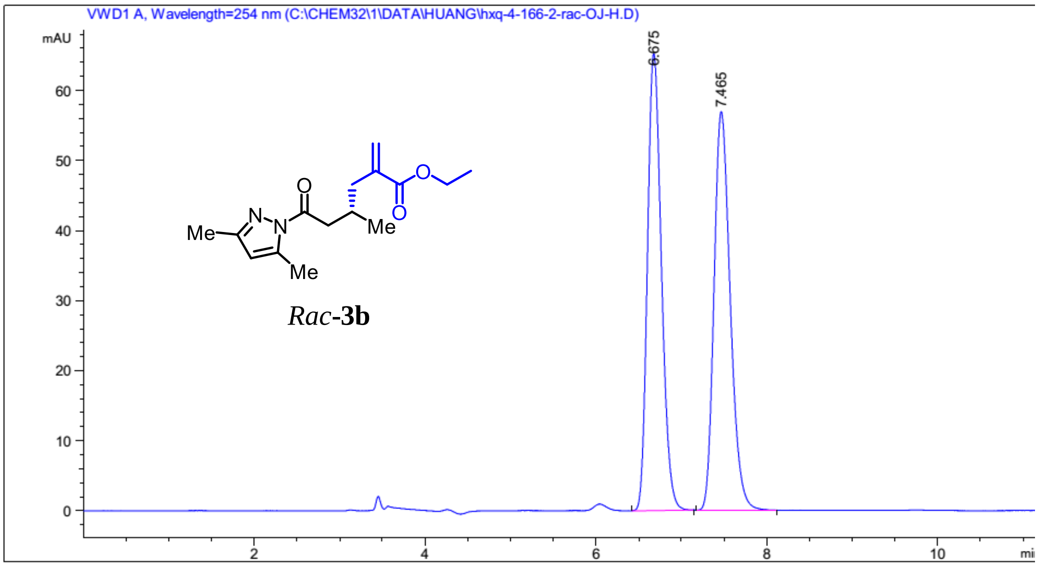

Peak #	RetTime [min]	Type	Width [min]	Area [mAU*s]	Height [mAU]	Area %
1	6.675	BB	0.1717	739.68506	65.26010	49.6411
2	7.465	BB	0.1964	750.38104	56.89471	50.3589

Figure S18. HPLC traces of *rac*-**3b**.

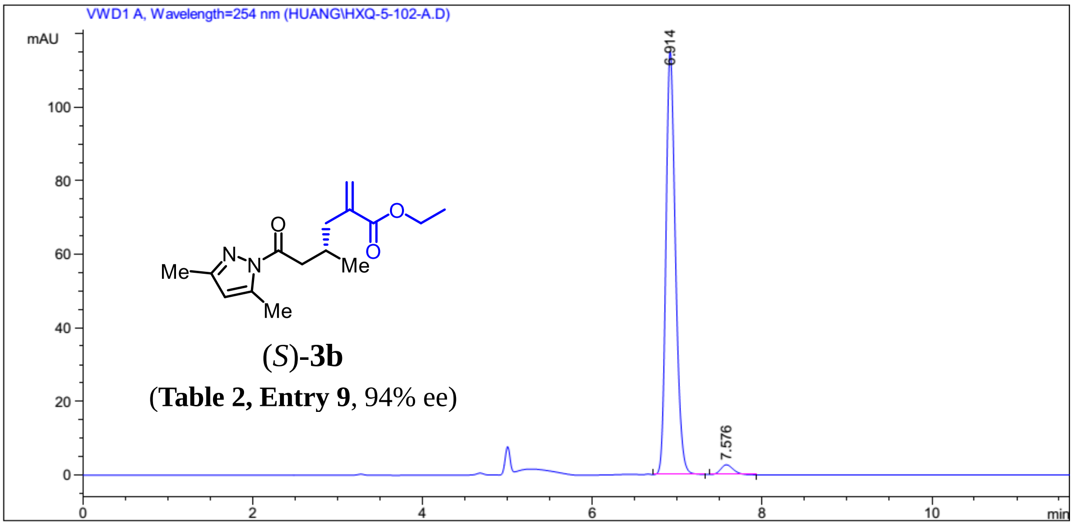

Peak #	RetTime [min]	Type	Width [min]	Area mAU *s	Height [mAU]	Area %
1	6.914	VB	0.1203	901.05347	115.32792	96.8434
2	7.576	BB	0.1644	29.36975	2.73962	3.1566

Figure S19. HPLC traces of (*S*)-**3b** (**Table 2, Entry 9**).

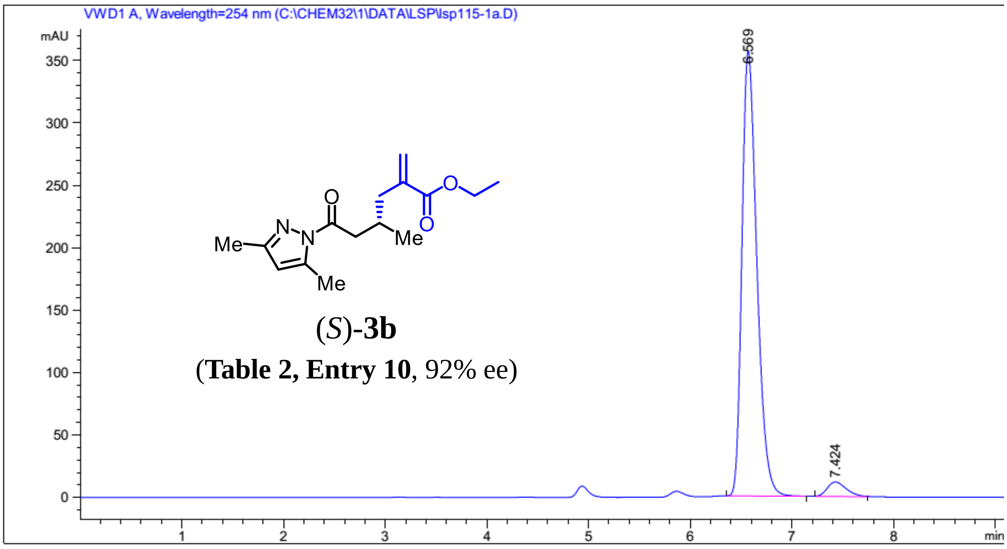

Peak #	RetTime [min]	Type	Width [min]	Area [mAU*s]	Height [mAU]	Area %
1	6.569	MM R	0.1697	3628.60669	356.30719	96.0379
2	7.424	MM R	0.2159	149.69856	11.55703	3.9621
Totals :				3778.30525	367.86422	

Figure S20. HPLC traces of (*S*)-**3b** (**Table 2, Entry 10**).

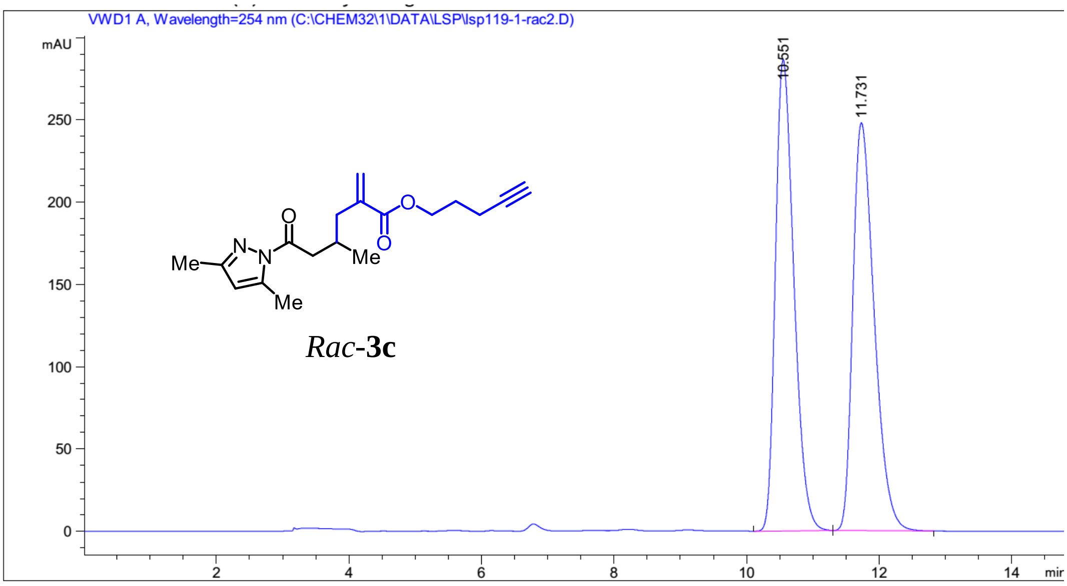

Peak #	RetTime [min]	Sig	Type	Area [mAU*s]	Height [mAU]	Area %
1	10.551	1	BB	5541.22998	286.22028	49.9872
2	11.731	1	BV R	5544.07178	247.74680	50.0128
Totals :				1.10853e4	533.96707	

Figure S21. HPLC traces of *rac*-**3c**.

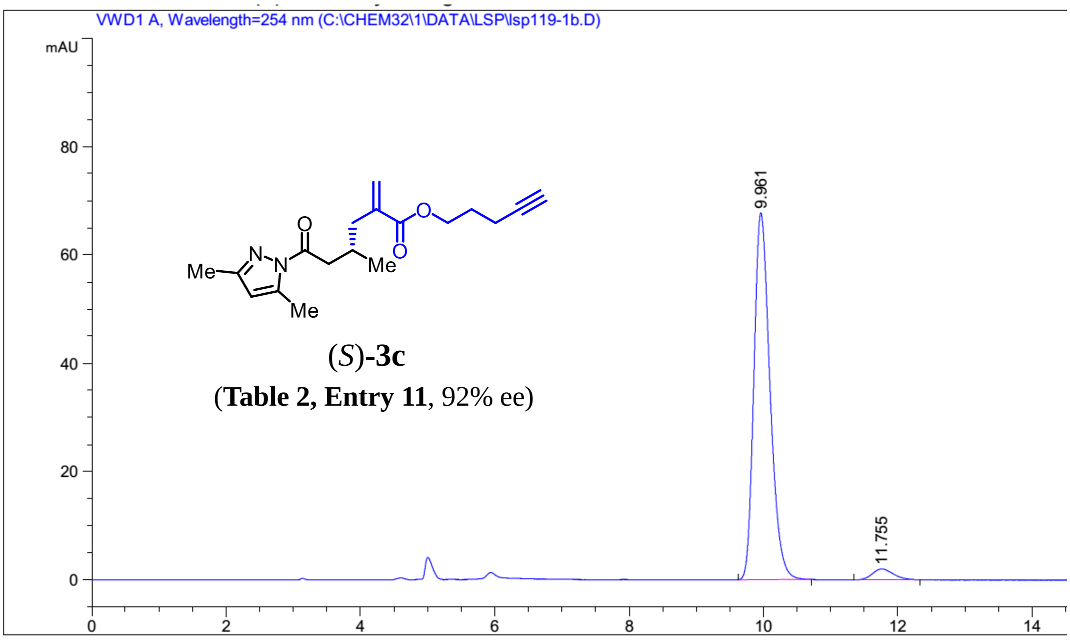

Peak #	RetTime [min]	Sig	Type	Area [mAU*s]	Height [mAU]	Area %
1	9.961	1	BB	1126.57617	67.71008	96.2203
2	11.755	1	BB	44.25342	2.02225	3.7797
Totals :				1170.82959	69.73233	

Figure S22. HPLC traces of (*S*)-**3c** (**Table 2, Entry 11**).

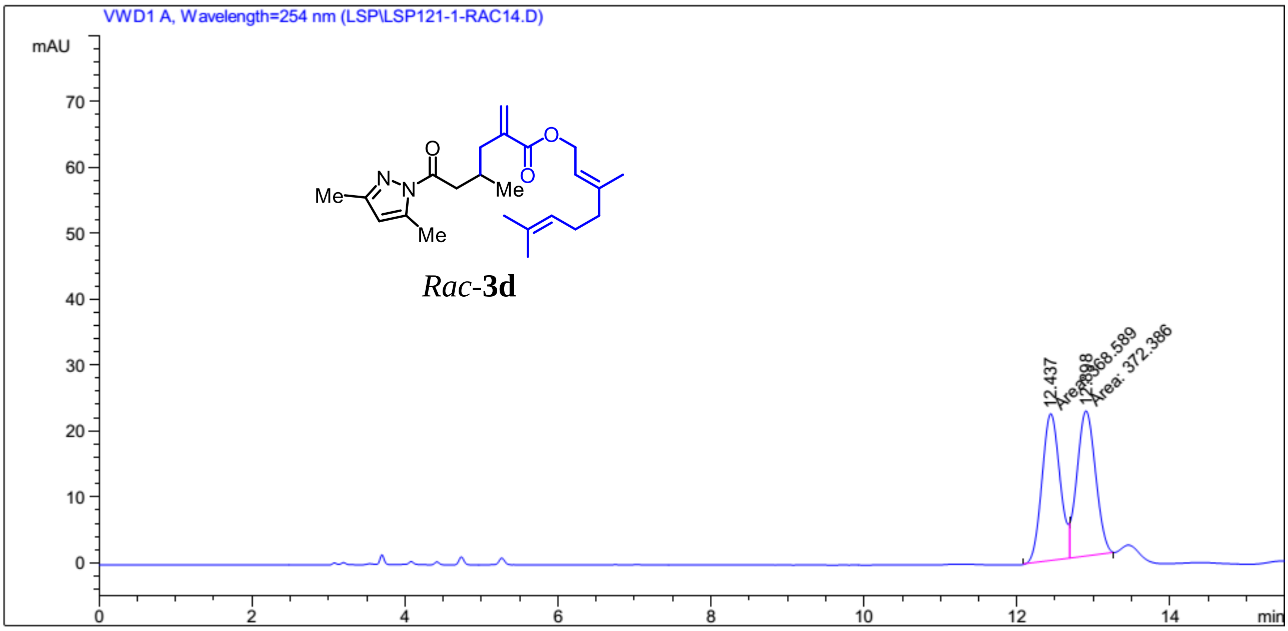

Peak #	RetTime [min]	Type	Width [min]	Area mAU *s	Height [mAU]	Area %
1	12.437	MF	0.2752	368.58881	22.32586	49.7438
2	12.898	FM	0.2812	372.38577	22.06785	50.2562

Figure S23. HPLC traces of *rac*-**3d.**

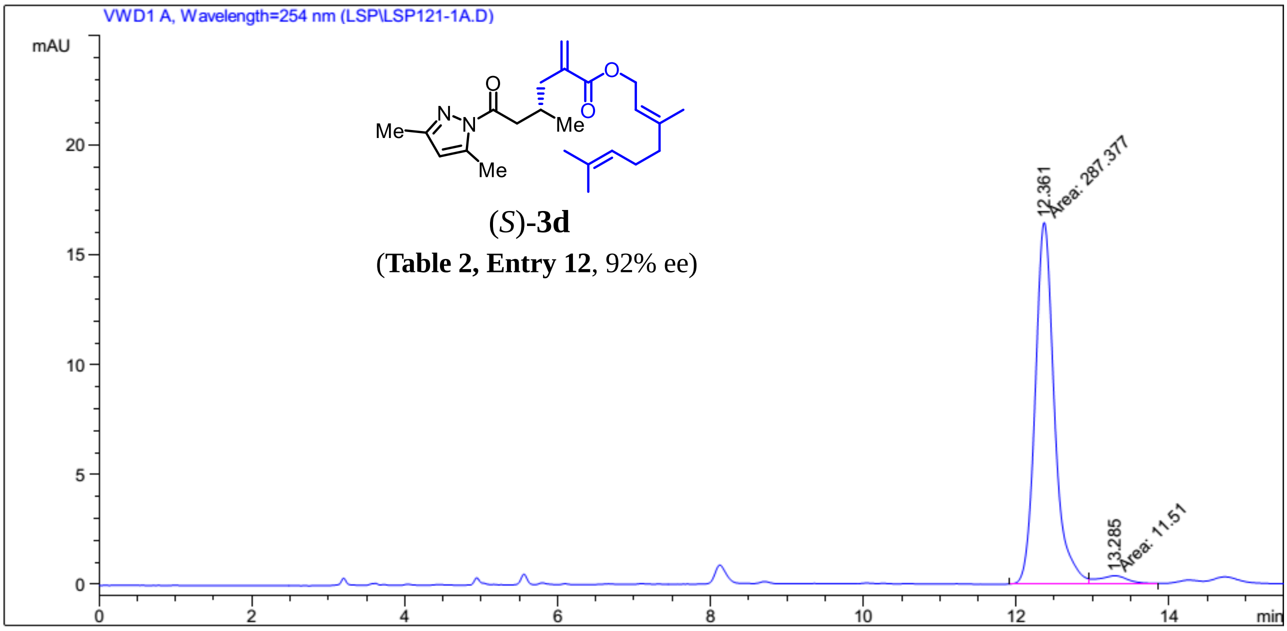

Peak #	RetTime [min]	Type	Width [min]	Area mAU *s	Height [mAU]	Area %
1	12.361	MM	0.2907	287.37656	16.47365	96.1490
2	13.285	MM	0.4900	11.51003	3.91491e-1	3.8510

Figure S24. HPLC traces of (*S*)-**3d** (**Table 2, Entry 12**).

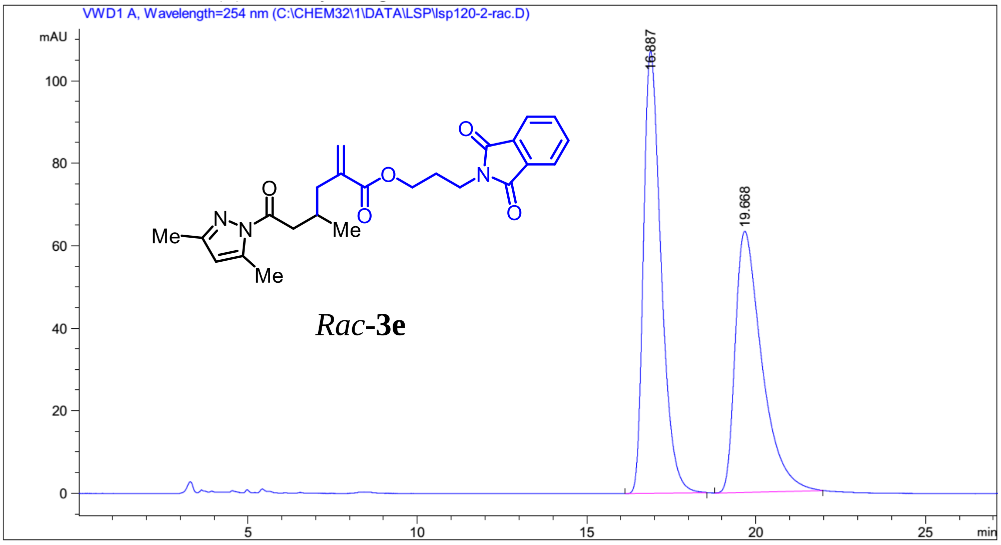

Peak #	RetTime [min]	Sig	Type	Area [mAU*s]	Height [mAU]	Area %
1	16.887	1	VV R	3883.58691	107.15952	52.8770
2	19.668	1	BV R	3460.98145	63.28626	47.1230
Totals :				7344.56836	170.44577	

Figure S25. HPLC traces of *rac*-**3e**.

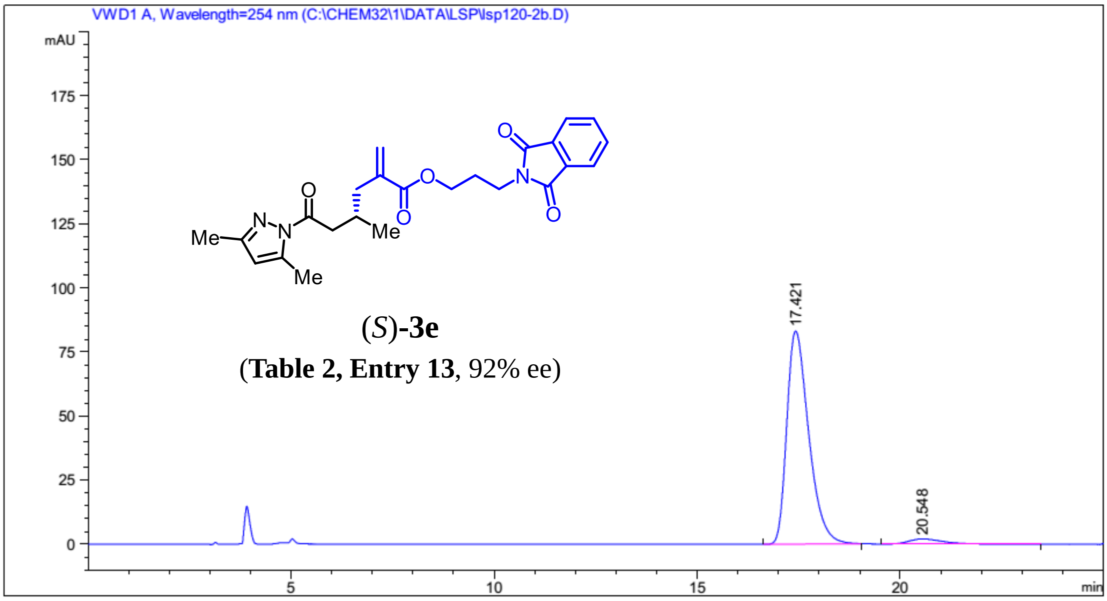

Peak #	RetTime [min]	Sig	Type	Area [mAU*s]	Height [mAU]	Area %
1	17.421	1	VB R	3119.23413	83.22392	96.0932
2	20.548	1	MM	126.81680	2.04945	3.9068
Totals :				3246.05093	85.27336	

Figure S26. HPLC traces of (*S*)-**3e** (**Table 2, Entry 13**).

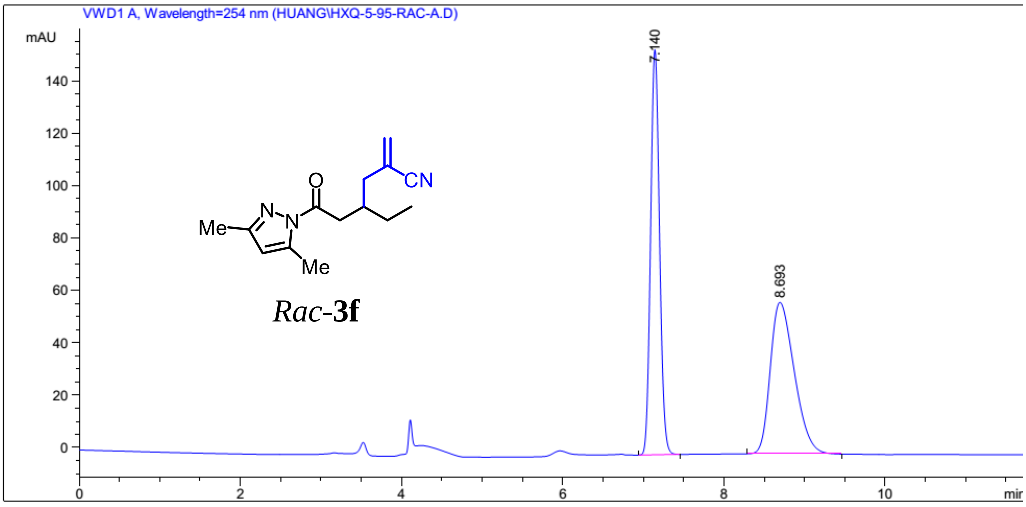

Peak #	RetTime [min]	Type	Width [min]	Area mAU *s	Height [mAU]	Area %
1	7.140	BB	0.1203	1190.85168	154.77521	49.7364
2	8.693	BB	0.3239	1203.47632	57.92344	50.2636

Totals : 2394.32800 212.69865

Figure S27. HPLC traces of *rac*-**3f**.

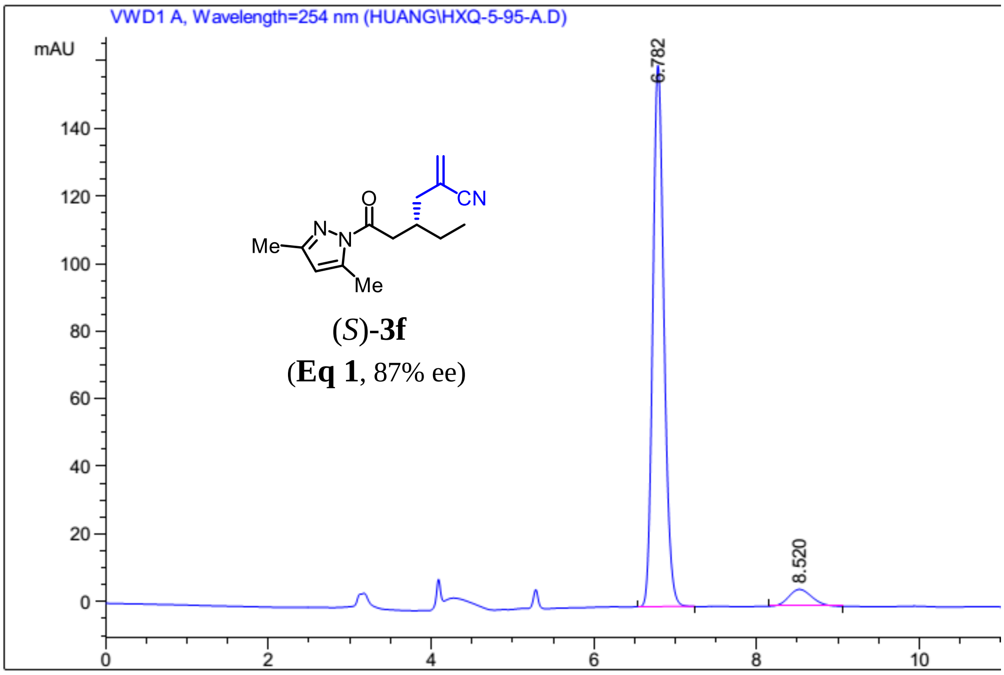

Peak #	RetTime [min]	Type	Width [min]	Area mAU *s	Height [mAU]	Area %
1	6.782	BB	0.1474	1524.52710	160.19037	93.6970
2	8.520	BB	0.3168	102.55573	5.05349	6.3030

Totals : 1627.08283 165.24386

Figure S28. HPLC traces of (*S*)-**3f** (**Eq 1**).

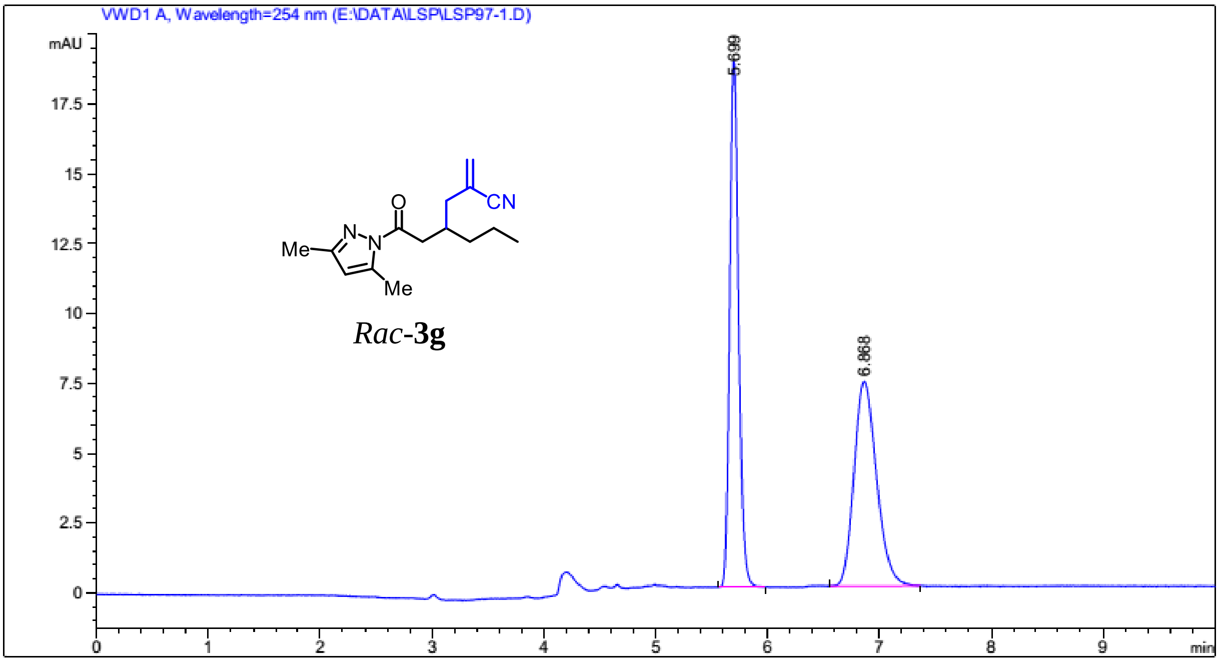

Peak #	RetTime [min]	Type	Width [min]	Area [mAU*s]	Height [mAU]	Area %
1	5.699	BB	0.0842	103.63793	18.82326	50.1234
2	6.868	BV R	0.1682	103.12770	7.32530	49.8766

Figure S29. HPLC traces of *rac*-**3g**.

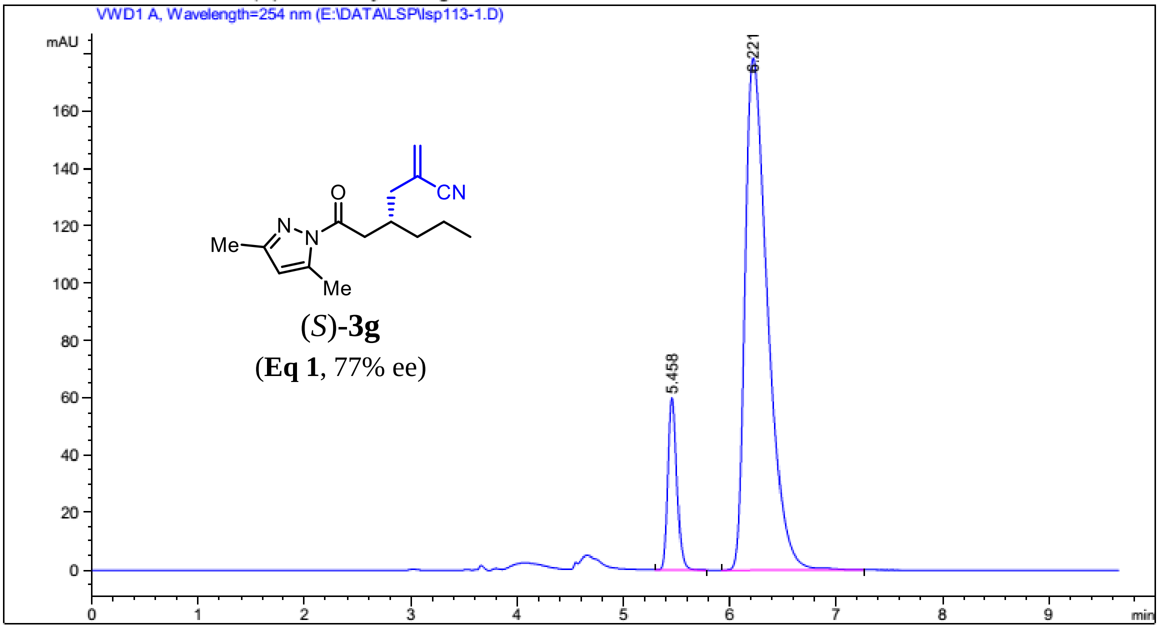

Peak #	RetTime [min]	Type	Width [min]	Area [mAU*s]	Height [mAU]	Area %
1	5.458	BV R	0.0891	349.98740	59.99574	11.6823
2	6.221	BV R	0.2248	2645.89185	178.58705	88.3177

Figure S30. HPLC traces of (*S*)-**3g** (**Eq 1**).

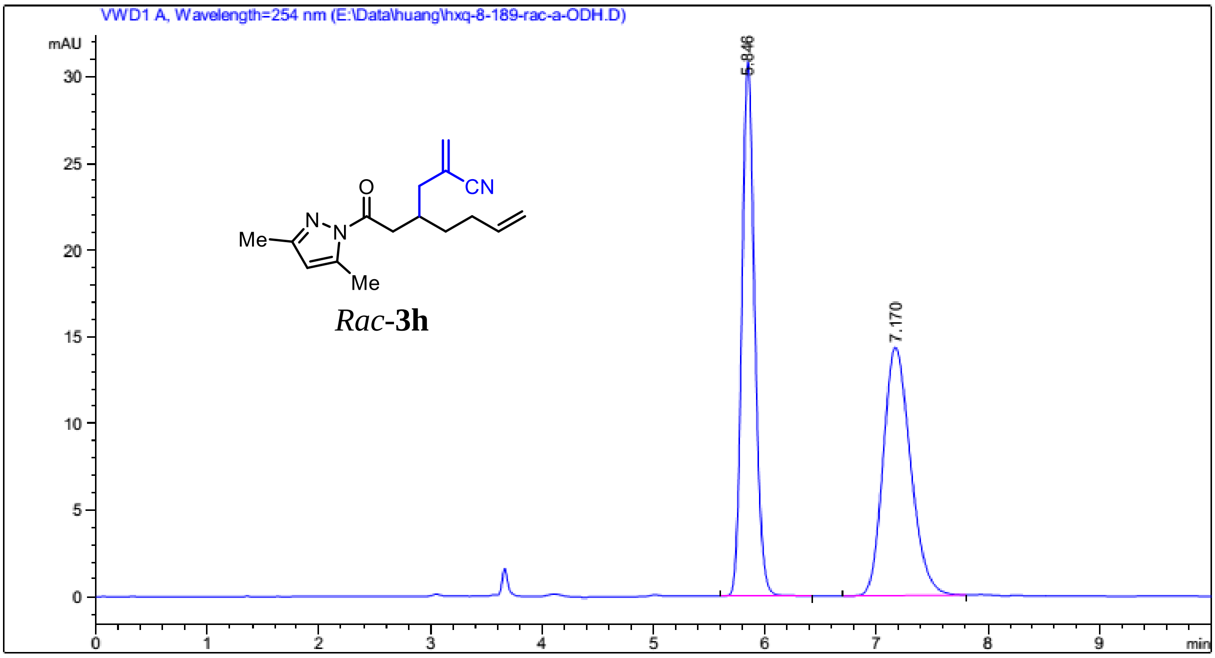

Peak #	RetTime [min]	Type	Width [min]	Area [mAU*s]	Height [mAU]	Area %
1	5.846	BB	0.1231	246.27420	30.86311	50.1533
2	7.170	BB	0.2644	244.76874	14.32068	49.8467

Figure S31. HPLC traces of *rac*-**3h**.

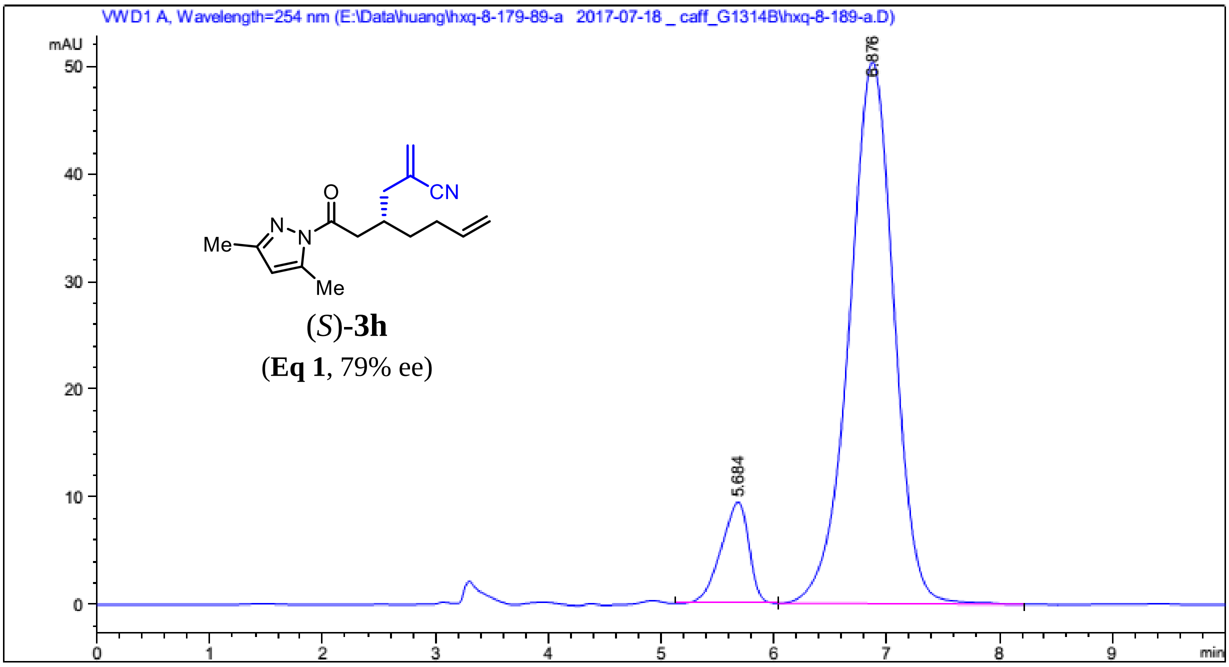

Peak #	RetTime [min]	Type	Width [min]	Area [mAU*s]	Height [mAU]	Area %
1	5.684	BB	0.2630	161.96022	9.40255	10.4686
2	6.876	BB	0.4219	1385.14258	50.35348	89.5314

Figure S32. HPLC traces of (*S*)-**3h** (**Eq 1**)

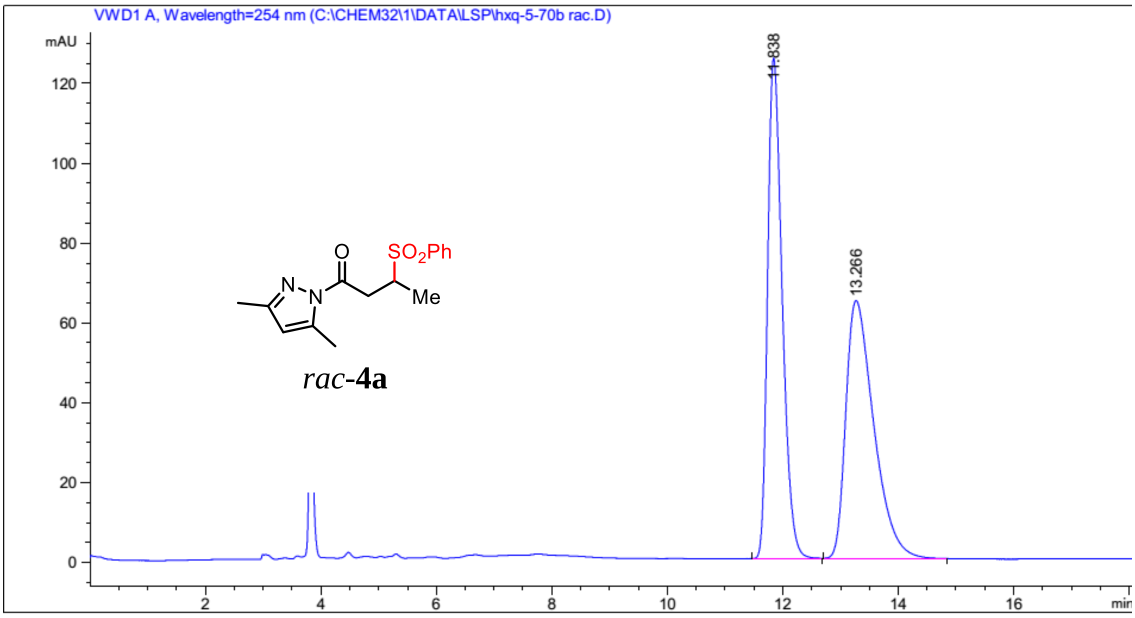

Peak #	RetTime [min]	Type	Width [min]	Area [mAU*s]	Height [mAU]	Area %
1	11.838	BB	0.2636	2195.37134	125.46828	50.2529
2	13.266	BB	0.4598	2173.27686	64.63612	49.7471

Figure S33. HPLC traces of *rac*-**4a**.

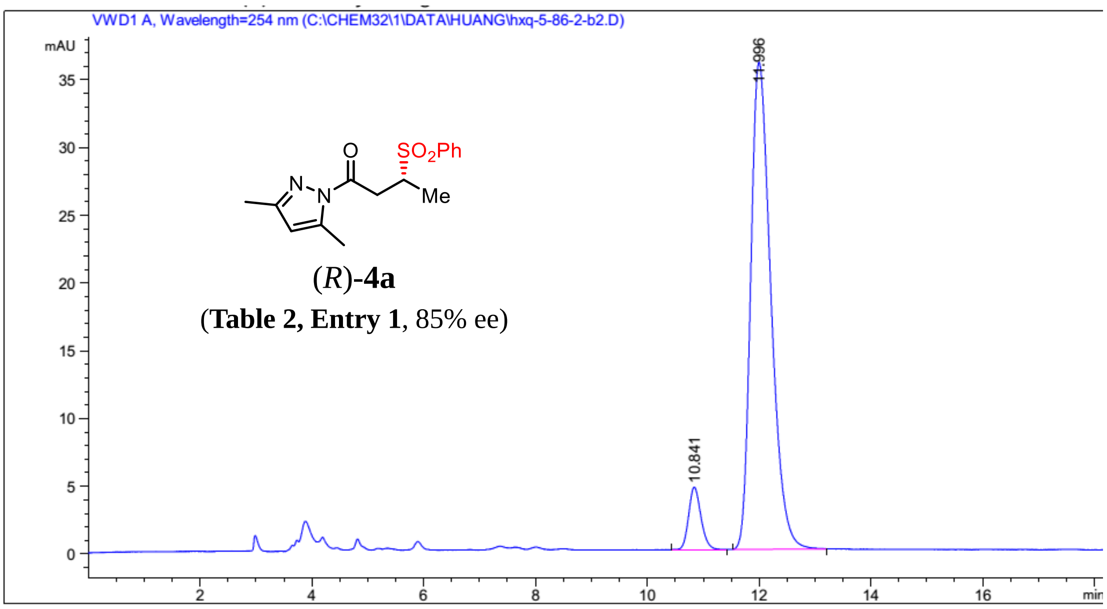

Peak #	RetTime [min]	Type	Width [min]	Area [mAU*s]	Height [mAU]	Area %
1	10.841	MM R	0.2565	71.10545	4.61941	7.3384
2	11.996	MM R	0.4161	897.84430	35.96385	92.6616

Figure S34. HPLC traces of (*R*)-**4a** (**Table 2, Entry 1**).

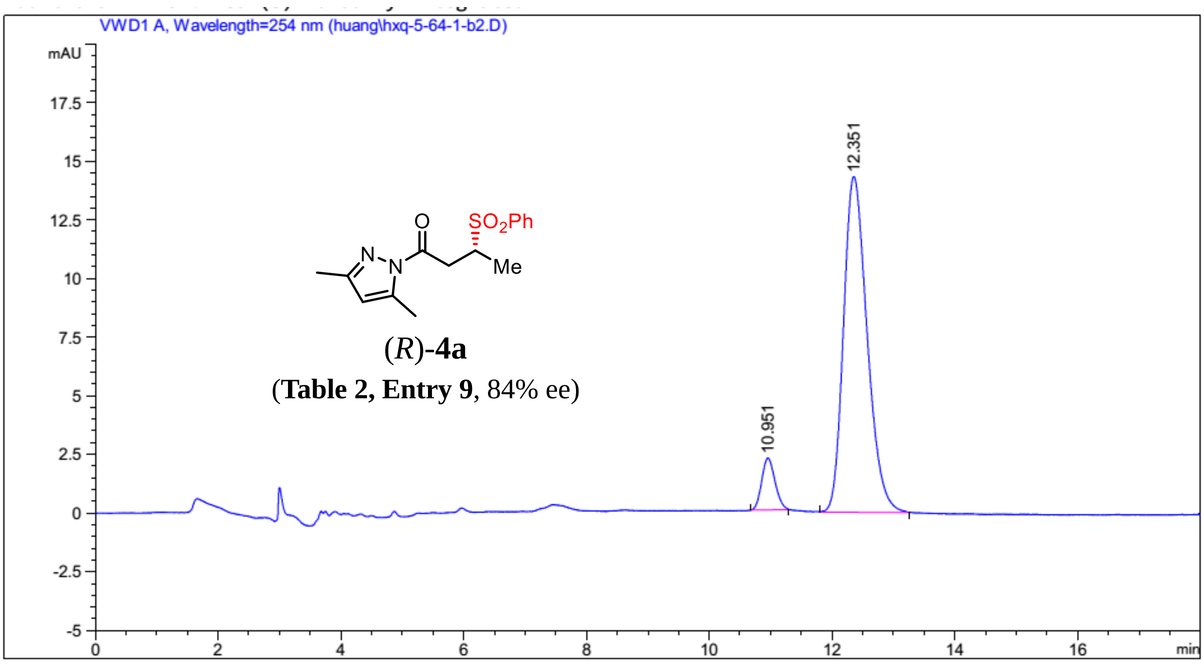

Peak #	RetTime [min]	Type	Width [min]	Area [mAU*s]	Height [mAU]	Area %
1	10.951	MM R	0.2518	33.53619	2.21951	8.0776
2	12.351	BB	0.3140	381.63998	14.31781	91.9224

Figure S35. HPLC traces of (*R*)-**4a** (**Table 2, Entry 9**).

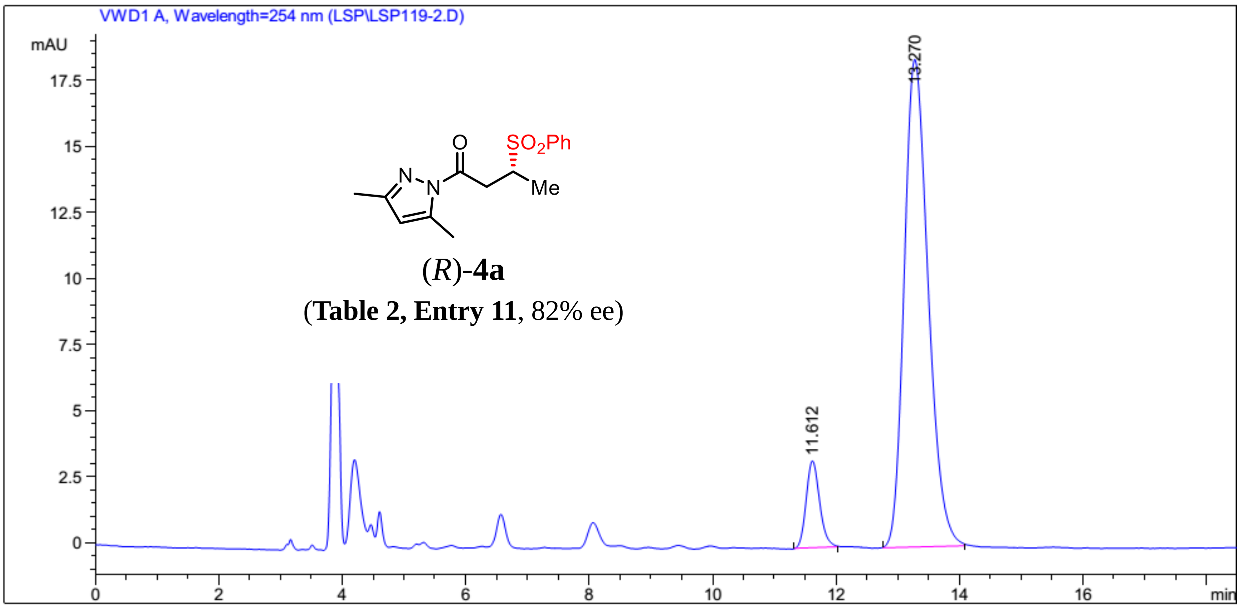

Peak #	RetTime [min]	Type	Width [min]	Area mAU *s	Height [mAU]	Area %
1	11.612	BB	0.2334	50.28230	3.29439	9.1745
2	13.270	BB	0.4167	497.78632	18.44817	90.8255
Totals :				548.06861	21.74256	

Figure S36. HPLC traces of (*R*)-**4a** (**Table 2, Entry 11**).

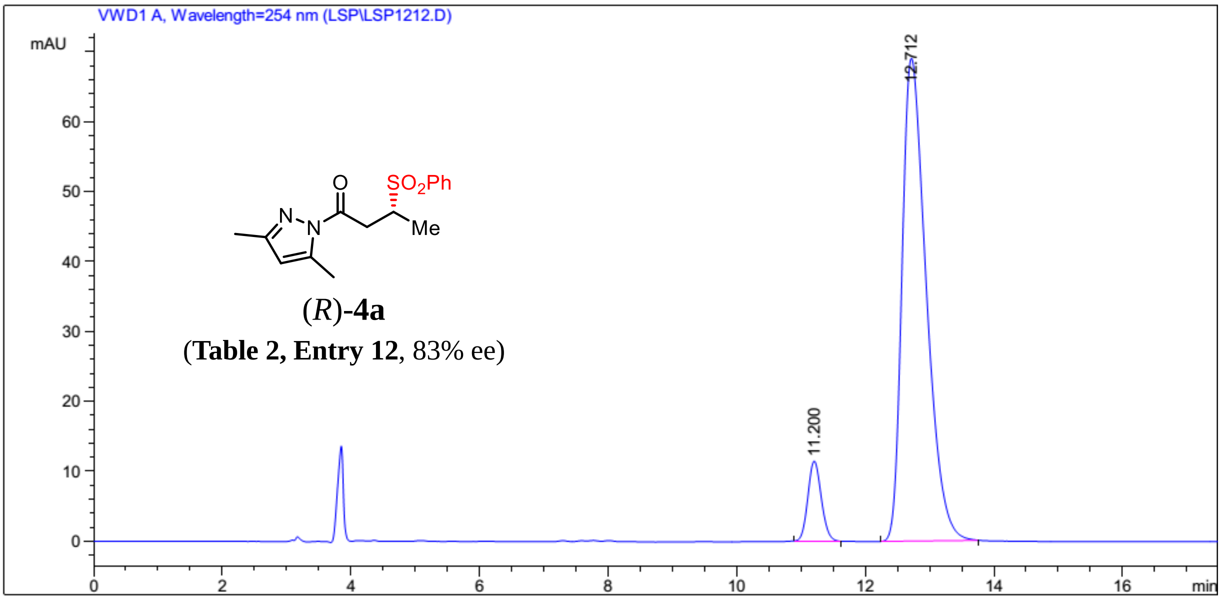

Peak #	RetTime [min]	Type	Width [min]	Area mAU *s	Height [mAU]	Area %
1	11.200	BB	0.2272	167.66081	11.48055	8.5030
2	12.712	BB	0.4009	1804.13269	69.06152	91.4970
Totals :				1971.79350	80.54206	

Figure S37. HPLC traces of (*R*)-**4a** (**Table 2, Entry 12**).

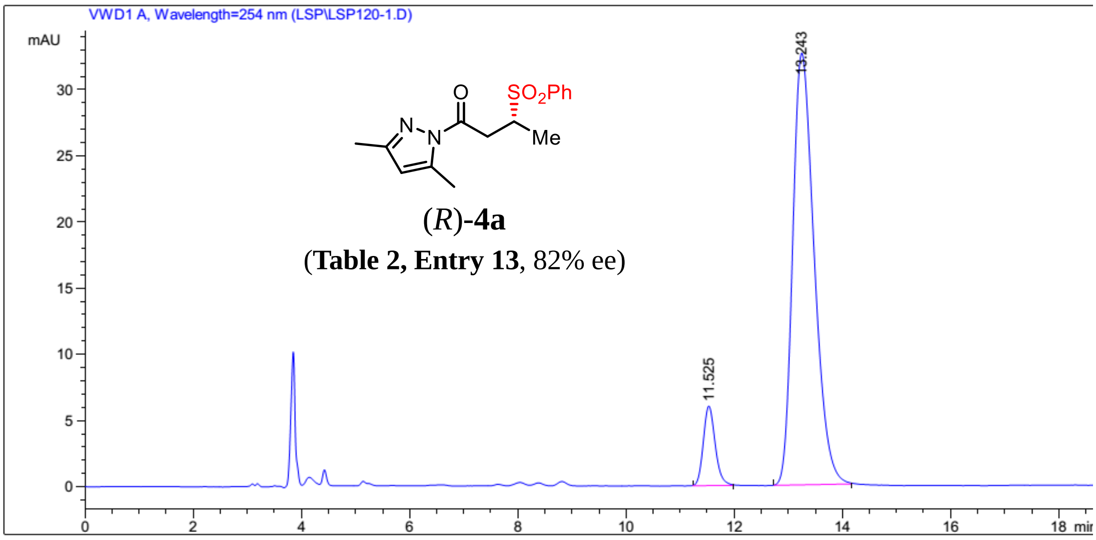

Peak #	RetTime [min]	Type	Width [min]	Area mAU *s	Height [mAU]	Area %
1	11.525	BB	0.2332	91.68983	6.01296	9.2498
2	13.243	BB	0.4278	899.57135	32.63813	90.7502
Totals :				991.26118	38.65109	

Figure S38. HPLC traces of (*R*)-**4a** (**Table 2, Entry 13**).

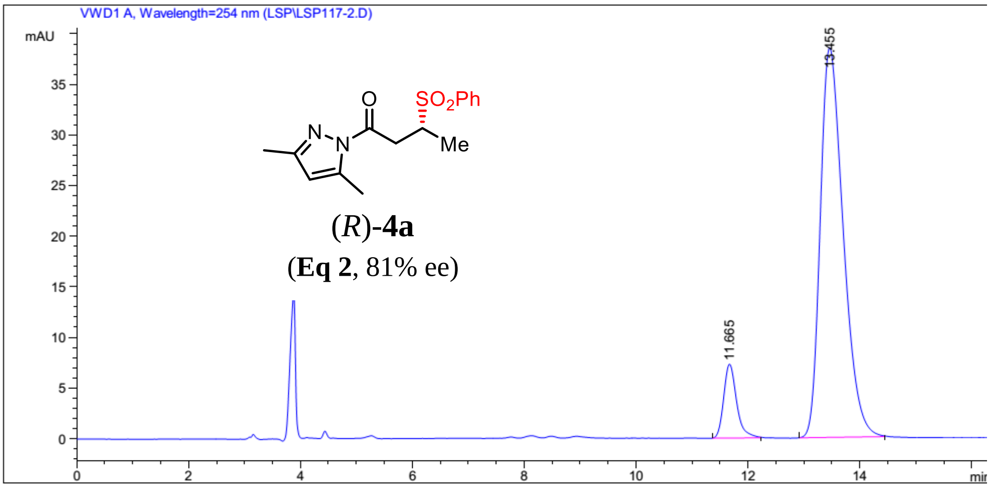

Signal 1: VWD1 A, Wavelength=254 nm

Peak #	RetTime [min]	Type	Width [min]	Area mAU *s	Height [mAU]	Area %
1	11.665	BB	0.2457	117.34642	7.30317	9.6634
2	13.455	BB	0.4386	1096.99194	38.51152	90.3366

Figure S39. HPLC traces of (*R*)-**4a** (**Eq 2**).

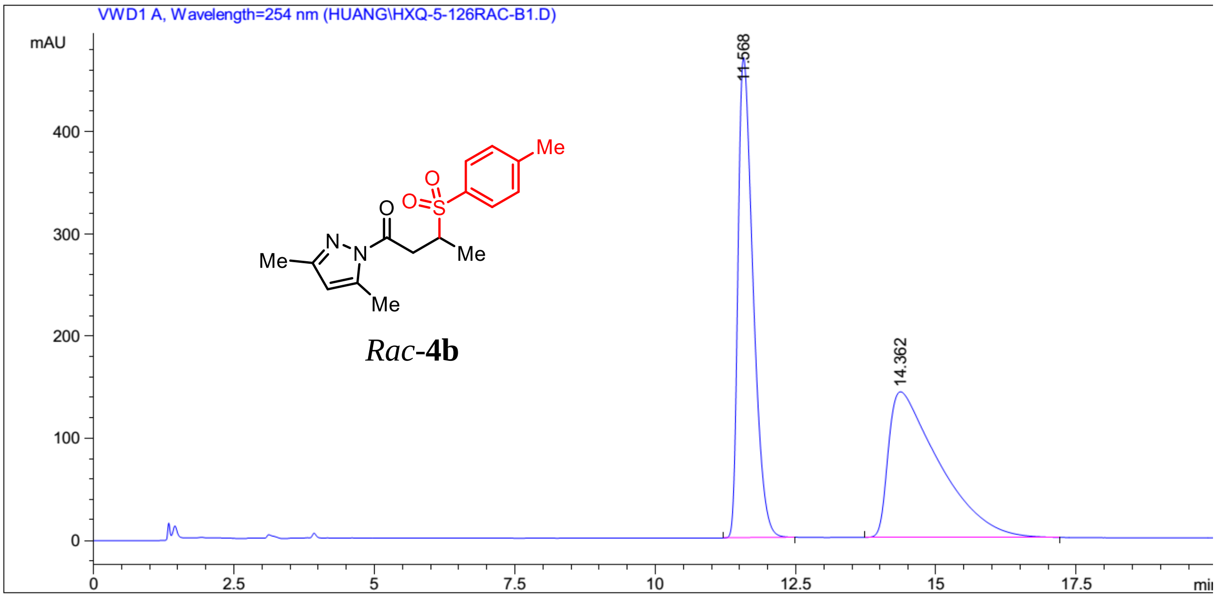

Peak #	RetTime [min]	Type	Width [min]	Area mAU *s	Height [mAU]	Area %
1	11.568	BB	0.2874	8762.41602	470.01019	49.4485
2	14.362	BB	0.8967	8957.88672	142.45035	50.5515
Totals :				1.77203e4	612.46054	

Figure S40. HPLC traces of *rac*-**4b**.

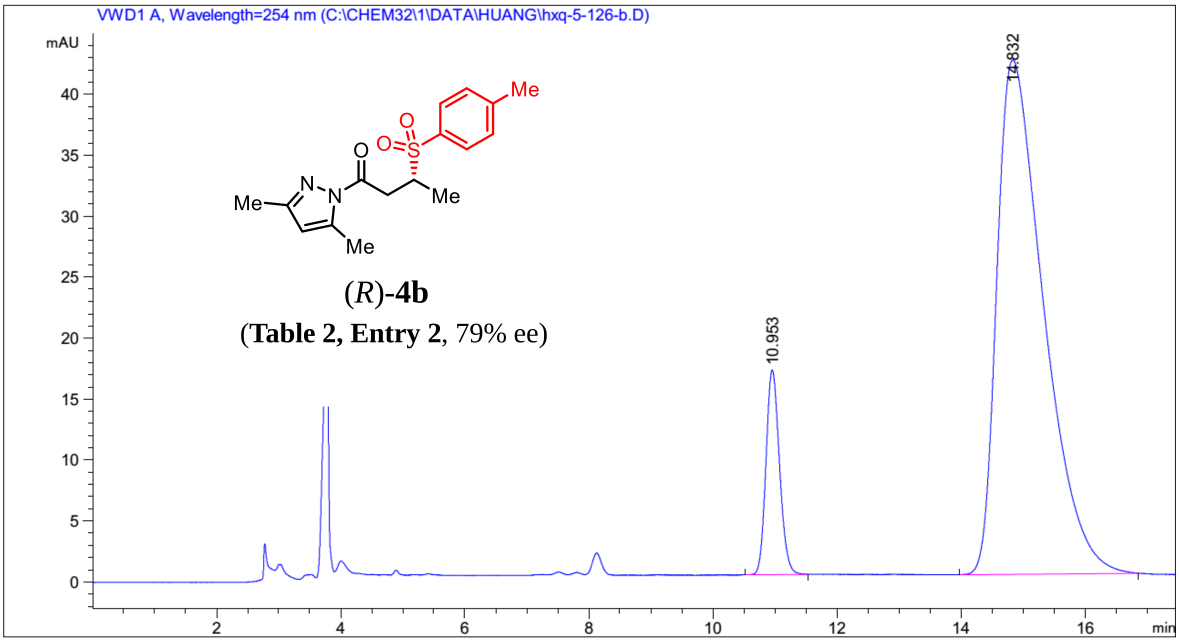

Peak #	RetTime [min]	Type	Width [min]	Area [mAU*s]	Height [mAU]	Area %
1	10.953	BB	0.1824	255.46745	16.80165	10.4017
2	14.832	VV R	0.6092	2200.55859	42.21118	89.5983

Figure S41. HPLC traces of (*R*)-**4b** (**Table 2, Entry 2**).

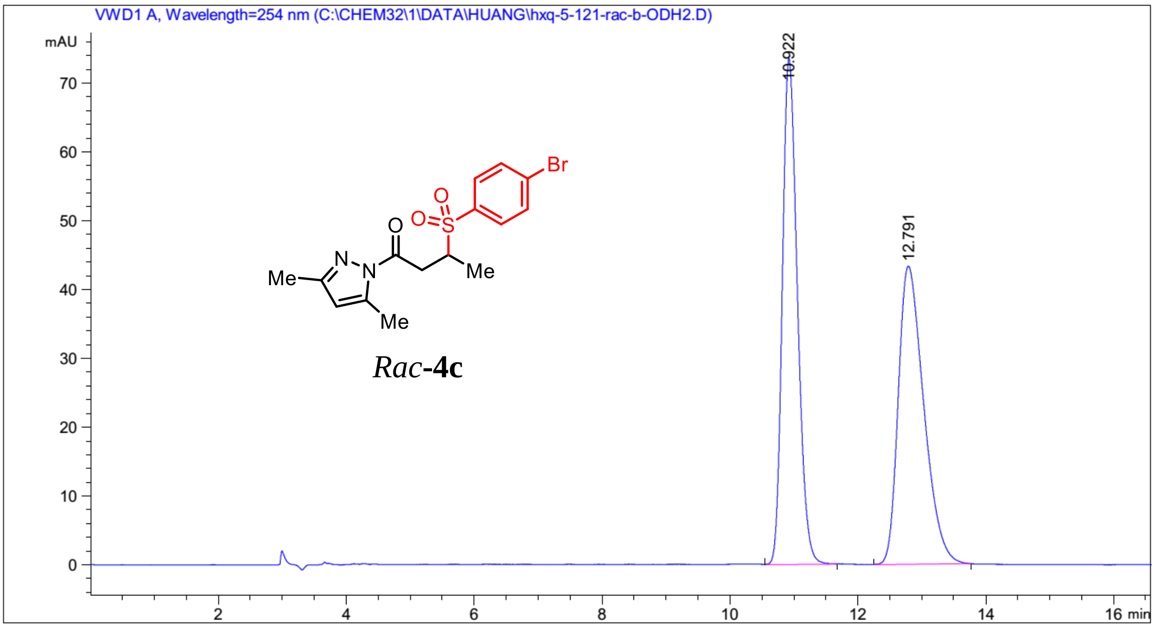

Peak #	RetTime [min]	Type	Width [min]	Area [mAU*s]	Height [mAU]	Area %
1	10.922	BV R	0.2449	1200.24792	73.52975	50.0197
2	12.791	BV R	0.3328	1199.30286	43.33937	49.9803

Figure S42. HPLC traces of *rac*-**4c**.

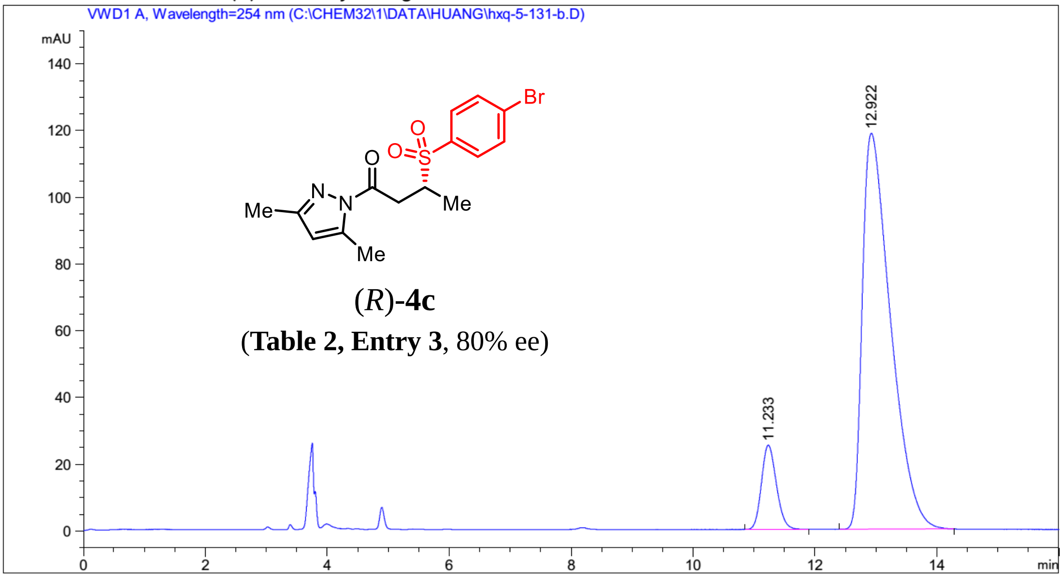

Peak #	RetTime [min]	Sig	Type	Area [mAU*s]	Height [mAU]	Area %
1	11.233	1	BB	429.83728	25.28220	10.1053
2	12.922	1	BB	3823.75635	118.75549	89.8947
Totals :				4253.59363	144.03769	

Figure S43. HPLC traces of (*R*)-**4c** (**Table 2, Entry 3**).

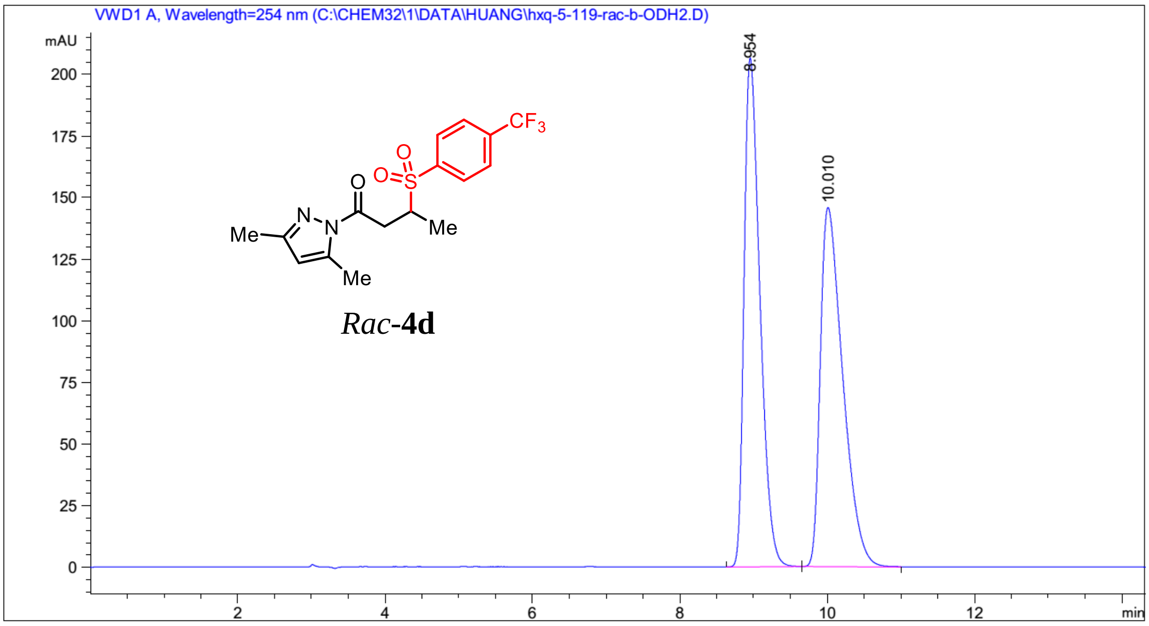

Peak #	RetTime [min]	Type	Width [min]	Area [mAU*s]	Height [mAU]	Area %
1	8.954	VB R	0.2269	3077.78955	206.44601	49.9498
2	10.010	BV R	0.3039	3083.97266	145.72467	50.0502
Totals :				6161.76221	352.17068	

Figure S44. HPLC traces of *rac*-**4d**.

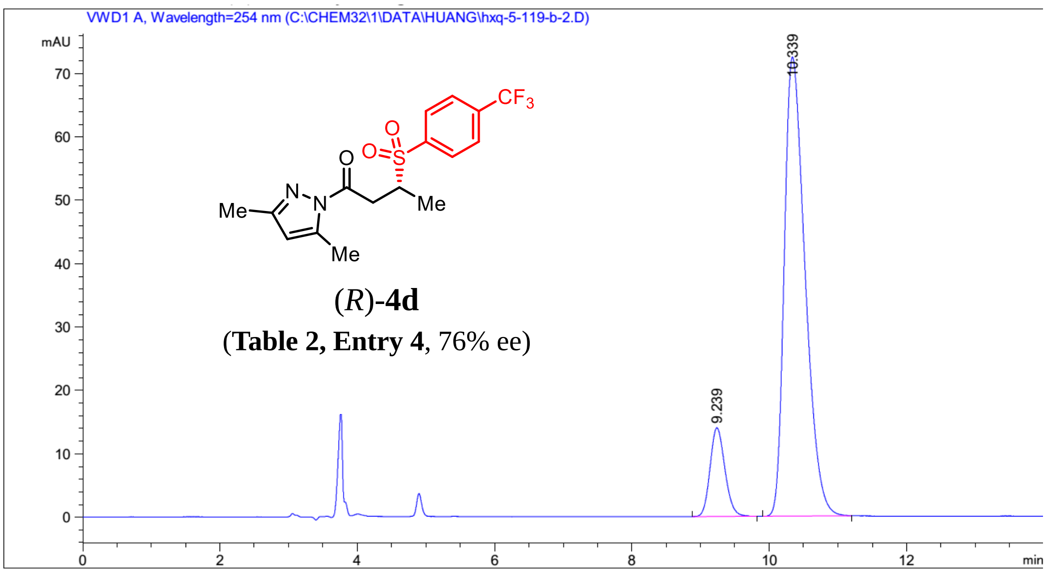

Peak #	RetTime [min]	Type	Width [min]	Area [mAU*s]	Height [mAU]	Area %
1	9.239	BB	0.1813	210.37302	14.00311	12.1529
2	10.339	BB	0.3004	1520.68164	72.45604	87.8471

Figure S45. HPLC traces of (*R*)-**4d** (**Table 2, Entry 4**).

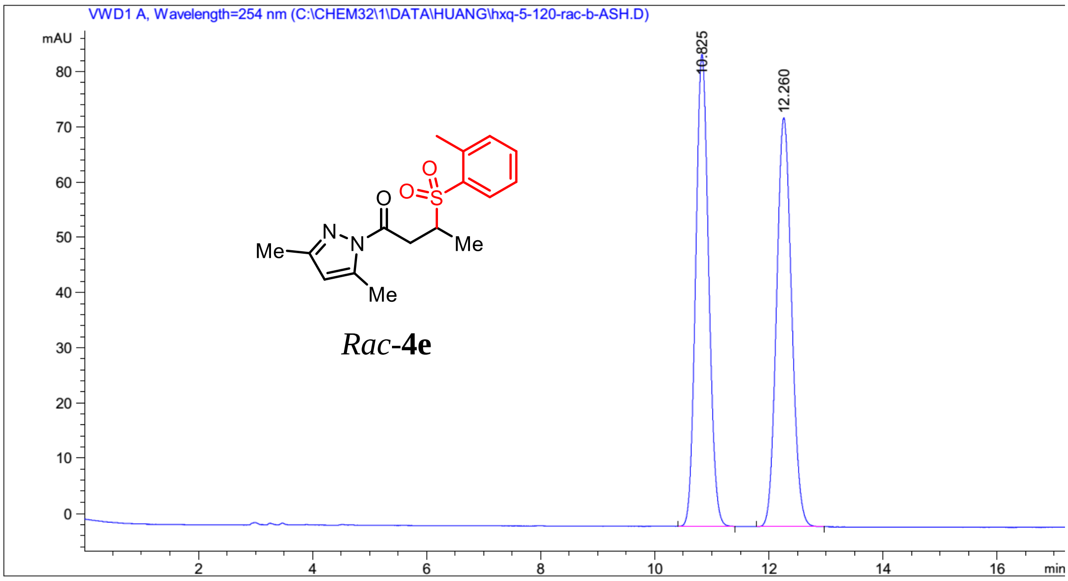

Peak #	RetTime [min]	Type	Width [min]	Area [mAU*s]	Height [mAU]	Area %
1	10.825	BB	0.2444	1351.39844	85.51785	49.8994
2	12.260	BV R	0.2697	1356.84717	74.07975	50.1006

Totals : 2708.24561 159.59760

Figure S46. HPLC traces of *rac*-**4e**.

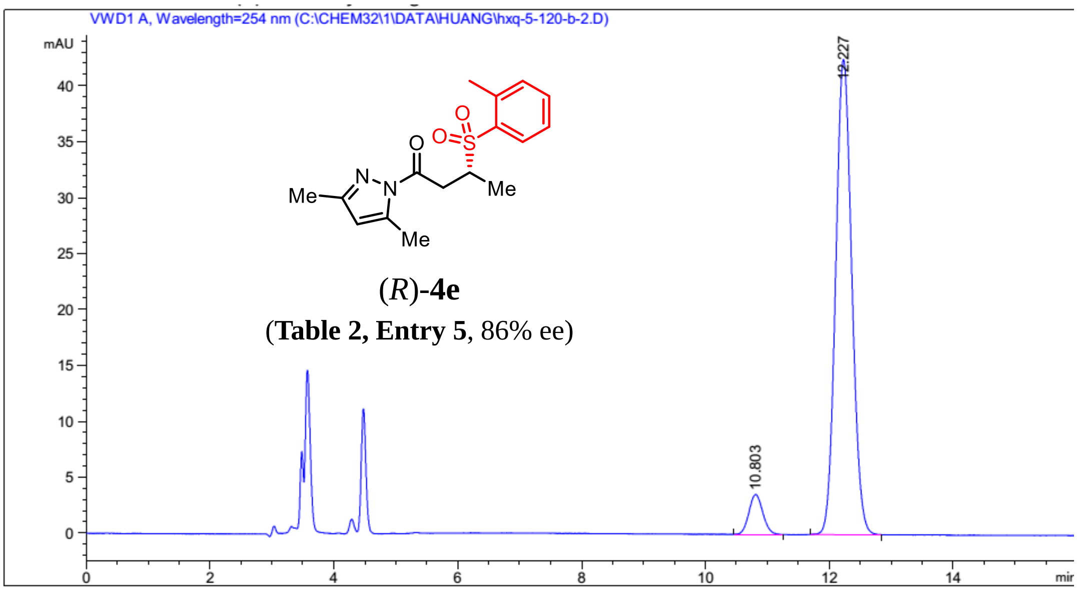

Peak #	RetTime [min]	Type	Width [min]	Area [mAU*s]	Height [mAU]	Area %
1	10.803	BB	0.1856	56.11126	3.58408	6.8033
2	12.227	BV R	0.2295	768.64886	42.49718	93.1967

Figure S47. HPLC traces of (*R*)-**4e** (**Table 2, Entry 5**).

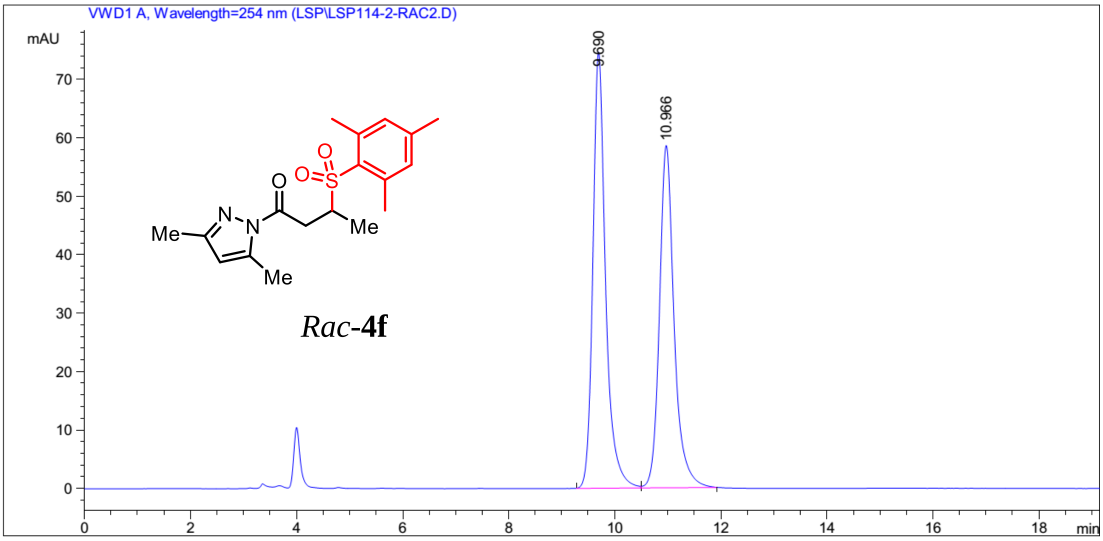

Peak #	RetTime [min]	Type	Width [min]	Area mAU *s	Height [mAU]	Area %
1	9.690	BV	0.2481	1242.96436	74.65981	52.3593
2	10.966	VB	0.2876	1130.94727	58.63719	47.6407
Totals :				2373.91162	133.29699	

Figure S48. HPLC traces of *rac*-**4f**.

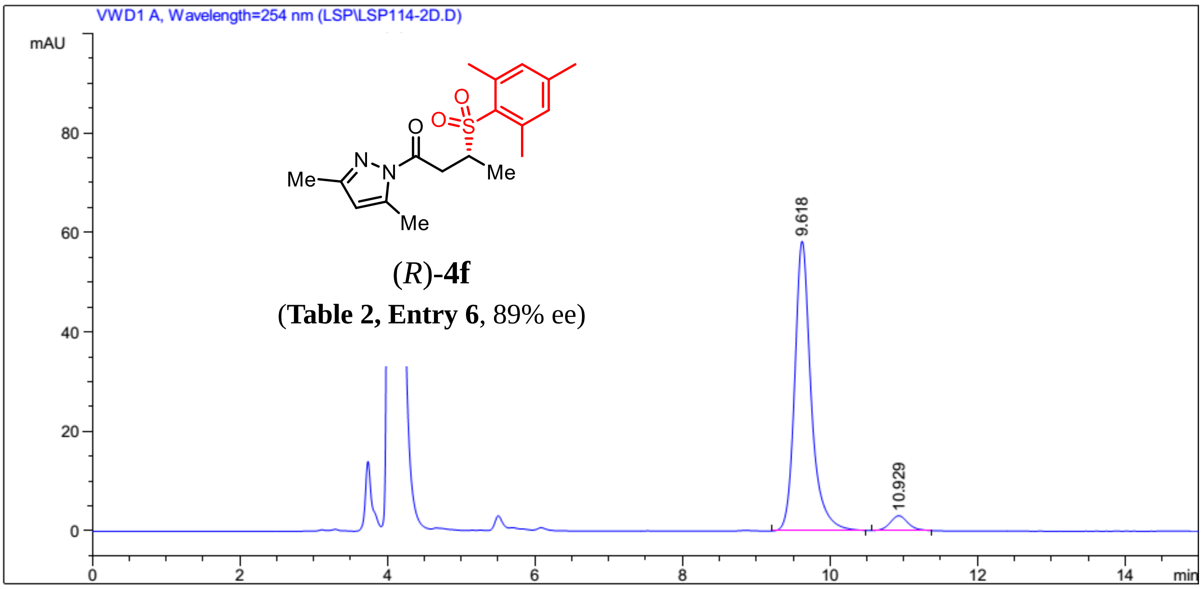

Peak #	RetTime [min]	Type	Width [min]	Area mAU *s	Height [mAU]	Area %
1	9.618	MM	0.2570	897.79773	58.22013	94.4645
2	10.930	MM	0.2910	52.60951	3.01309	5.5355
Totals :				950.40724	61.23322	

Figure S49. HPLC traces of (*R*)-**4f** (**Table 2, Entry 6**).

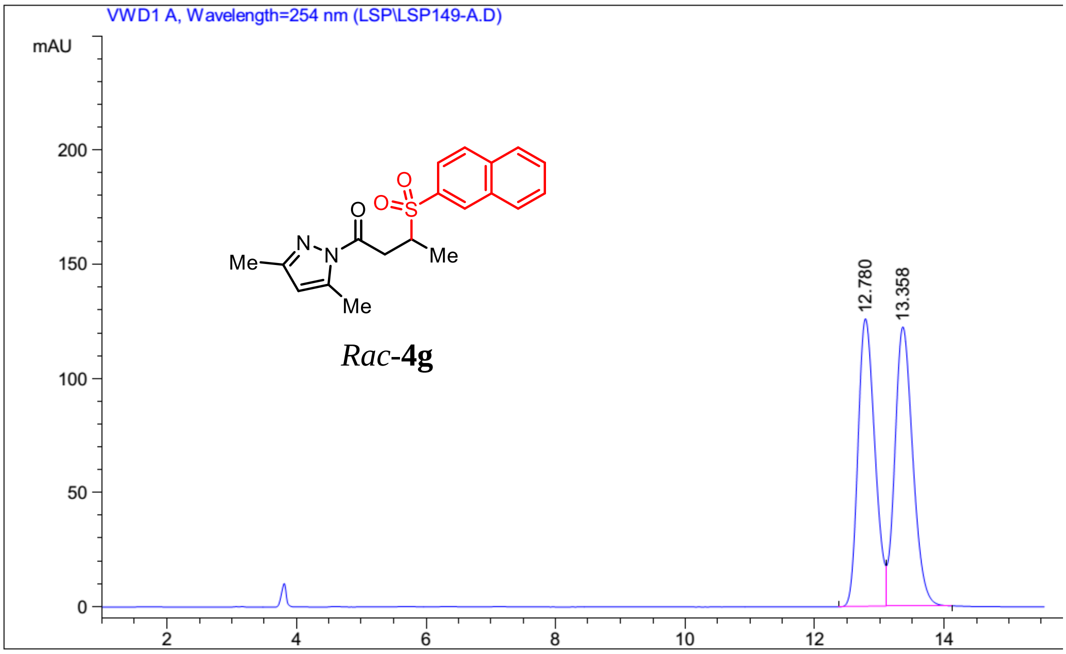

Peak #	RetTime [min]	Type	Width [min]	Area mAU *s	Height [mAU]	Area %
1	12.780	BV	0.2823	2279.17334	126.04043	49.0451
2	13.358	VB	0.2972	2367.92676	122.34003	50.9549
Totals :				4647.10010	248.38046	

Figure S50. HPLC traces of *rac*-**4g**.

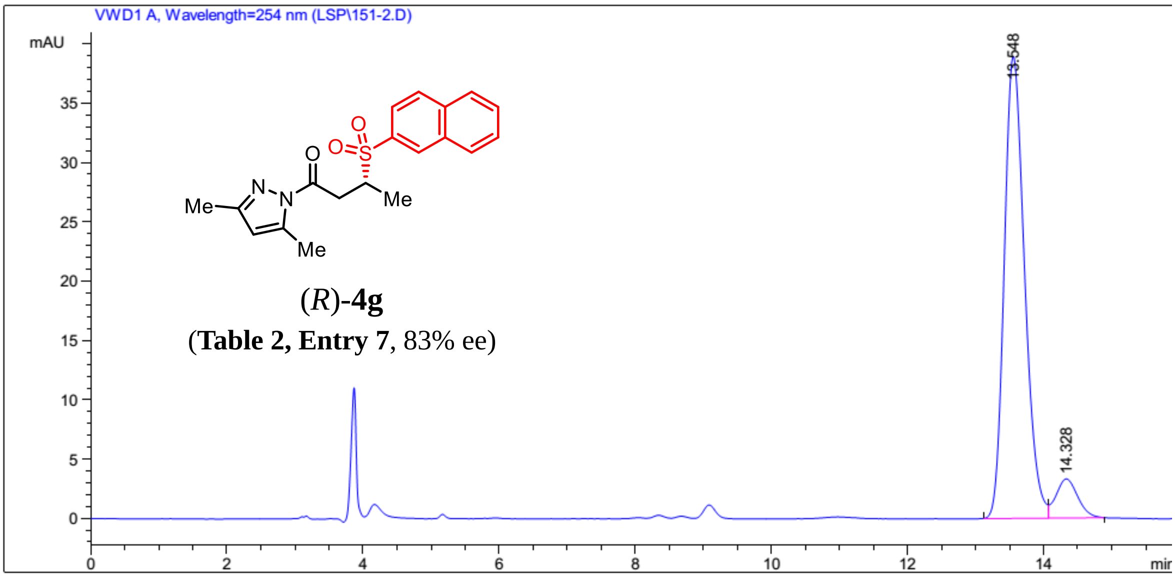

Peak #	RetTime [min]	Type	Width [min]	Area mAU *s	Height [mAU]	Area %
1	13.548	BV	0.3208	809.12994	38.98560	91.5429
2	14.328	VB	0.3396	74.75074	3.32264	8.4571
Totals :				883.88068	42.30824	

Figure S51. HPLC traces of (*R*)-**4g** (**Table 2, Entry 7**).

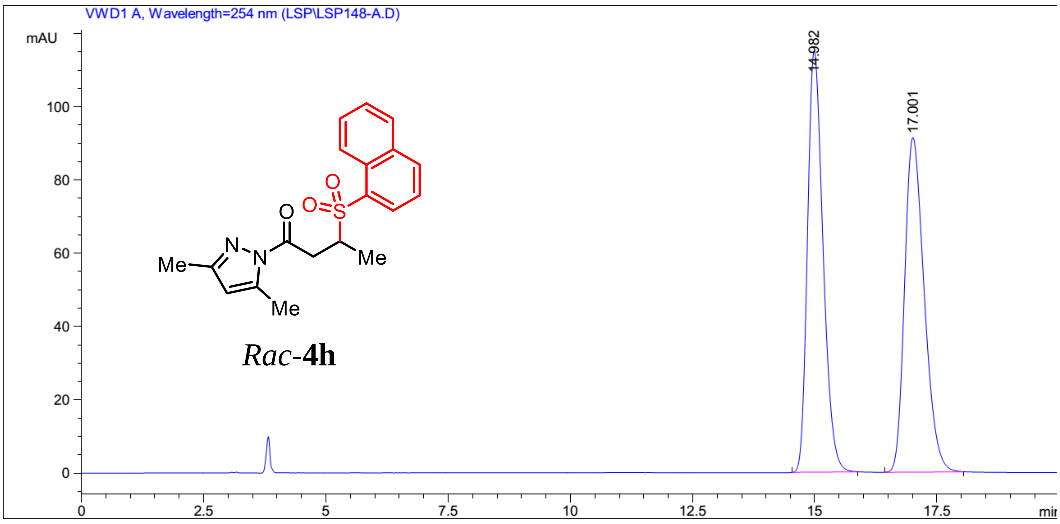

Peak #	RetTime [min]	Type	Width [min]	Area mAU *s	Height [mAU]	Area %
1	14.982	BB	0.3424	2545.13281	115.12033	49.9798
2	17.001	BB	0.4313	2547.18921	91.42813	50.0202
Totals :				5092.32202	206.54846	

Figure S52. HPLC traces of *rac*-**4h**.

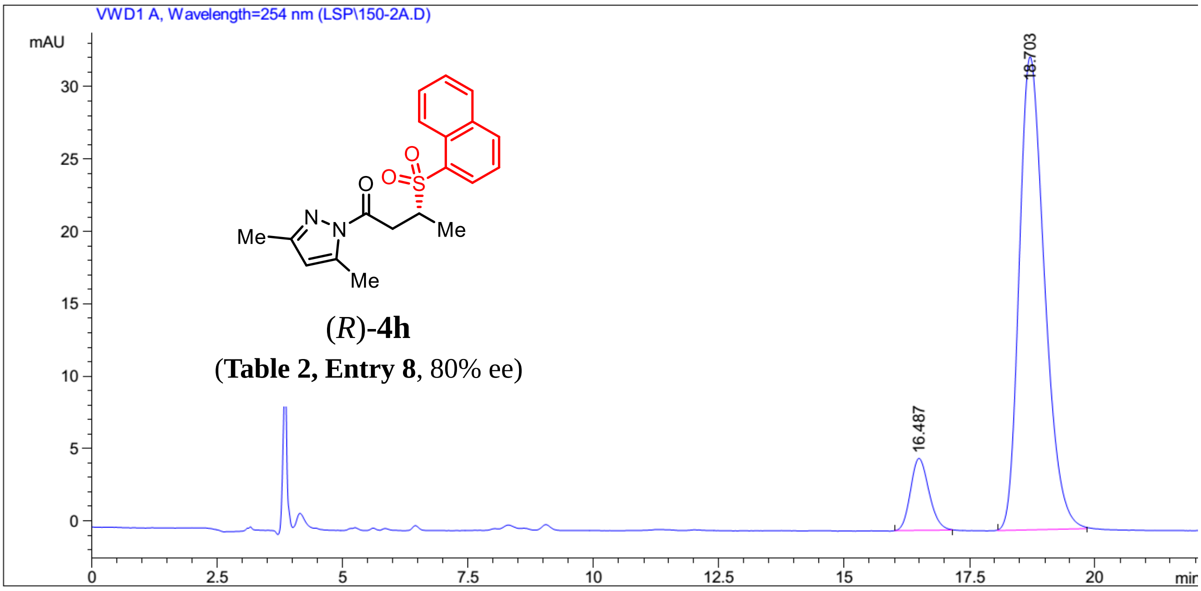

Peak #	RetTime [min]	Type	Width [min]	Area mAU *s	Height [mAU]	Area %
1	16.487	BB	0.3970	128.87476	4.99753	10.1104
2	18.703	BB	0.5383	1145.80627	32.70365	89.8896
Totals :				1274.68103	37.70118	

Figure S53. HPLC traces of (*R*)-**4h** (**Table 2, Entry 8**).

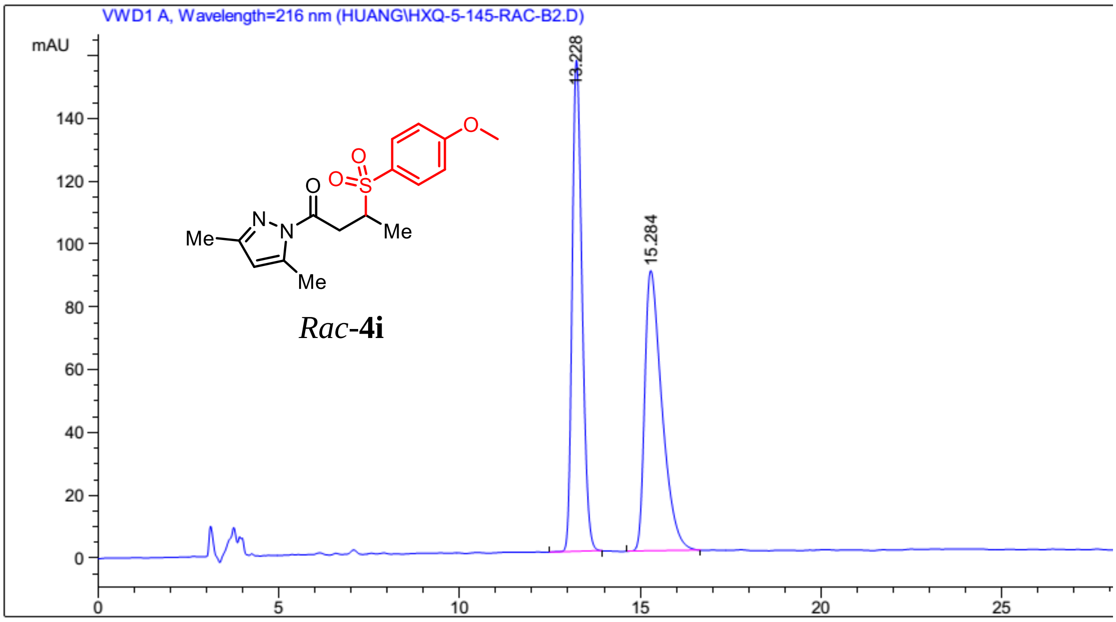

Peak #	RetTime [min]	Type	Width [min]	Area mAU *s	Height [mAU]	Area %
1	13.228	BB	0.2937	2962.84375	156.46800	49.9486
2	15.284	BB	0.5000	2968.93872	89.30674	50.0514
Totals :				5931.78247	245.77474	

Figure S54. HPLC traces of *rac*-**4i.**

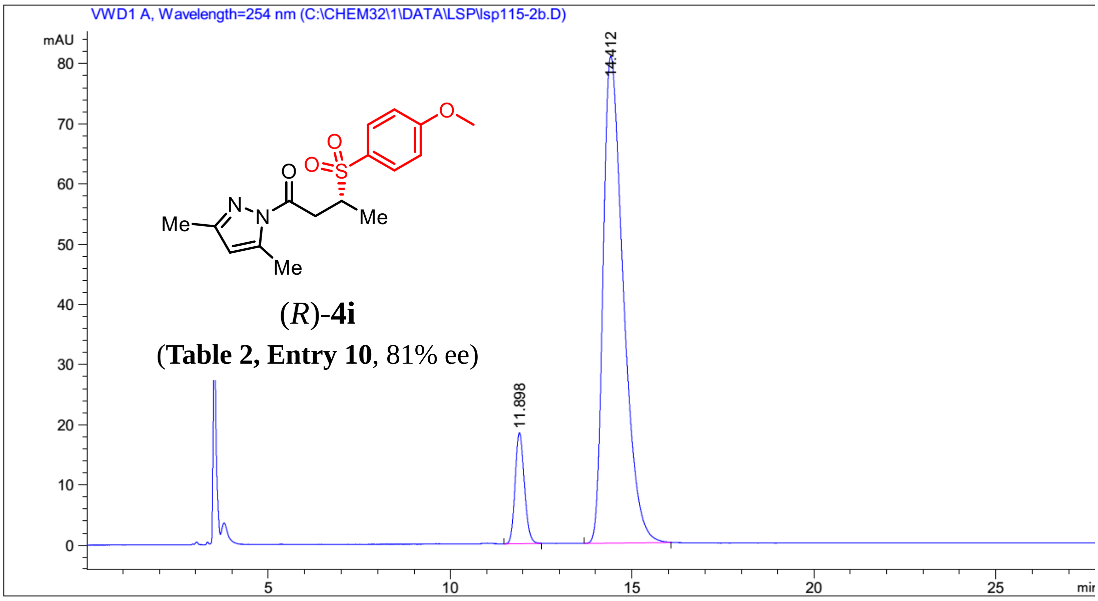

Peak #	RetTime [min]	Type	Width [min]	Area [mAU*s]	Height [mAU]	Area %
1	11.898	VV R	0.2090	326.41470	18.43900	9.4860
2	14.412	VV R	0.4617	3114.59399	80.98930	90.5140

Figure S55. HPLC traces of (*R*)-**4i** (**Table 2, Entry 10**).

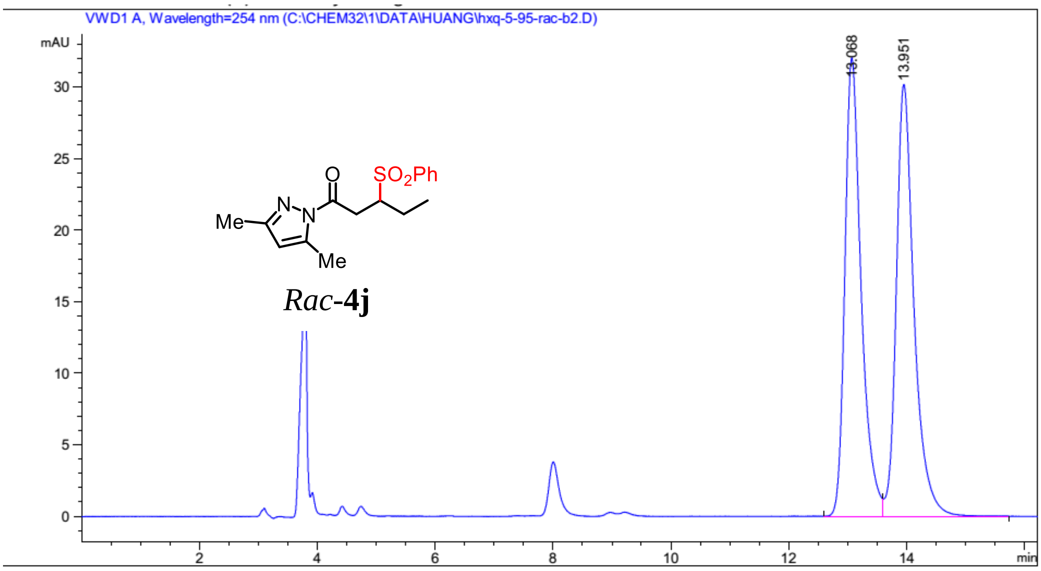

Peak #	RetTime [min]	Sig	Type	Area [mAU*s]	Height [mAU]	Area %
1	13.068	1	BV	616.88562	32.05754	49.4134
2	13.951	1	VB	631.53168	30.19418	50.5866
Totals :				1248.41730	62.25172	

Figure S56. HPLC traces of *rac*-**4j**.

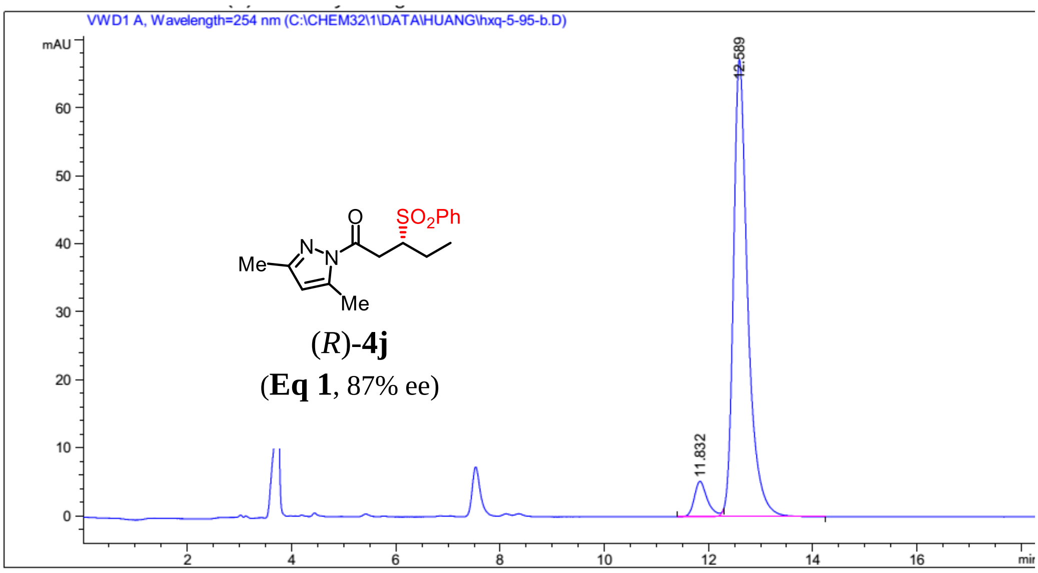

Peak #	RetTime [min]	Sig	Type	Area [mAU*s]	Height [mAU]	Area %
1	11.832	1	BV E	90.15114	5.20055	6.5721
2	12.589	1	VB R	1281.57886	67.19083	93.4279
Totals :				1371.73000	72.39138	

Figure S57. HPLC traces of (*R*)-**4j** (**Eq 1**).

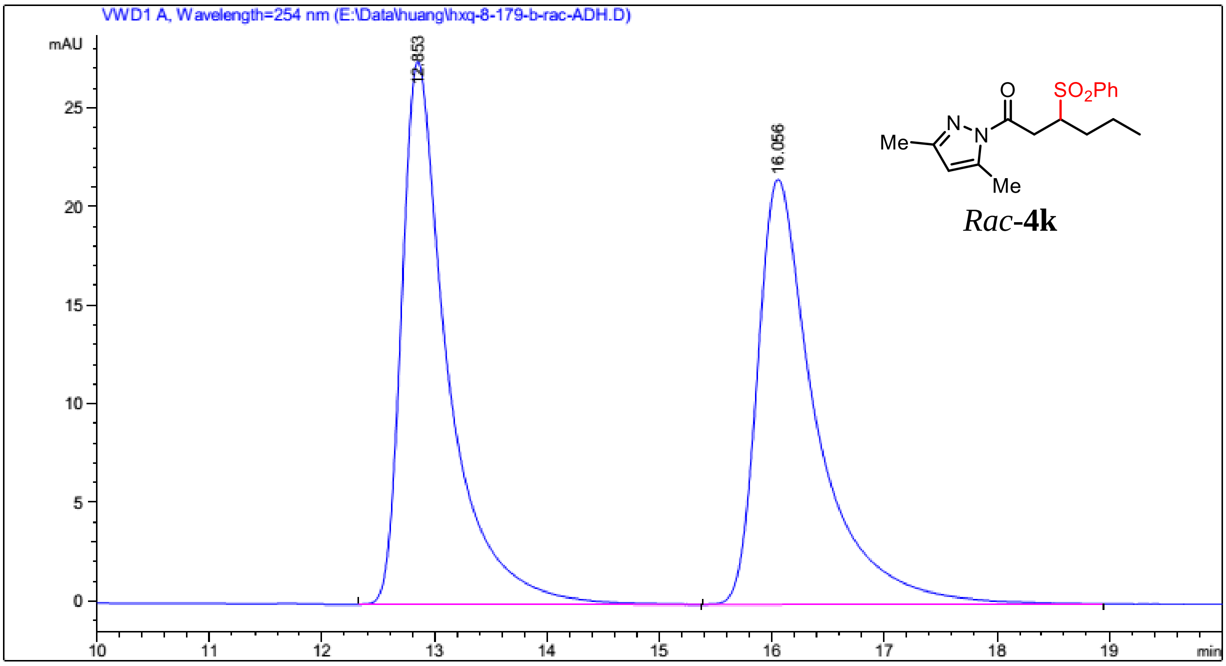

Peak #	RetTime [min]	Type	Width [min]	Area [mAU*s]	Height [mAU]	Area %
1	12.853	BB	0.4152	786.22095	27.53284	50.1074
2	16.056	BB	0.5255	782.84998	21.56517	49.8926

Figure S58. HPLC traces of *rac*-**4k**.

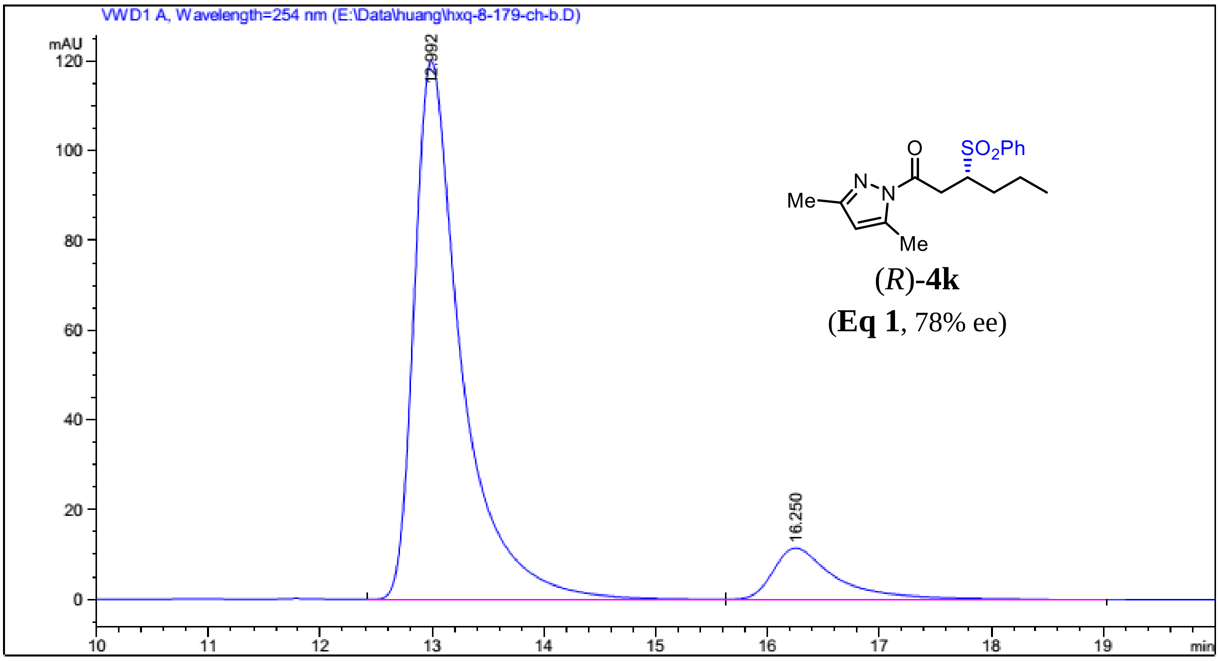

Peak #	RetTime [min]	Type	Width [min]	Area [mAU*s]	Height [mAU]	Area %
1	12.992	BB	0.4322	3537.34741	119.90150	88.8365
2	16.250	BB	0.5648	444.51563	11.40248	11.1635

Figure S59. HPLC traces of (*R*)-**4k** (**Eq 1**).

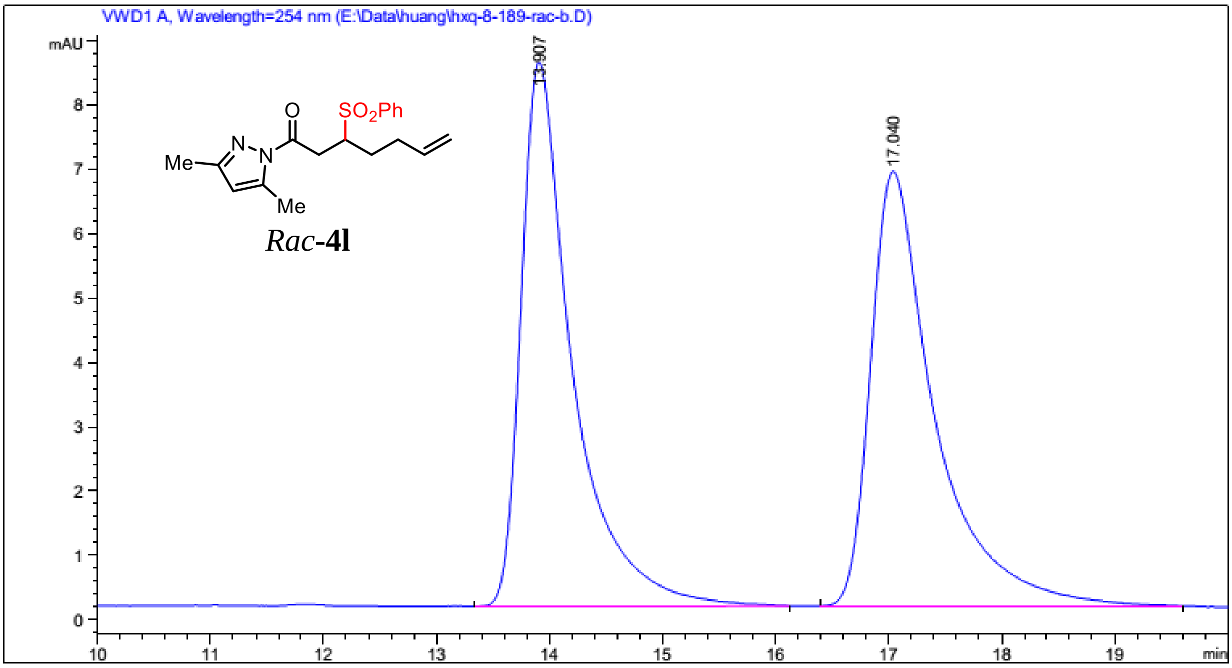

Peak #	RetTime [min]	Type	Width [min]	Area [mAU*s]	Height [mAU]	Area %
1	13.907	BB	0.4614	268.51575	8.45767	50.1923
2	17.040	BB	0.5746	266.45828	6.74868	49.8077

Figure S60. HPLC traces of *rac*-**4l**.

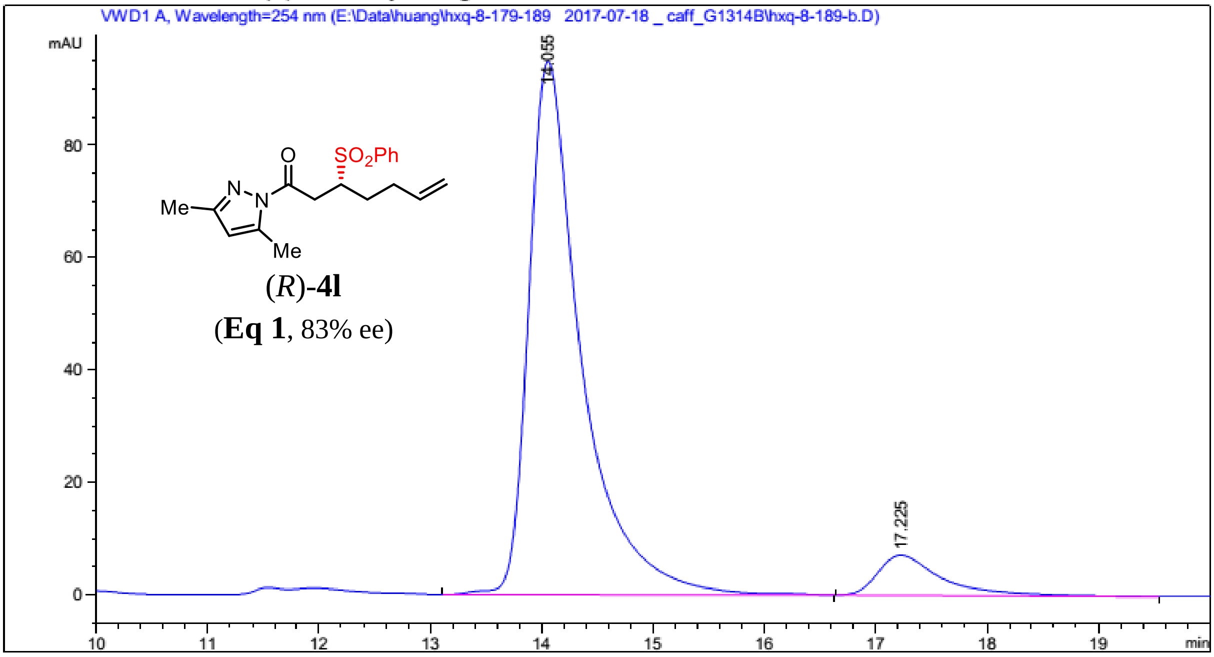

Peak #	RetTime [min]	Type	Width [min]	Area [mAU*s]	Height [mAU]	Area %
1	14.055	BB	0.4817	3132.79468	95.00914	91.6515
2	17.225	BB	0.5825	285.36353	7.18316	8.3485

Figure S61. HPLC traces of (*R*)-**4l** (**Eq 1**).

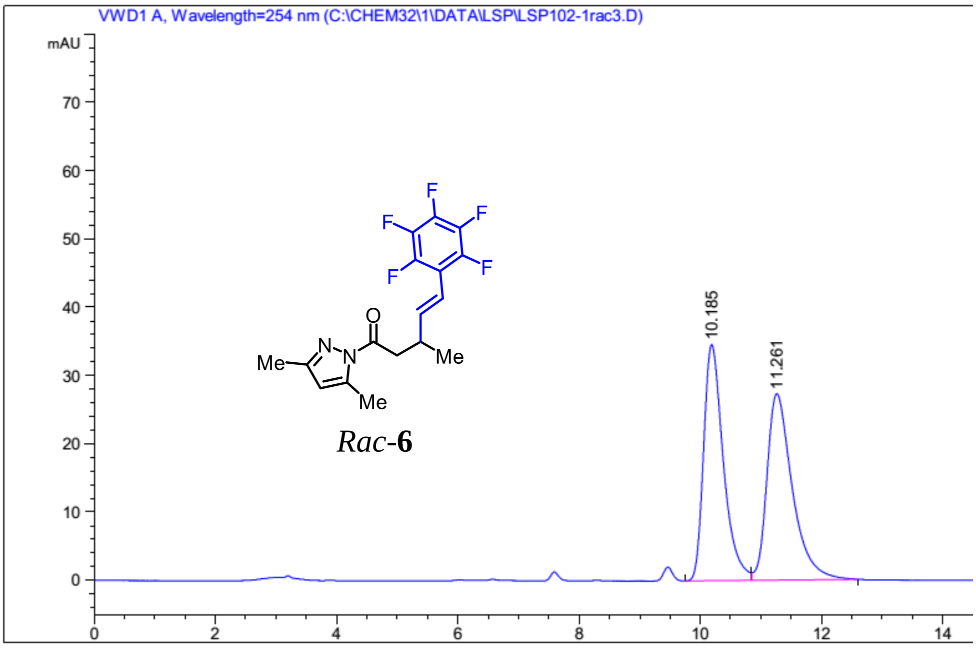

Peak #	RetTime [min]	Type	Width [min]	Area [mAU*s]	Height [mAU]	Area %
1	10.185	BV	0.3123	782.00269	34.58651	49.5600
2	11.261	VV R	0.3480	795.88818	27.30594	50.4400

Figure S62. HPLC traces of *rac*-**6**.

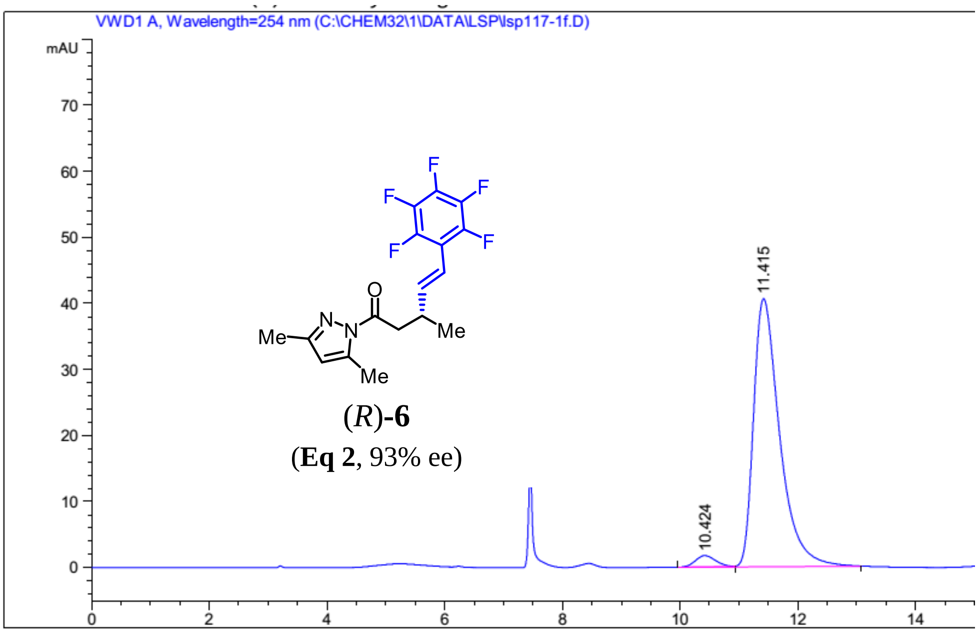

Peak #	RetTime [min]	Type	Width [min]	Area [mAU*s]	Height [mAU]	Area %
1	10.424	BV E	0.2891	42.15159	1.73378	3.2596
2	11.415	VB R	0.3785	1250.98792	40.59832	96.7404

Figure S63. HPLC traces of (*R*)-**6** (**Eq 2**).

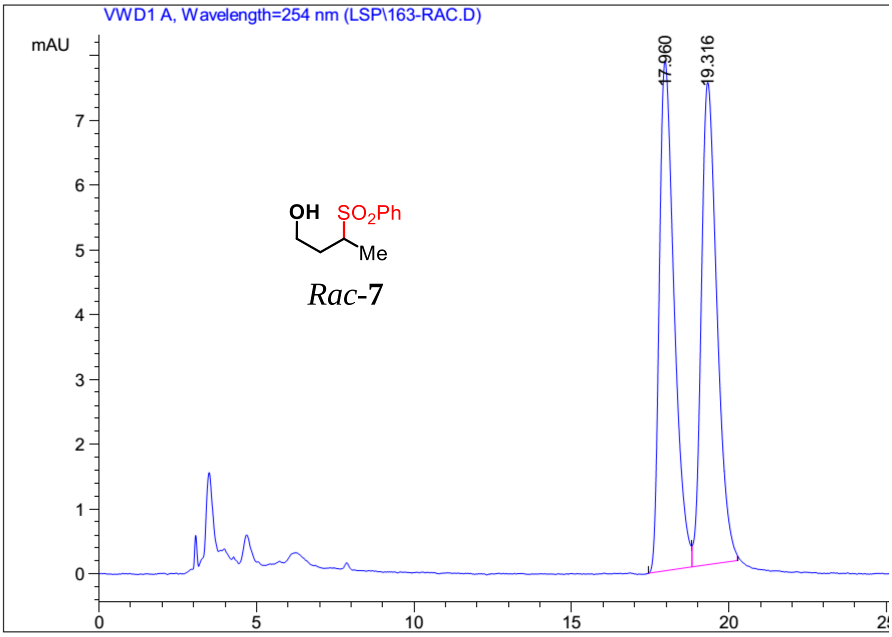

Peak #	RetTime [min]	Type	Width [min]	Area mAU *s	Height [mAU]	Area %
1	17.960	BB	0.5034	258.47421	7.88221	49.9812
2	19.316	BB	0.5286	258.66904	7.45366	50.0188
Totals :				517.14325	15.33587	

Figure S64. HPLC traces of *rac*-**7**.

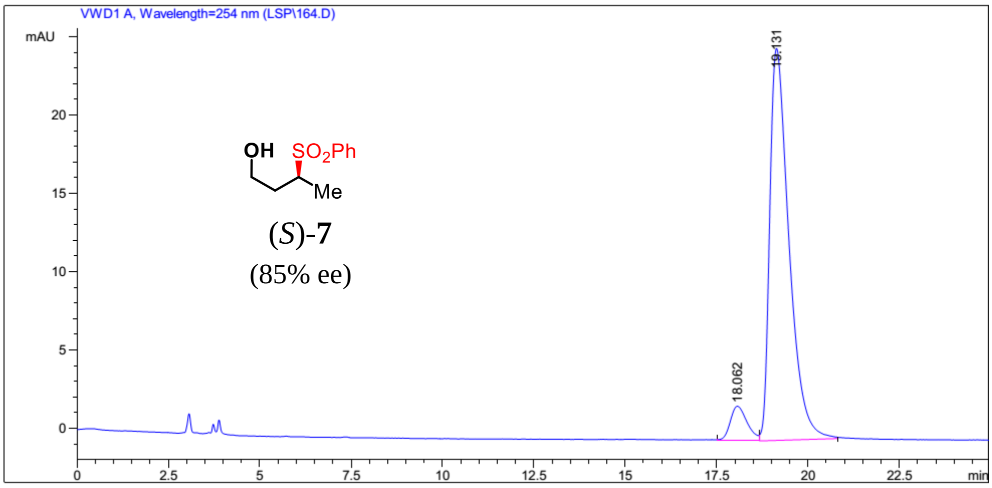

Peak #	RetTime [min]	Type	Width [min]	Area mAU *s	Height [mAU]	Area %
1	18.062	MM R	0.5439	72.59333	2.22464	7.3469
2	19.131	MM R	0.6100	915.48694	25.01384	92.6531
Totals :				988.08027	27.23848	

Figure S65. HPLC traces of (*S*)-**7**.

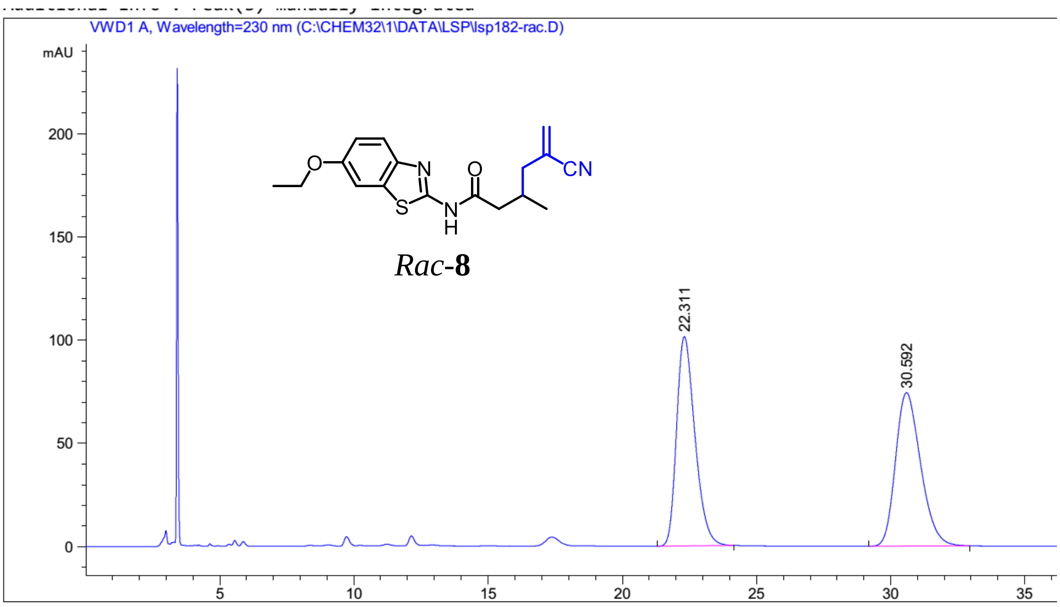

Peak #	RetTime [min]	Type	Width [min]	Area [mAU*s]	Height [mAU]	Area %
1	22.311	BB	0.5754	4817.23096	101.37652	49.9299
2	30.592	BB	0.7604	4830.76709	74.26881	50.0701

Figure S66. HPLC traces of *rac*-**8**.

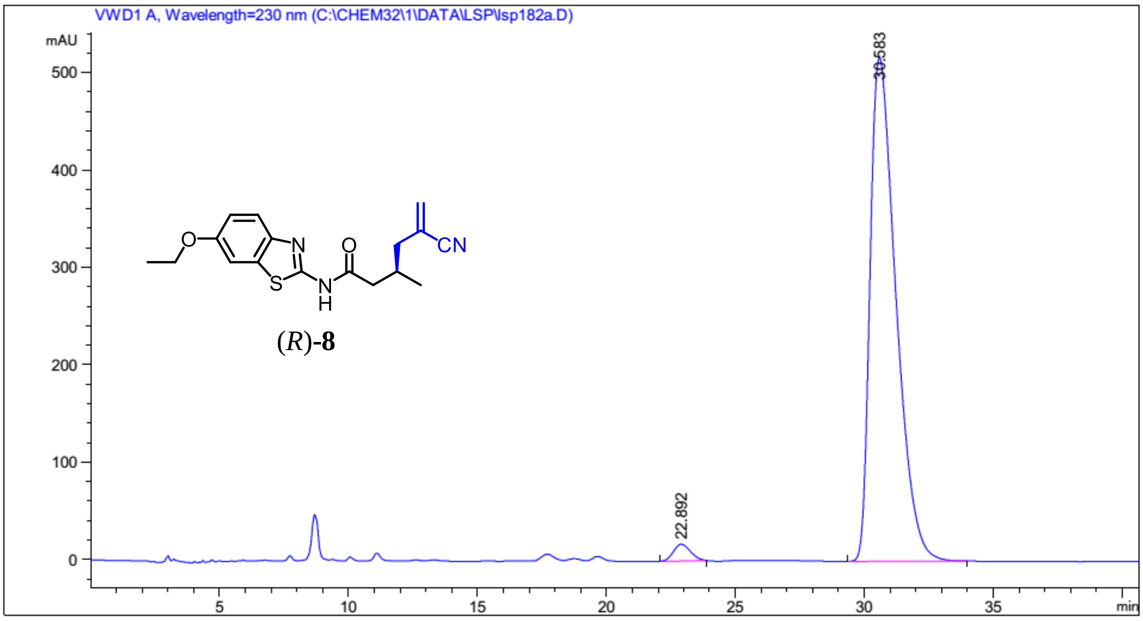

Peak #	RetTime [min]	Type	Width [min]	Area [mAU*s]	Height [mAU]	Area %
1	22.892	MM	0.7685	793.06097	17.19859	2.1626
2	30.583	BB	0.9633	3.58780e4	517.21069	97.8374

Figure S67. HPLC traces of (*R*)-**8**.

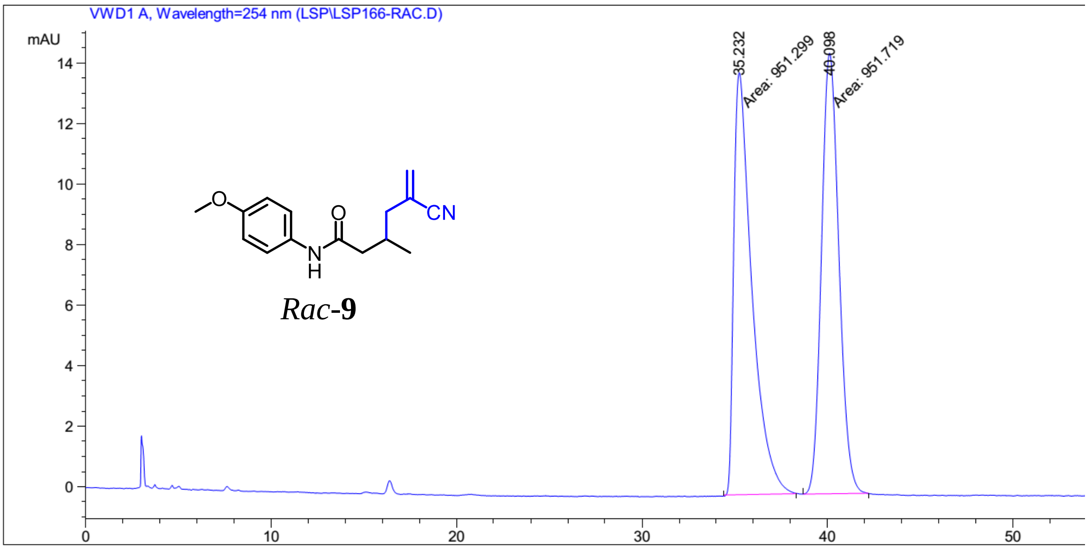

Peak #	RetTime [min]	Type	Width [min]	Area mAU *s	Height [mAU]	Area %
1	35.232	MM	1.1360	951.29907	13.95656	49.9890
2	40.098	MM	1.0885	951.71906	14.57227	50.0110
Totals :				1903.01813	28.52882	

Figure S68**.** HPLC traces of *rac*-**9**.

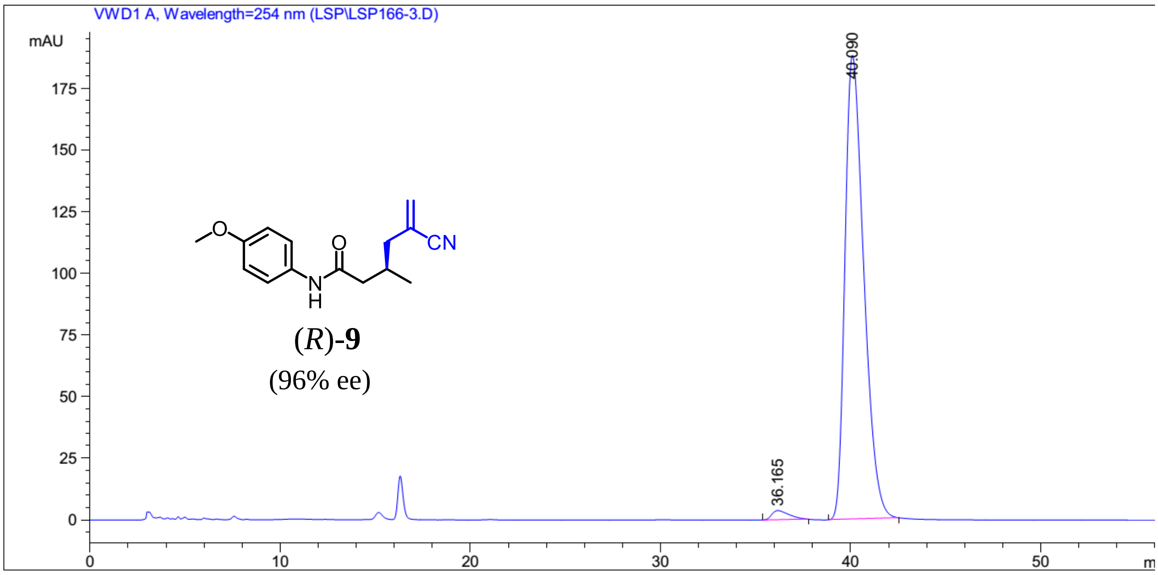

Peak #	RetTime [min]	Type	Width [min]	Area mAU *s	Height [mAU]	Area %
1	36.165	BB	0.7971	241.82167	3.75910	1.8344
2	40.090	BB	1.0500	1.29405e4	188.14247	98.1656
Totals :				1.31823e4	191.90158	

Figure S69**.** HPLC traces of (*R*)-**9**.

10. HPLC Traces of the Robustness Screen

Table S5. Reaction compatibility in the present of additives[a]

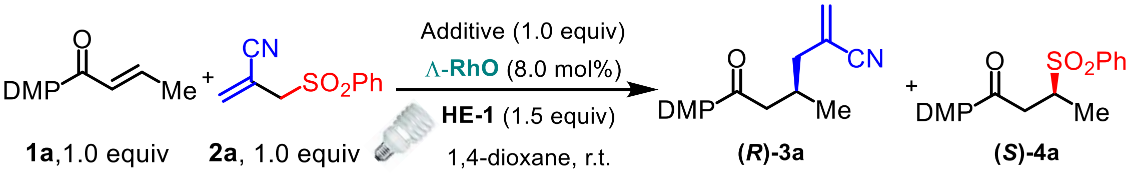

		Additive	(*R*)-3a		(*S*)-4a	
Entry	Additive	Recovered[b]	Yield[b]	ee[c]	Yield[b]	ee[c]
1	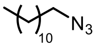	88%	86%	96% ee	85%	86% ee
2	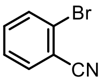	94%	86%	96% ee	85%	83% ee
3	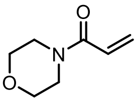	99%	86%	96% ee	92%	83% ee
4	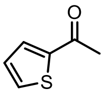	88%	82%	96% ee	88%	83% ee
5	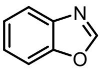	95%	86%	95% ee	92%	85% ee
6	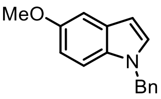	99%	86%	96% ee	85%	83% ee
7	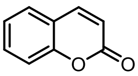	96%	82%	96% ee	92%	85% ee
8	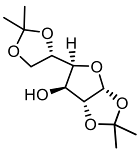	99%	82%	95% ee	92%	83% ee
9	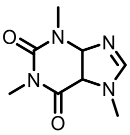	99%	86%	96% ee	92%	85% ee
10	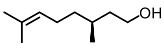	94%	86%	96% ee	92%	85% ee
11	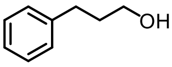	92%	86%	96% ee	89%	83% ee

12	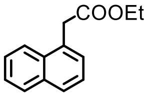	95%	85%	95% ee	90%	85% ee
13	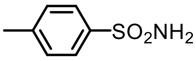	99%	86%	95% ee	92%	83% ee
14	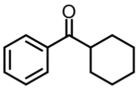	95%	86%	95% ee	88%	82% ee
15	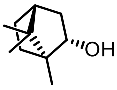	90%	86%	95% ee	92%	83% ee
16	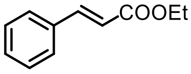	94%	82%	96% ee	88%	83% ee

[a] Reaction conditions: **1a** (0.20 mmol), **2a** (0.10 mmol), Δ-**RhO** (8.0 mol%), **HE-1** (0.15 mmol) and additives (0.1 mmol) in 1,4-dioxane (1.0 mL, 0.1 M) were stirred at room temperature for 24 h irradiated with 21 W CFL. [b] Isolated yield. [c] Determined by HPLC on a chiral stationary phase.

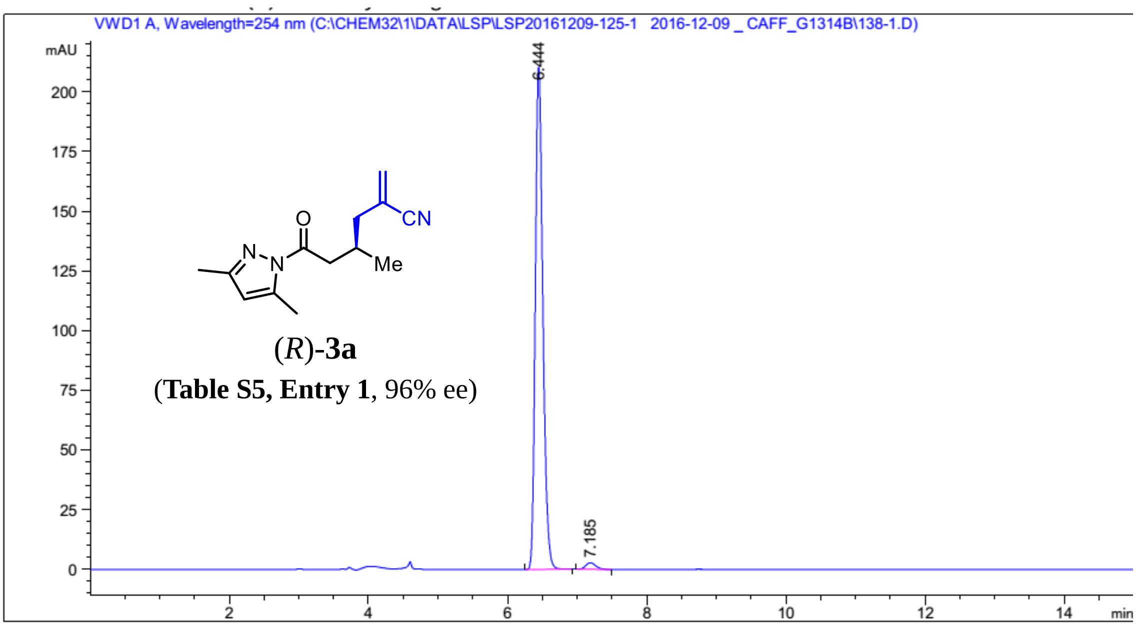

Peak #	RetTime [min]	Type	Width [min]	Area [mAU*s]	Height [mAU]	Area %
1	6.444	BB	0.1163	1583.33728	210.41260	98.2843
2	7.185	BB	0.1178	27.63959	2.81002	1.7157
Totals :				1610.97687	213.22262	

Figure S70. HPLC traces of (*R*)-**3a** (**Table S5, Entry 1**).

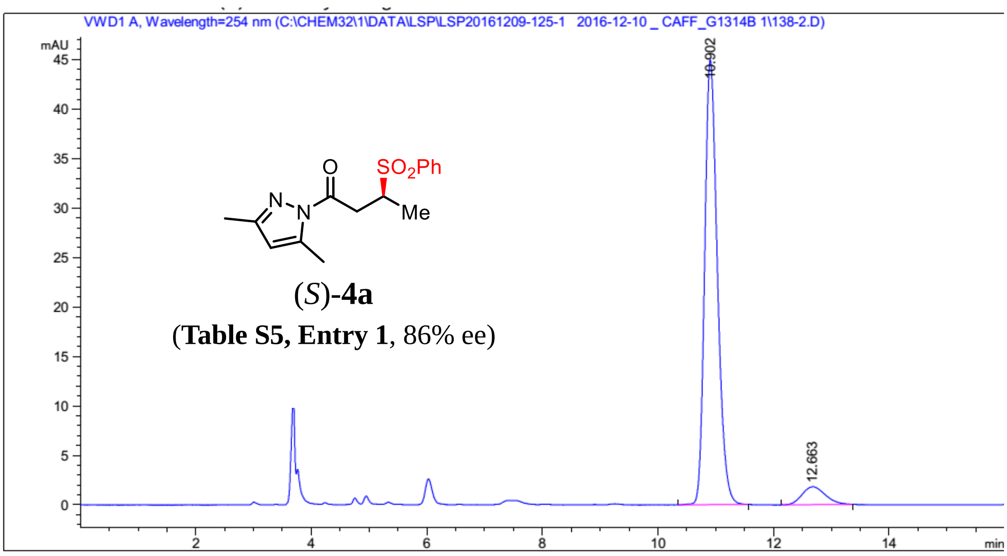

Peak #	RetTime [min]	Type	Width [min]	Area [mAU*s]	Height [mAU]	Area %
1	10.902	BB	0.2284	687.39563	44.96198	93.0383
2	12.663	BB	0.3322	51.43543	1.82034	6.9617
Totals :				738.83106	46.78232	

Figure S71. HPLC traces of (*S*)-**3a** (**Table S5, Entry 1**).

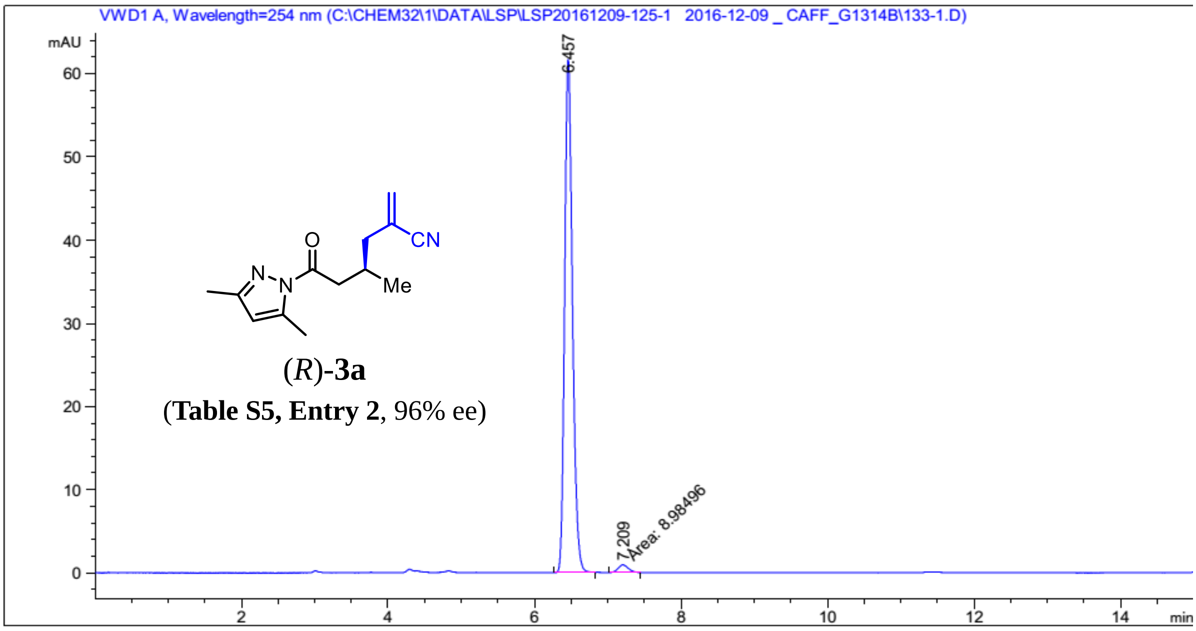

Peak #	RetTime [min]	Type	Width [min]	Area [mAU*s]	Height [mAU]	Area %
1	6.457	BB	0.1143	459.03085	61.71605	98.0802
2	7.209	MM	0.1638	8.98496	9.14476e-1	1.9198
Totals :				468.01582	62.63052	

Figure S72. HPLC traces of (*R*)-**3a** (**Table S5, Entry 2**).

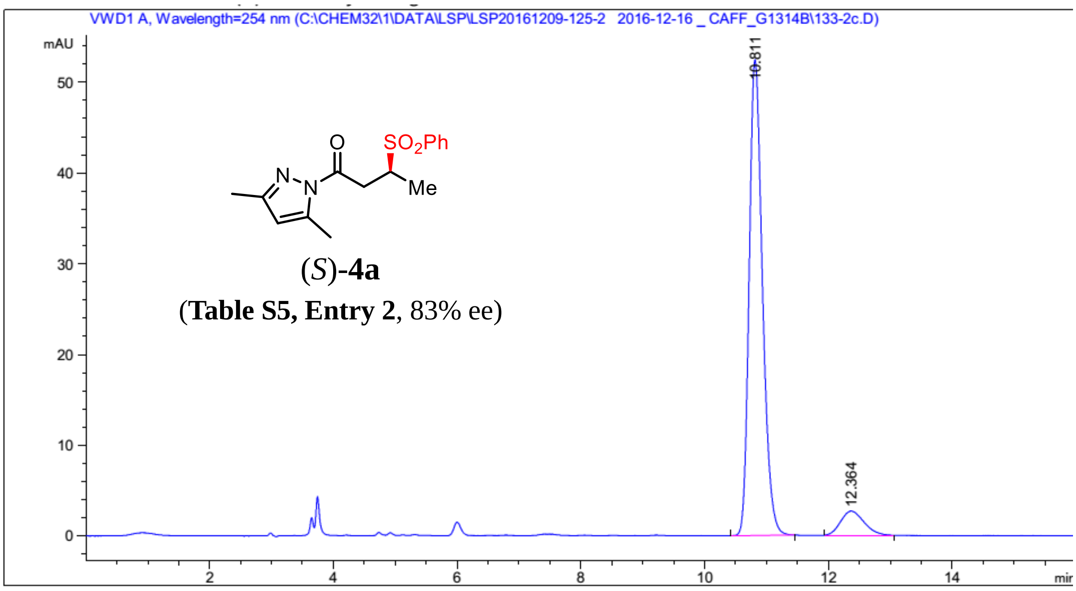

Peak #	RetTime [min]	Type	Width [min]	Area [mAU*s]	Height [mAU]	Area %
1	10.811	BB	0.2239	782.07947	52.48033	91.7469
2	12.364	BB	0.3099	70.35233	2.67030	8.2531
Totals :				852.43179	55.15063	

Figure S73. HPLC traces of (*S*)-**4a** (**Table S5, Entry 2**).

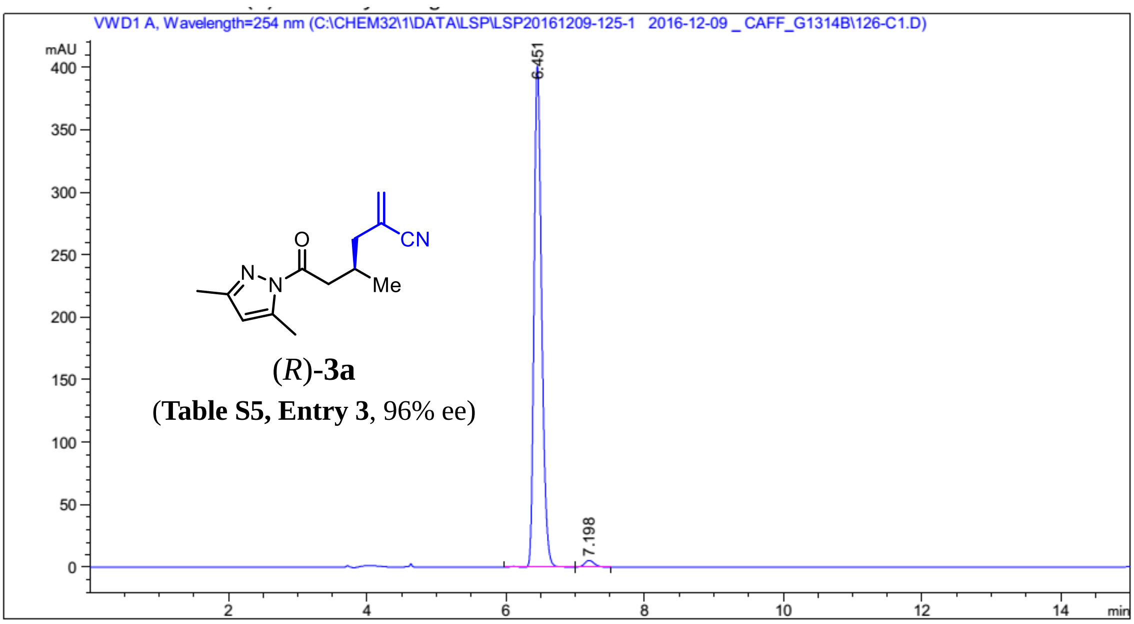

Peak #	RetTime [min]	Type	Width [min]	Area [mAU*s]	Height [mAU]	Area %
1	6.451	VV R	0.1169	3055.68896	401.11685	98.2458
2	7.198	VB	0.1364	54.56079	5.44695	1.7542
Totals :				3110.24975	406.56380	

Figure S74. HPLC traces of (*R*)-**3a** (**Table S5, Entry 3**).

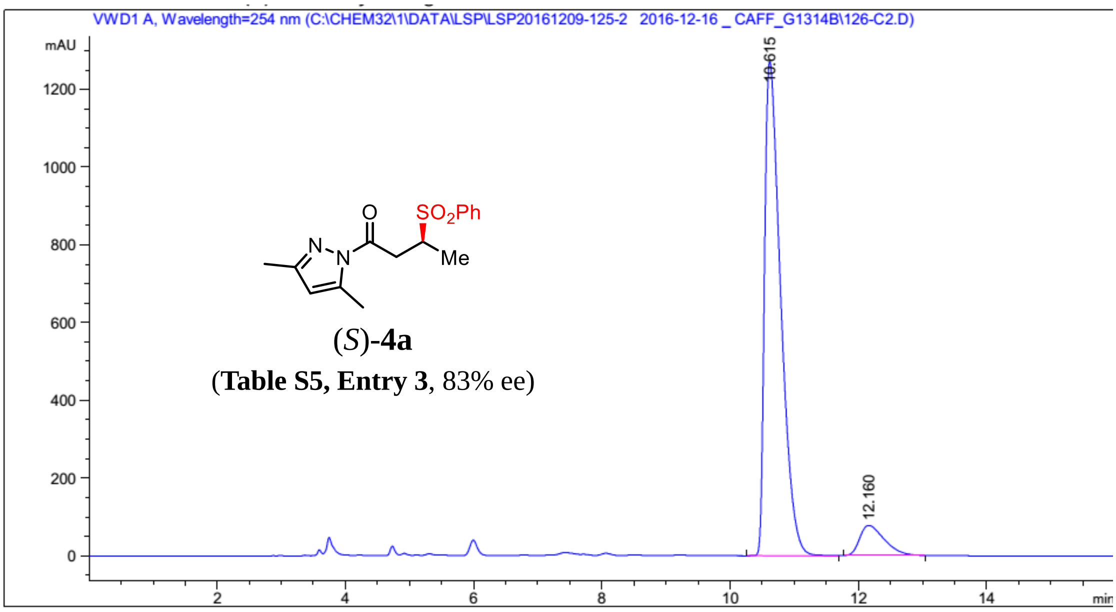

Peak #	RetTime [min]	Type	Width [min]	Area [mAU*s]	Height [mAU]	Area %
1	10.615	BV	0.2684	2.21730e4	1271.82153	91.4173
2	12.160	MM R	0.4465	2081.71436	77.70979	8.5827
Totals :				2.42547e4	1349.53132	

Figure S75. HPLC traces of (*S*)-**4a** (**Table S5, Entry 3**).

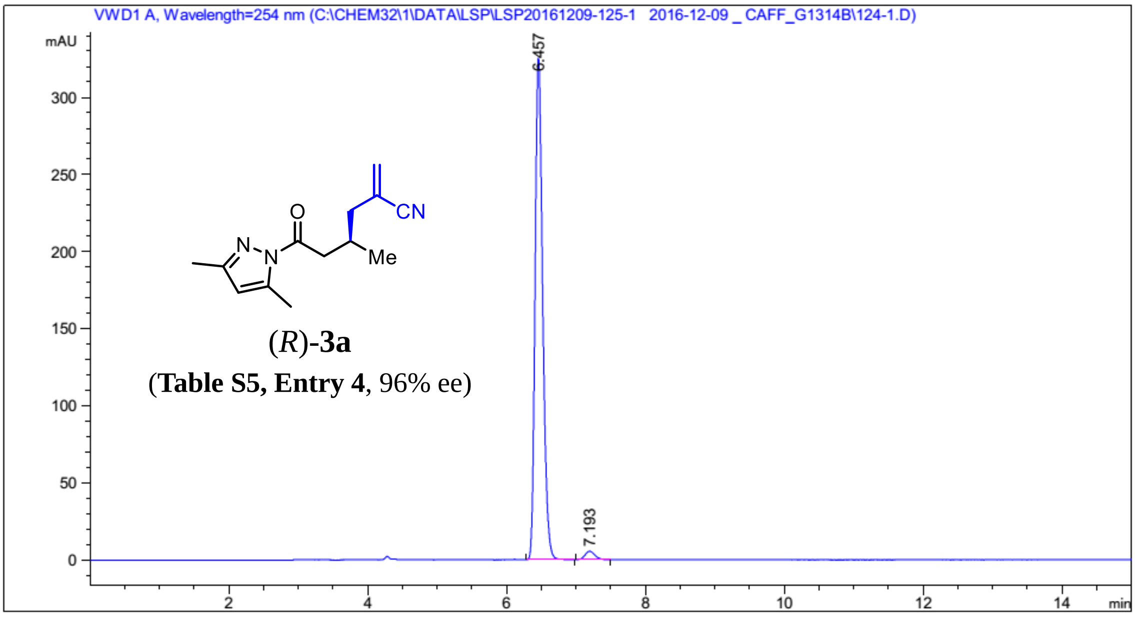

Peak #	RetTime [min]	Type	Width [min]	Area [mAU*s]	Height [mAU]	Area %
1	6.457	BB	0.1172	2476.61768	325.61740	97.8810
2	7.193	BB	0.1358	53.61487	5.44906	2.1190

Totals : 2530.23254 331.06646

Figure S76. HPLC traces of (*R*)-**3a** (**Table S5, Entry 4**).

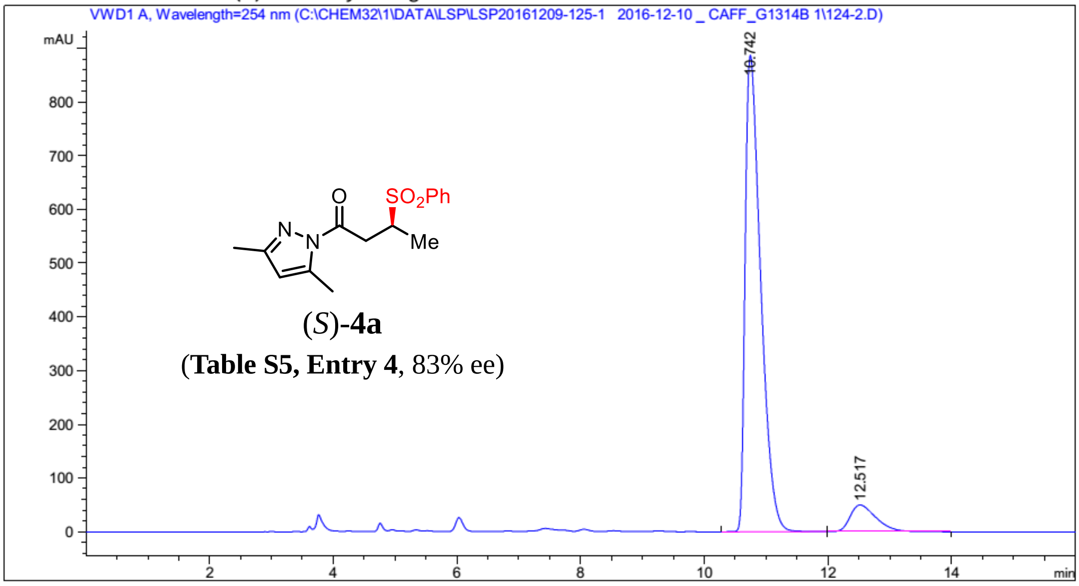

Peak #	RetTime [min]	Type	Width [min]	Area [mAU*s]	Height [mAU]	Area %
1	10.742	BB	0.2633	1.54602e4	887.03229	91.4393
2	12.517	BB	0.4237	1447.40479	49.78208	8.5607

Totals : 1.69076e4 936.81437

Figure S77. HPLC traces of (*S*)-**4a** (**Table S5, Entry 4**).

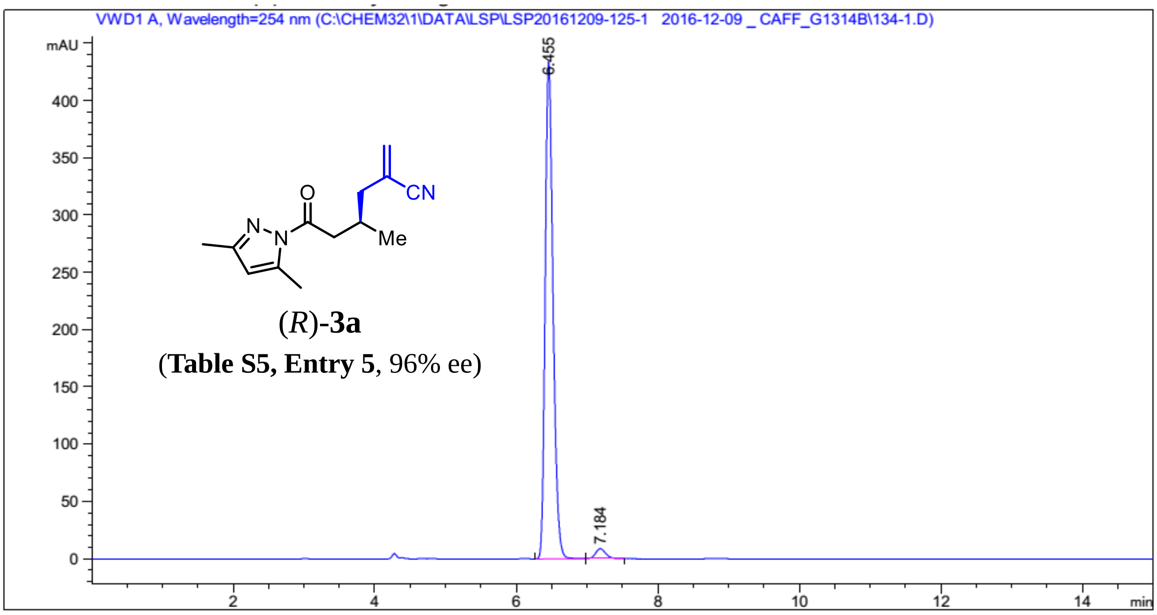

Peak #	RetTime [min]	Type	Width [min]	Area [mAU*s]	Height [mAU]	Area %
1	6.455	BV	0.1193	3356.32153	433.74936	97.4528
2	7.184	VB	0.1439	87.72746	8.71999	2.5472
Totals :				3444.04899	442.46935	

Figure S78. HPLC traces of (*R*)-**3a** (**Table S5, Entry 5**).

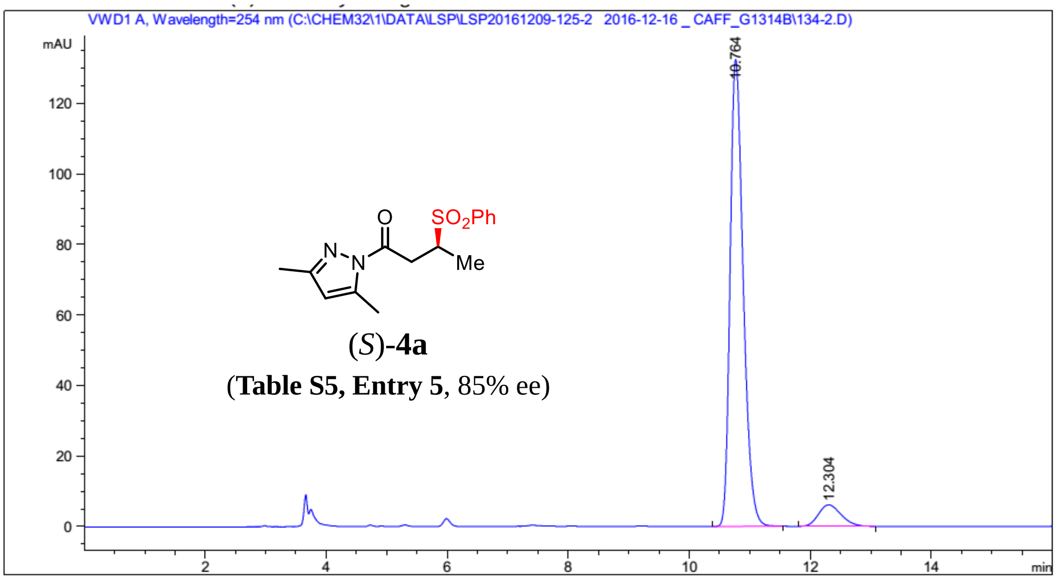

Peak #	RetTime [min]	Type	Width [min]	Area [mAU*s]	Height [mAU]	Area %
1	10.764	BB	0.2303	1987.87732	132.28204	92.4717
2	12.304	BB	0.3117	161.83772	6.12595	7.5283
Totals :				2149.71504	138.40800	

Figure S79. HPLC traces of (*S*)-**4a** (**Table S5, Entry 5**).

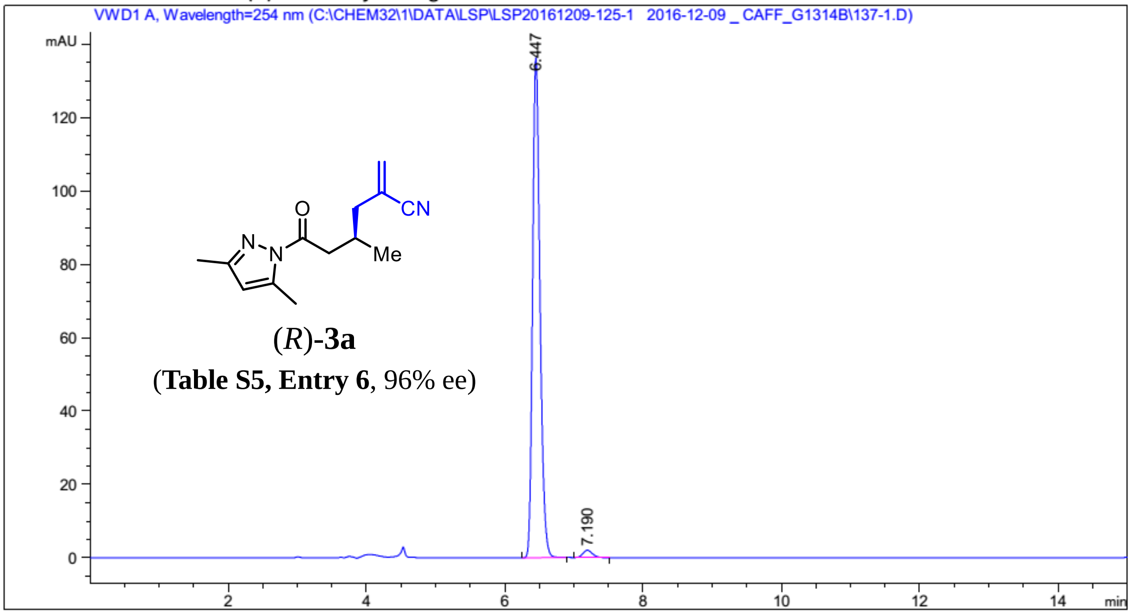

Peak #	RetTime [min]	Type	Width [min]	Area [mAU*s]	Height [mAU]	Area %
1	6.447	BB	0.1135	1011.05219	136.39075	98.0636
2	7.190	BB	0.1165	19.96458	2.03348	1.9364
Totals :				1031.01676	138.42423	

Figure S80. HPLC traces of (*R*)-**3a** (**Table S5, Entry 6**).

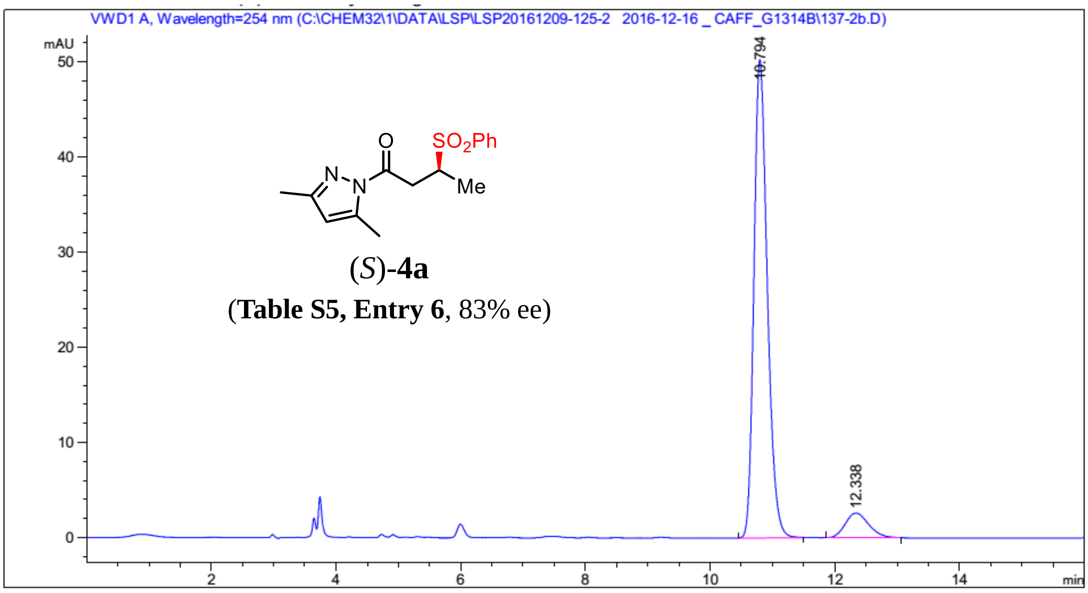

Peak #	RetTime [min]	Type	Width [min]	Area [mAU*s]	Height [mAU]	Area %
1	10.794	BB	0.2172	748.14874	50.26173	91.5862
2	12.338	BB	0.3100	68.73033	2.61181	8.4138
Totals :				816.87907	52.87354	

Figure S81. HPLC traces of (*S*)-**4a** (**Table S5, Entry 6**).

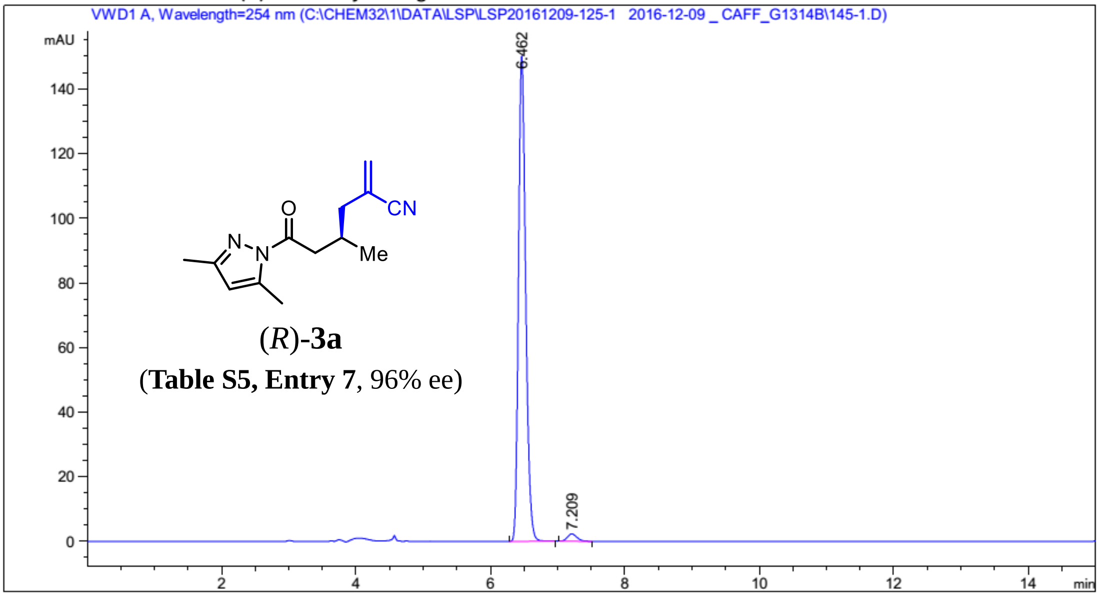

Peak #	RetTime [min]	Type	Width [min]	Area [mAU*s]	Height [mAU]	Area %
1	6.462	BB	0.1148	1116.55640	150.11342	98.0193
2	7.209	BB	0.1184	22.56307	2.28146	1.9807
Totals :				1139.11946	152.39488	

Figure S82**.** HPLC traces of (*R*)-**3a** (**Table S5, Entry 7**).

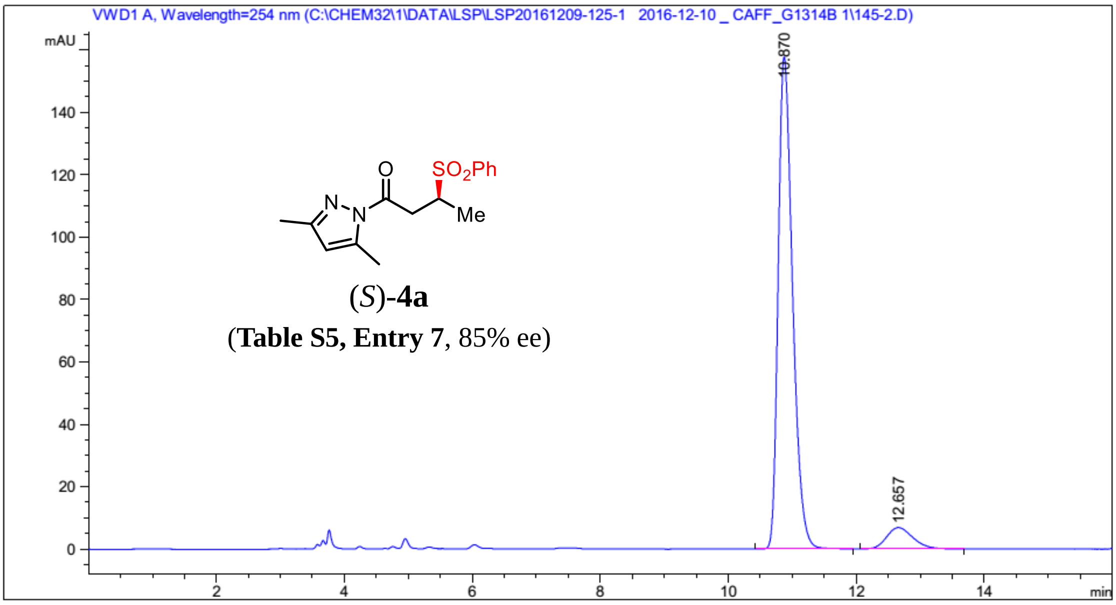

Peak #	RetTime [min]	Type	Width [min]	Area [mAU*s]	Height [mAU]	Area %
1	10.870	BB	0.2420	2460.53589	157.69205	92.5965
2	12.657	BB	0.3384	196.73201	6.84244	7.4035
Totals :				2657.26790	164.53449	

Figure S83**.** HPLC traces of (*S*)-**4a** (**Table S5, Entry 7**).

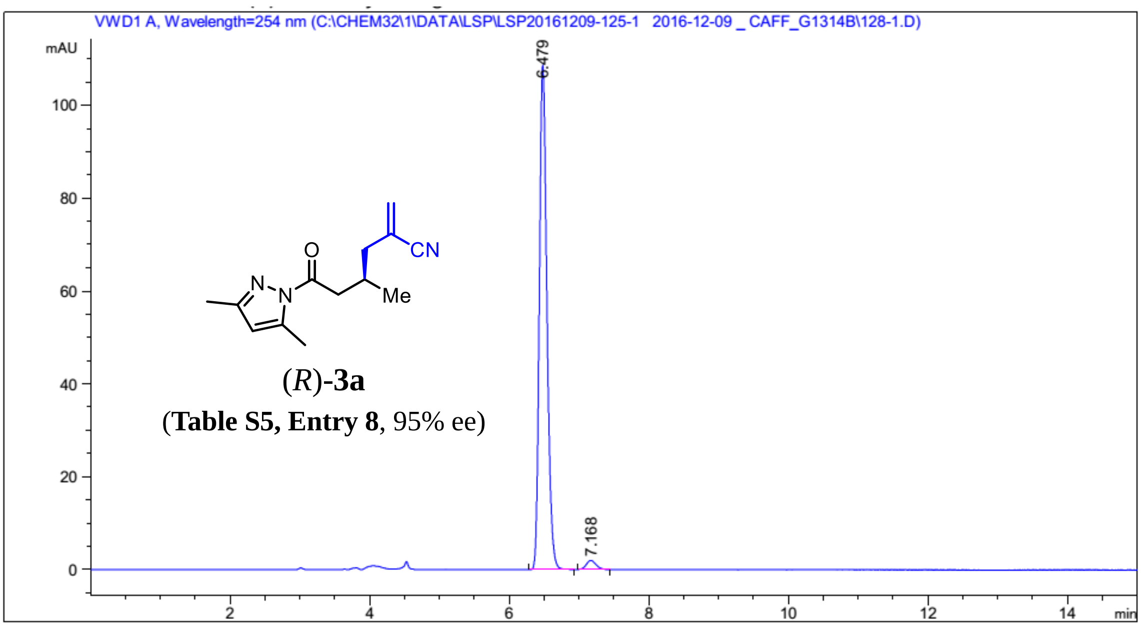

Peak #	RetTime [min]	Type	Width [min]	Area [mAU*s]	Height [mAU]	Area %
1	6.479	BB	0.1154	819.53131	108.75346	97.7045
2	7.168	BB	0.1192	19.25420	1.97549	2.2955
Totals :				838.78551	110.72894	

Figure S84. HPLC traces of (*R*)-**3a** (**Table S5, Entry 8**).

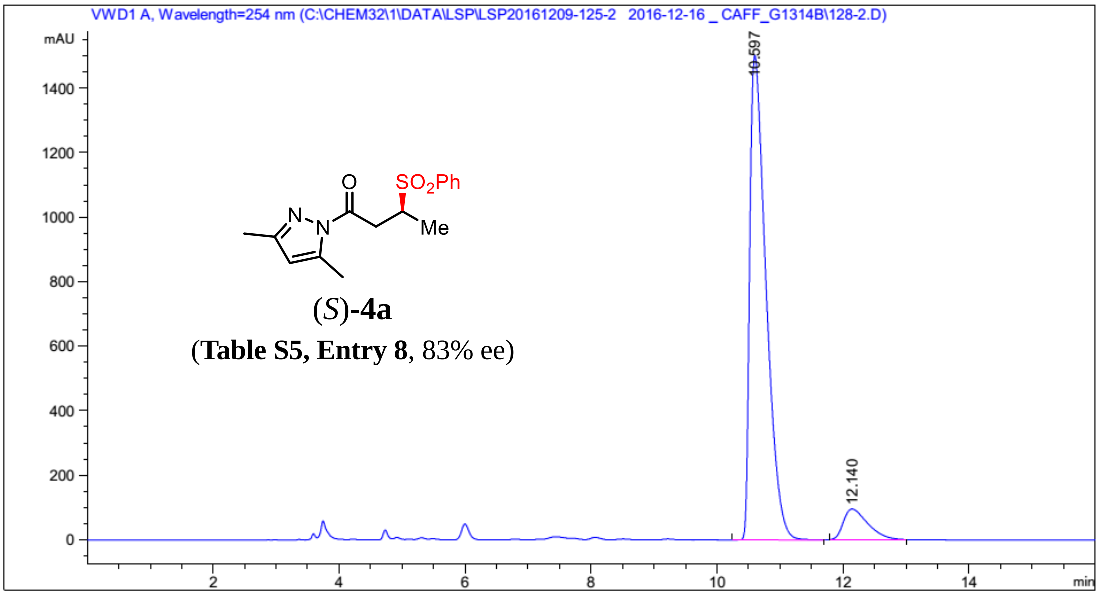

Peak #	RetTime [min]	Type	Width [min]	Area [mAU*s]	Height [mAU]	Area %
1	10.597	BV	0.2751	2.68923e4	1500.70374	91.3645
2	12.140	MM R	0.4474	2541.78296	94.68487	8.6355
Totals :				2.94340e4	1595.38860	

Figure S85. HPLC traces of (*S*)-**4a** (**Table S5, Entry 8**).

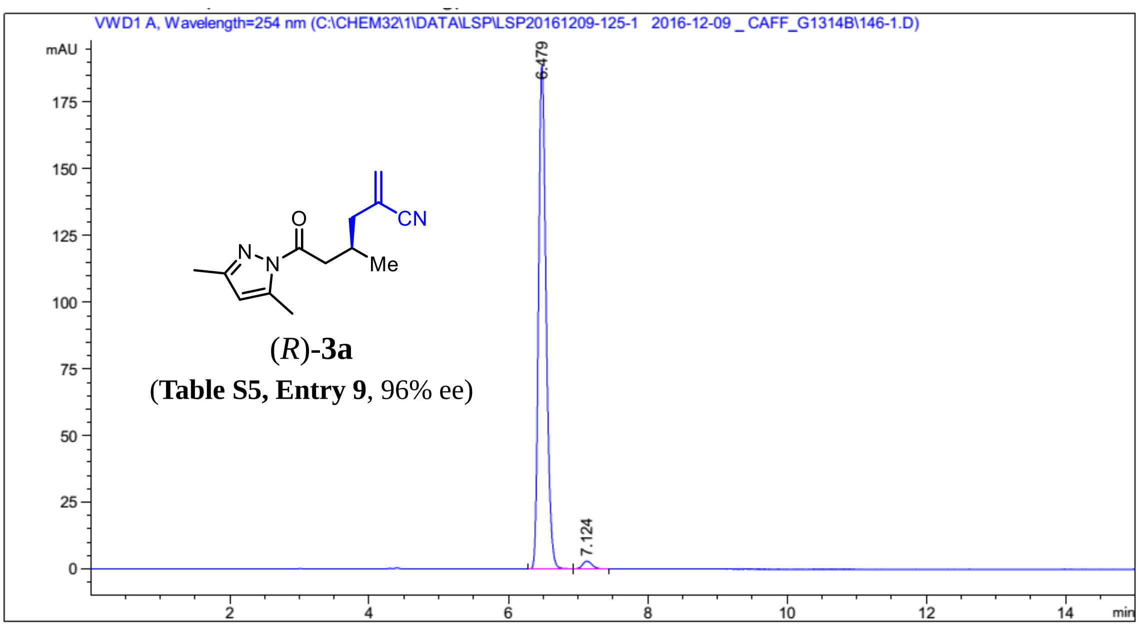

Peak #	RetTime [min]	Type	Width [min]	Area [mAU*s]	Height [mAU]	Area %
1	6.479	BV	0.1163	1429.18030	188.80899	97.9456
2	7.124	VB	0.1233	29.97697	3.02069	2.0544
Totals :				1459.15727	191.82968	

Figure S86. HPLC traces of (*R*)-**3a** (**Table S5, Entry 9**).

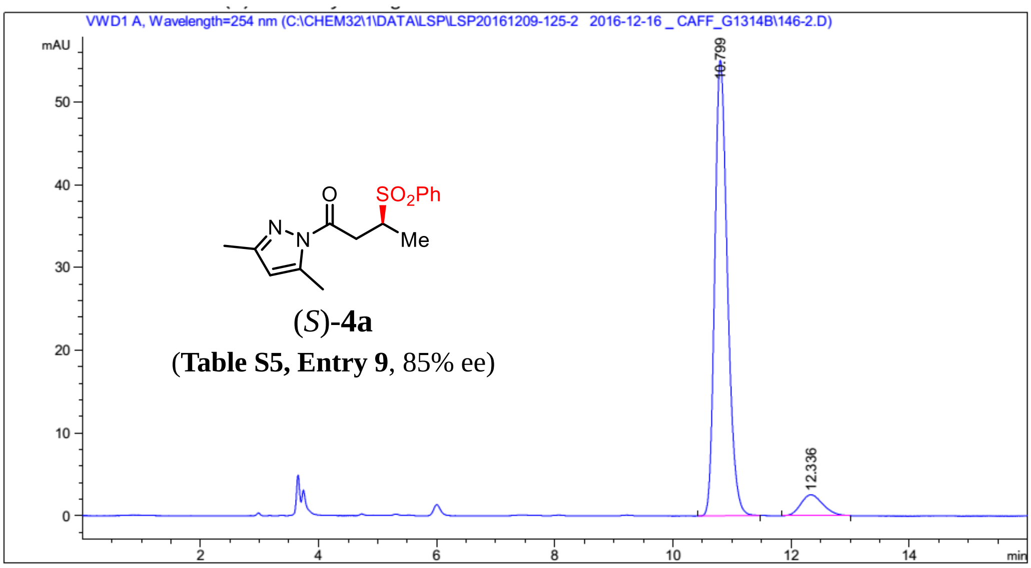

Peak #	RetTime [min]	Type	Width [min]	Area [mAU*s]	Height [mAU]	Area %
1	10.799	BB	0.2270	818.55615	55.05112	92.4759
2	12.336	BB	0.3082	66.60007	2.53722	7.5241
Totals :				885.15623	57.58834	

Figure S87. HPLC traces of (*S*)-**4a** (**Table S5, Entry 9**).

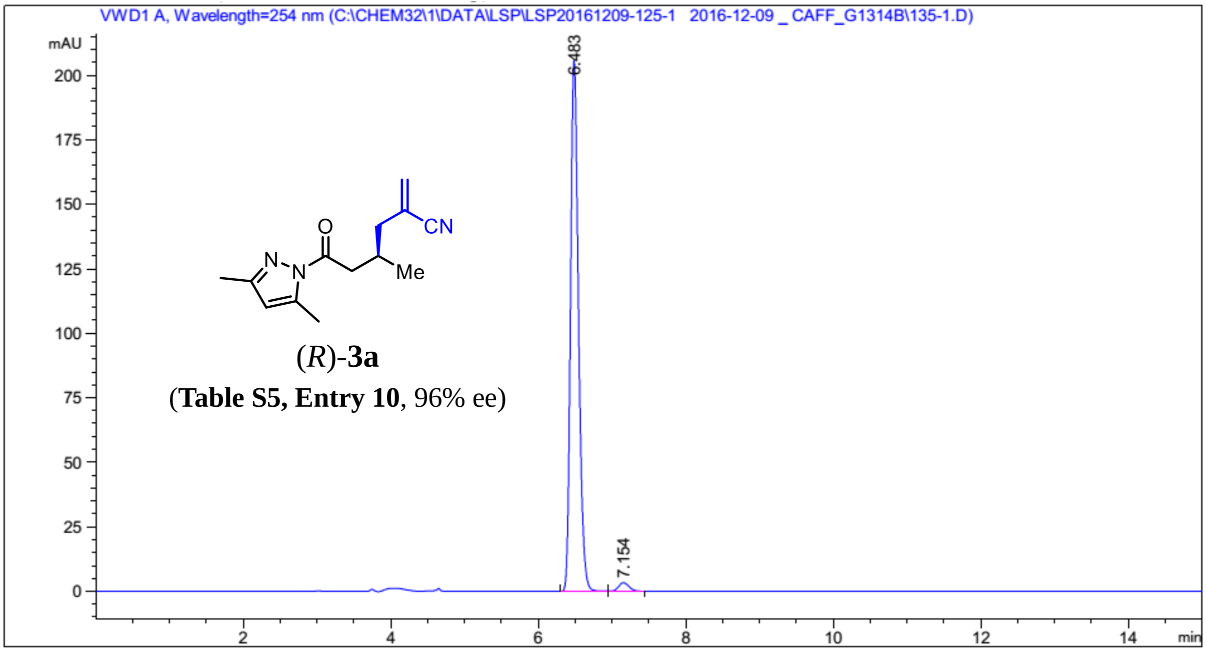

Peak #	RetTime [min]	Type	Width [min]	Area [mAU*s]	Height [mAU]	Area %
1	6.483	BV	0.1162	1545.66345	205.69731	97.8973
2	7.154	VV R	0.1252	33.19832	3.37754	2.1027
Totals :				1578.86177	209.07485	

Figure S88. HPLC traces of (*R*)-**3a** (**Table S5, Entry 10**).

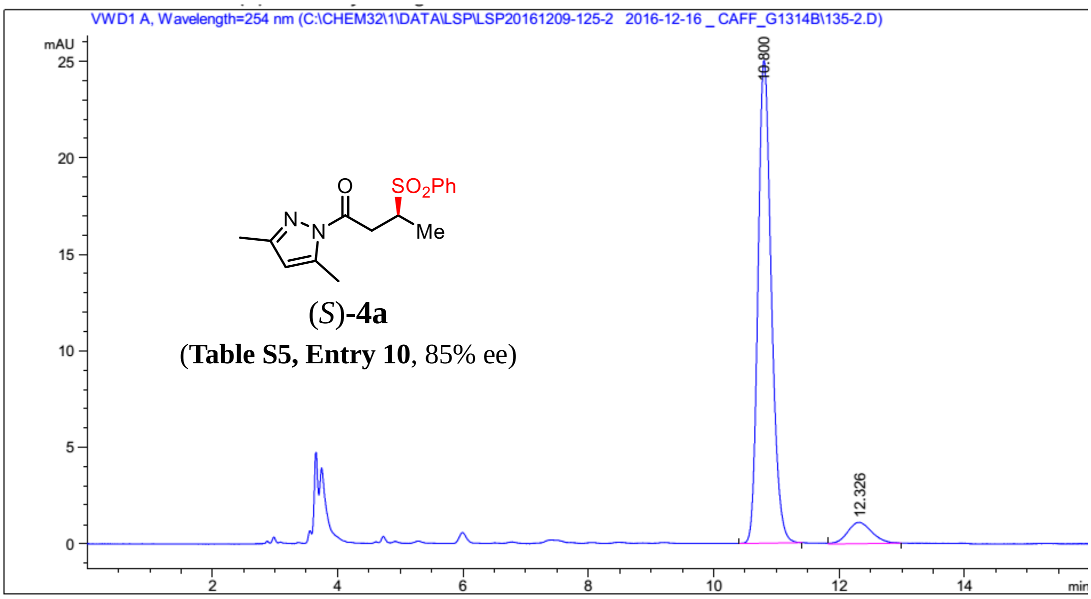

Peak #	RetTime [min]	Type	Width [min]	Area [mAU*s]	Height [mAU]	Area %
1	10.800	BB	0.2108	369.50555	25.01874	92.5117
2	12.326	MM R	0.4495	29.90926	1.10889	7.4883
Totals :				399.41482	26.12763	

Figure S89. HPLC traces of (*S*)-**4a** (**Table S5, Entry 10**).

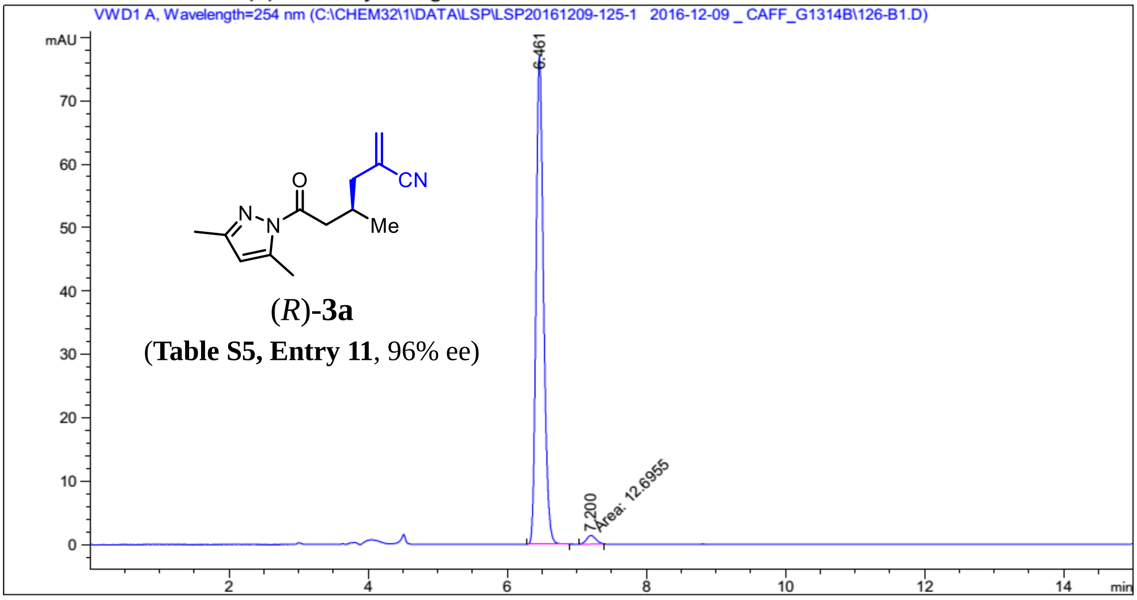

Peak #	RetTime [min]	Type	Width [min]	Area [mAU*s]	Height [mAU]	Area %
1	6.461	BB	0.1137	575.20874	76.99812	97.8406
2	7.200	MM	0.1567	12.69548	1.35043	2.1594
Totals :				587.90422	78.34854	

Figure S90**.** HPLC traces of (*R*)-**3a** (**Table S5, Entry 11**).

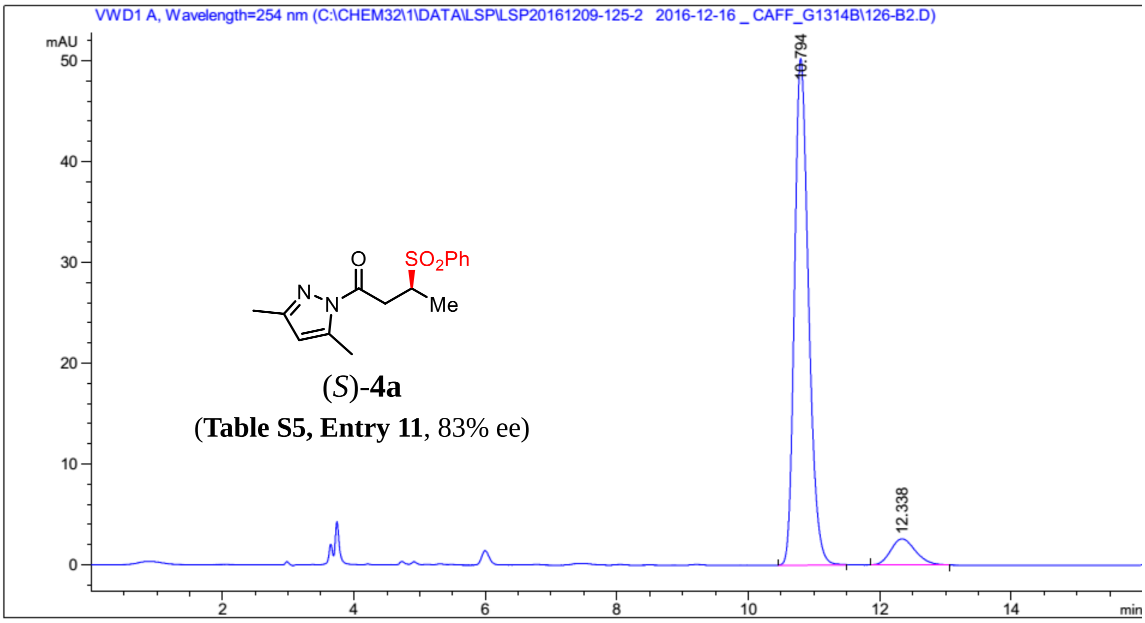

Peak #	RetTime [min]	Type	Width [min]	Area [mAU*s]	Height [mAU]	Area %
1	10.794	BB	0.2172	748.14874	50.26173	91.5862
2	12.338	BB	0.3100	68.73033	2.61181	8.4138
Totals :				816.87907	52.87354	

Figure S91**.** HPLC traces of (*S*)-**4a** (**Table S5, Entry 11**).

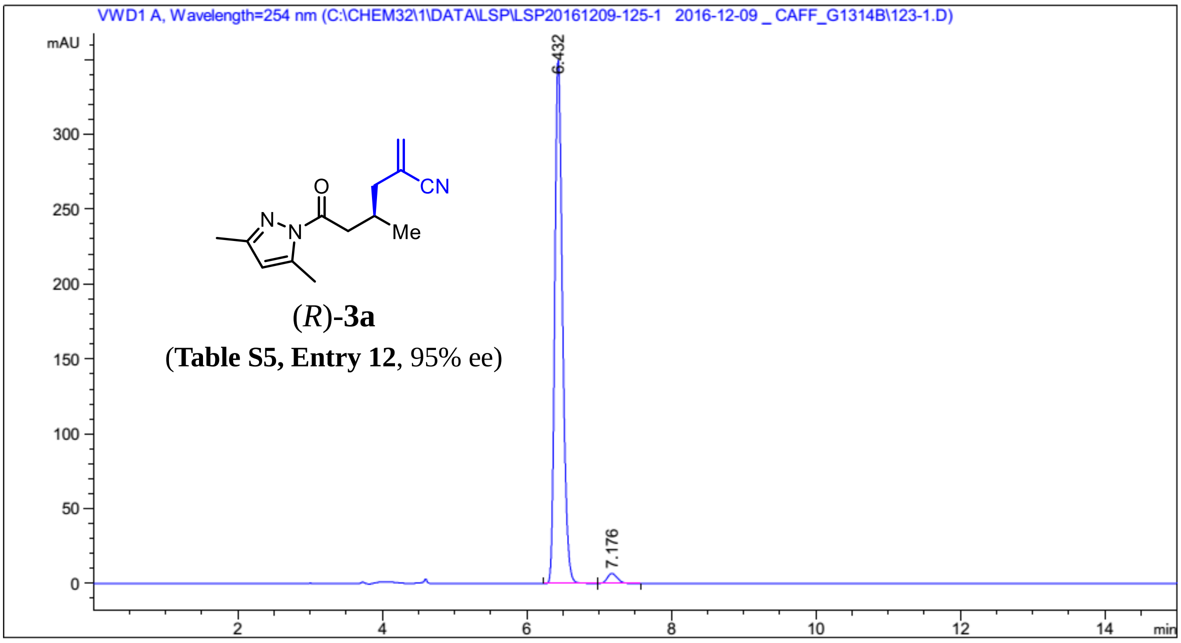

Peak #	RetTime [min]	Type	Width [min]	Area [mAU*s]	Height [mAU]	Area %
1	6.432	BV	0.1144	2603.62646	349.56348	97.4476
2	7.176	VB	0.1424	68.19535	6.80554	2.5524
Totals :				2671.82182	356.36902	

Figure S92. HPLC traces of (*R*)-**3a** (**Table S5, Entry 12**).

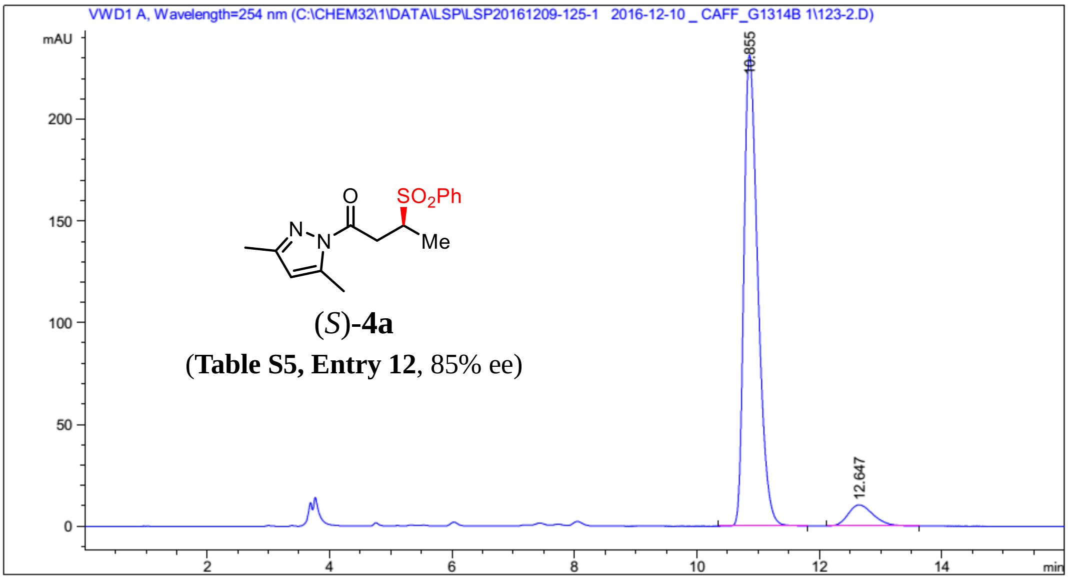

Peak #	RetTime [min]	Type	Width [min]	Area [mAU*s]	Height [mAU]	Area %
1	10.855	BB	0.2403	3659.43335	231.71220	92.4404
2	12.647	BB	0.3384	299.26288	10.38002	7.5596
Totals :				3958.69623	242.09222	

Figure S93. HPLC traces of (*S*)-**4a** (**Table S5, Entry 12**).

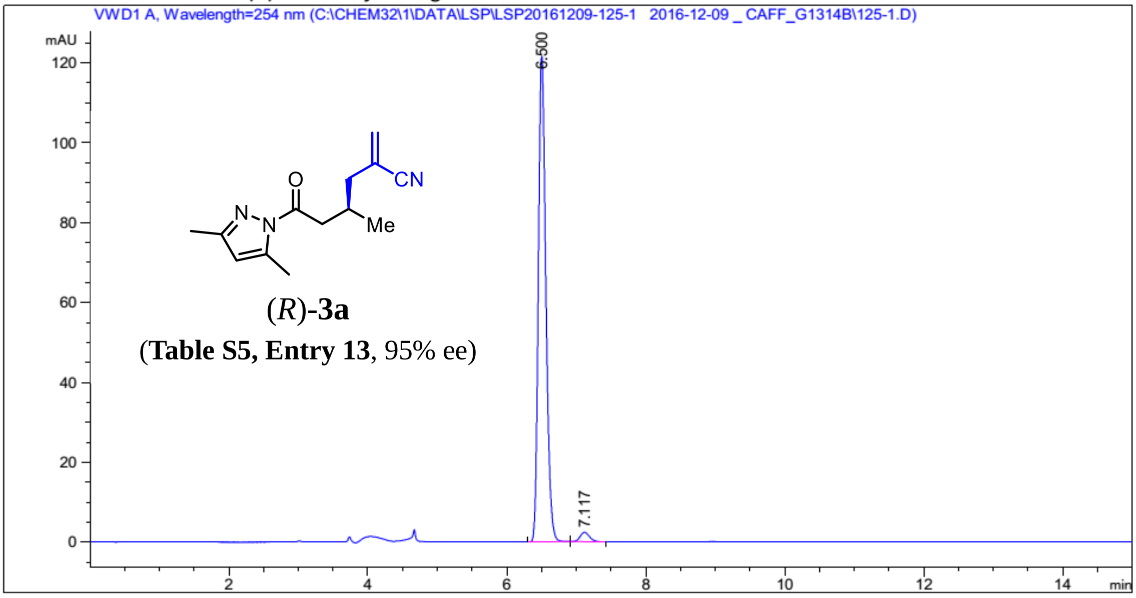

Peak #	RetTime [min]	Type	Width [min]	Area [mAU*s]	Height [mAU]	Area %
1	6.500	BB	0.1155	911.76398	121.61312	97.5853
2	7.117	BB	0.1205	22.56141	2.36085	2.4147
Totals :				934.32539	123.97397	

Figure S94. HPLC traces of (*R*)-**3a** (**Table S5, Entry 13**).

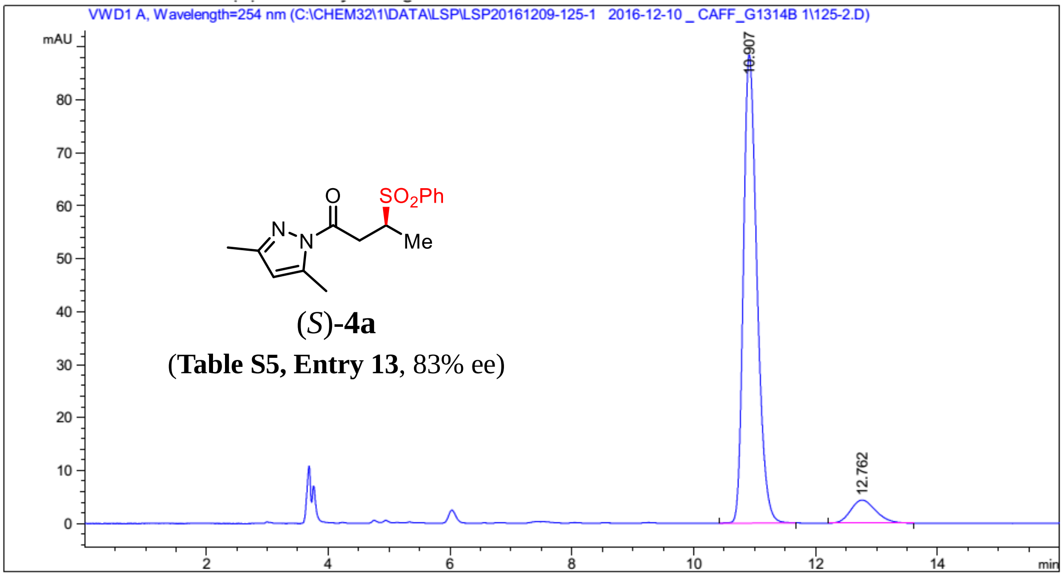

Peak #	RetTime [min]	Type	Width [min]	Area [mAU*s]	Height [mAU]	Area %
1	10.907	BB	0.2338	1367.08386	88.46869	91.4742
2	12.762	BB	0.3398	127.41823	4.39465	8.5258
Totals :				1494.50209	92.86334	

Figure S95. HPLC traces of (*S*)-**4a** (**Table S5, Entry 13**).

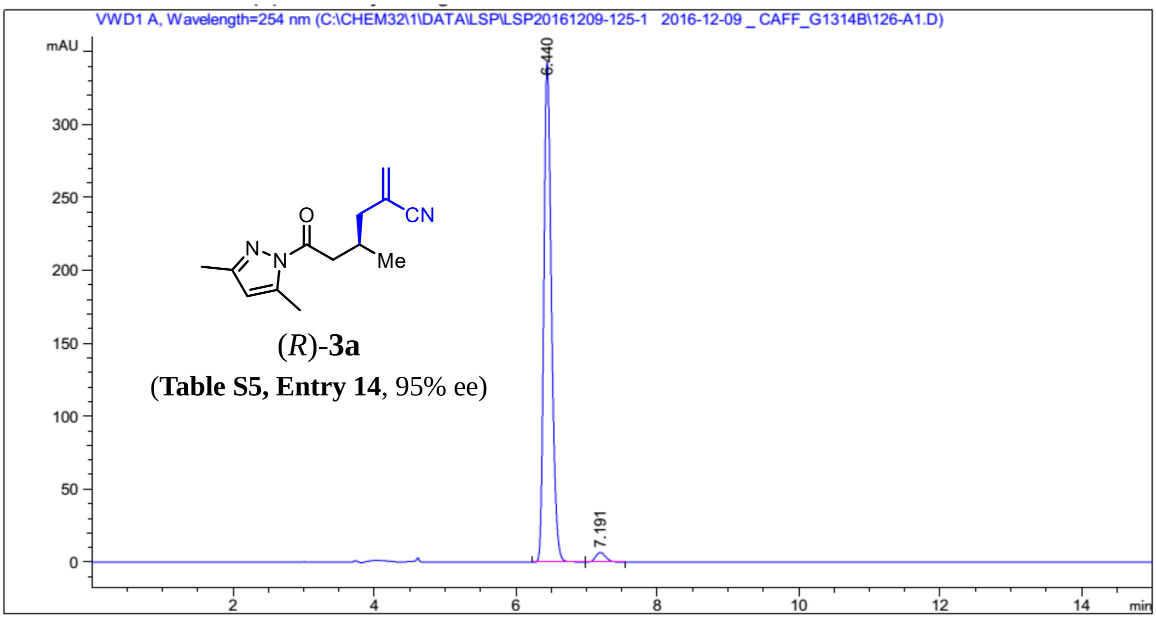

Peak #	RetTime [min]	Type	Width [min]	Area [mAU*s]	Height [mAU]	Area %
1	6.440	BV	0.1155	2569.82129	342.66757	97.4529
2	7.191	VB	0.1446	67.16708	6.69328	2.5471
Totals :				2636.98837	349.36085	

Figure S96. HPLC traces of (*R*)-**3a** (**Table S5, Entry 14**).

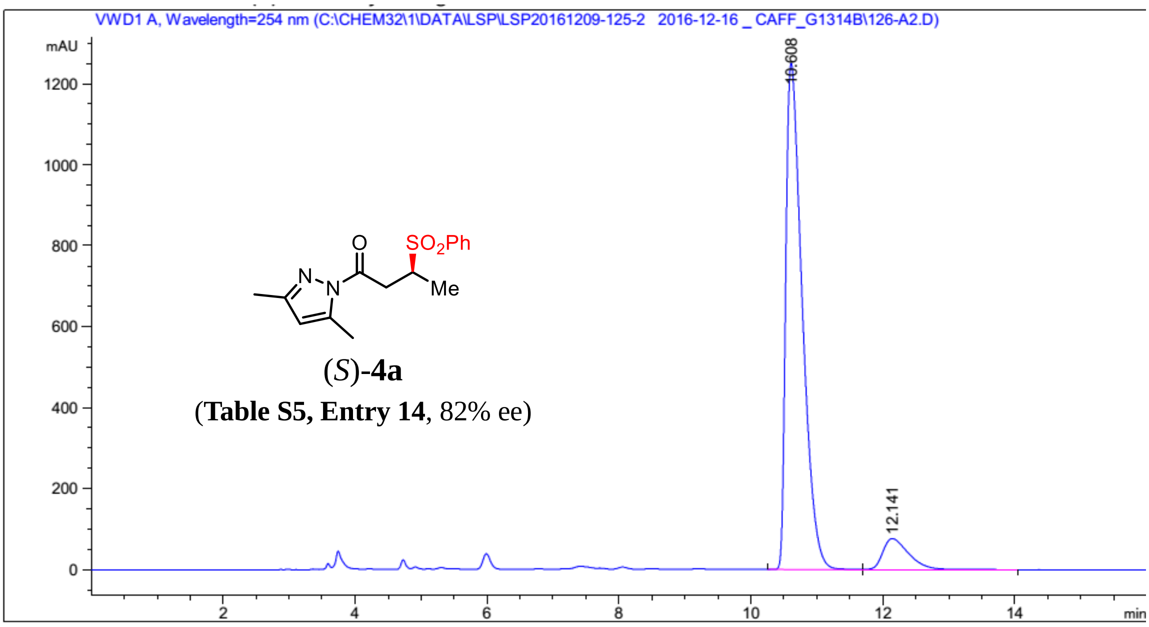

Peak #	RetTime [min]	Type	Width [min]	Area [mAU*s]	Height [mAU]	Area %
1	10.608	BV	0.2633	2.15952e4	1251.33691	91.2047
2	12.141	VB	0.4103	2082.53076	76.57831	8.7953
Totals :				2.36778e4	1327.91522	

Figure S97. HPLC traces of (*S*)-**4a** (**Table S5, Entry 14**).

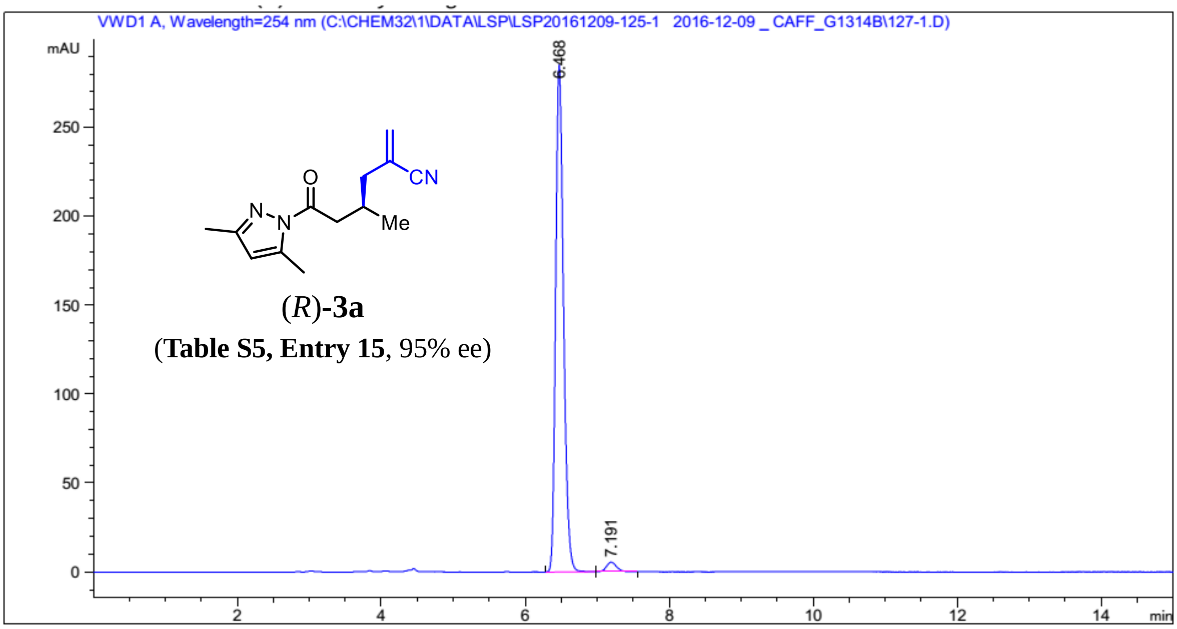

Peak #	RetTime [min]	Type	Width [min]	Area [mAU*s]	Height [mAU]	Area %
1	6.468	BV	0.1166	2163.41528	284.96329	97.5544
2	7.191	VV R	0.1256	54.23380	5.42511	2.4456
Totals :				2217.64908	290.38840	

Figure S98. HPLC traces of (*R*)-**3a** (**Table S5, Entry 15**).

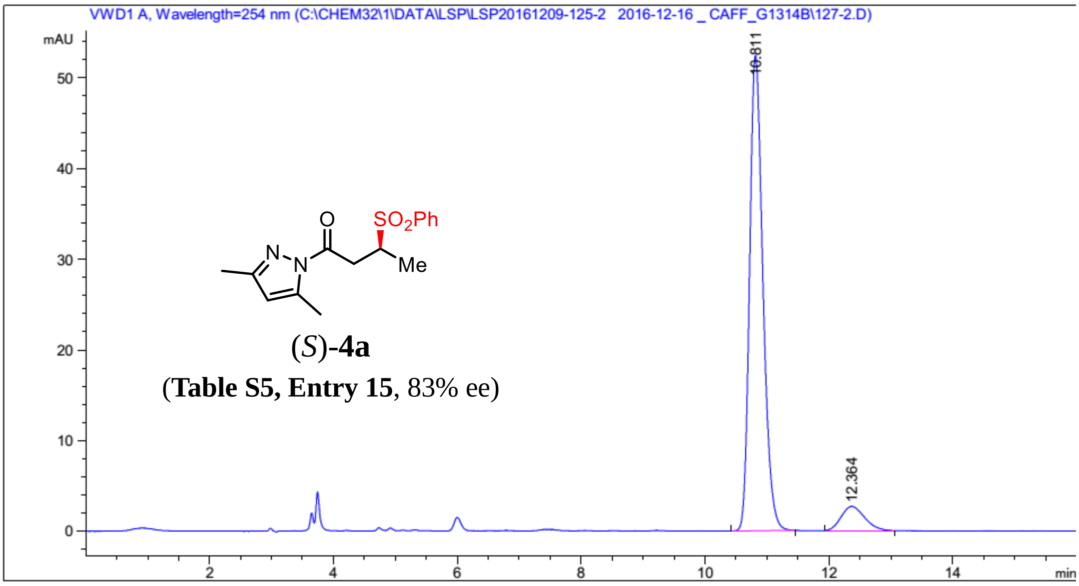

Peak #	RetTime [min]	Type	Width [min]	Area [mAU*s]	Height [mAU]	Area %
1	10.811	BB	0.2239	782.07947	52.48033	91.7469
2	12.364	BB	0.3099	70.35233	2.67030	8.2531
Totals :				852.43179	55.15063	

Figure S99. HPLC traces of (*S*)-**4a** (**Table S5, Entry 15**).

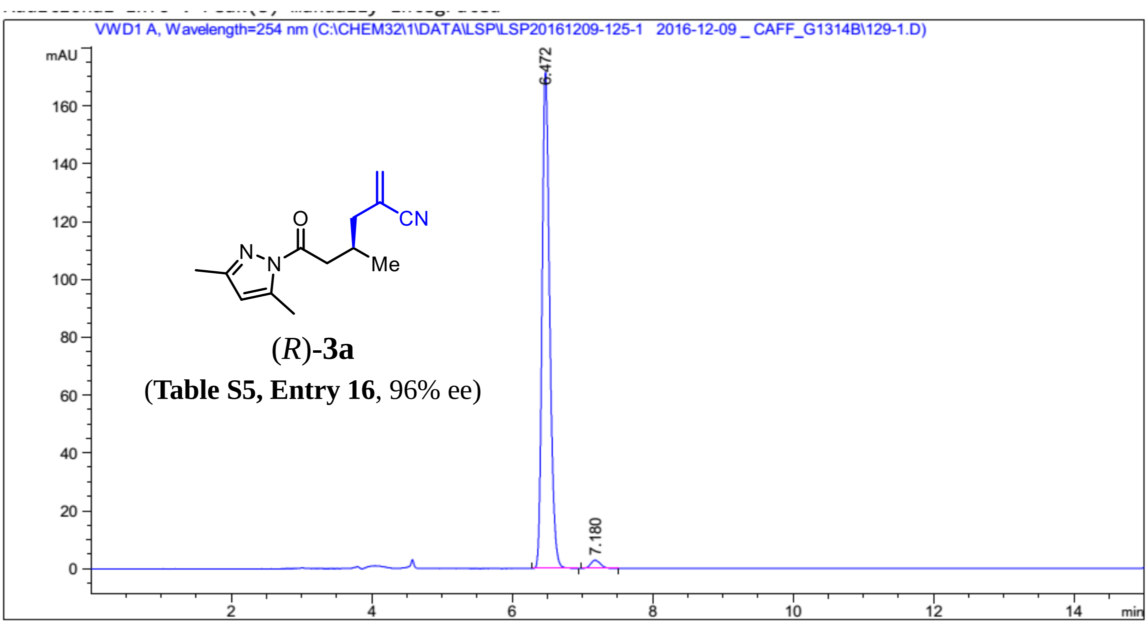

Peak #	RetTime [min]	Type	Width [min]	Area [mAU*s]	Height [mAU]	Area %
1	6.472	BB	0.1143	1292.17407	171.70543	97.8893
2	7.180	BB	0.1279	27.86150	2.86575	2.1107
Totals :				1320.03557	174.57118	

Figure S100. HPLC traces of (*R*)-**3a** (**Table S5, Entry 16**).

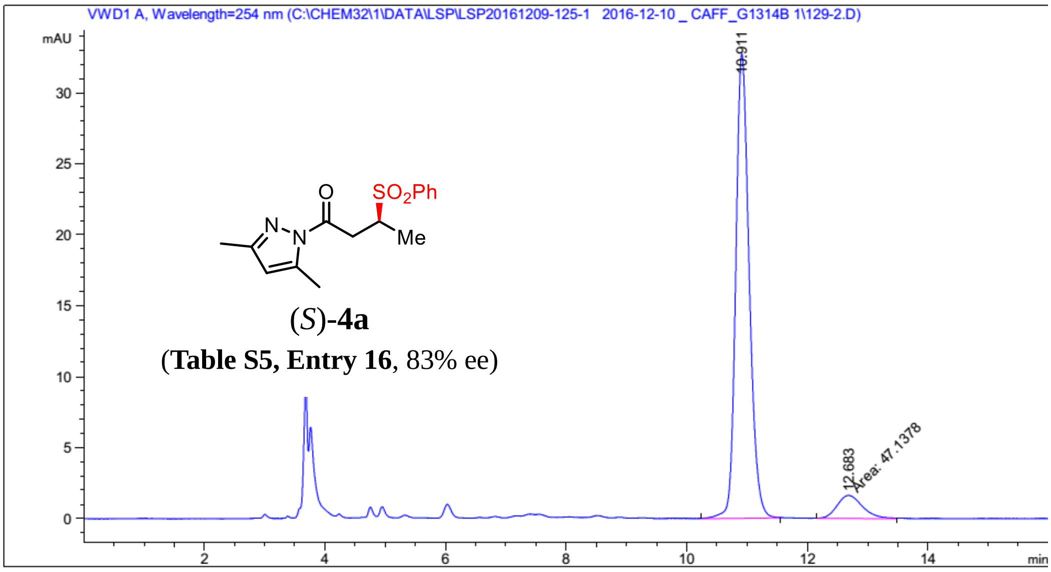

Peak #	RetTime [min]	Type	Width [min]	Area [mAU*s]	Height [mAU]	Area %
1	10.911	BB	0.2297	506.44687	32.70663	91.4850
2	12.683	MM	0.4843	47.13784	1.62223	8.5150
Totals :				553.58471	34.32886	

Figure S101. HPLC traces of (*S*)-**4a** (**Table S5, Entry 16**).

11. Single-Crystal X-Ray Diffraction Studies

X-ray data were collected with a Bruker 3 circuit D8 Quest diffractometer with MoKα radiation (microfocus tube with multilayer optics) and Photon 100 CMOS detector at 100 K. Scaling and absorption correction was performed by using the SADABS software package of Bruker. Structures were solved using direct methods in SHELXT and refined using the full matrix least squares procedure in SHELXL-2014. The hydrogen atoms were placed in calculated positions and refined as riding on their respective C atom, and Uiso(H) was set at 1.2 Ueq(Csp^2) and 1.5 Ueq(Csp^3). Disorder was refined using restraints for both the geometry and the anisotropic displacement factors. The absolute configuration of **4d** and **9** have been determined.

11.1 Crystal structure of RhO-1a

Single crystals of **RhO-1a** suitable for X-ray diffraction were obtained by slow diffusion from a solution of raacemic **RhO-1a** (20 mg) in CH_2Cl_2 (2.0 mL) layered with ethyl ether (1.0 mL) at room temperature for several days in a NMR tube.

Crystal structure, data and details of the structure determination for **RhO-1a** are presented in the Figure S102 and Table S6.

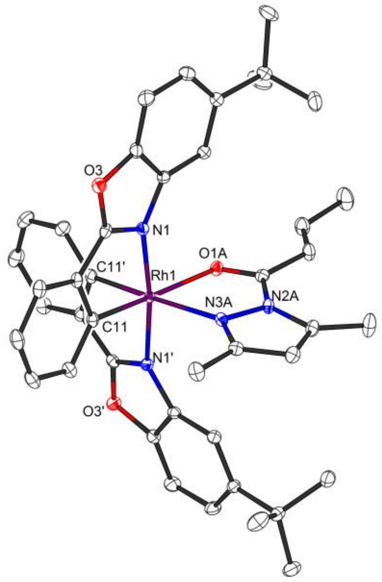

Figure S102. Crystal structure of **RhO-1a**.

Table S6. Crystal data and structure refinement for **RhO-1a**.

Crystal data		
Identification code	hxqE104_0m	
Habitus, colour	nugget, pale yellow	
Crystal size	0.34 x 0.25 x 0.23 mm^3	
Crystal system	Triclinic	
Space group	P-1	Z = 2
Unit cell dimensions	a = 11.9931(5) Å	α = 89.480(1)°.
	b = 13.7713(6) Å	β = 67.088(1)°.
	c = 14.7325(6) Å	γ = 79.592(1)°.
Volume	2199.33(16) $Å^3$	
3Cell determination	9783 peaks with Theta 2.6 to 27.5°.	
Empirical formula	$C_{44} H_{46} Cl_2 F_6 N_4 O_3 P Rh$	
Moiety formula	$C_{43} H_{44} N_4 O_3 Rh, F_6 P, C H_2 Cl_2$	
Formula weight	997.63	
Density (calculated)	1.506 Mg/m^3	
Absorption coefficient	0.616 mm^{-1}	
F(000)	1020	
Data collection:		
Diffractometer type	Bruker D8 QUEST area detector	
Wavelength	0.71073 Å	
Temperature	100(2) K	
Theta range for data collection	2.248 to 27.564°.	
Index ranges	-15<=h<=15, -17<=k<=17, -19<=l<=18	
Data collection software	APEX3 (Bruker AXS Inc., 2015)[15]	
Cell refinement software	SAINT V8.35A (Bruker AXS Inc., 2015)[16]	
Data reduction software	SAINT V8.35A (Bruker AXS Inc., 2015)	
Solution and refinement:		
Reflections collected	82467	
Independent reflections	10124 [R(int) = 0.0338]	
Completeness to theta = 25.242°	99.9 %	
Observed reflections	9422[I > 2(I)]	
Reflections used for refinement	10124	
Absorption correction	Semi-empirical from equivalents[17]	
Max. and min. transmission	0.87 and 0.82	
Largest diff. peak and hole	0.438 and -0.606 e.$Å^{-3}$	
Solution	Direct methods	
Refinement	Full-matrix least-squares on F^2	
Treatment of hydrogen atoms	Calculated positions, constr. ref.	
Programs used	XT V2014/1 (Bruker AXS Inc., 2014)[18]	
	SHELXL-2014/7 (Sheldrick, 2014)[19]	
	DIAMOND (Crystal Impact)[20]	
	ShelXle (Hübschle, Sheldrick, Dittrich, 2011)[21]	
Data / restraints / parameters	10124 / 144 / 590	
Goodness-of-fit on F^2	1.038	
R index (all data)	wR2 = 0.0560	
R index conventional [I>2sigma(I)]	R1 = 0.0223	

11.2 Crystal structure of 4d

Single crystals of **4d** suitable for X-ray diffraction were obtained by slow diffusion from a solution of **4d** (20 mg) in ethyl ether (0.5 mL) layered with n-hexane (0.5 mL) at 4 ^{o}C for several days in a NMR tube.

Crystal structure, data and details of the structure determination for **4d** are presented in the Figure S103 and Table S7.

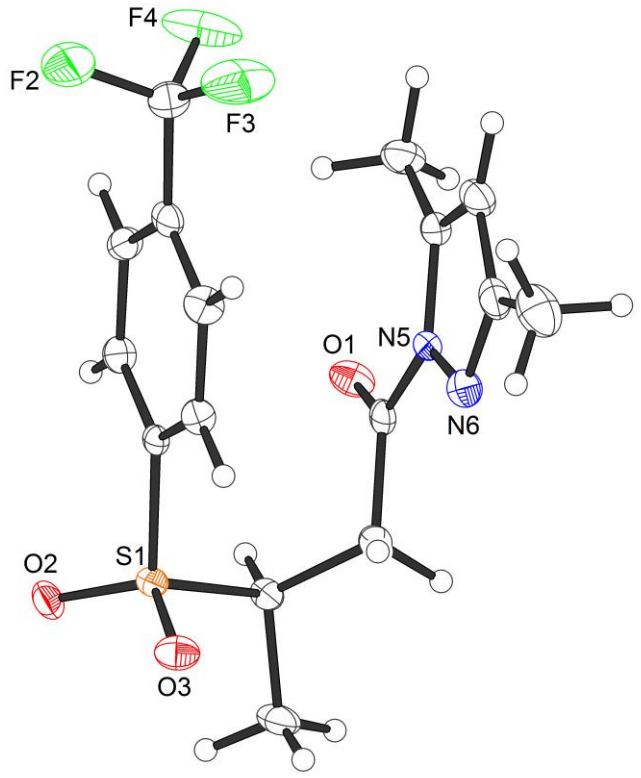

Figure S103. Crystal structure of **4d**.

Table S7. Crystal data and structure refinement for **4d**.

Crystal data		
Identification code	hxq119b_0m	
Habitus, colour	block, colourless	
Crystal size	0.31 x 0.13 x 0.08 mm^3	
Crystal system	Tetragonal	
Space group	$P4_32_12$	Z = 8
Unit cell dimensions	a = 7.1300(2) Å	α = 90°.
	b = 7.1300(2) Å	β = 90°.
	c = 66.238(2) Å	γ = 90°.
Volume	3367.3(2) $Å^3$	
Cell determination	9348 peaks with Theta 2.5 to 25.2°.	
Empirical formula	$C_{16} H_{17} F_3 N_2 O_3 S$	
Moiety formula	$C_{16} H_{17} F_3 N_2 O_3 S$	
Formula weight	374.37	
Density (calculated)	1.477 Mg/m^3	
Absorption coefficient	0.242 mm^{-1}	
F(000)	1552	
Data collection:		
Diffractometer type	Bruker D8 QUEST area detector	
Wavelength	0.71073 Å	
Temperature	110(2) K	
Theta range for data collection	2.460 to 25.262°.	
Index ranges	-8<=h<=8, -8<=k<=8, -79<=l<=79	
Data collection software	APEX3 (Bruker AXS Inc., 2015)[15]	
Cell refinement software	SAINT V8.37A (Bruker AXS Inc., 2015)[16]	
Data reduction software	SAINT V8.37A (Bruker AXS Inc., 2015)	
Solution and refinement:		
Reflections collected	35175	
Independent reflections	3048 [R(int) = 0.0499]	
Completeness to theta = 25.242°	99.9 %	
Observed reflections	2921[$I > 2\sigma(I)$]	
Reflections used for refinement	3048	
Absorption correction	Semi-empirical from equivalents[17]	
Max. and min. transmission	0.98 and 0.84	
Flack parameter (absolute struct.)	0.00(3)[22]	
Largest diff. peak and hole	0.308 and -0.260 e.$Å^{-3}$	
Solution	"Dual space"	
Refinement	Full-matrix least-squares on F^2	
Treatment of hydrogen atoms	Calculated positions, constr. ref.	
Programs used	XT V2014/1 (Bruker AXS Inc., 2014)[18]	
	SHELXL-2014/7 (Sheldrick, 2014)[19]	
	DIAMOND (Crystal Impact)[20]	
	ShelXle (Hübschle, Sheldrick, Dittrich, 2011)[21]	
Data / restraints / parameters	3048 / 0 / 229	
Goodness-of-fit on F^2	1.224	
R index (all data)	wR2 = 0.1020	
R index conventional [I>2sigma(I)]	R1 = 0.0479	

11.3 Crystal structure of 9

Single crystals of compound **9**, which was obtained via transamidation of (*R*)-**3a** (*obtained from the reactions catalyzed by Λ-**RhO***, suitable for X-ray diffraction were obtained by slow diffusion from a solution of **9** (30 mg) see Section 8.3), in CH_2Cl_2 (0.5 mL) layered with n-hexane (0.5 mL) at room temperature for several days in a NMR tube.

Crystal structure, data and details of the structure determination for **9** are presented in the Figure S104 and Table S8.

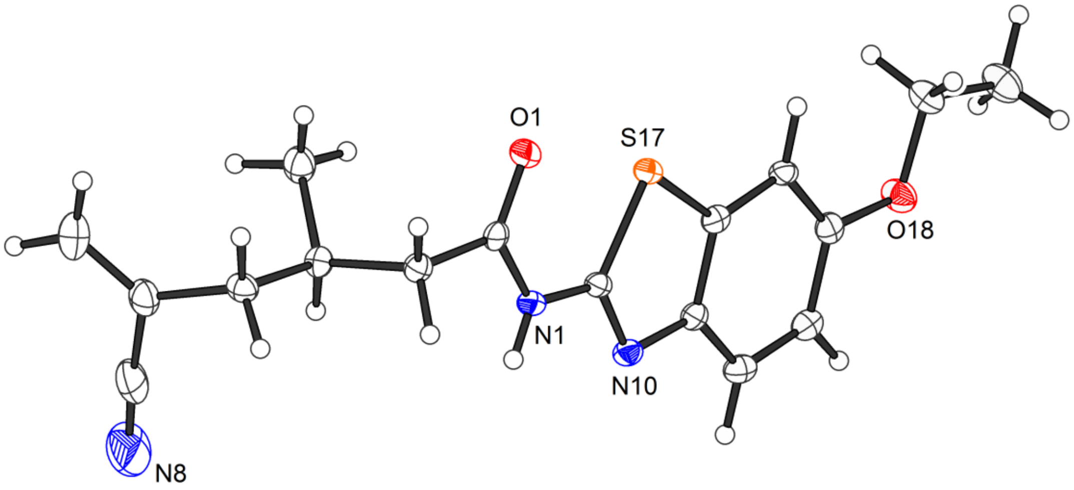

Figure S104. Crystal structure of **9**.

Table S8. Crystal data and structure refinement for **9**.

Crystal data

Identification code	lsp182_0m	
Habitus, colour	plate, colourless	
Crystal size	0.45 x 0.40 x 0.04 mm^3	
Crystal system	Monoclinic	
Space group	$P2_1$	Z = 8
Unit cell dimensions	a = 15.7976(9) Å	α = 90°.
	b = 12.9960(8) Å	β = 112.625(2)°.
	c = 17.9469(11) Å	γ = 90°.
Volume	3401.0(4) $Å^3$	
Cell determination	9782 peaks with Theta 2.7 to 26.4°.	
Empirical formula	$C_{17} H_{19} N_3 O_2 S$	
Moiety formula	$C_{17} H_{19} N_3 O_2 S$	
Formula weight	329.41	
Density (calculated)	1.287 Mg/m^3	
Absorption coefficient	0.203 mm^{-1}	
F(000)	1392	

Data collection:

Diffractometer type	Bruker D8 QUEST area detector
Wavelength	0.71073 Å
Temperature	100(2) K
Theta range for data collection	2.144 to 26.420°.
Index ranges	-19<=h<=19, -16<=k<=16, -22<=l<=19
Data collection software	APEX3 (Bruker AXS Inc., 2015)[15]
Cell refinement software	SAINT V8.37A (Bruker AXS Inc., 2015)[16]
Data reduction software	SAINT V8.37A (Bruker AXS Inc., 2015)

Solution and refinement:

Reflections collected	40409
Independent reflections	13945 [R(int) = 0.0372]
Completeness to theta = 25.242°	99.9 %
Observed reflections	12300[$I > 2\sigma(I)$]
Reflections used for refinement	13945
Absorption correction	Semi-empirical from equivalents[17]
Max. and min. transmission	0.99 and 0.91
Flack parameter (absolute struct.)	0.01(2)[22]
Largest diff. peak and hole	0.241 and -0.264 e.$Å^{-3}$
Solution	Direct methods
Refinement	Full-matrix least-squares on F^2
Treatment of hydrogen atoms	CH calculated positions, constr. ref., NH located, isotr. ref.
Programs used	XT V2014/1 (Bruker AXS Inc., 2014)[18]
	SHELXL-2014/7 (Sheldrick, 2014)[19]
	DIAMOND (Crystal Impact[20]
	ShelXle (Hübschle, Sheldrick, Dittrich, 2011)[21]
Data / restraints / parameters	13945 / 1 / 853
Goodness-of-fit on F^2	1.037
R index (all data)	wR2 = 0.0759
R index conventional [I>2sigma(I)]	R1 = 0.0353

12. NMR Spectra of New Compounds

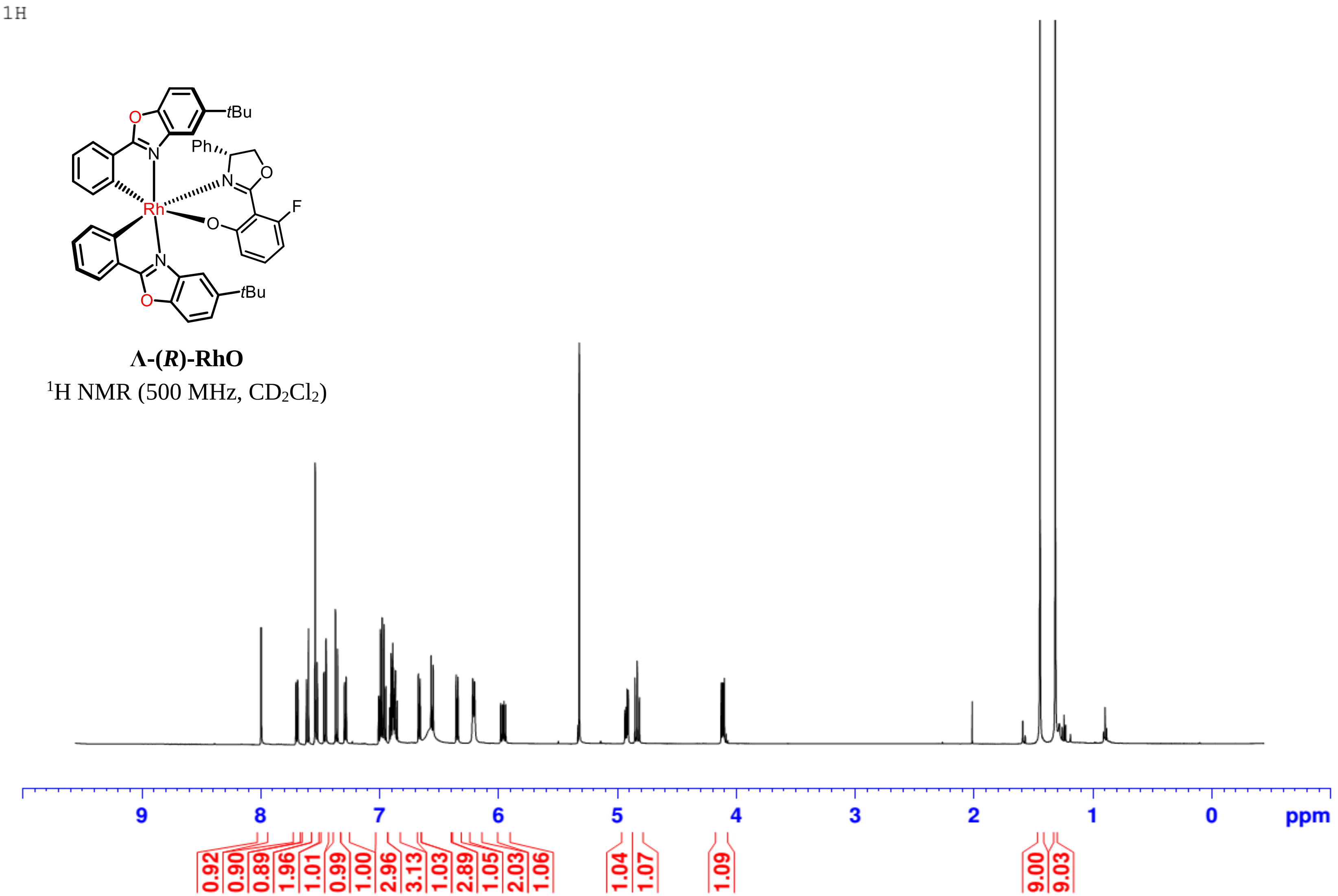

Λ-(*R*)-RhO

^{1}H NMR (500 MHz, CD_2Cl_2)

13C

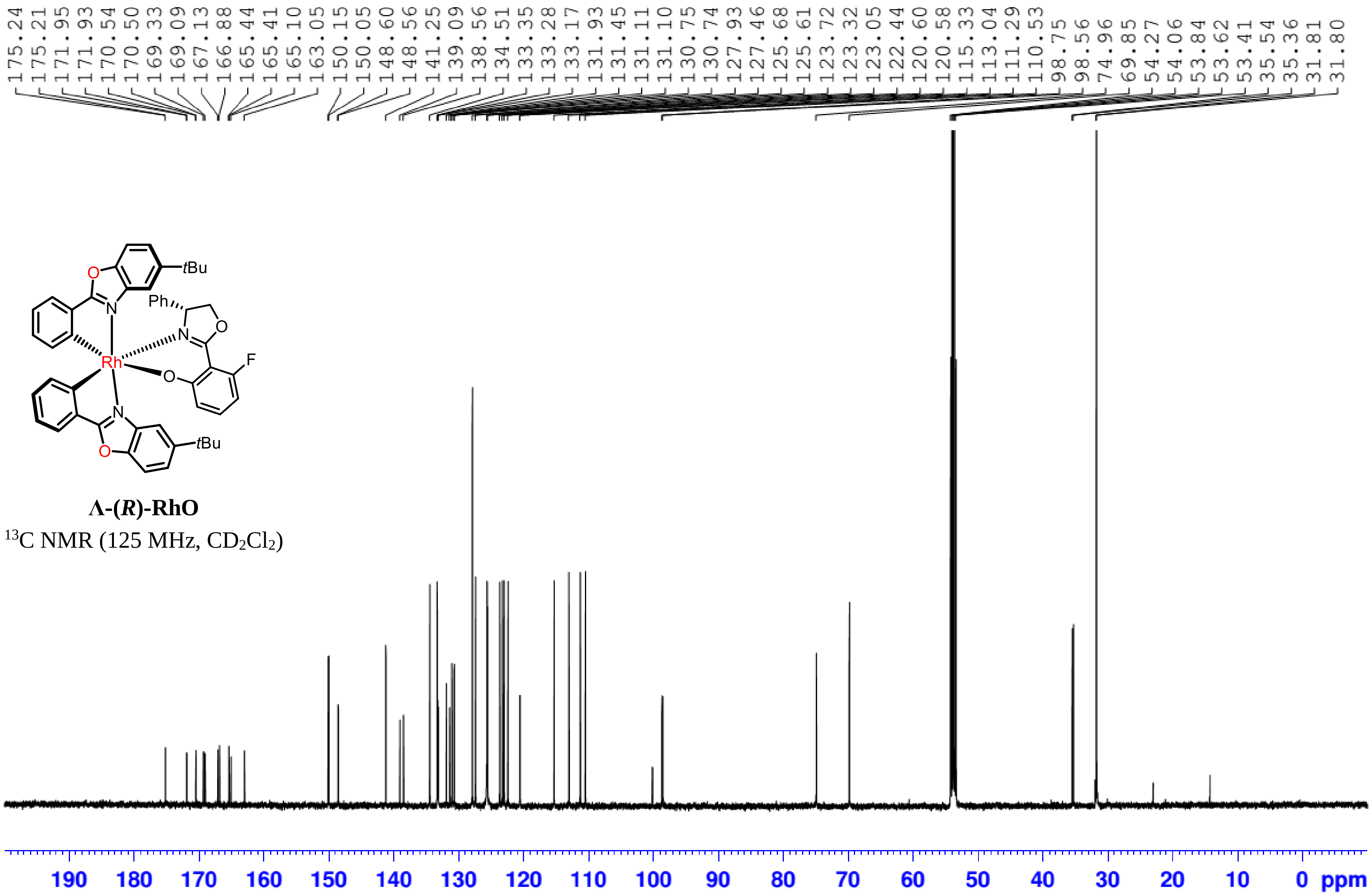

Λ-(*R*)-RhO

^{13}C NMR (125 MHz, CD_2Cl_2)

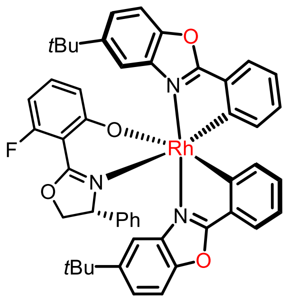

Δ-(*R*)-RhO

^{1}H NMR (500 MHz, CD_2Cl_2)

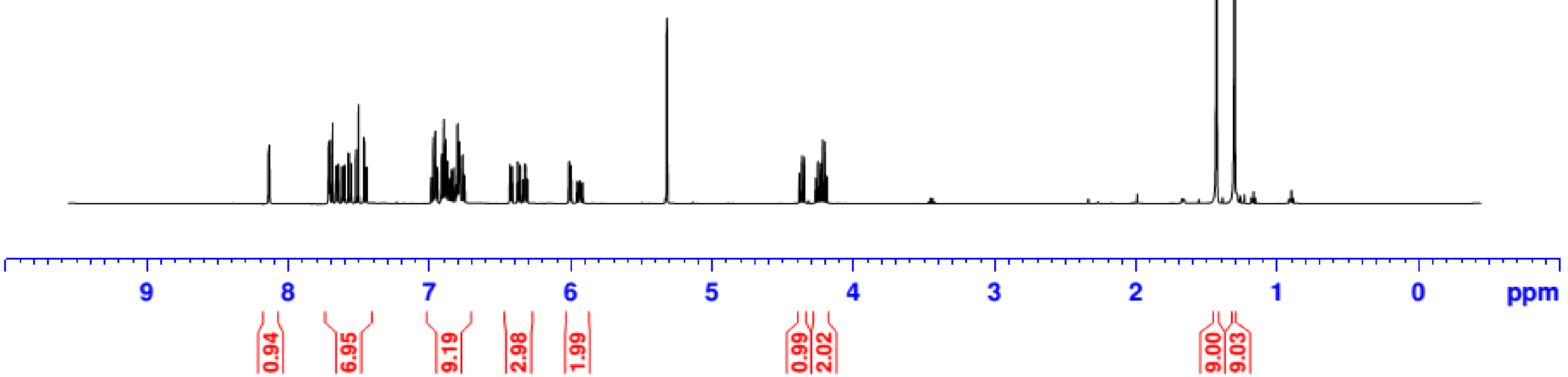

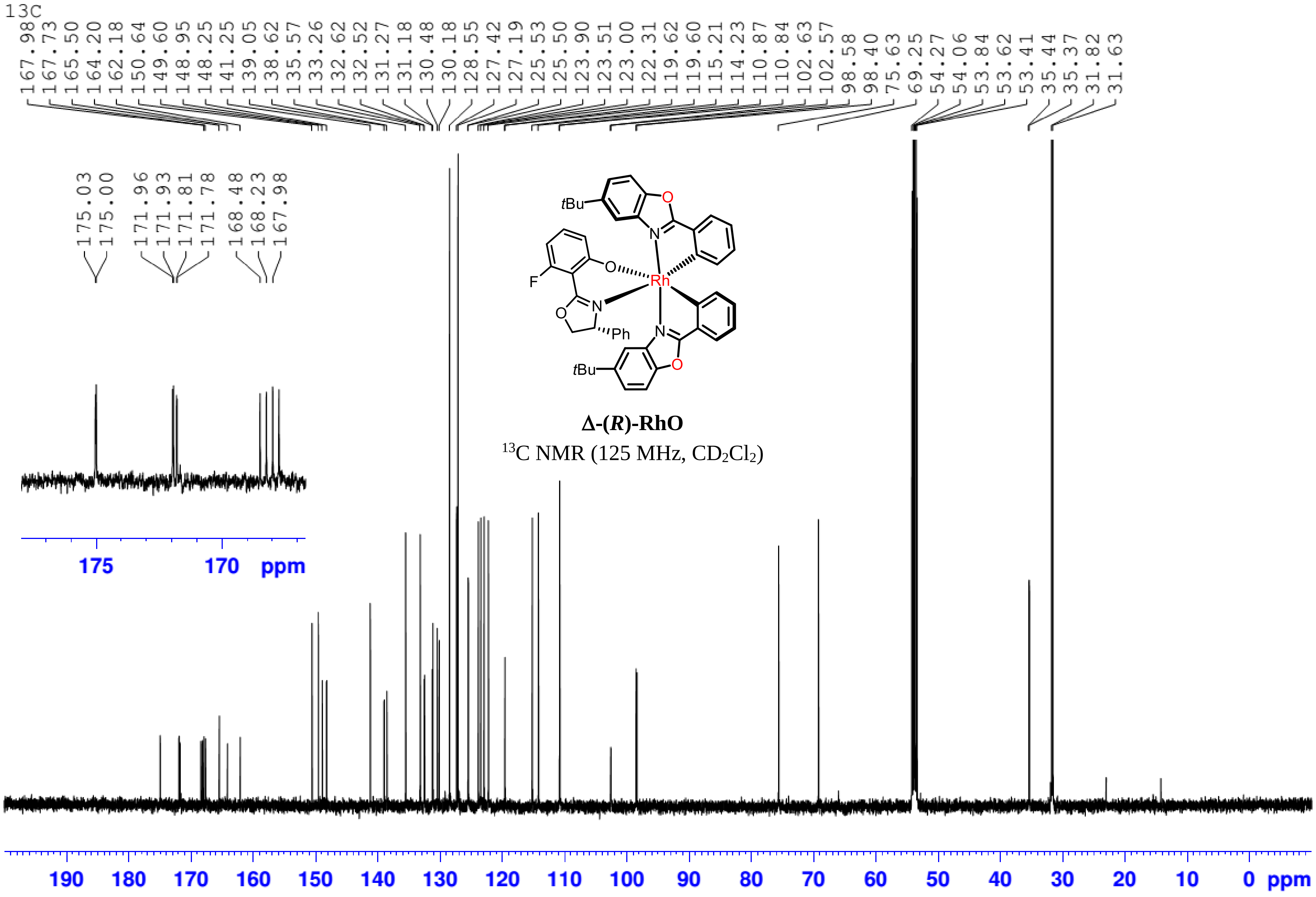

Δ-(*R*)-RhO

^{13}C NMR (125 MHz, CD_2Cl_2)

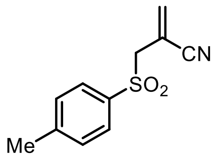

2b

^{1}H NMR (300 MHz, $CDCl_3$)

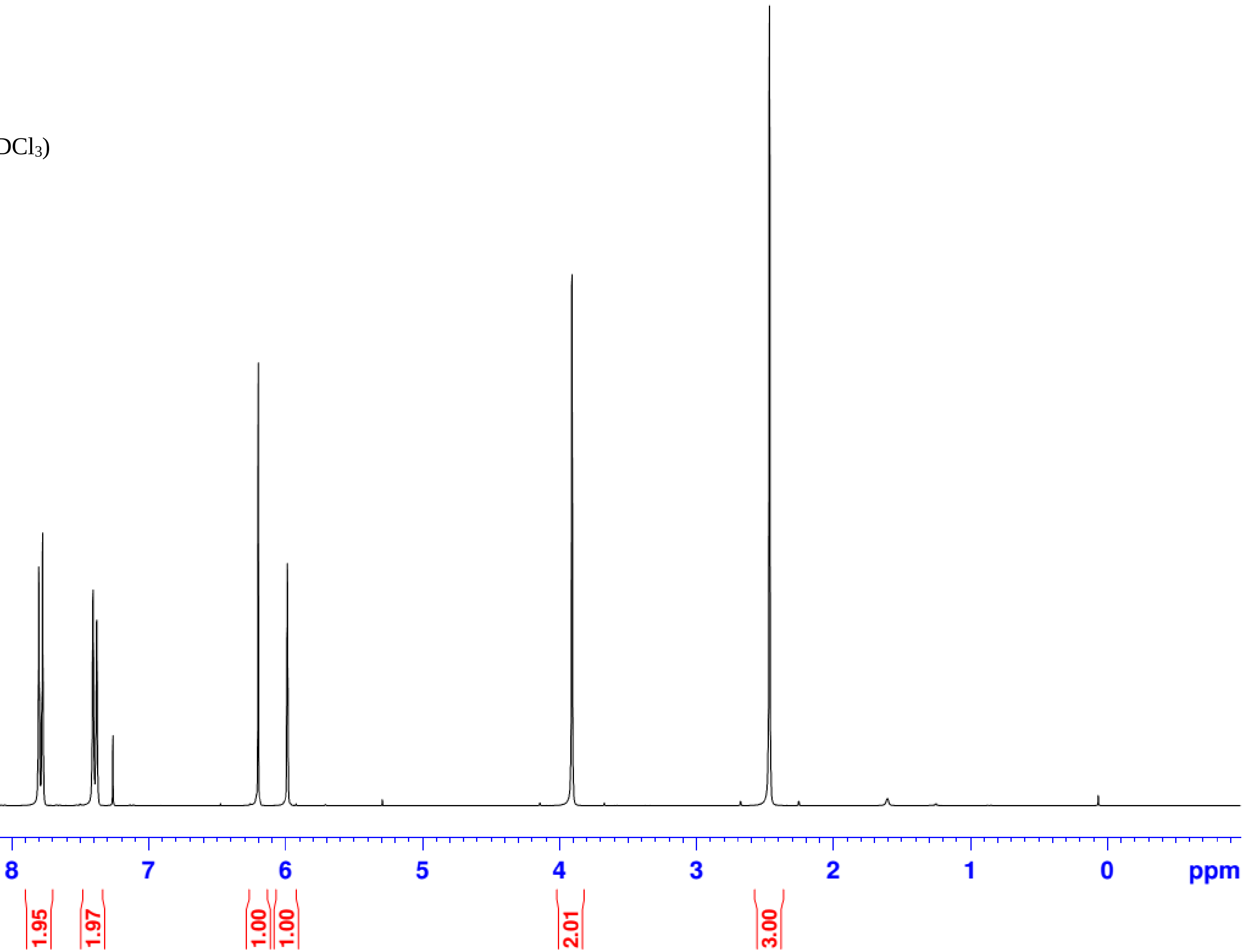

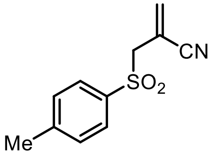

2b

^{13}C NMR (75 MHz, $CDCl_3$)

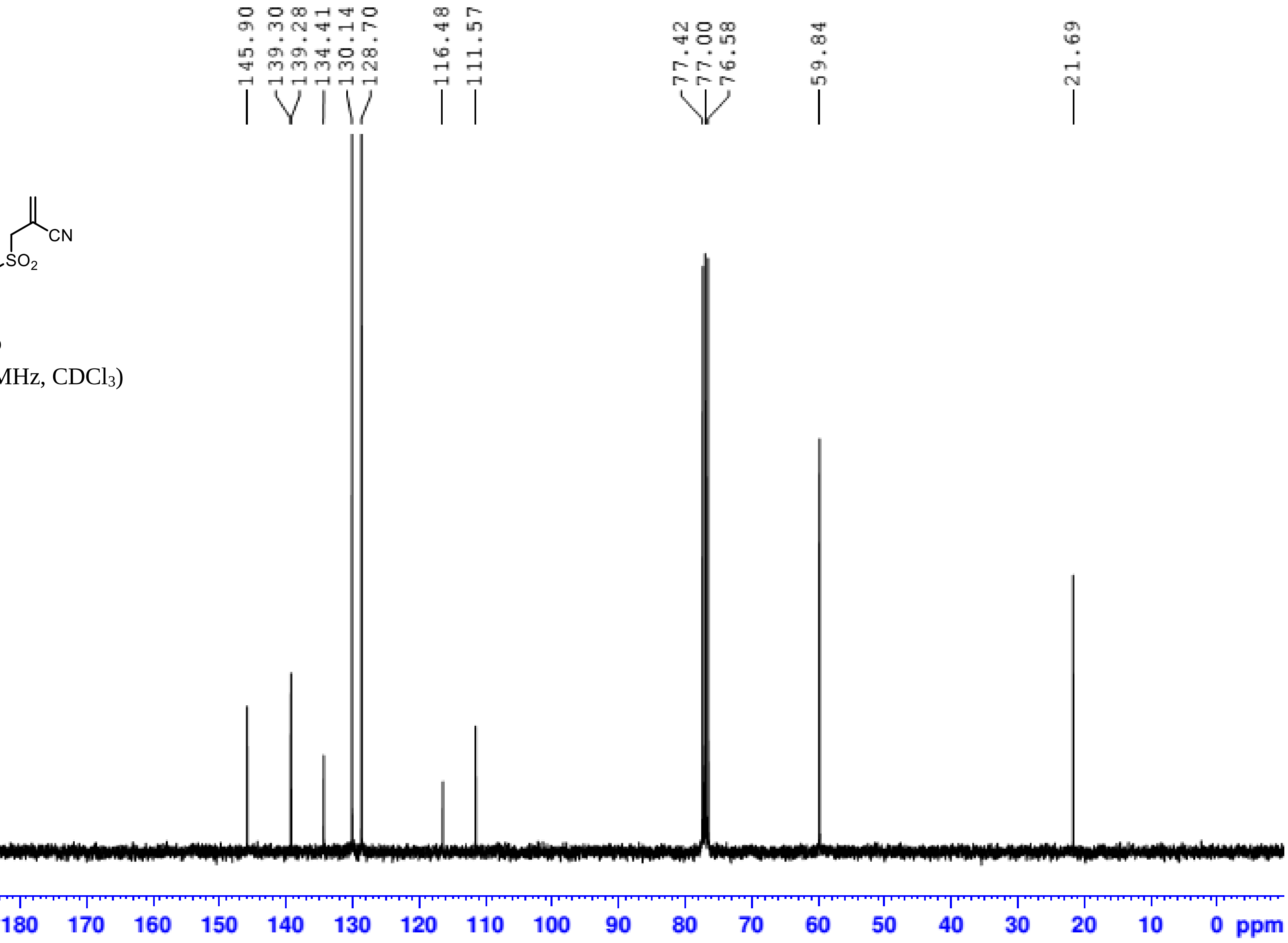

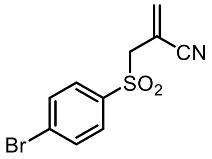

2c

^{1}H NMR (300 MHz, $CDCl_3$)

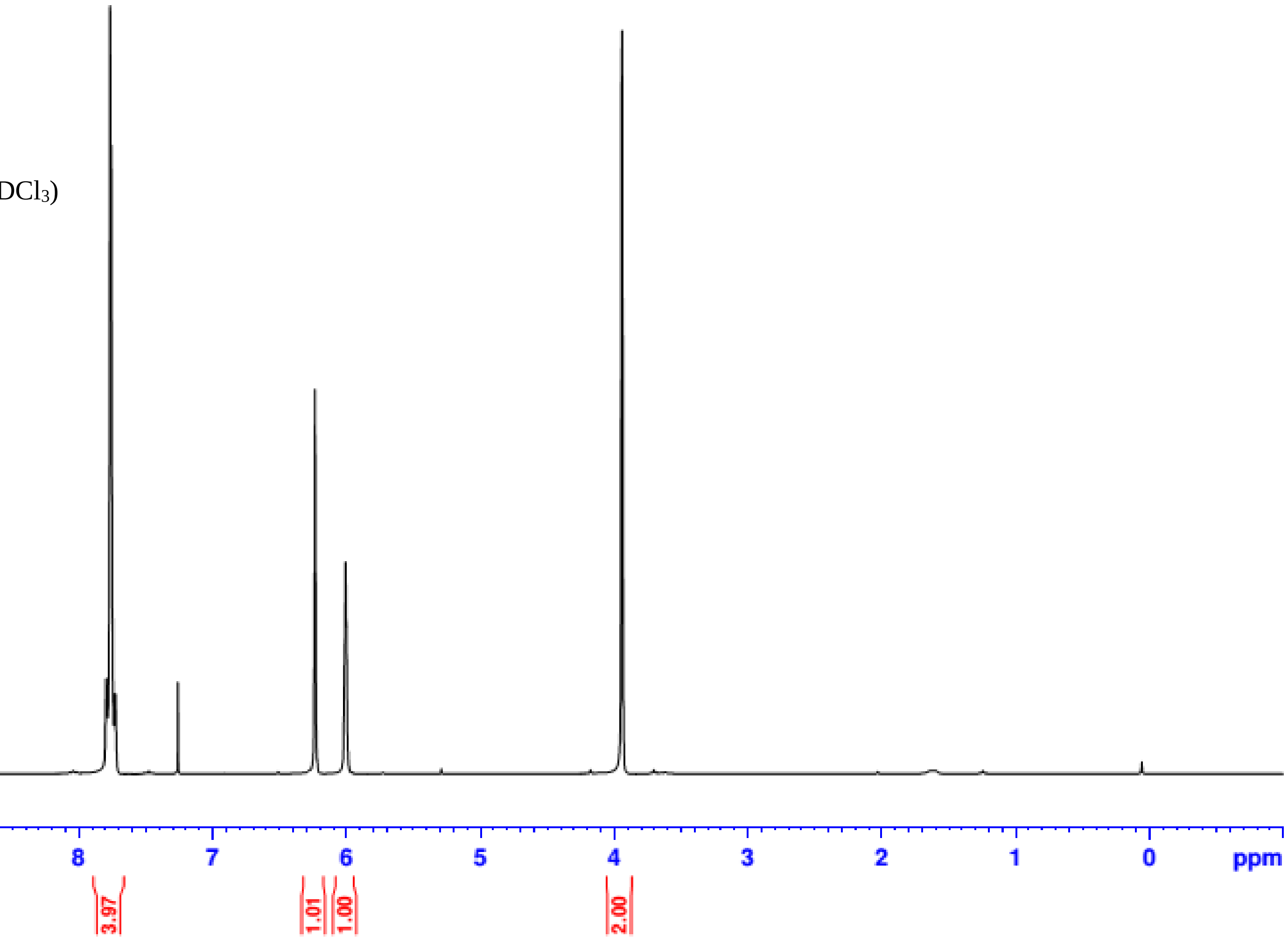

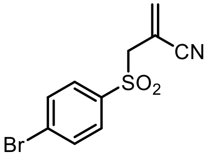

2c

^{13}C NMR (75 MHz, $CDCl_3$)

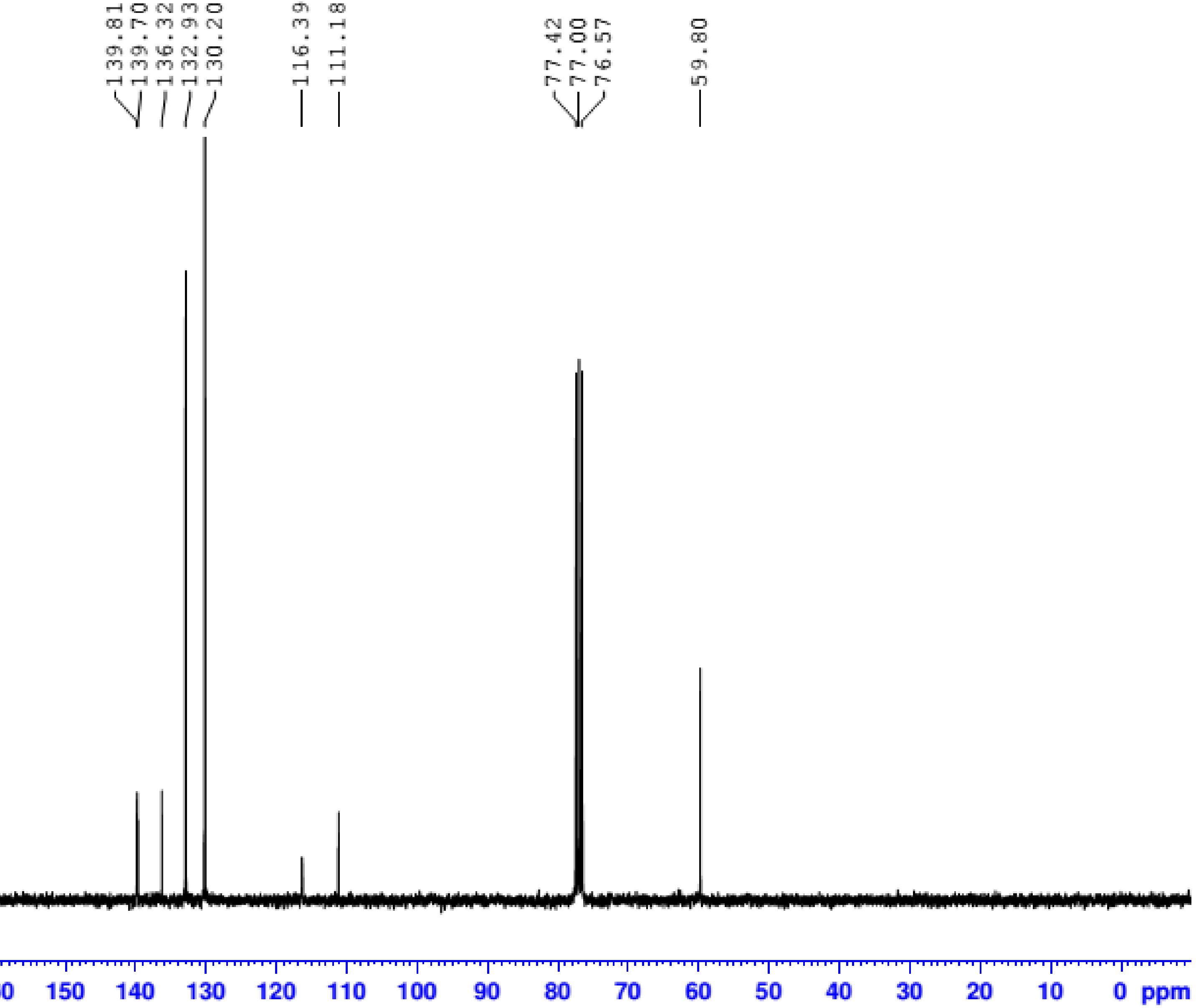

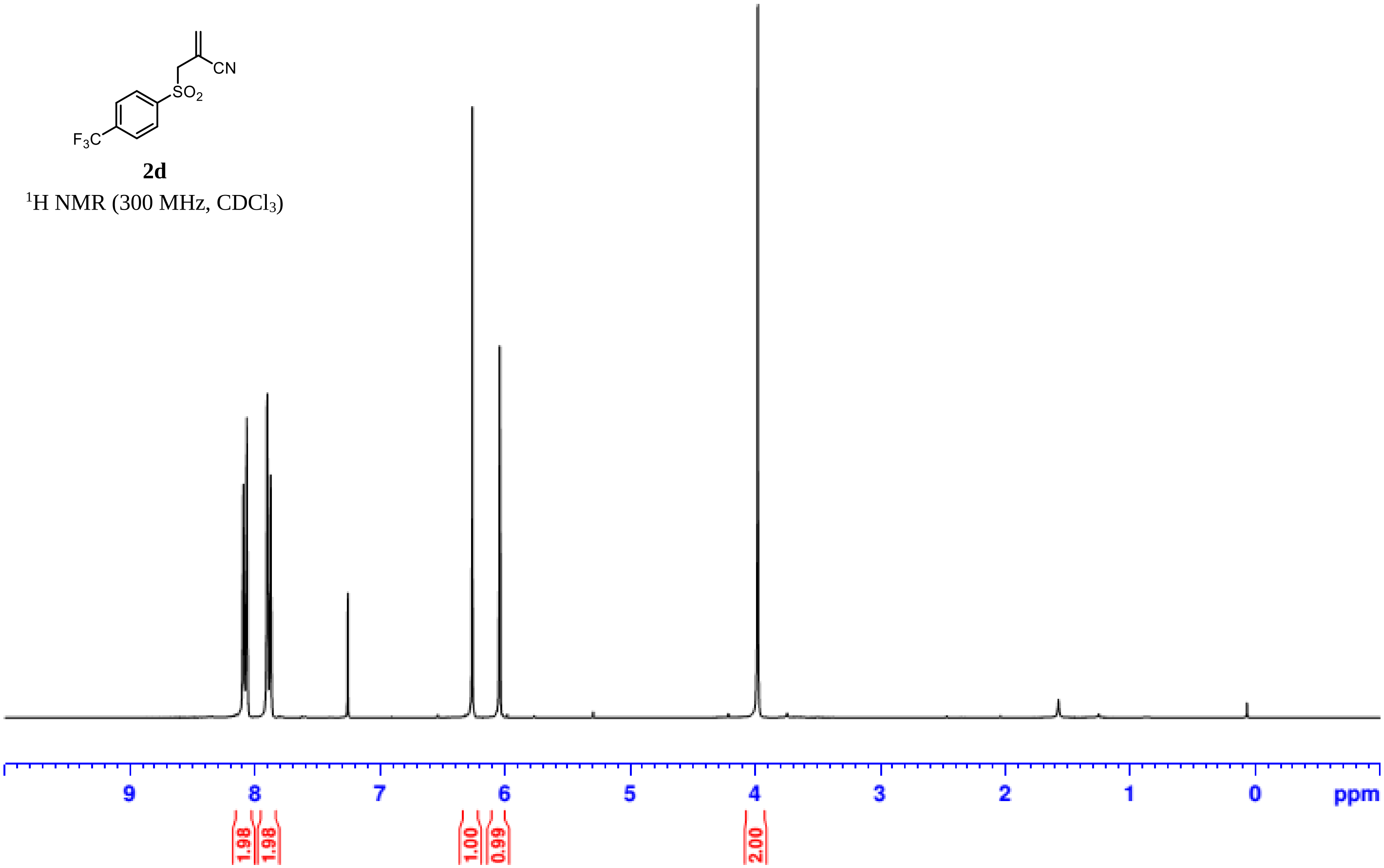

2d

^{1}H NMR (300 MHz, $CDCl_3$)

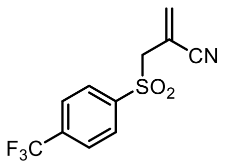

2d

^{13}C NMR (75 MHz, $CDCl_3$)

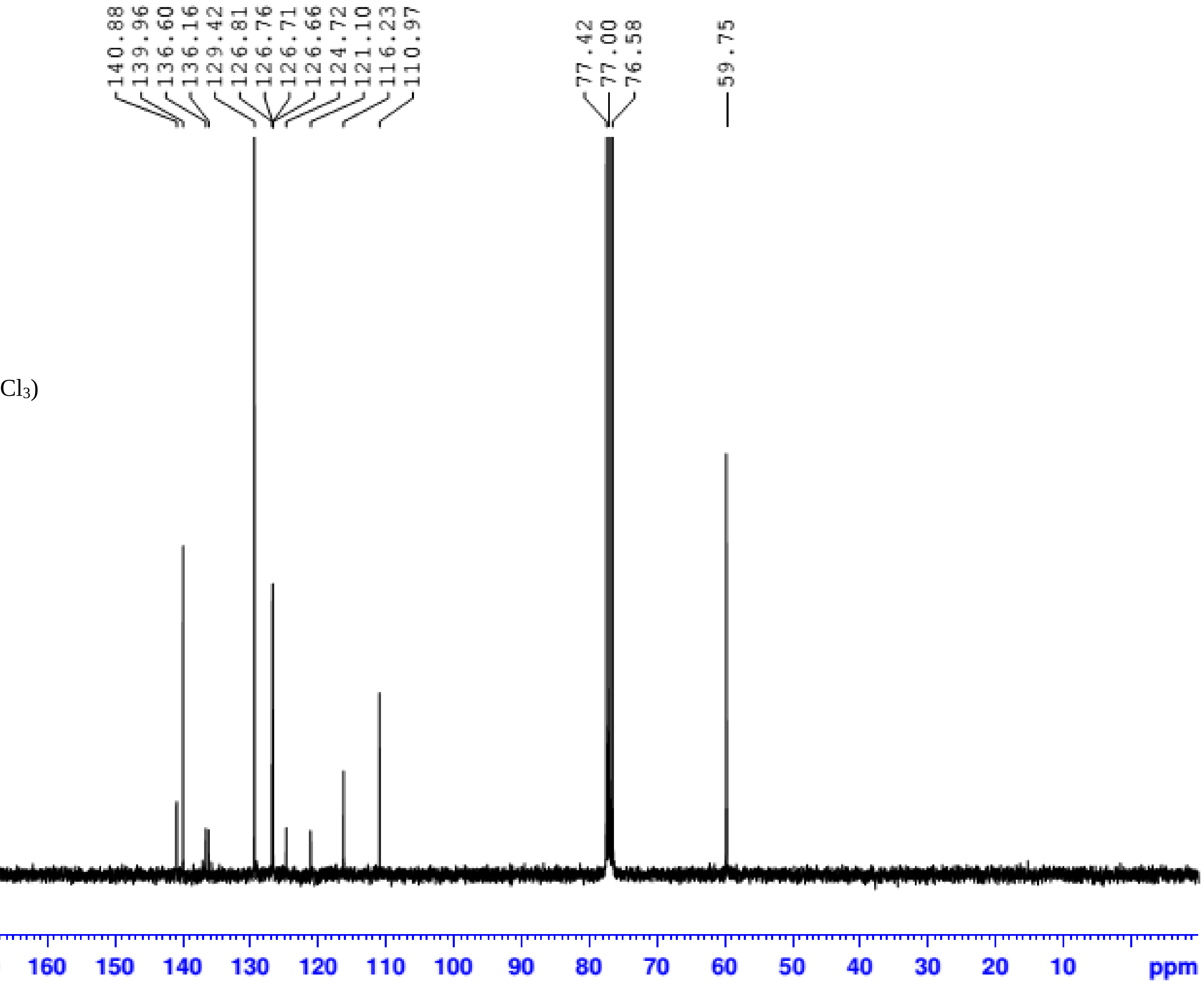

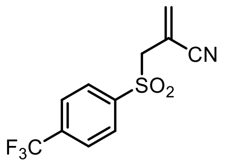

2d

^{19}F NMR (282 MHz, $CDCl_3$)

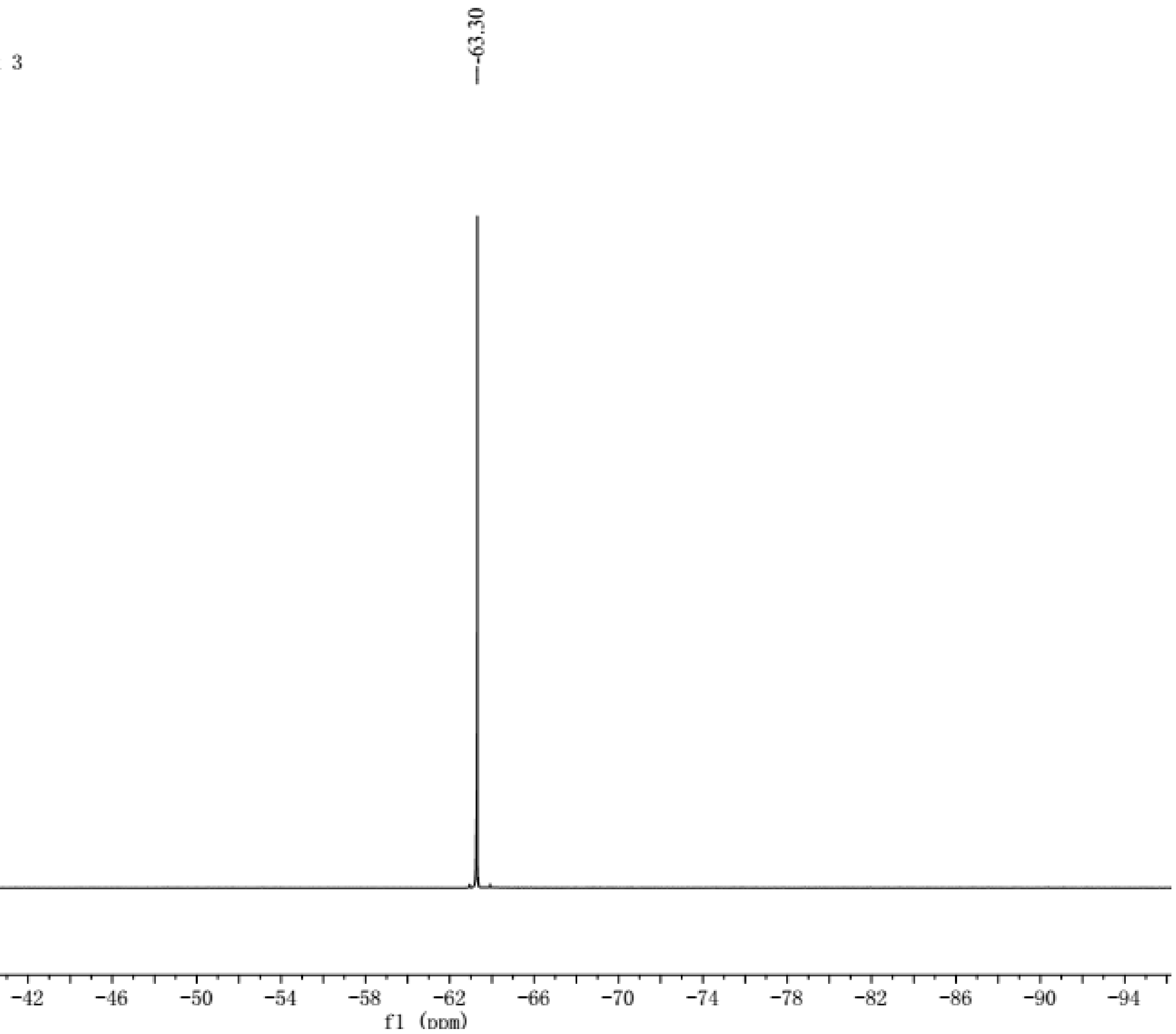

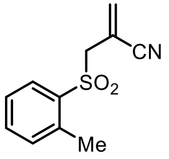

2e

^{1}H NMR (300 MHz, $CDCl_3$)

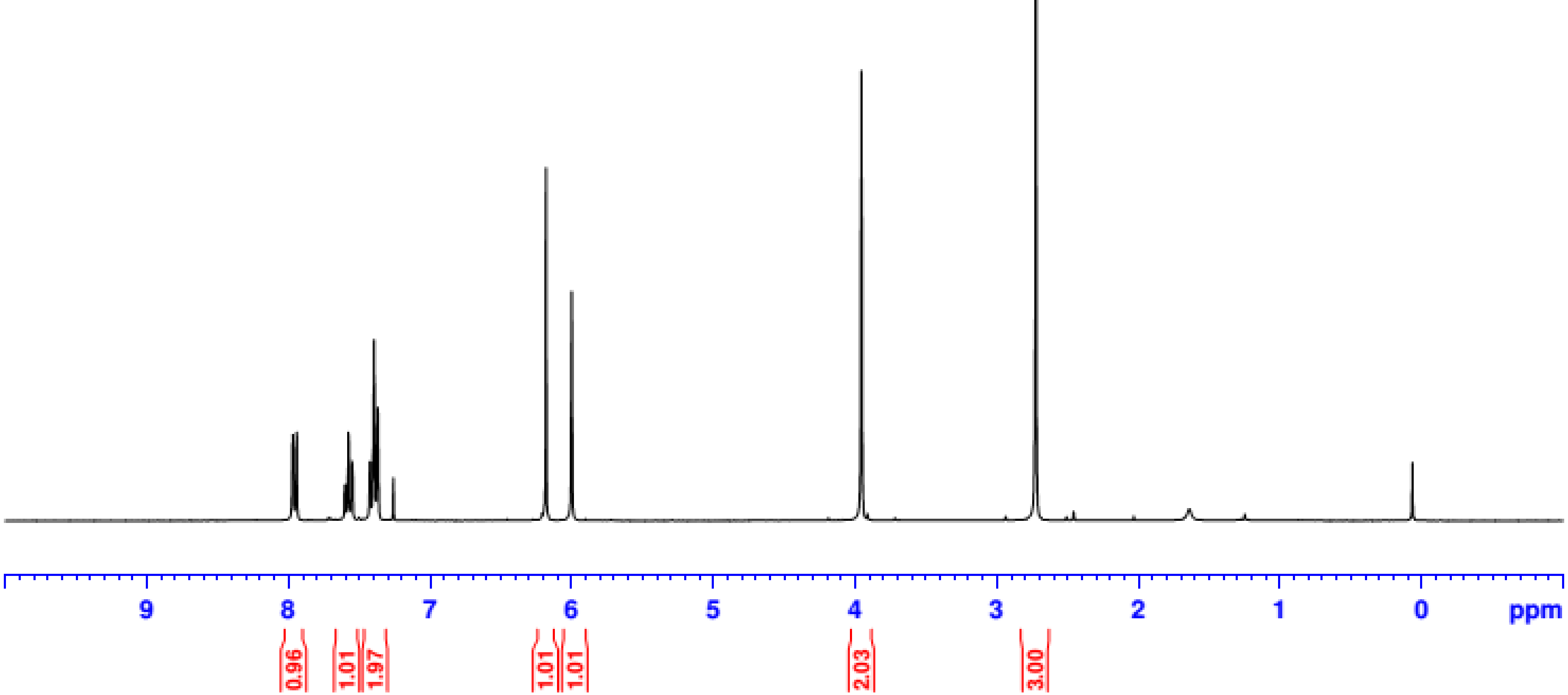

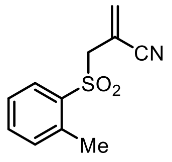

2e

^{13}C NMR (75 MHz, $CDCl_3$)

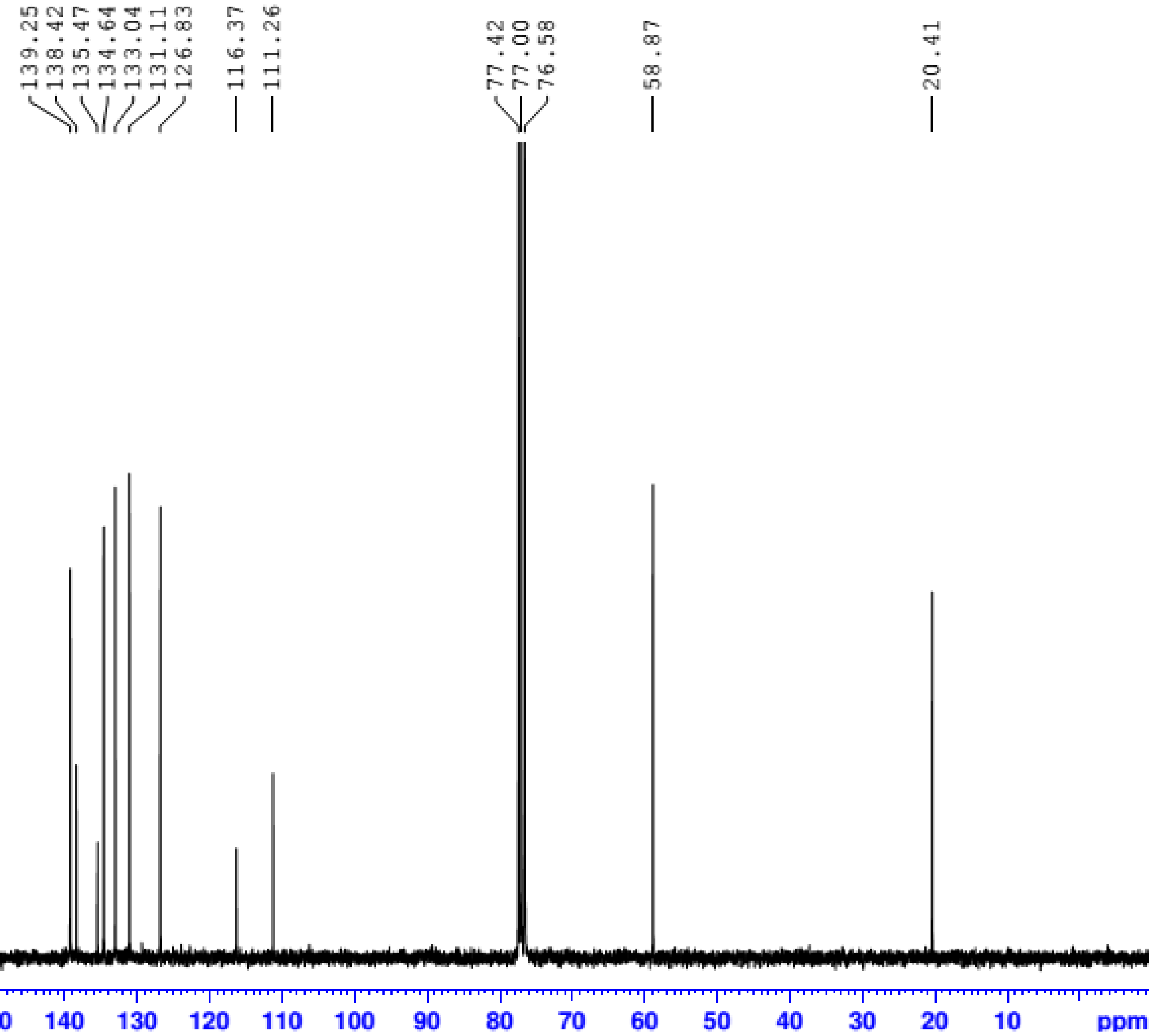

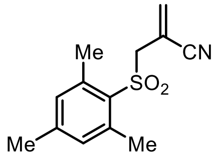

2f

^{1}H NMR (300 MHz, $CDCl_3$)

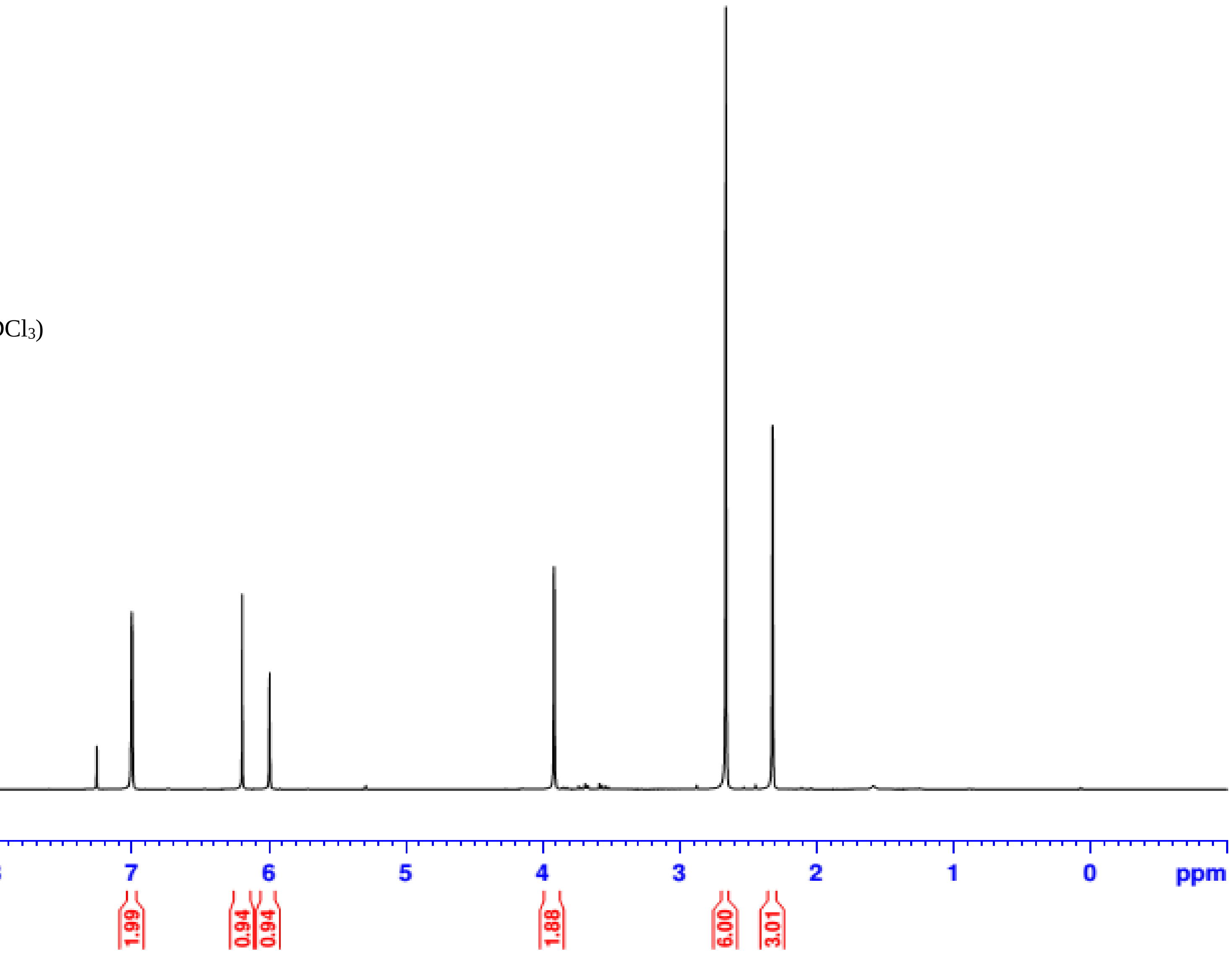

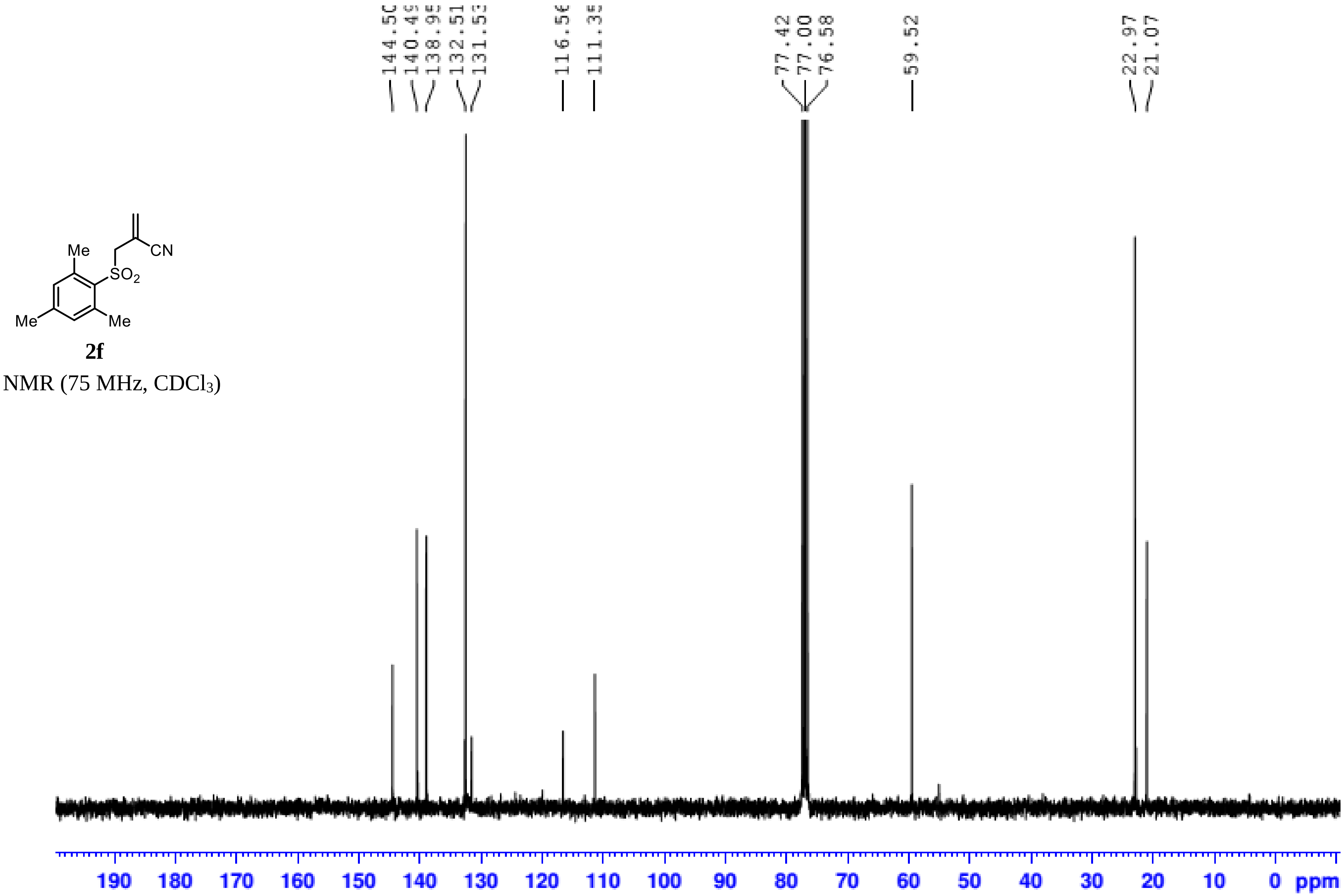

2f

^{13}C NMR (75 MHz, $CDCl_3$)

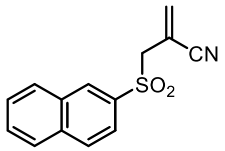

2g

^{1}H NMR (300 MHz, $CDCl_3$)

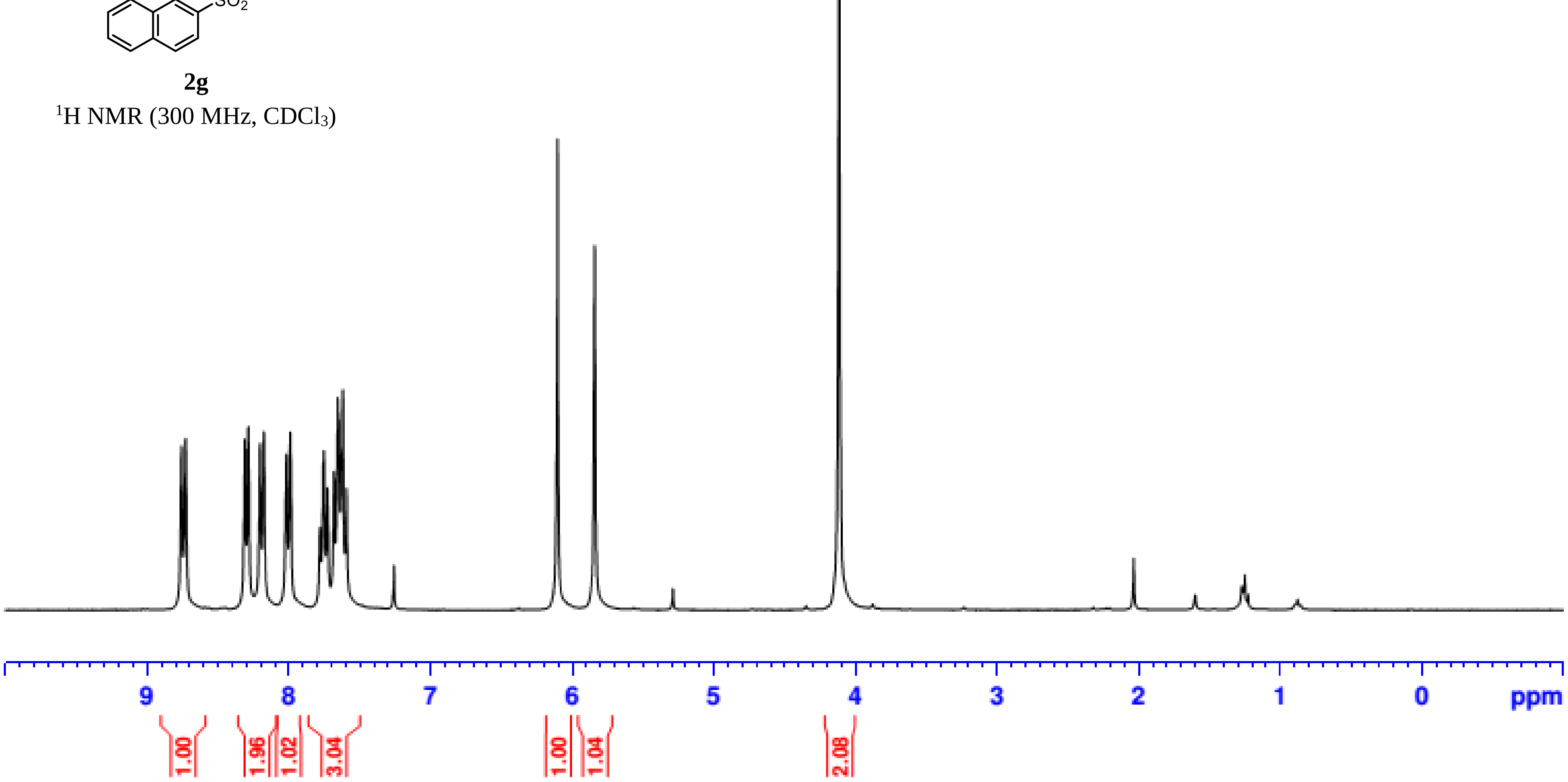

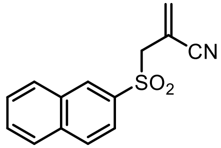

2g

^{13}C NMR (75 MHz, $CDCl_3$)

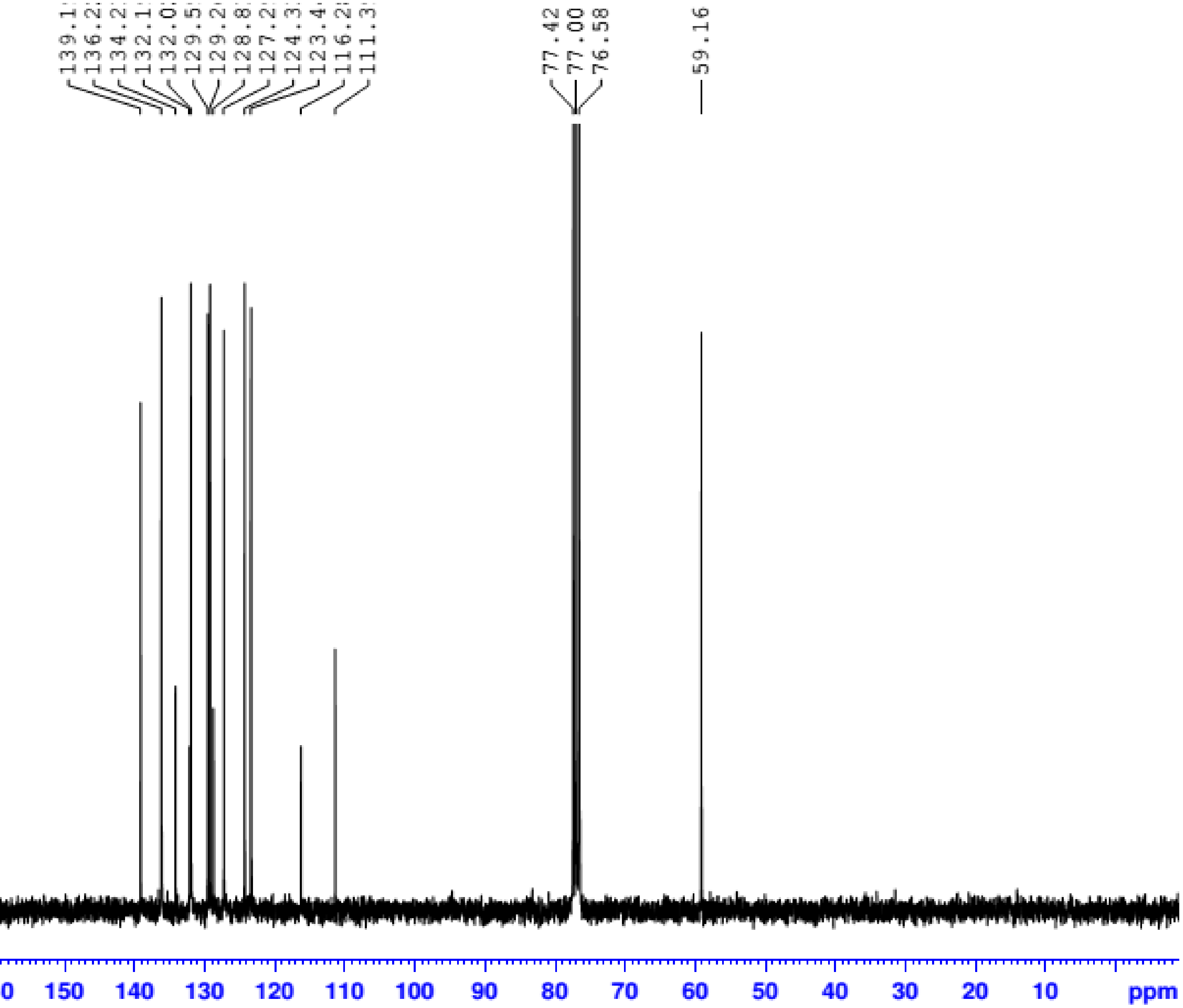

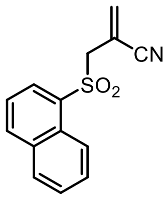

2h

^{1}H NMR (300 MHz, $CDCl_3$)

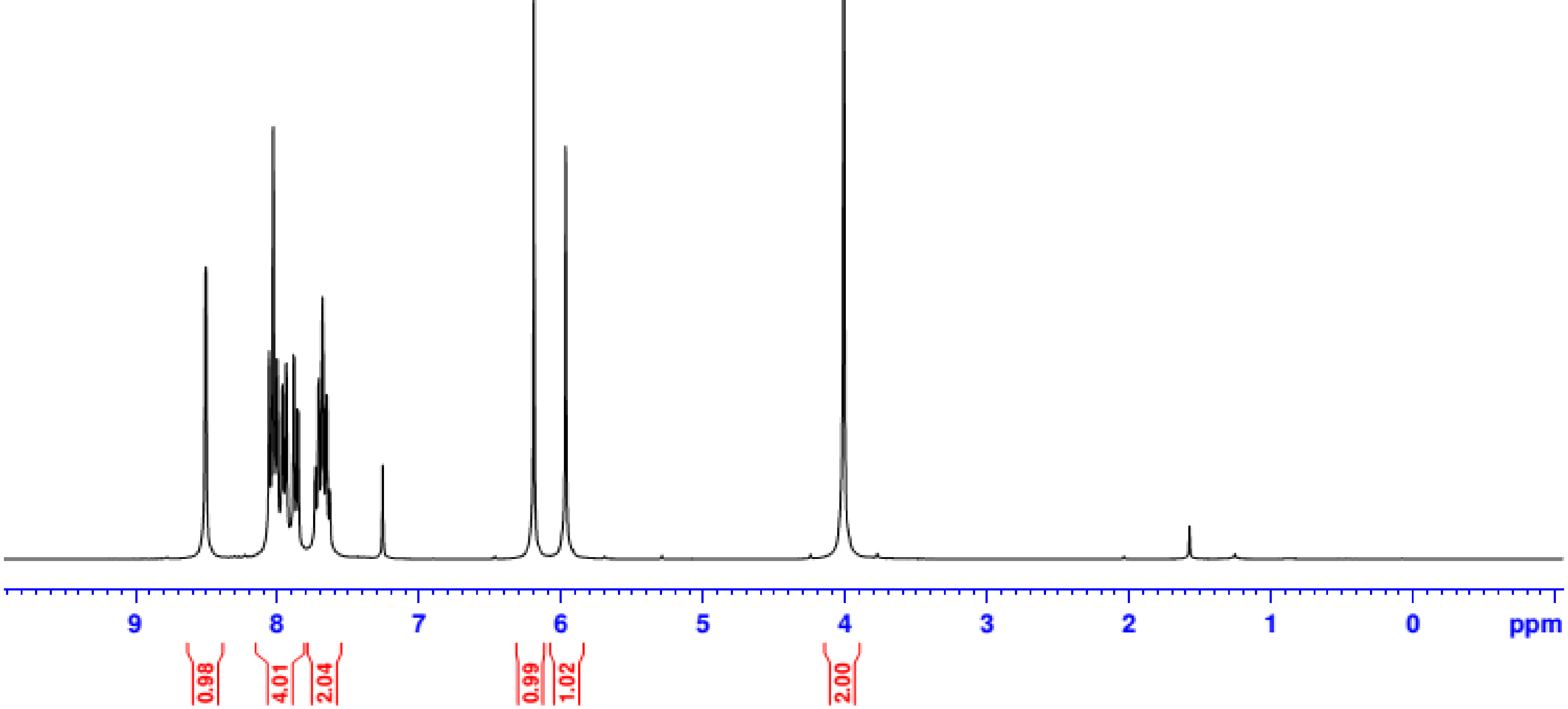

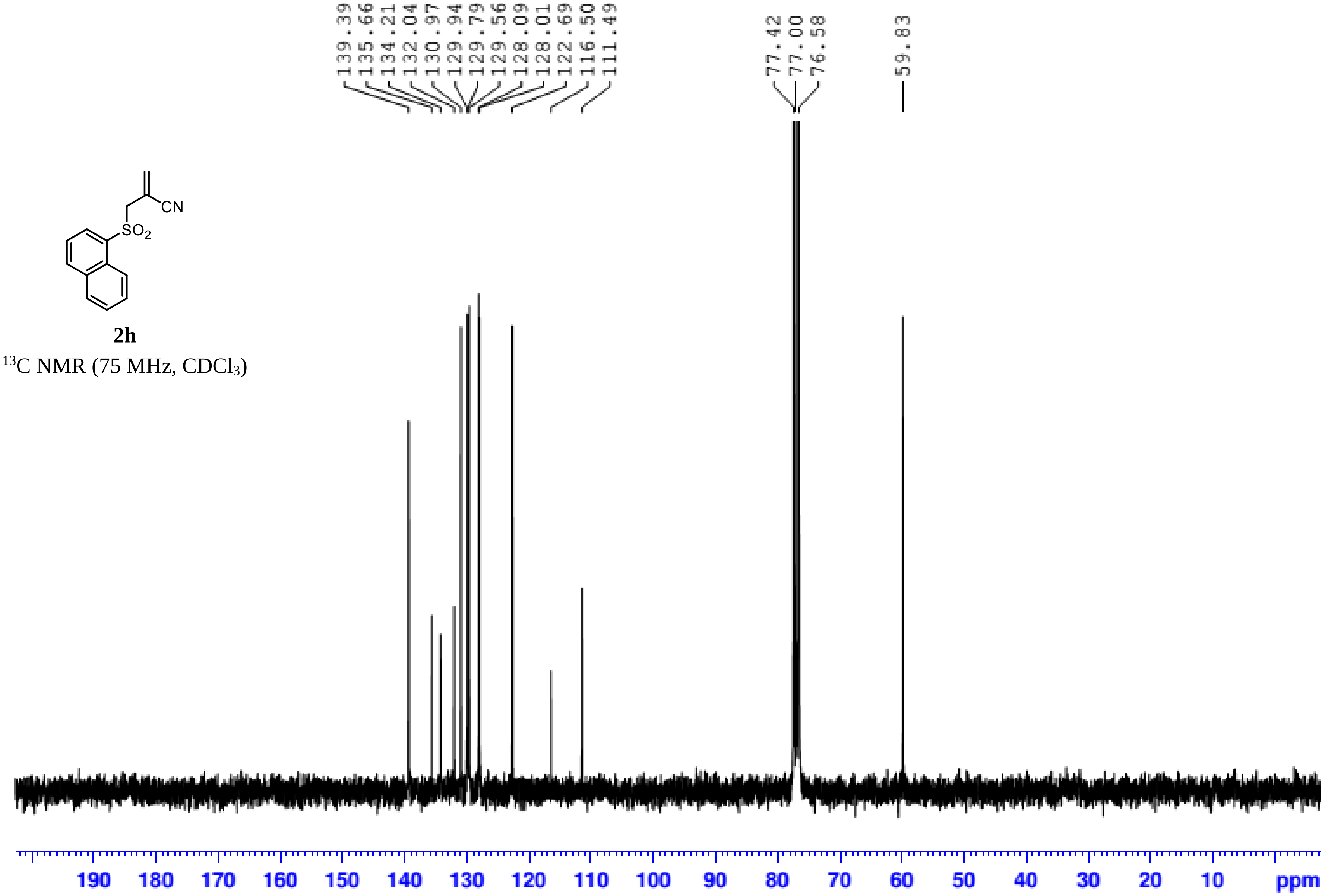

2h

^{13}C NMR (75 MHz, $CDCl_3$)

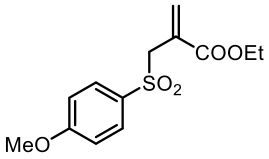

2i

^{1}H NMR (300 MHz, $CDCl_3$)

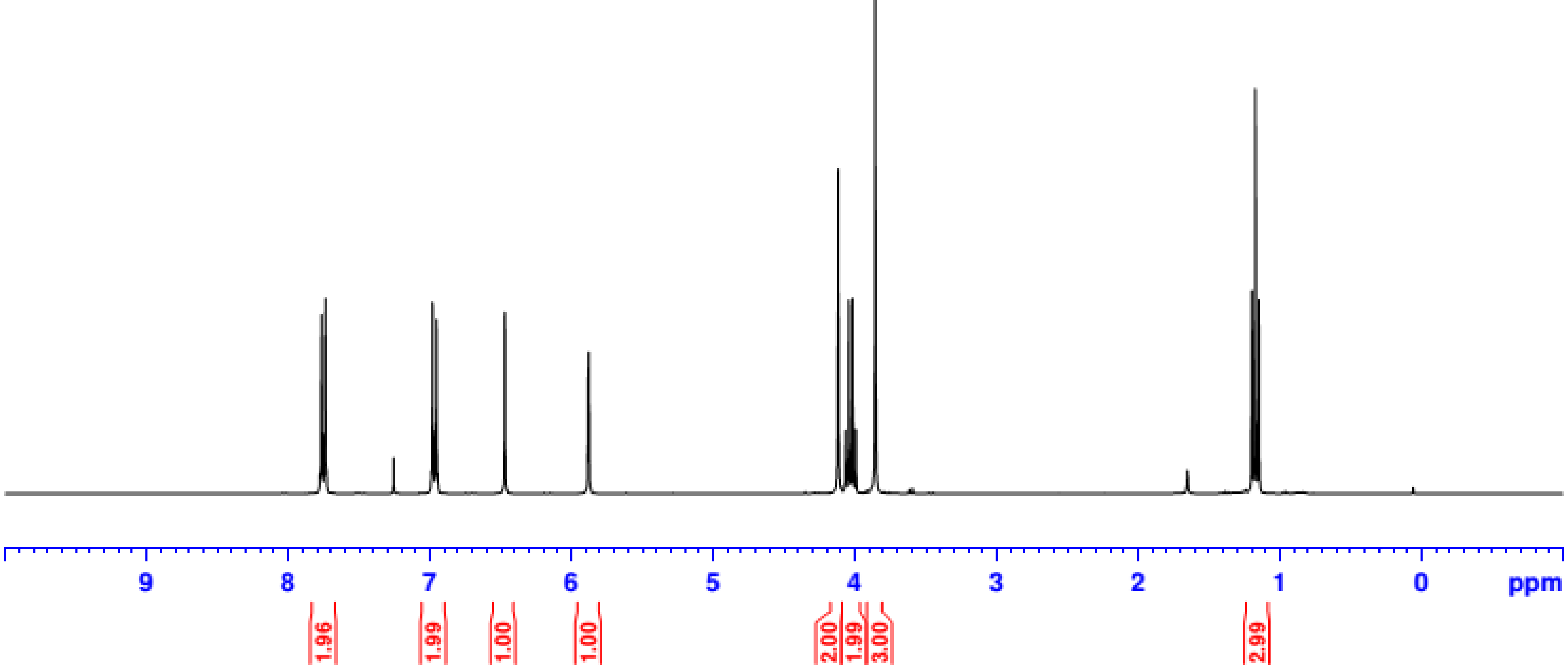

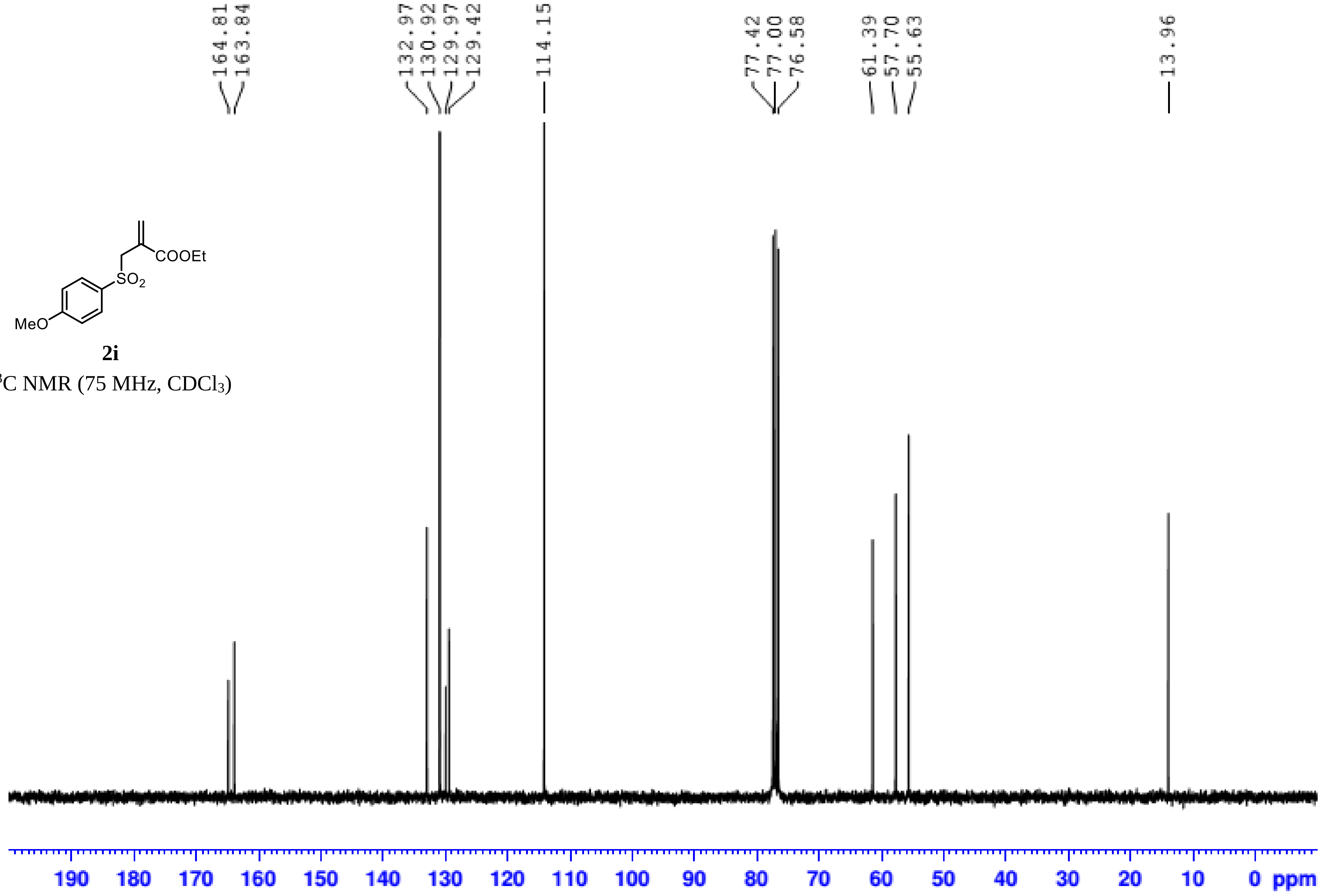

2i

^{13}C NMR (75 MHz, $CDCl_3$)

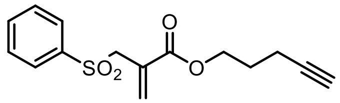

2k

^{1}H NMR (300 MHz, $CDCl_3$)

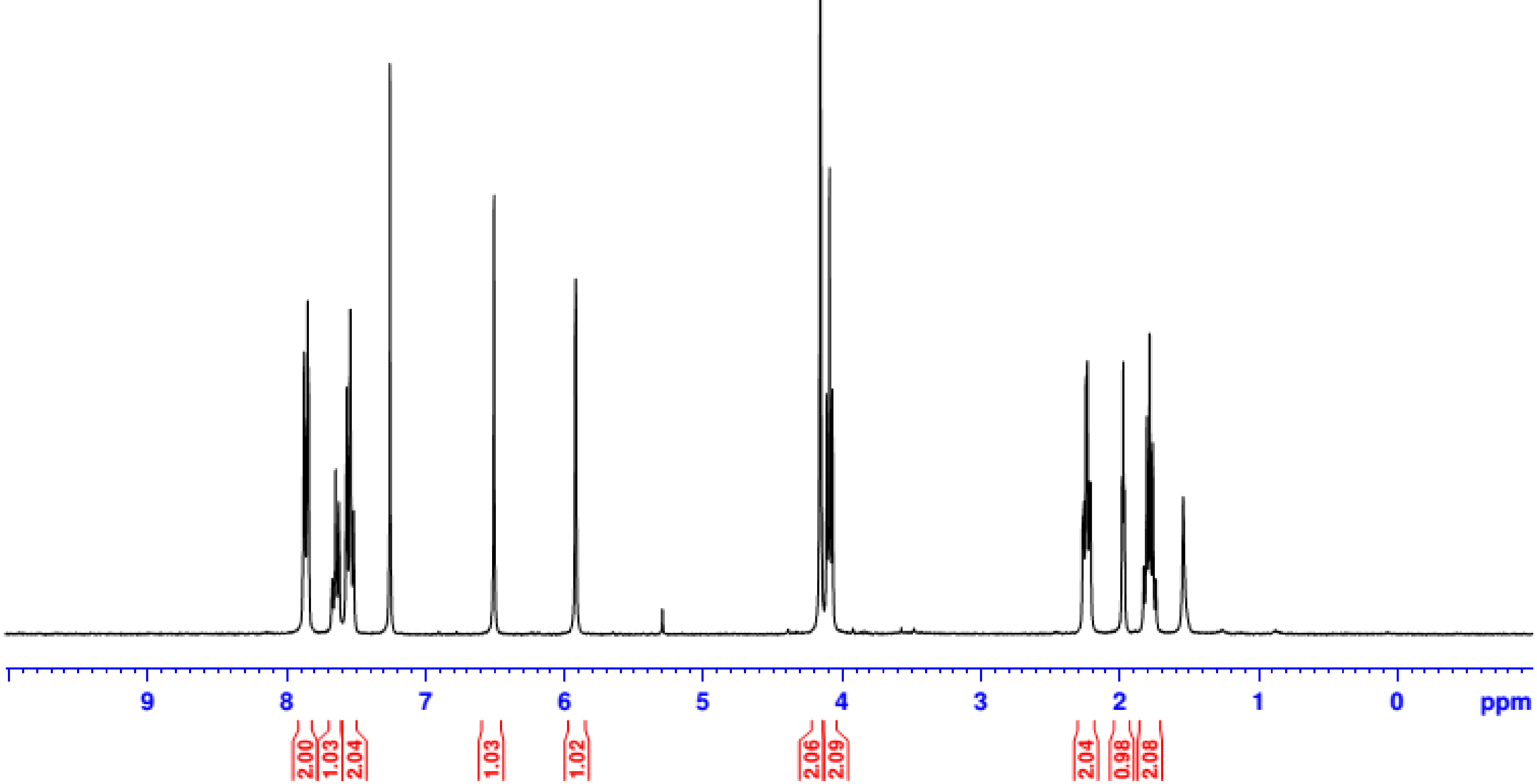

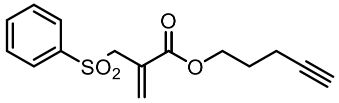

2k

^{13}C NMR (75 MHz, $CDCl_3$)

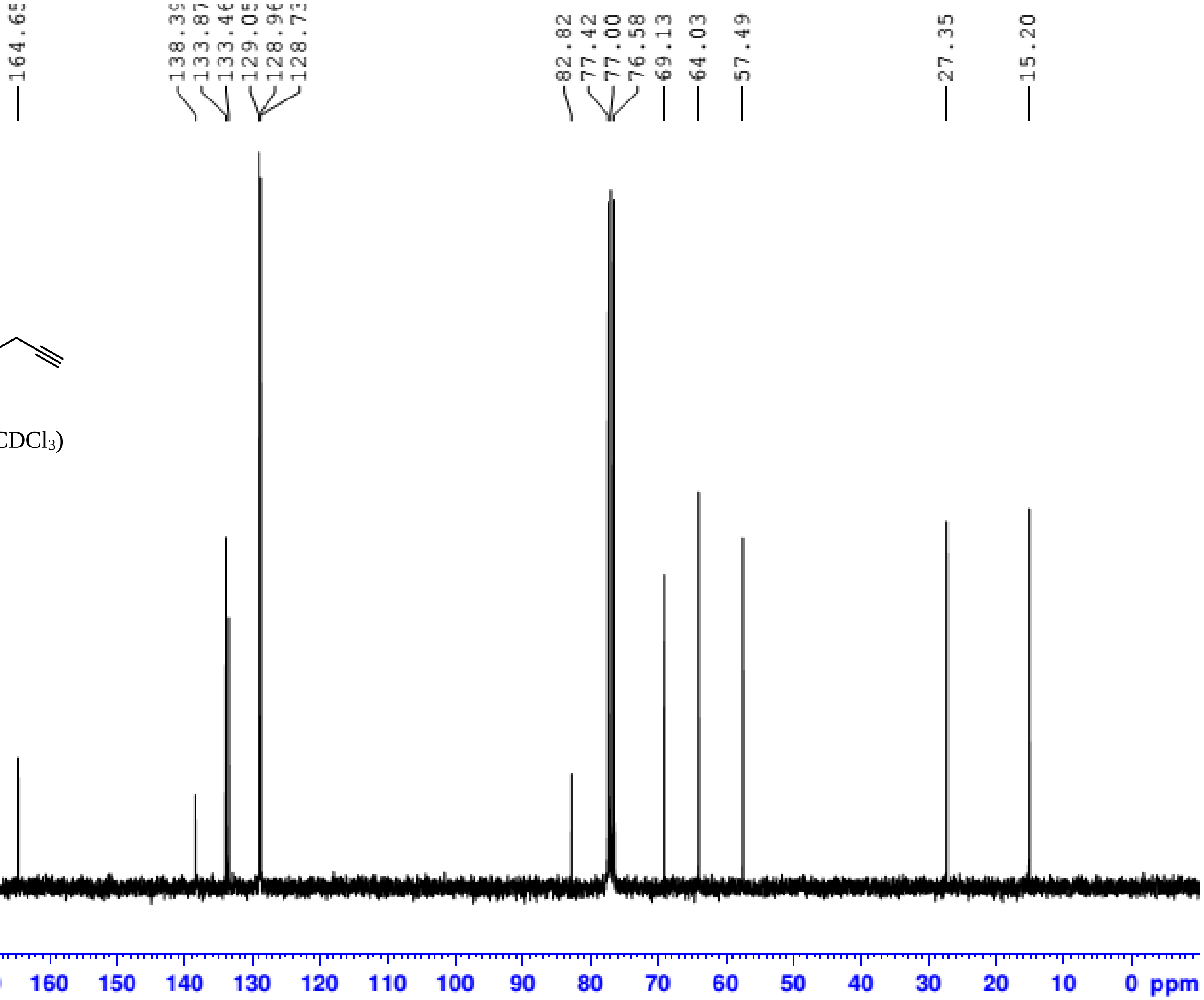

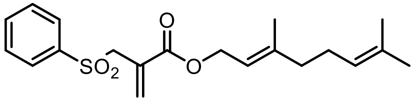

2l

^{1}H NMR (300 MHz, $CDCl_3$)

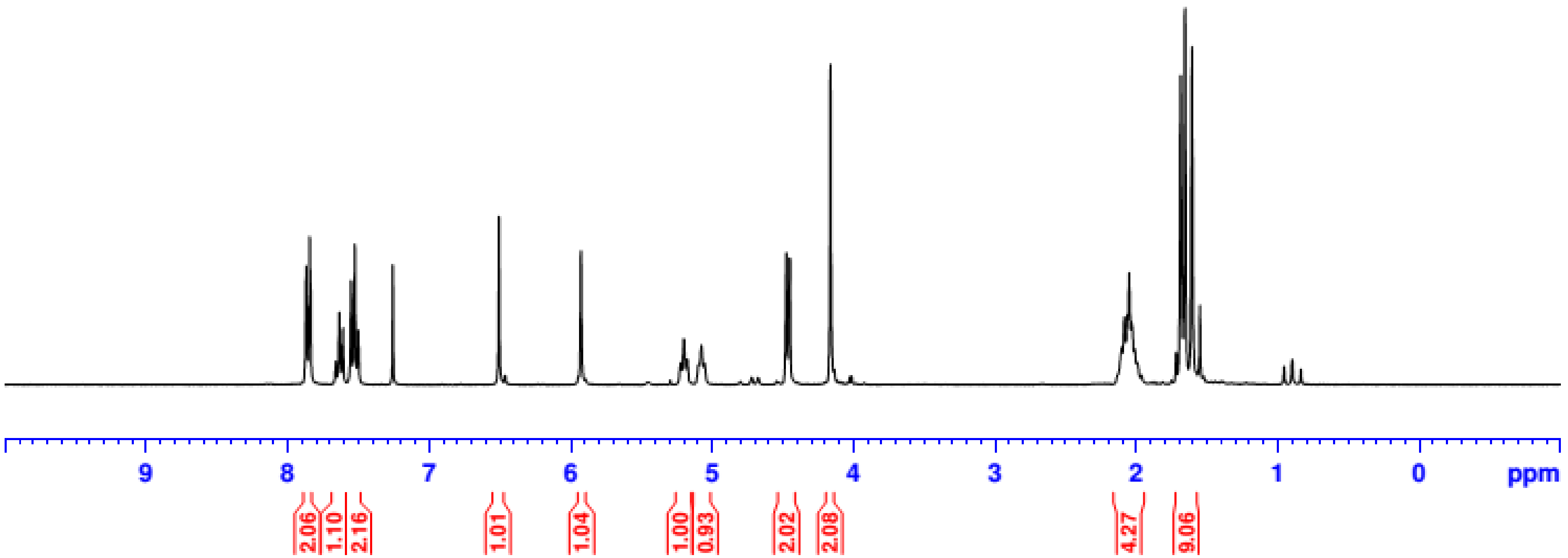

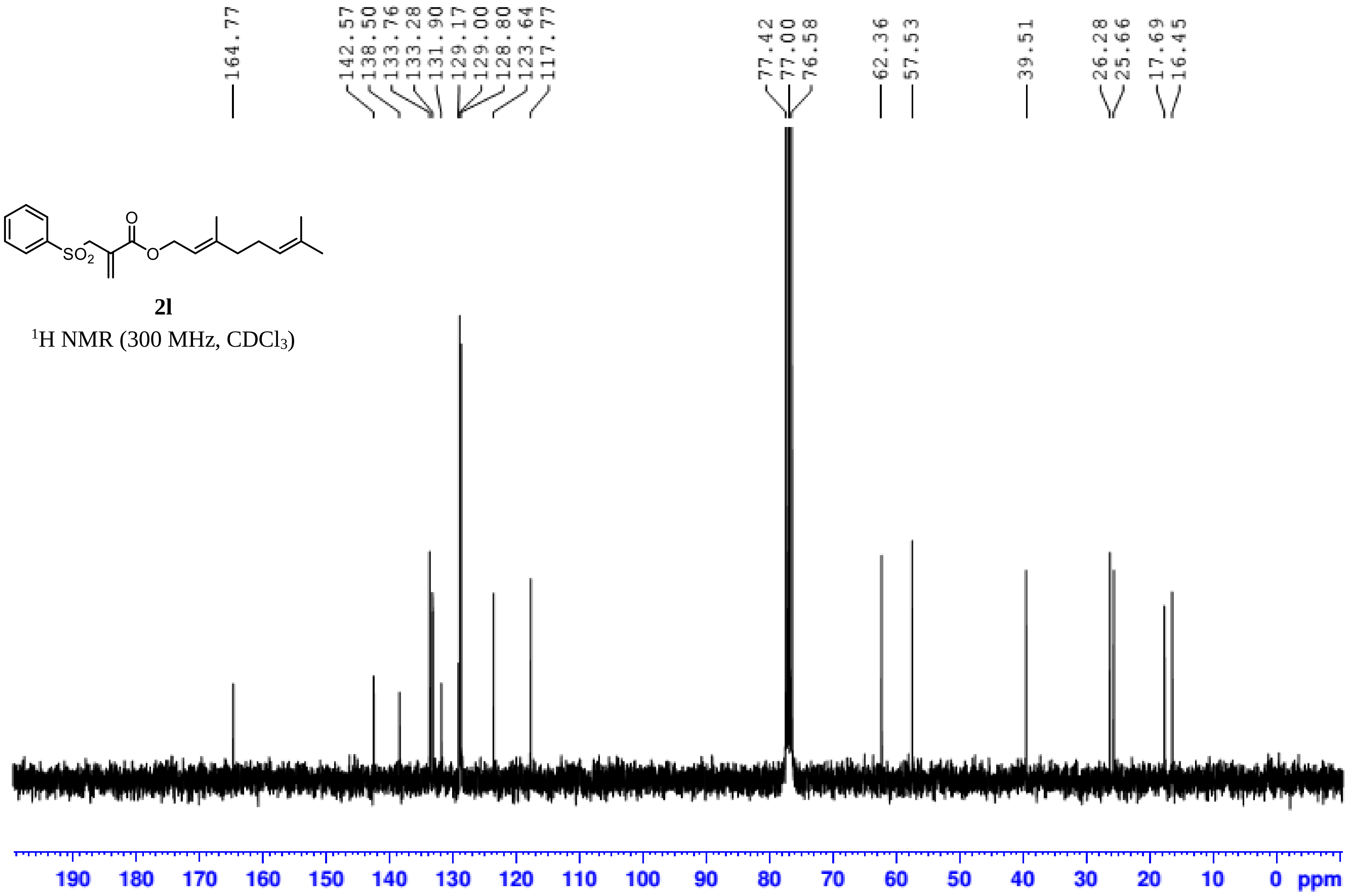

2l

^{1}H NMR (300 MHz, $CDCl_3$)

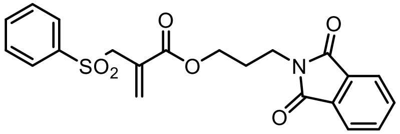

2m

^{1}H NMR (300 MHz, CDCl$_3$)

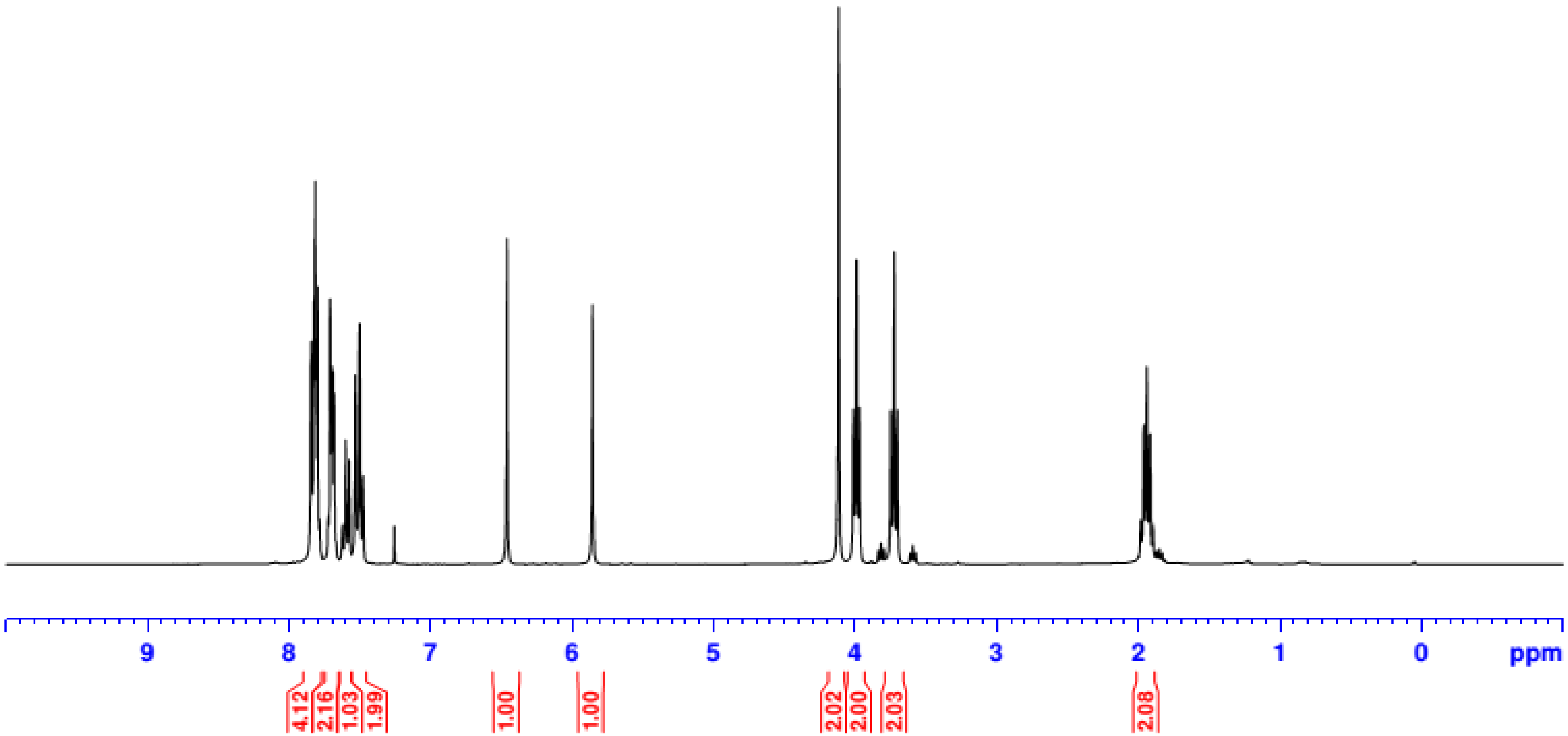

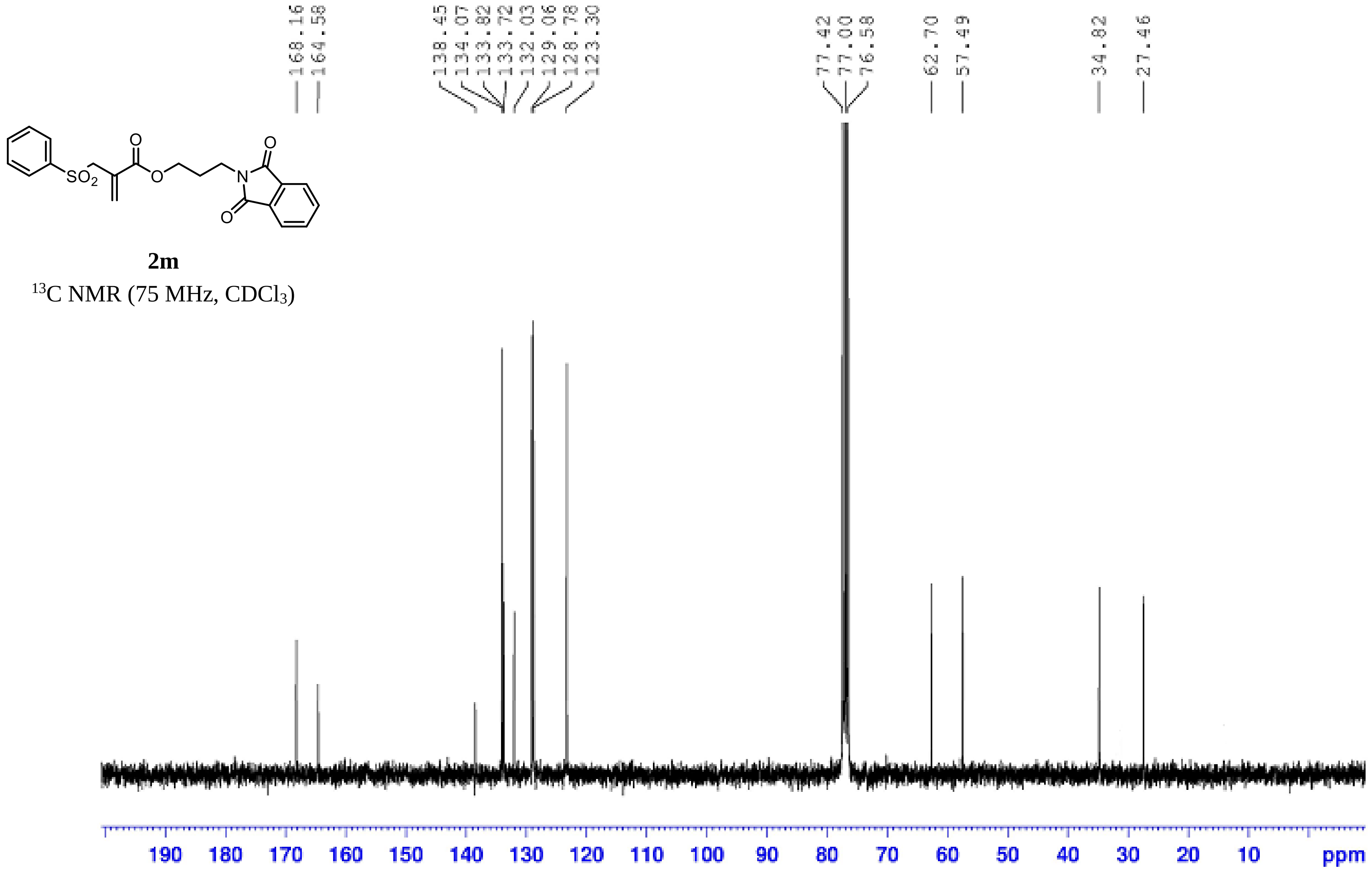

2m

^{13}C NMR (75 MHz, $CDCl_3$)

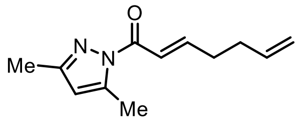

1d

^{1}H NMR (300 MHz, $CDCl_3$)

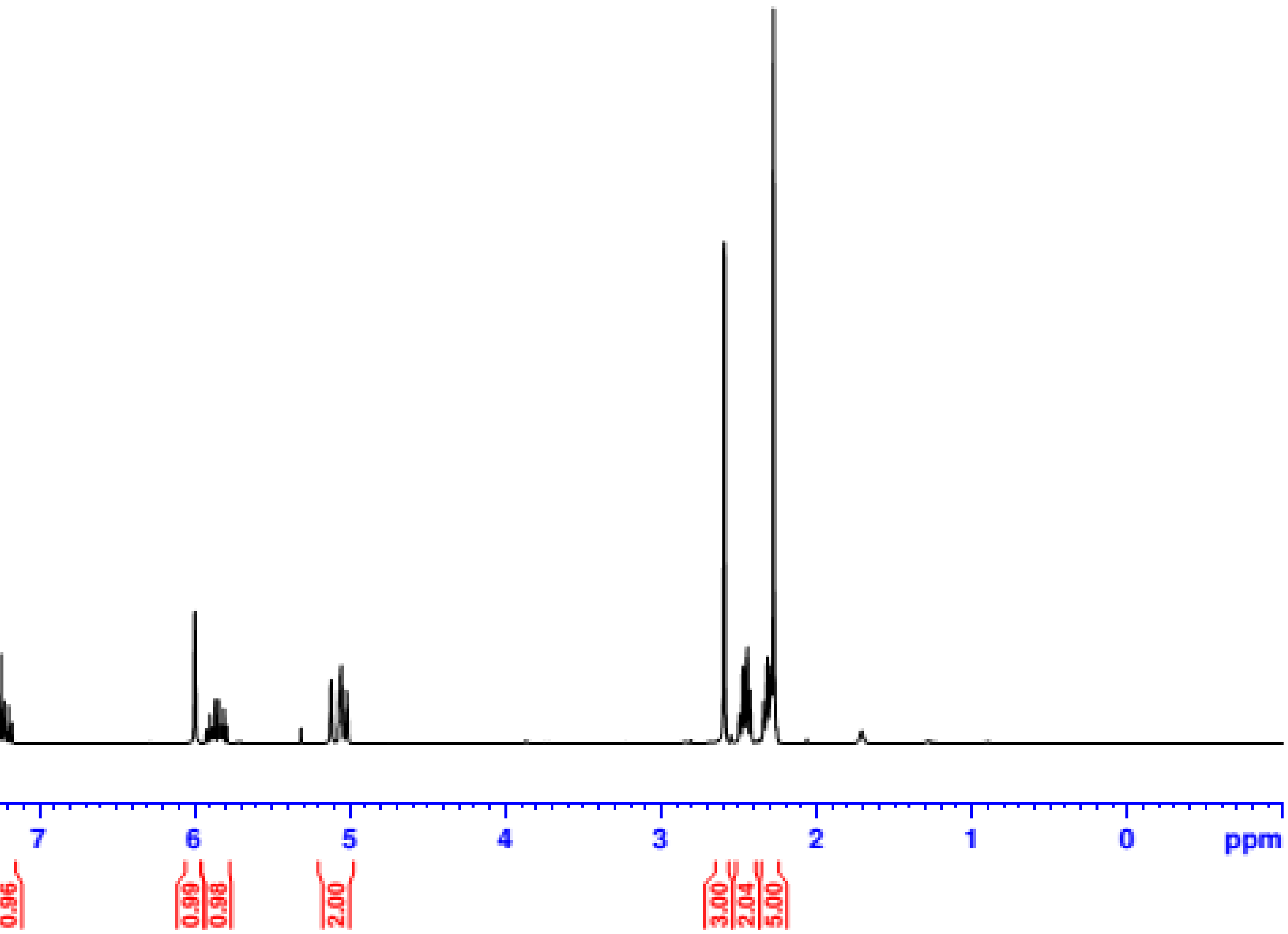

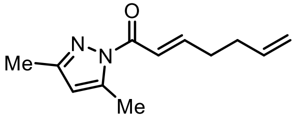

1d

^{13}C NMR (75 MHz, $CDCl_3$)

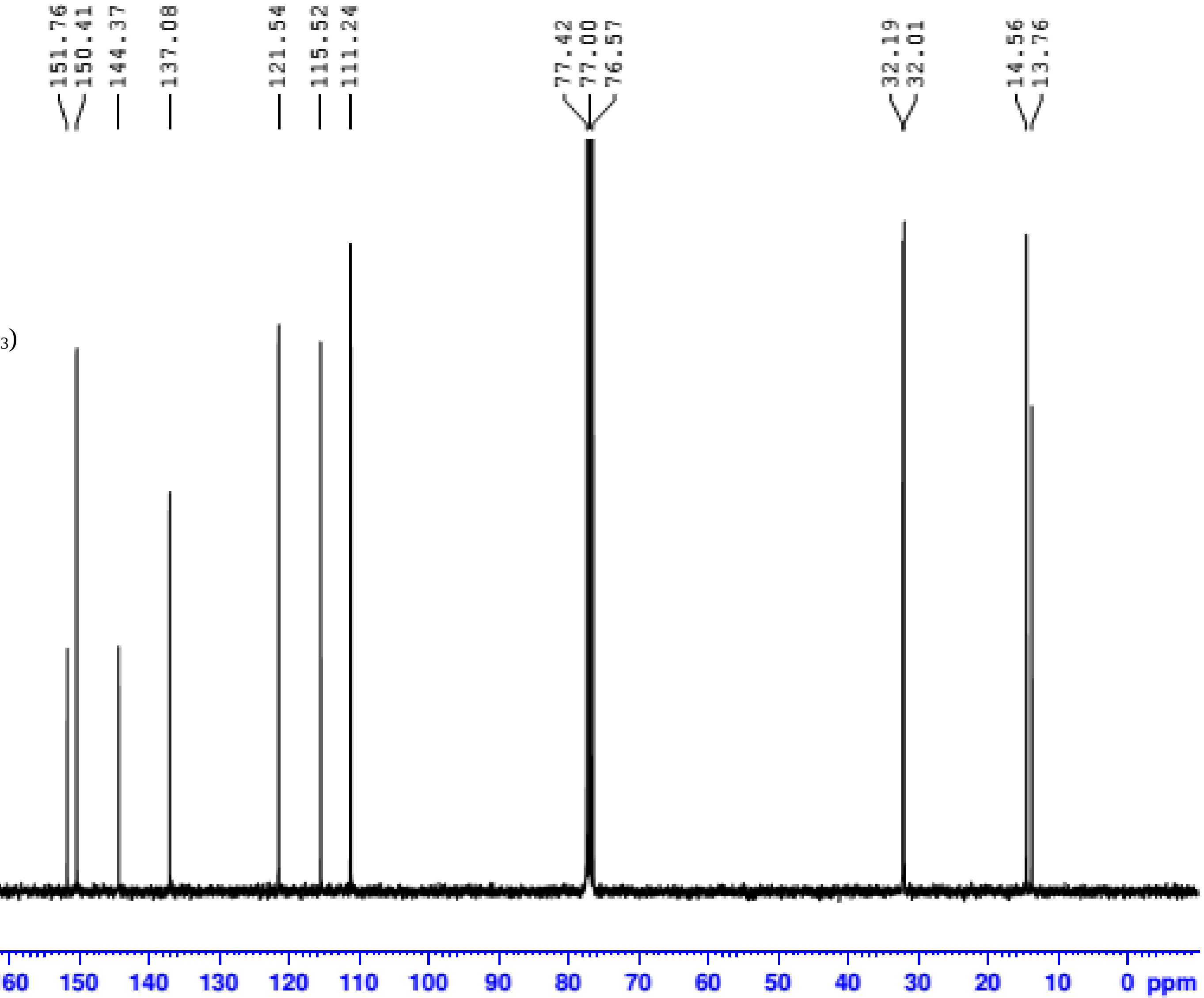

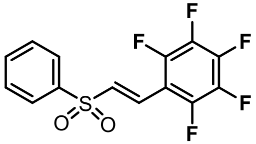

5

^{1}H NMR (500 MHz, $CDCl_3$)

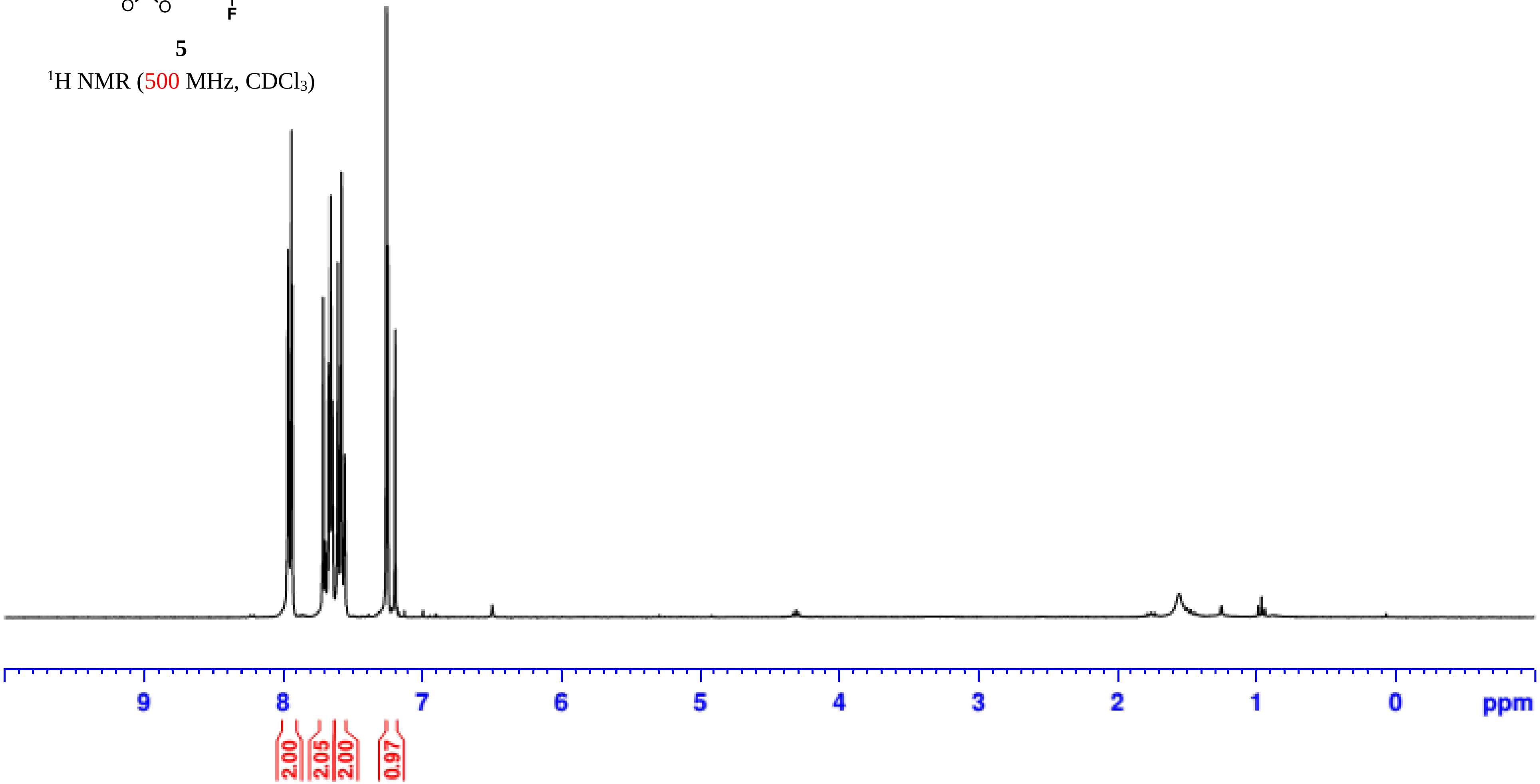

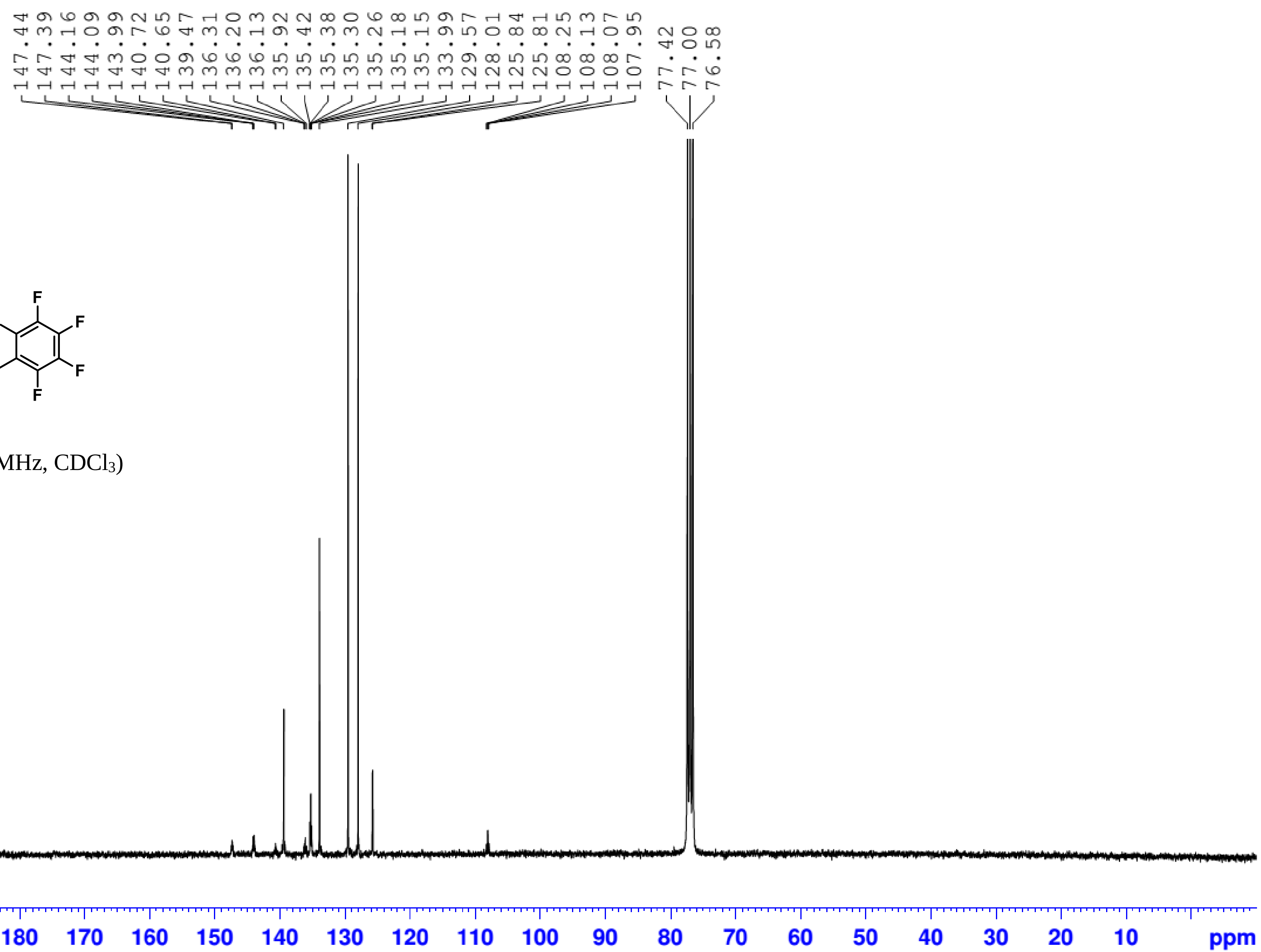

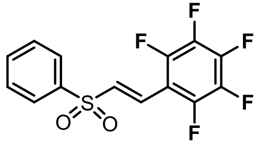

5

^{13}C NMR (125 MHz, $CDCl_3$)

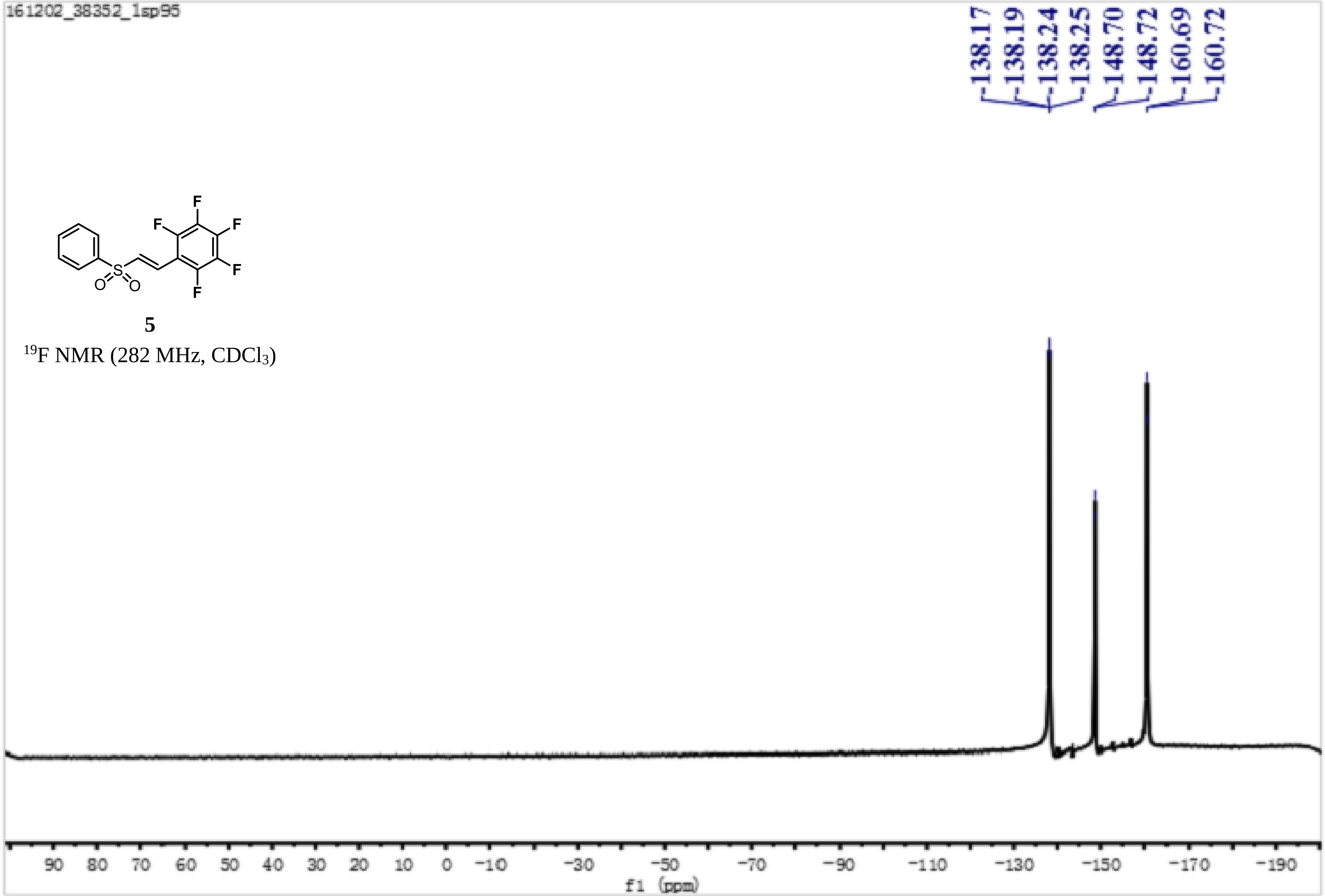

5

^{19}F NMR (282 MHz, $CDCl_3$)

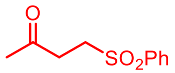

4a'

^{1}H NMR (300 MHz, $CDCl_3$)

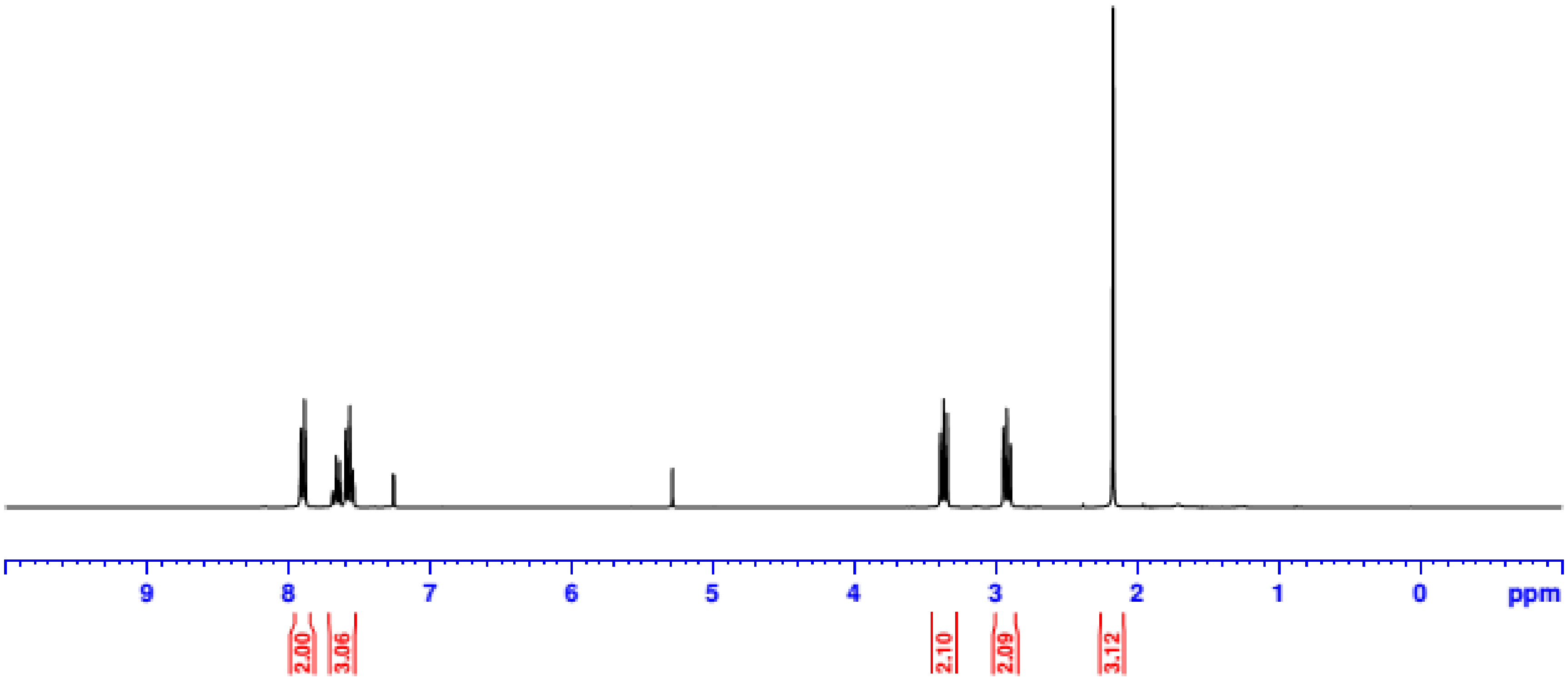

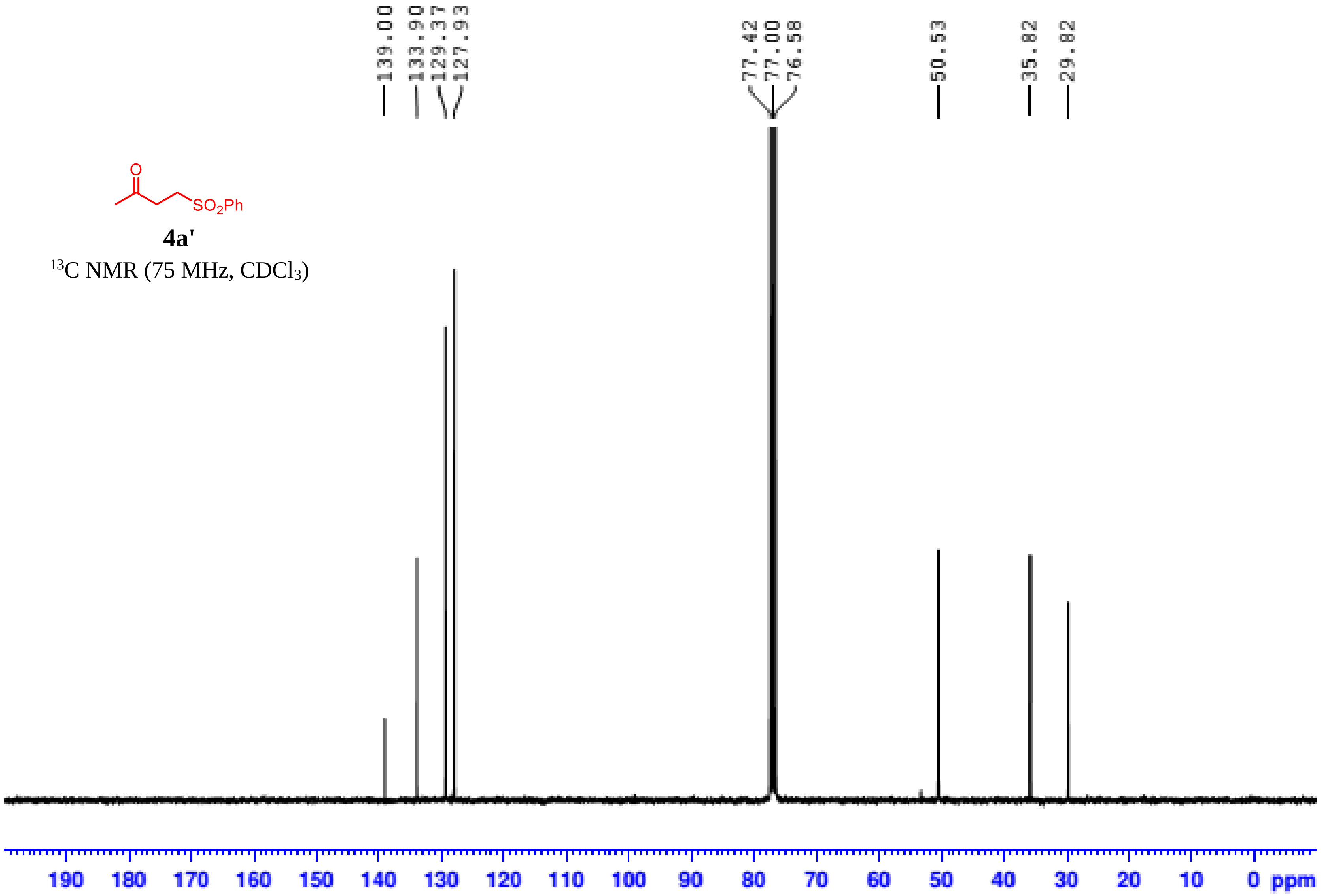

4a'

^{13}C NMR (75 MHz, $CDCl_3$)

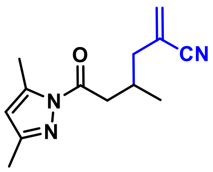

3a

^{1}H NMR (300 MHz, $CDCl_3$)

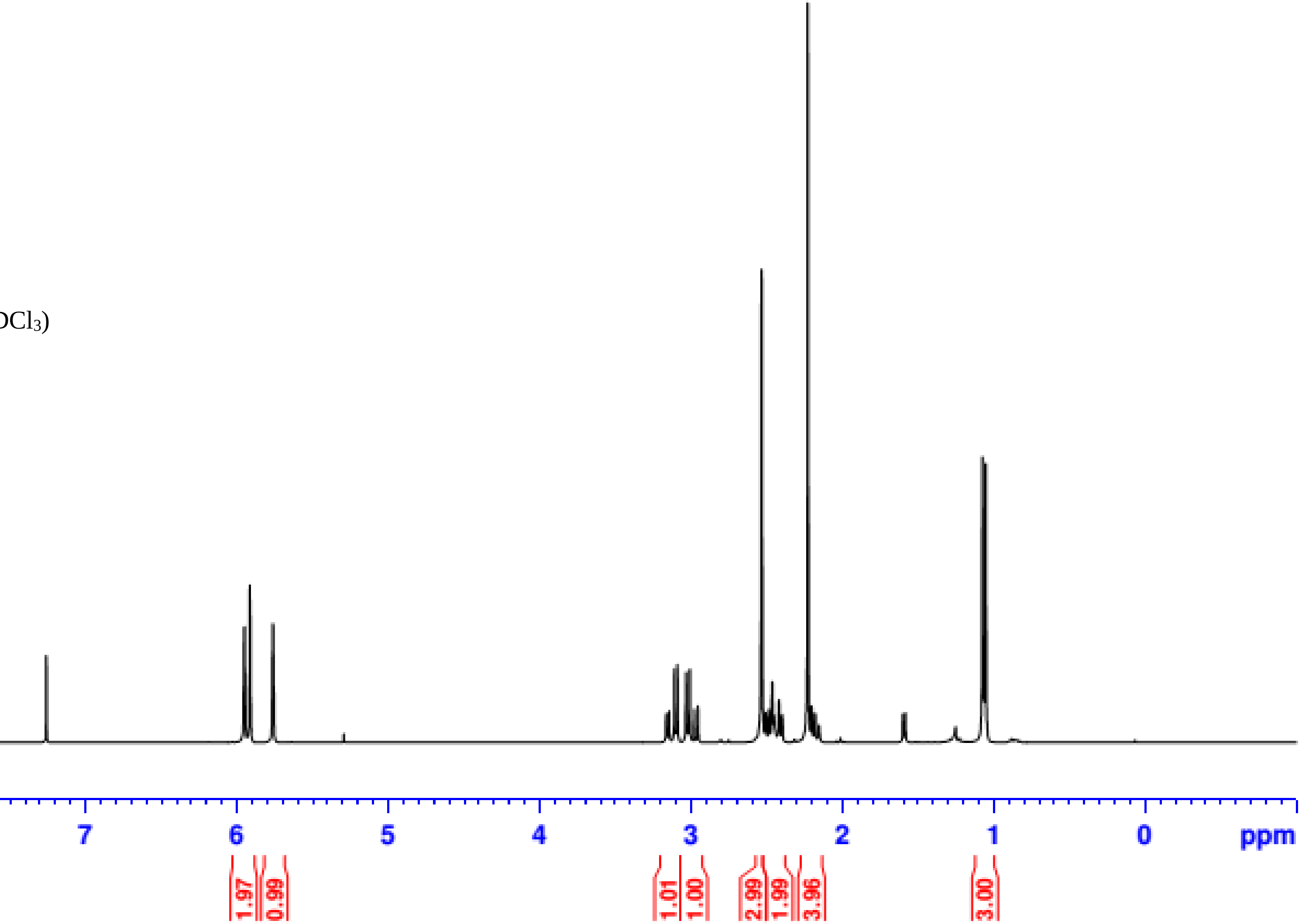

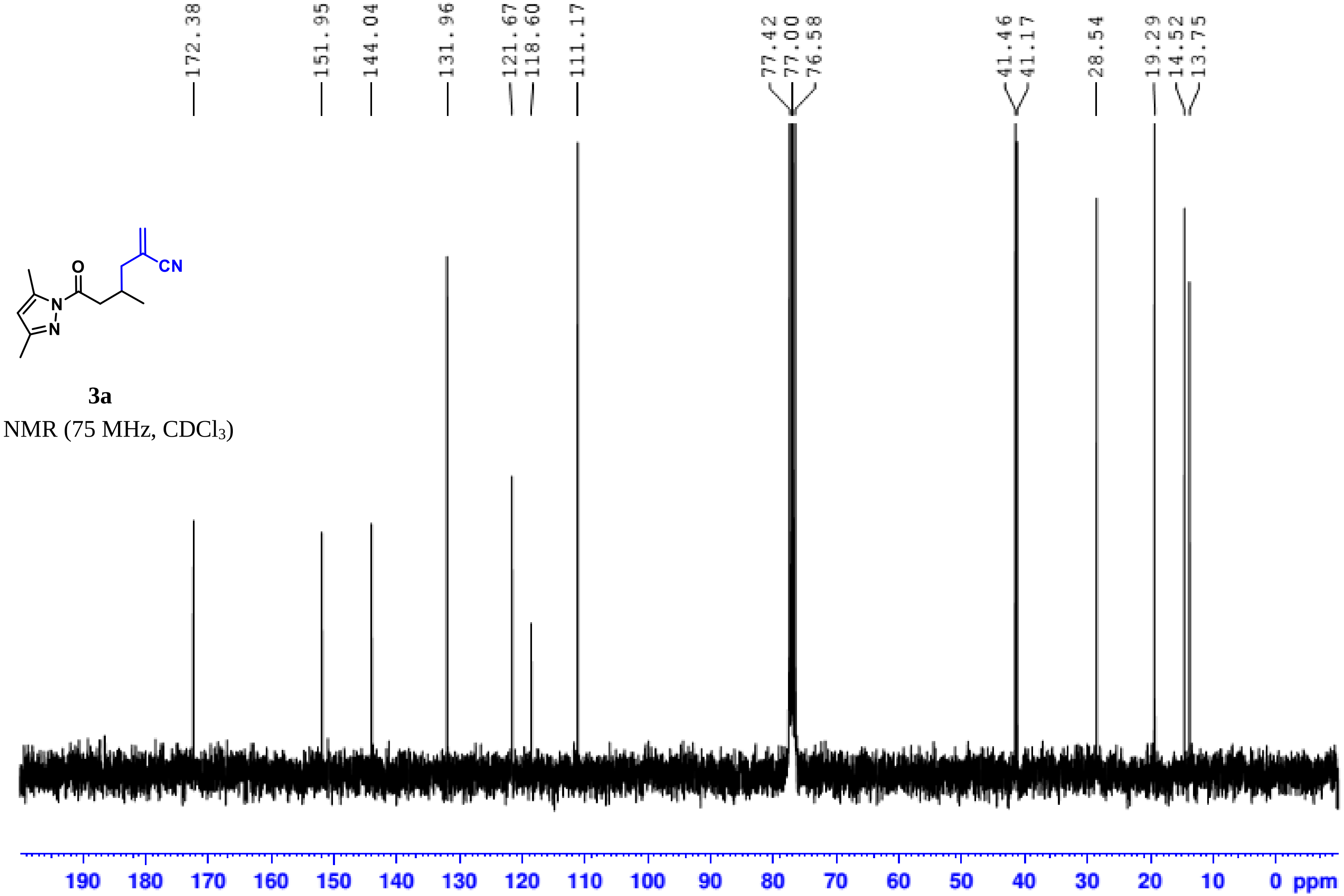

3a

^{13}C NMR (75 MHz, $CDCl_3$)

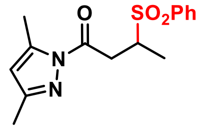

4a

^{1}H NMR (500 MHz, $CDCl_3$)

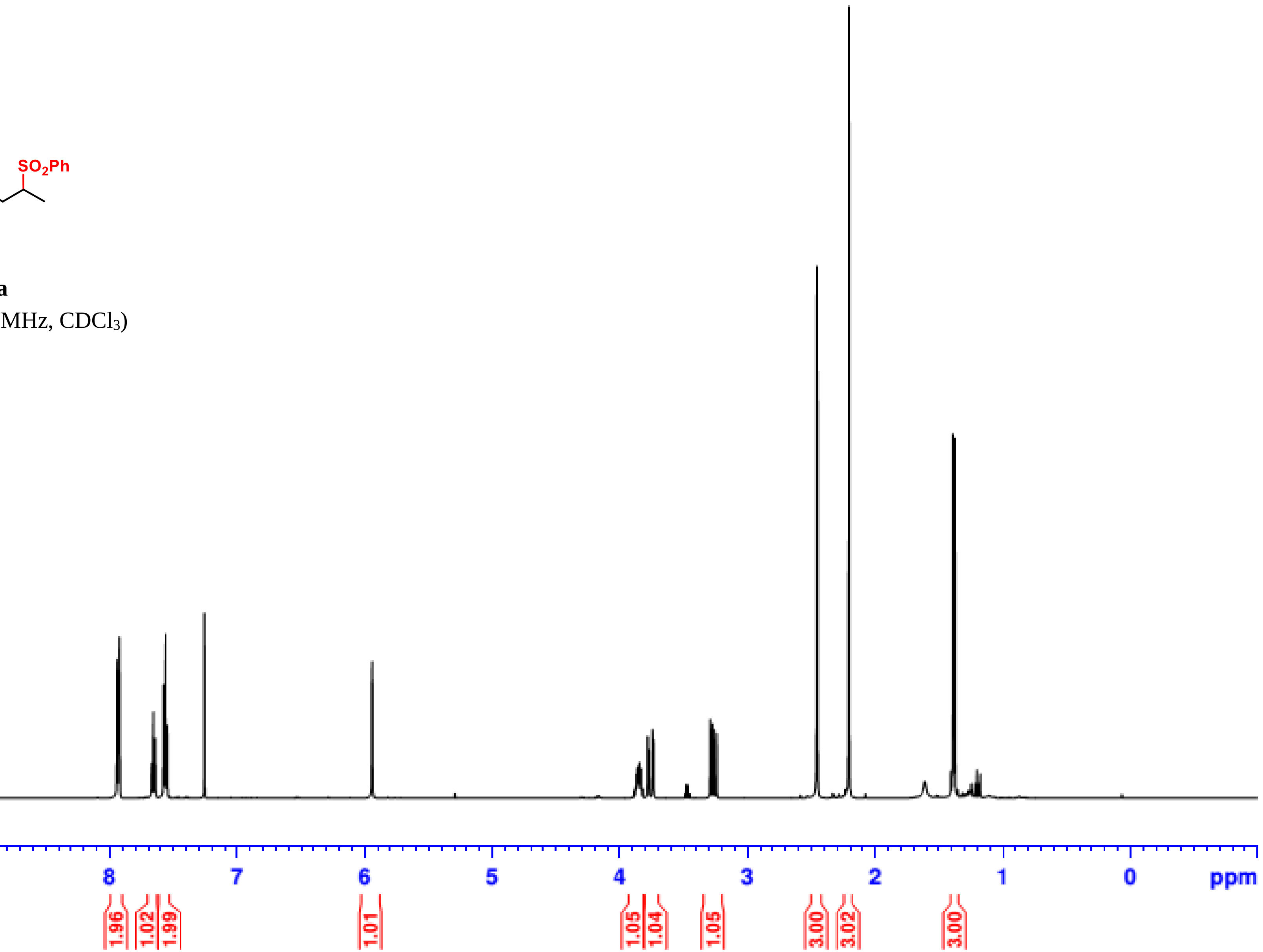

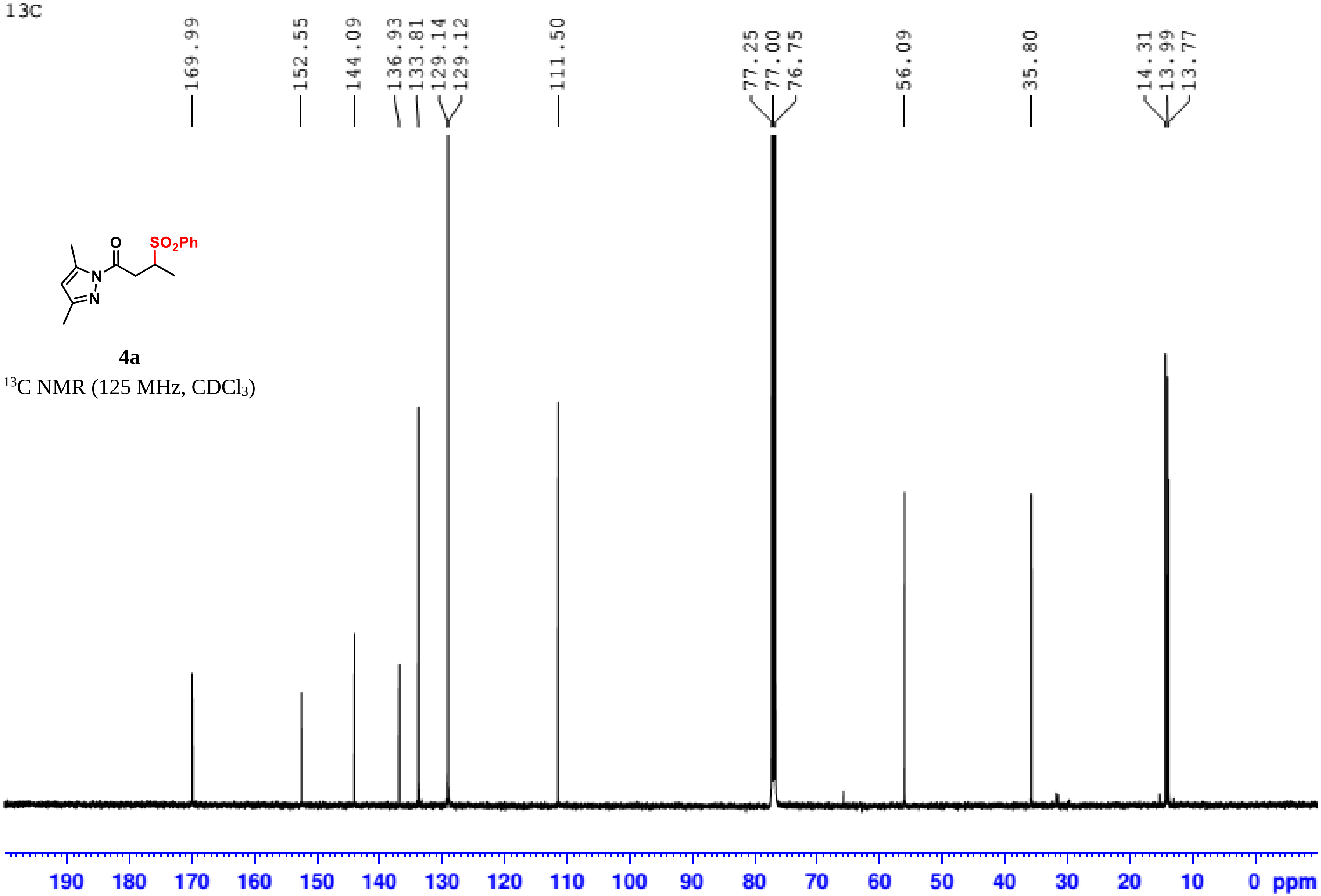

4a

^{13}C NMR (125 MHz, $CDCl_3$)

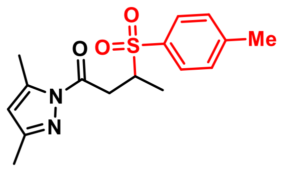

4b

^{1}H NMR (500 MHz, $CDCl_3$)

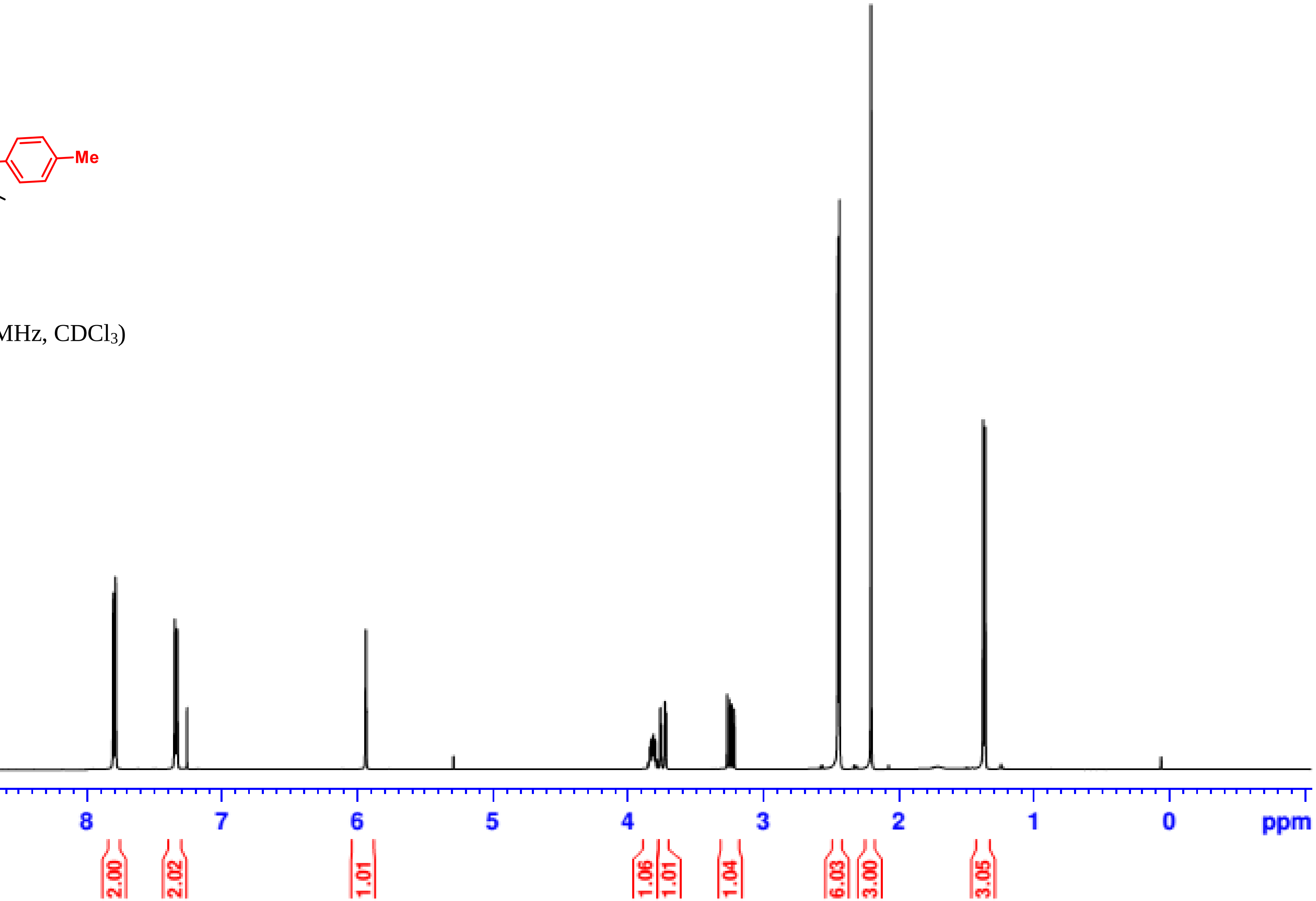

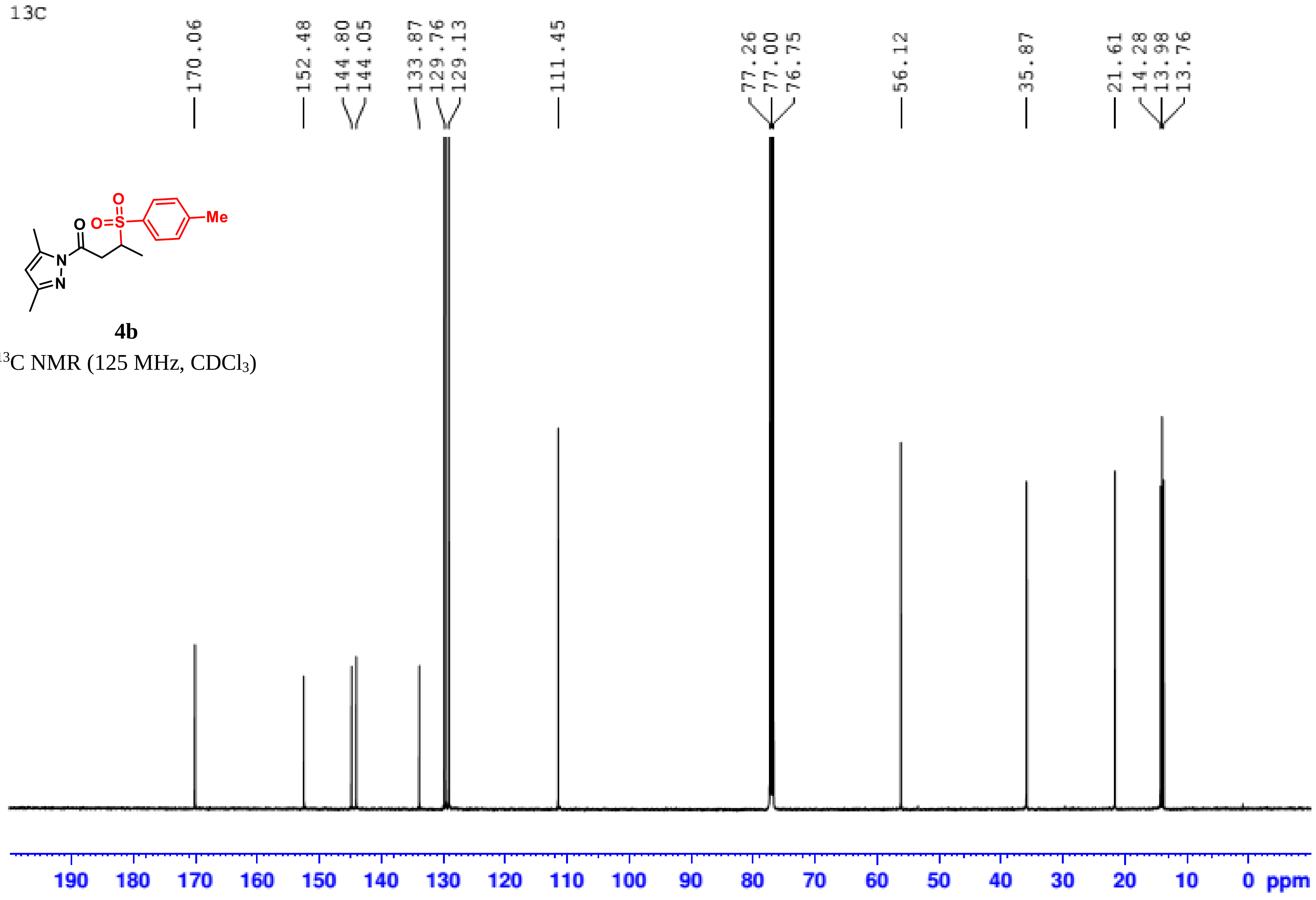

4b

^{13}C NMR (125 MHz, $CDCl_3$)

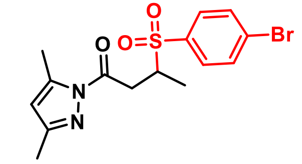

4c

^{1}H NMR (500 MHz, $CDCl_3$)

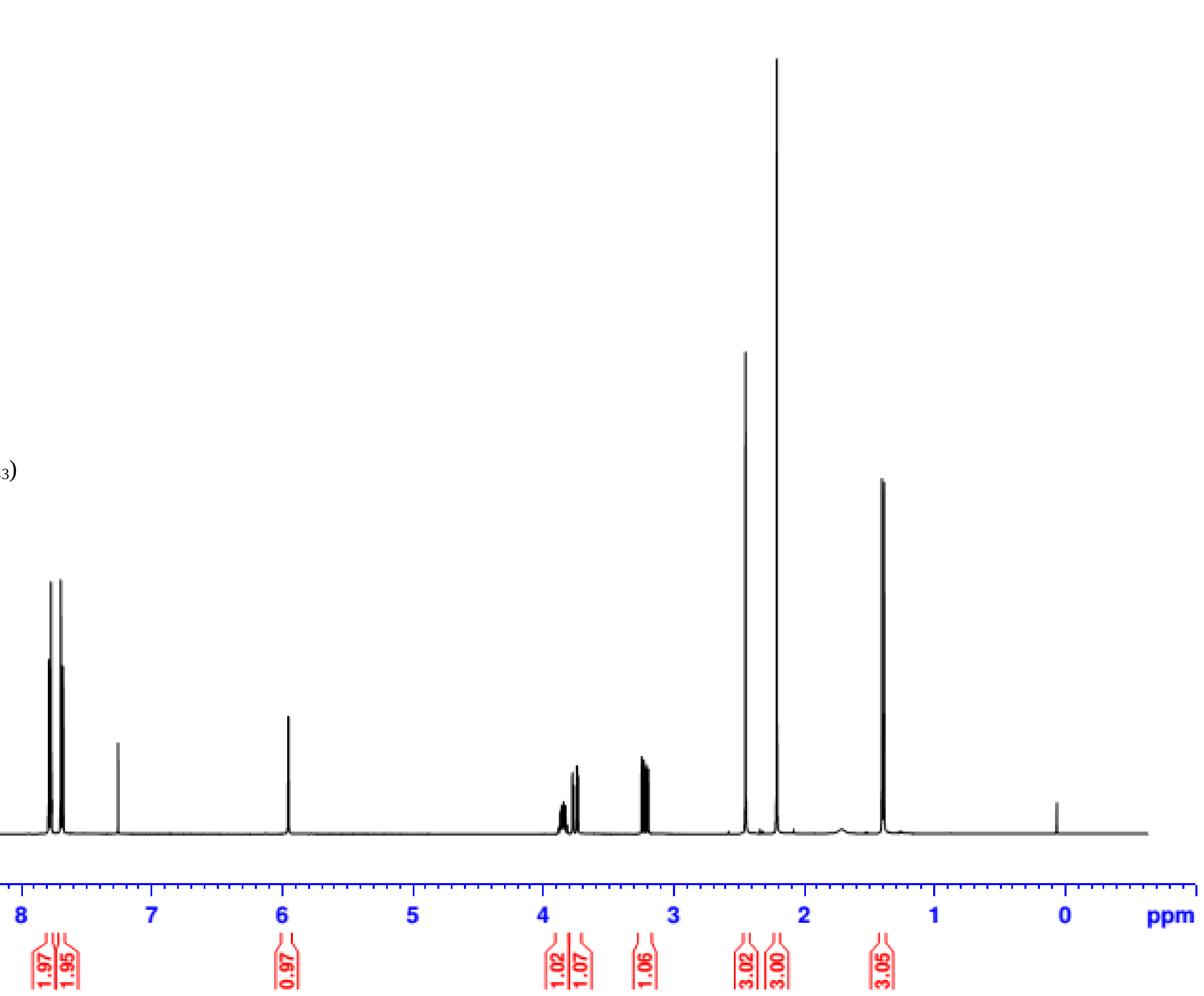

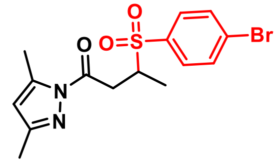

4c

^{1}H NMR (125 MHz, $CDCl_3$)

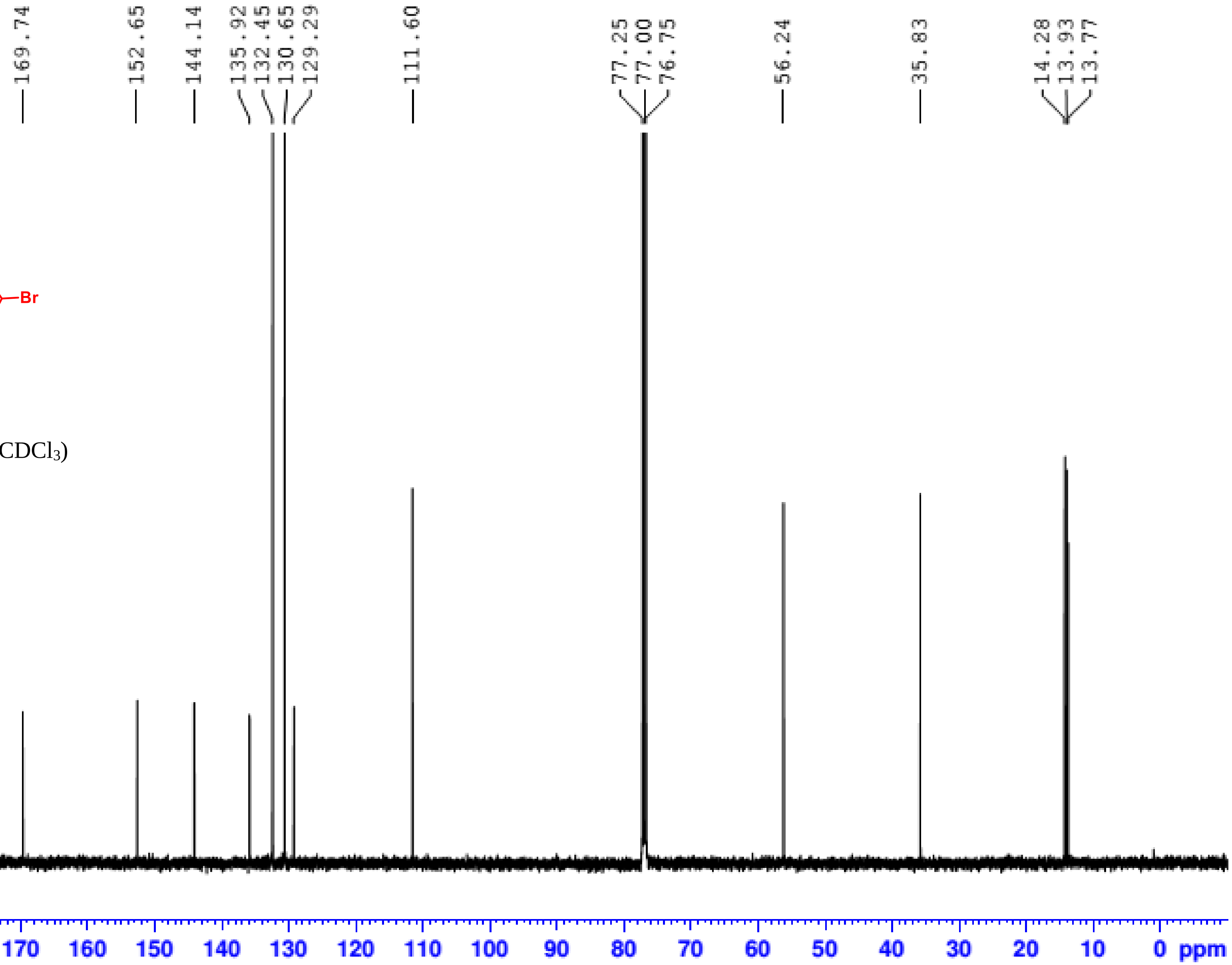

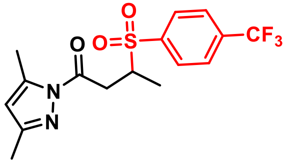

4d

^{1}H NMR (500 MHz, $CDCl_3$)

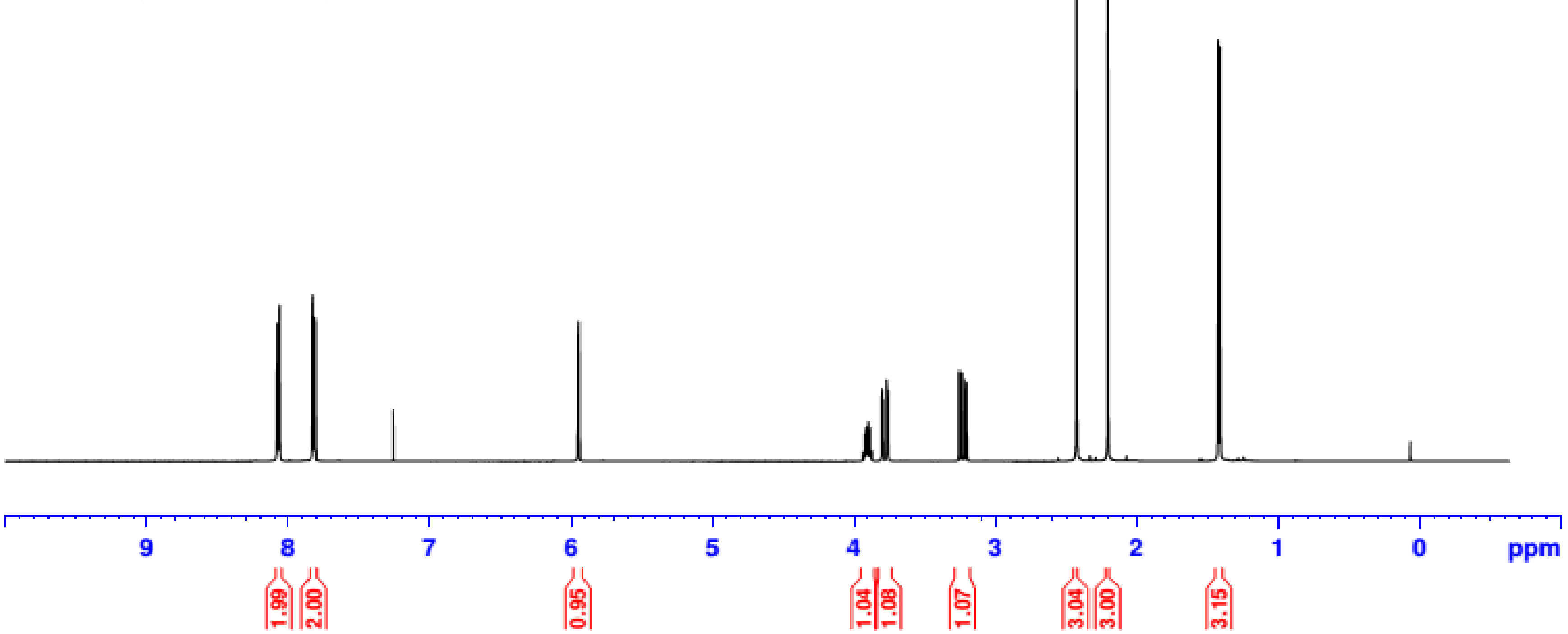

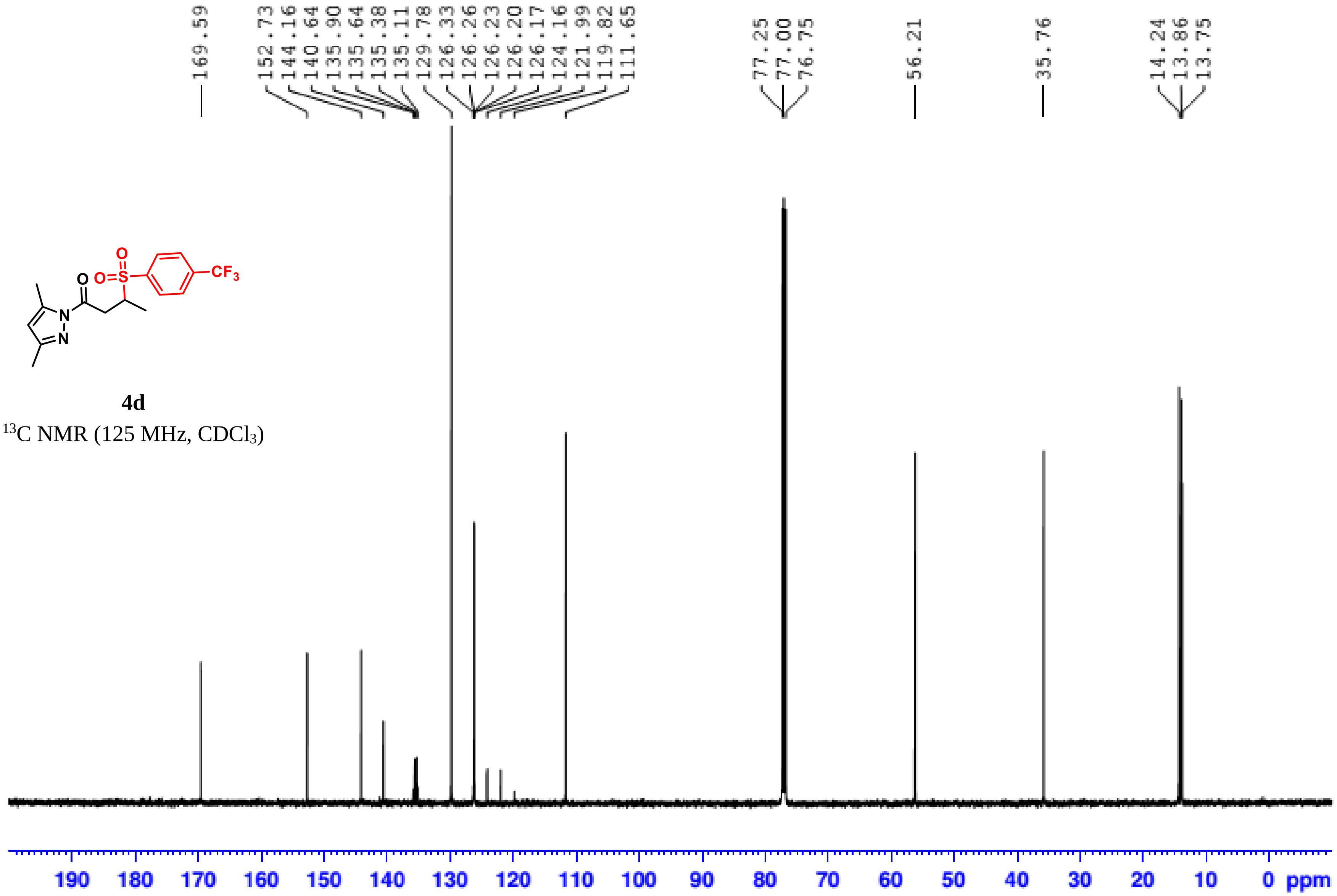

4d

^{13}C NMR (125 MHz, $CDCl_3$)

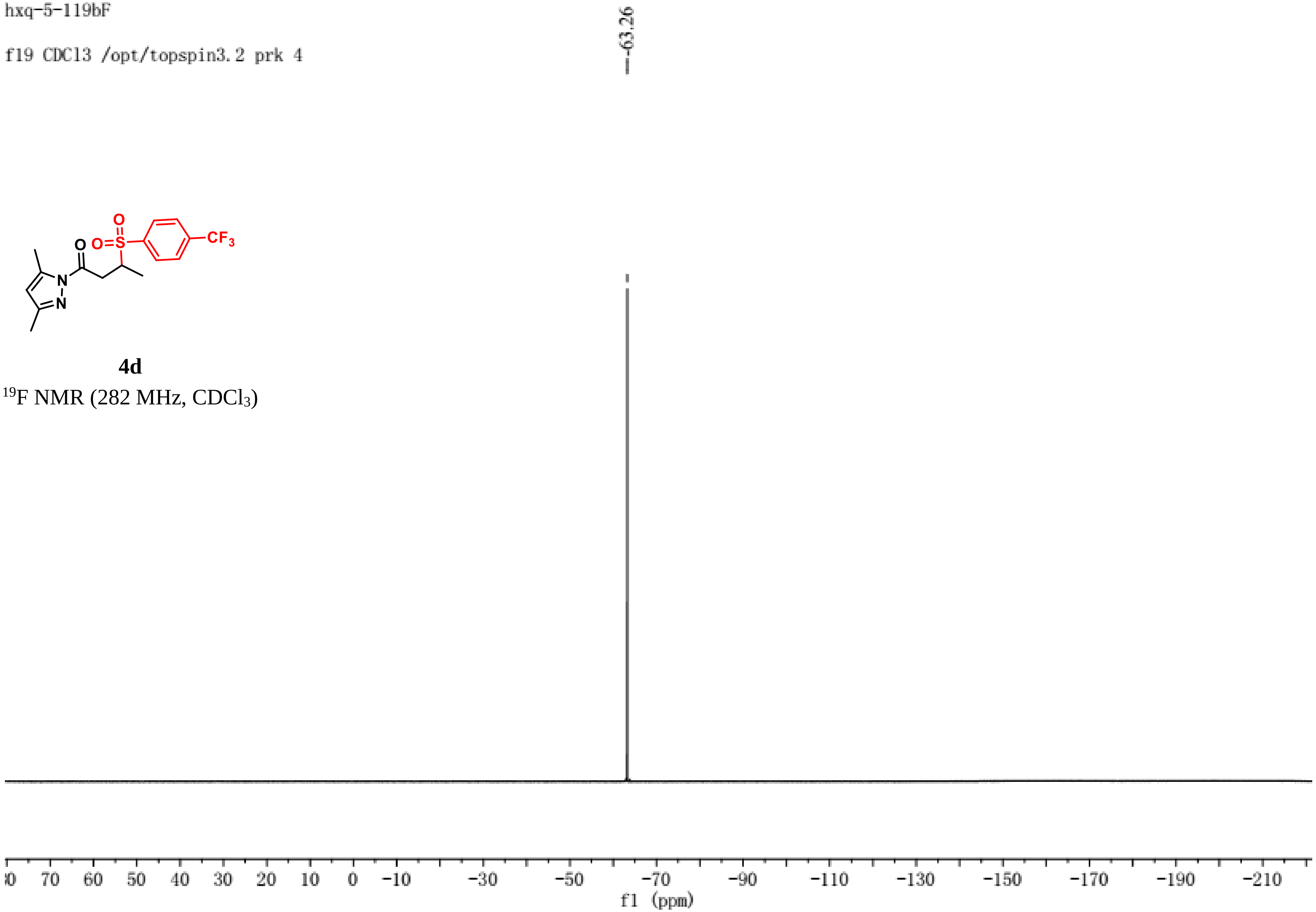

4d

^{19}F NMR (282 MHz, $CDCl_3$)

1H

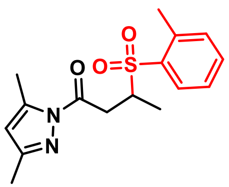

4e

^{1}H NMR (500 MHz, $CDCl_3$)

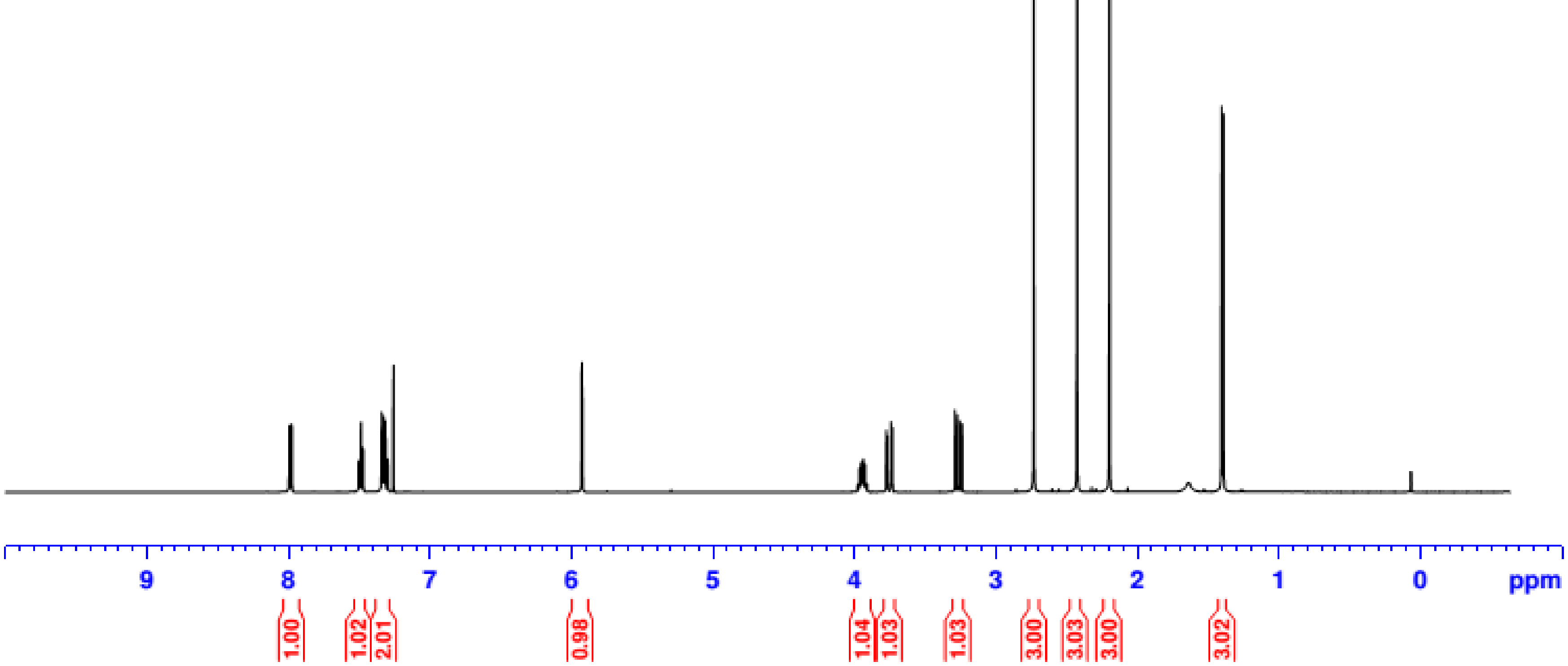

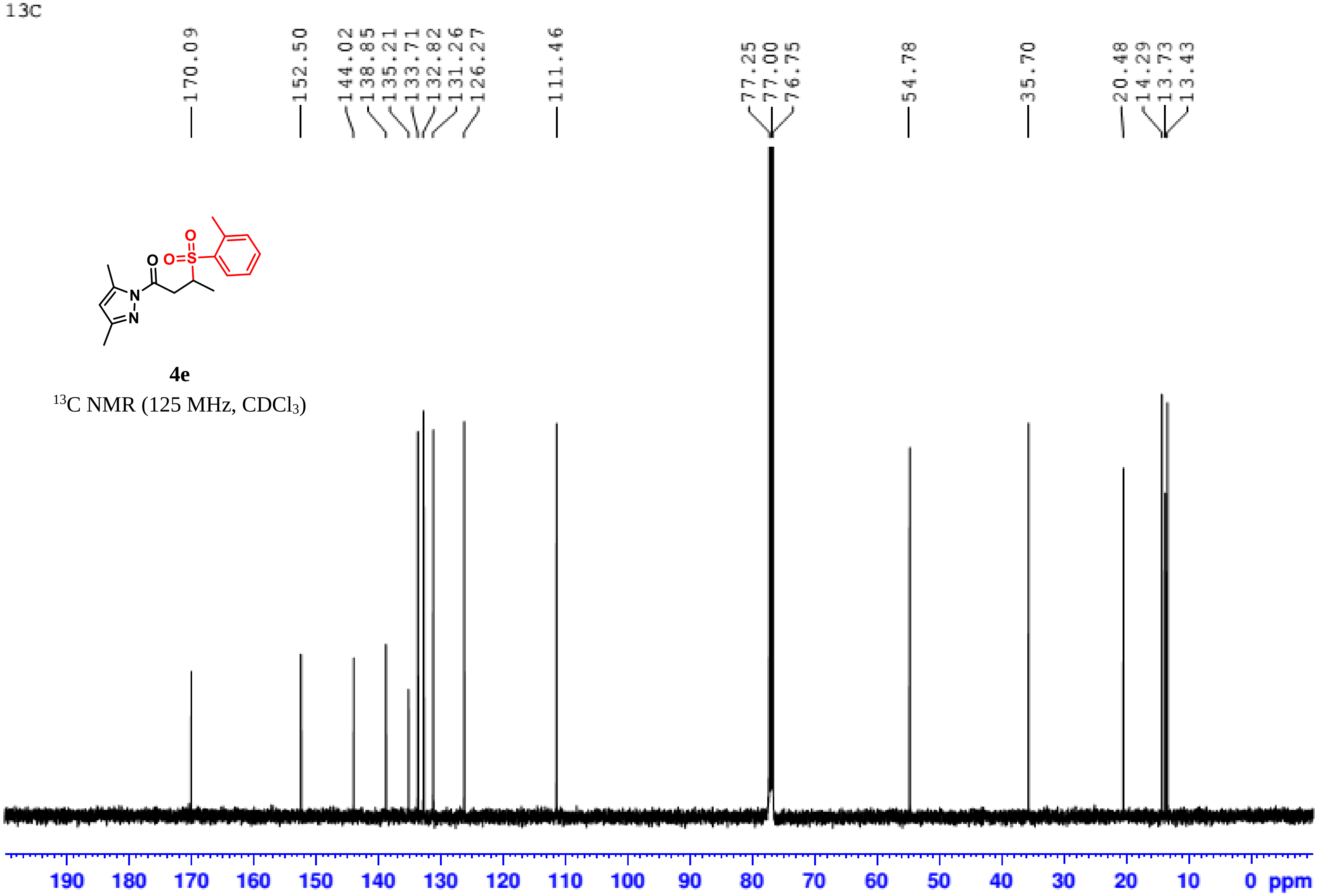

4e

^{13}C NMR (125 MHz, $CDCl_3$)

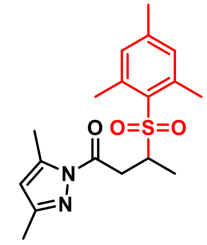

4f

^{1}H NMR (500 MHz, $CDCl_3$)

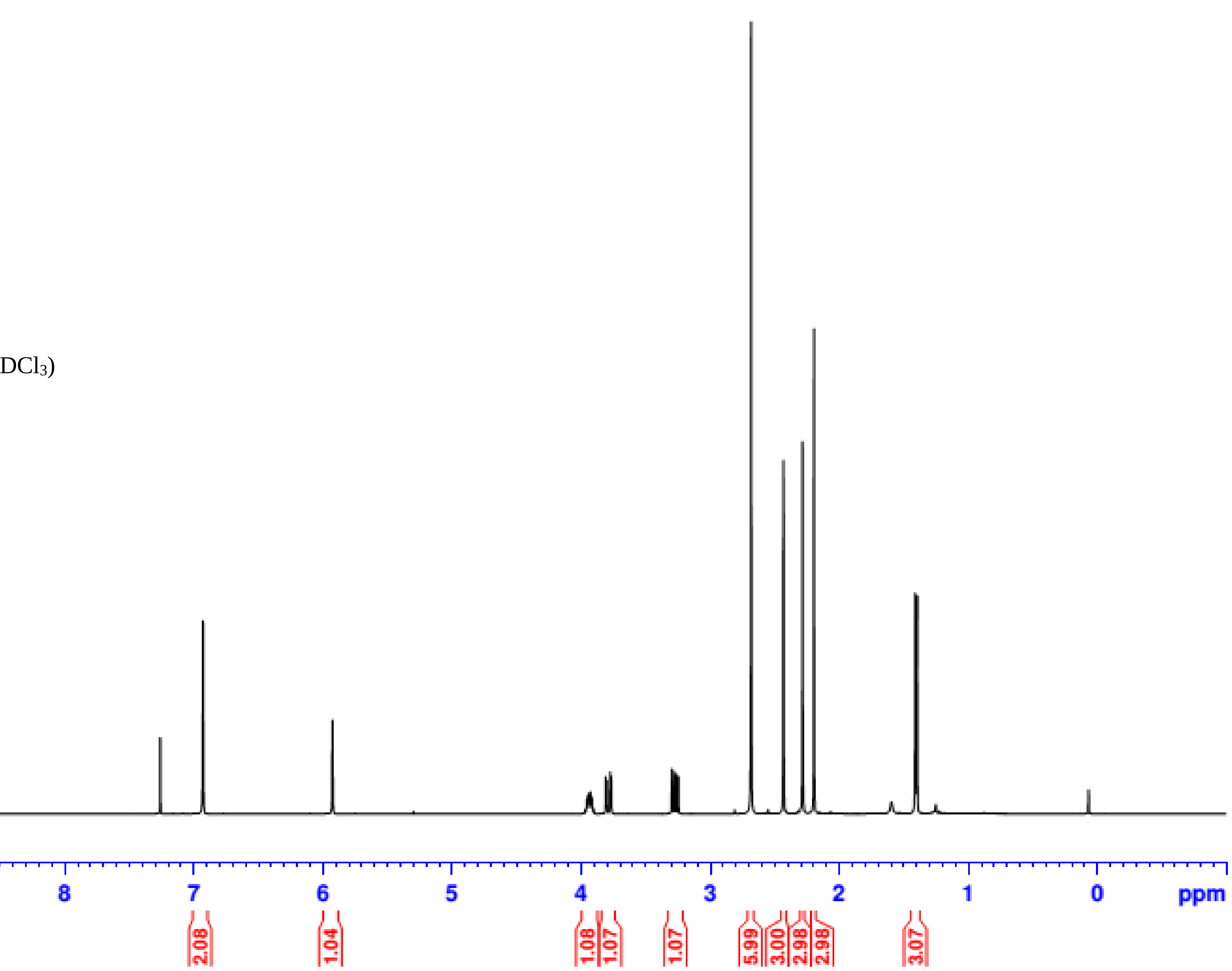

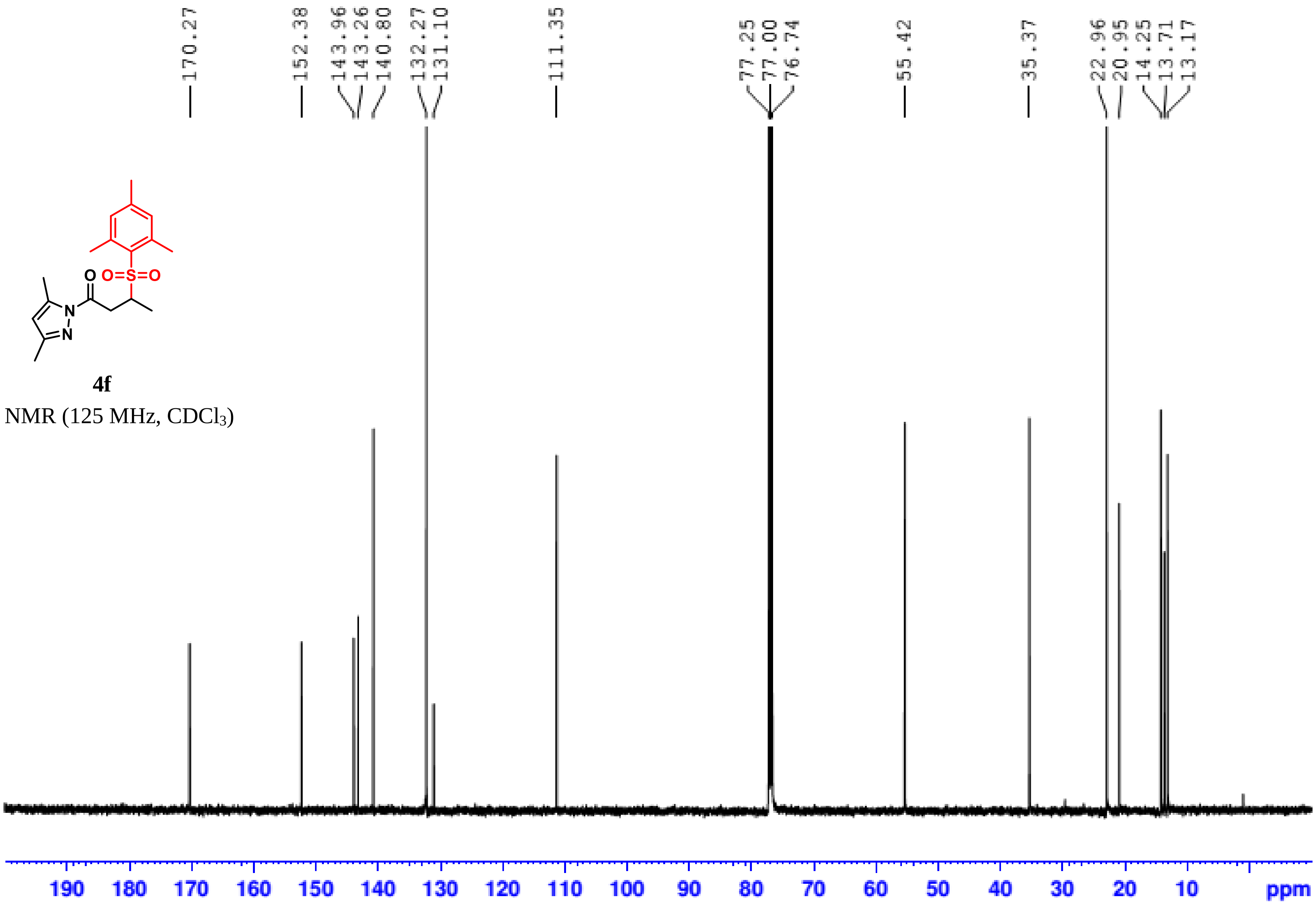

4f

^{13}C NMR (125 MHz, $CDCl_3$)

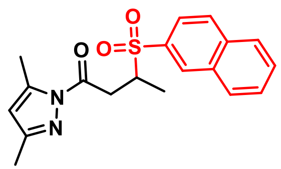

4g

^{1}H NMR (300 MHz, $CDCl_3$)

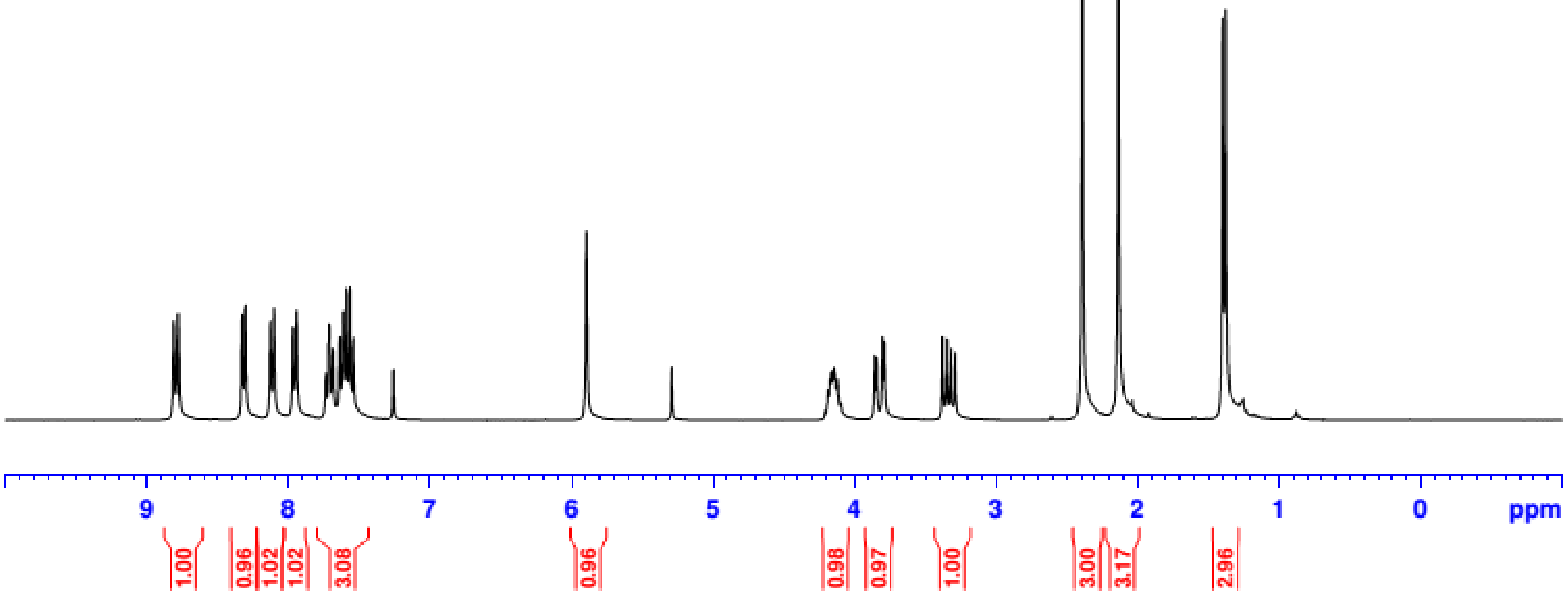

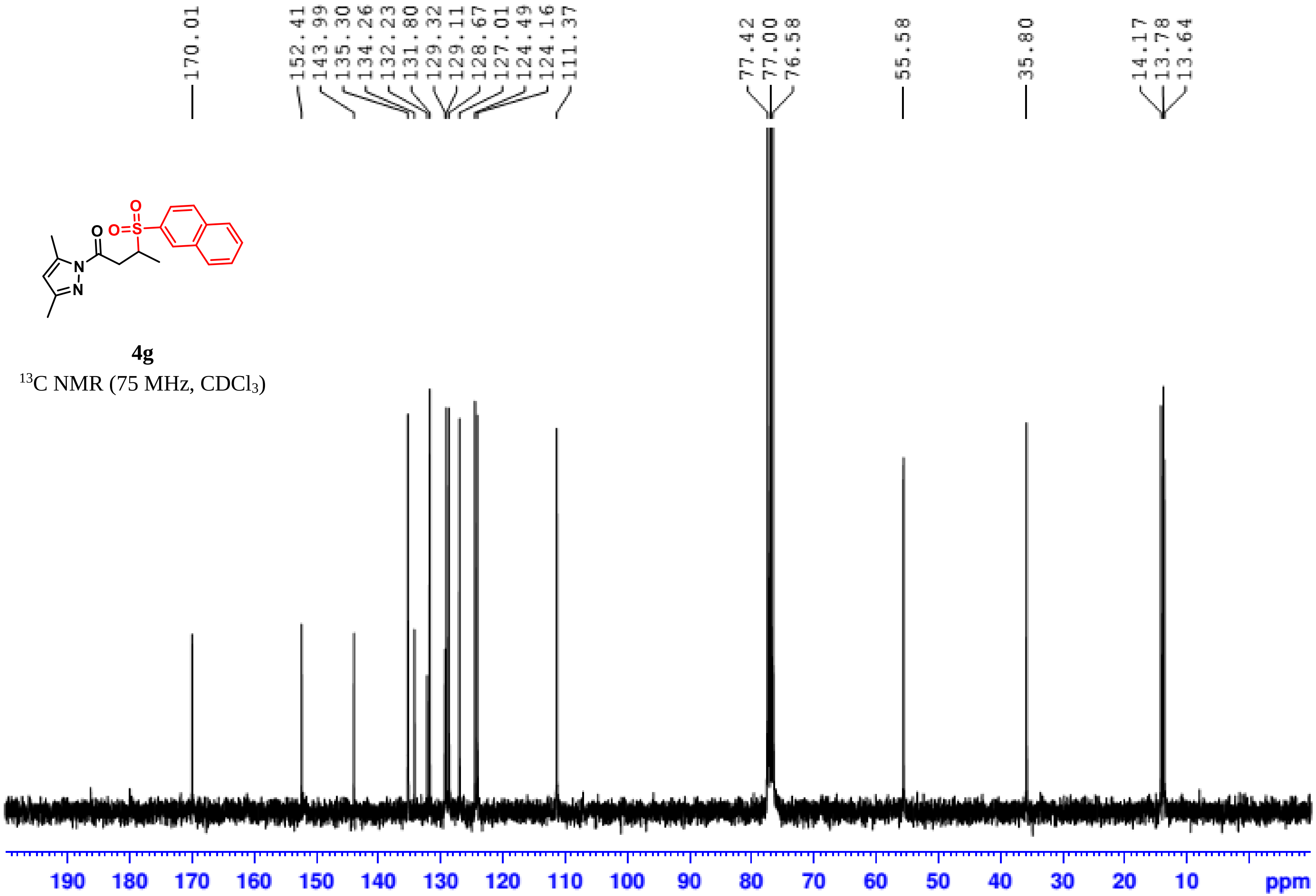

4g

^{13}C NMR (75 MHz, $CDCl_3$)

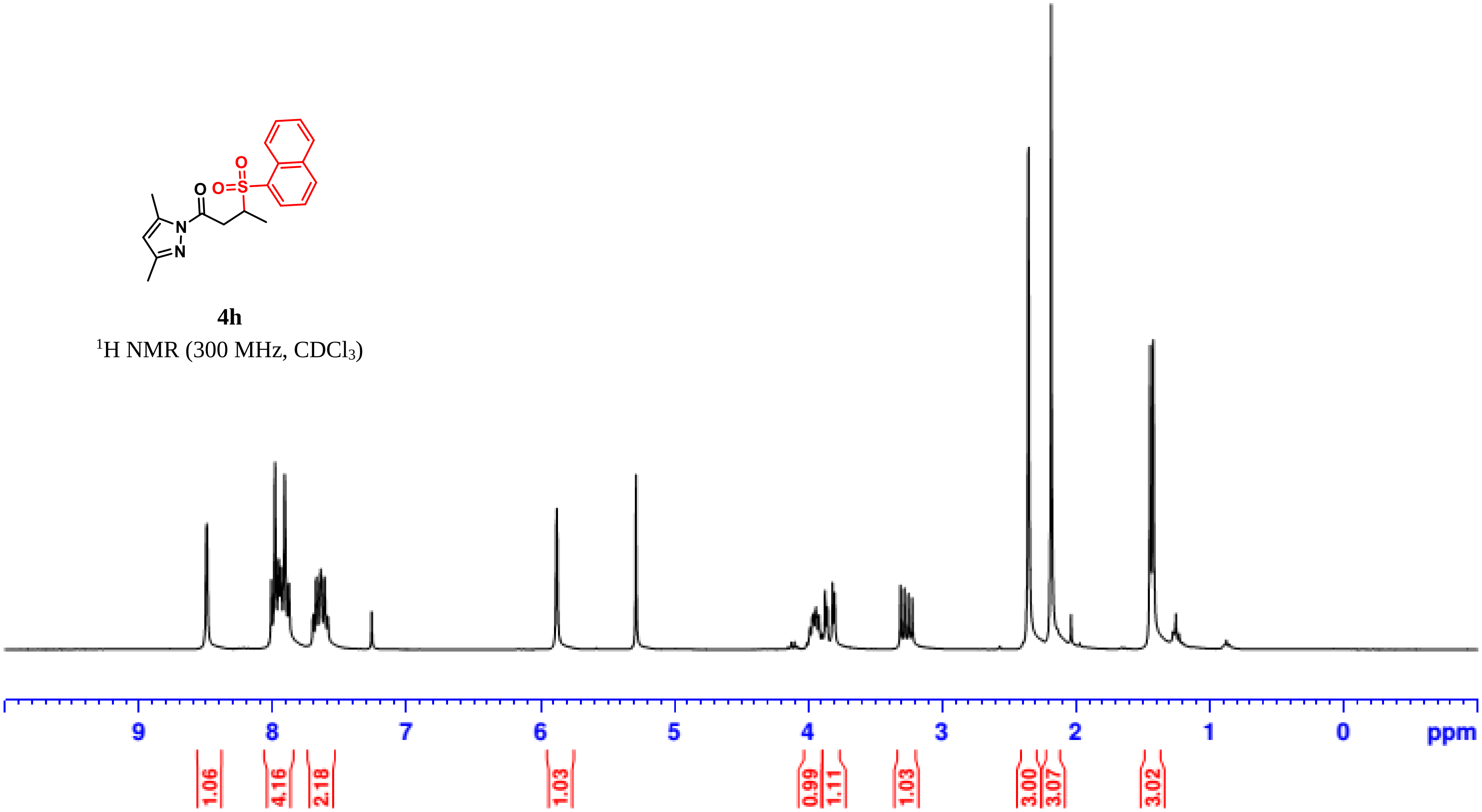

4h

^{1}H NMR (300 MHz, $CDCl_3$)

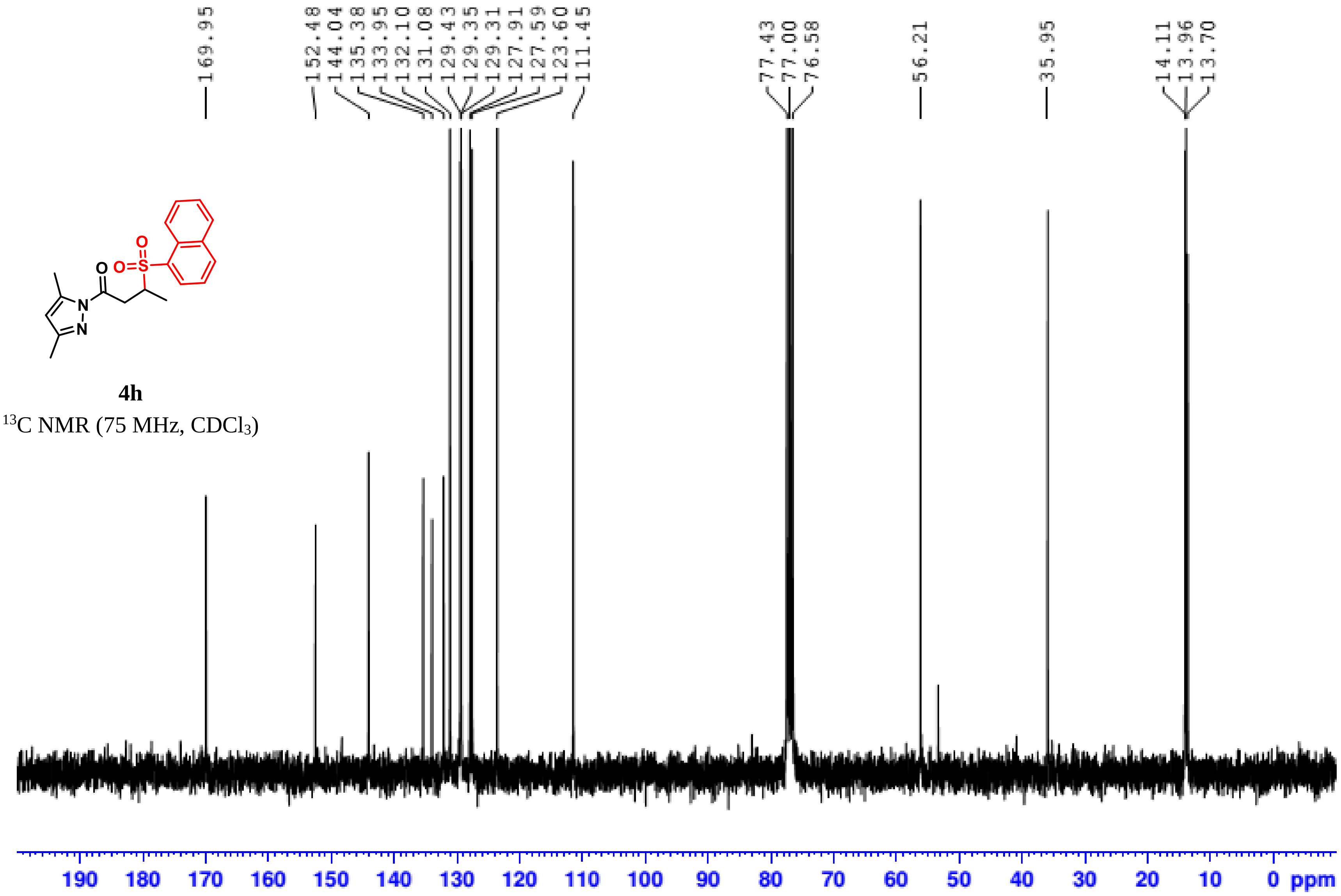

4h

^{13}C NMR (75 MHz, $CDCl_3$)

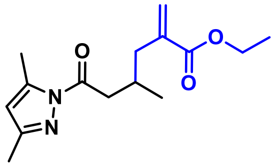

3b

^{1}H NMR (300 MHz, $CDCl_3$)

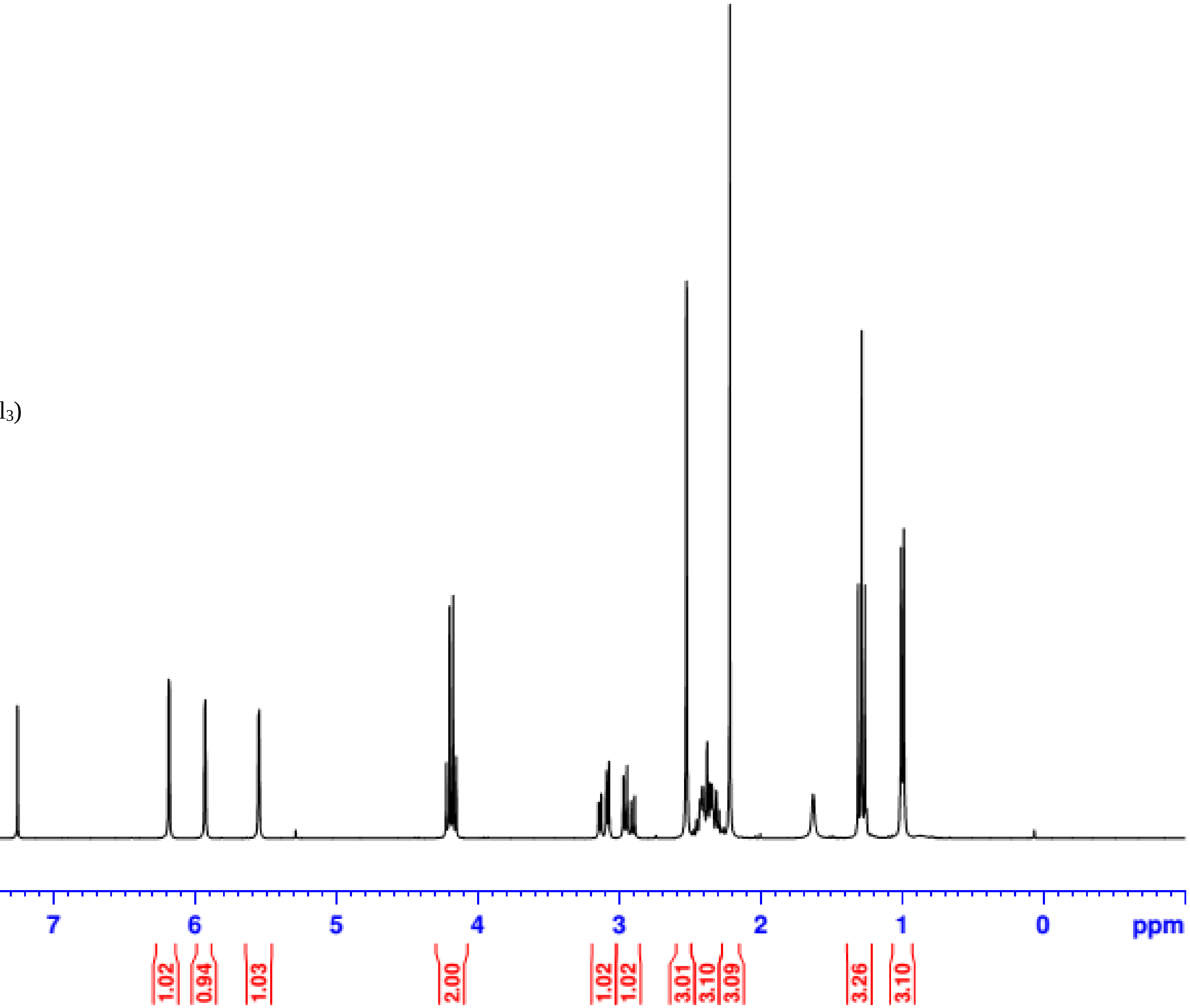

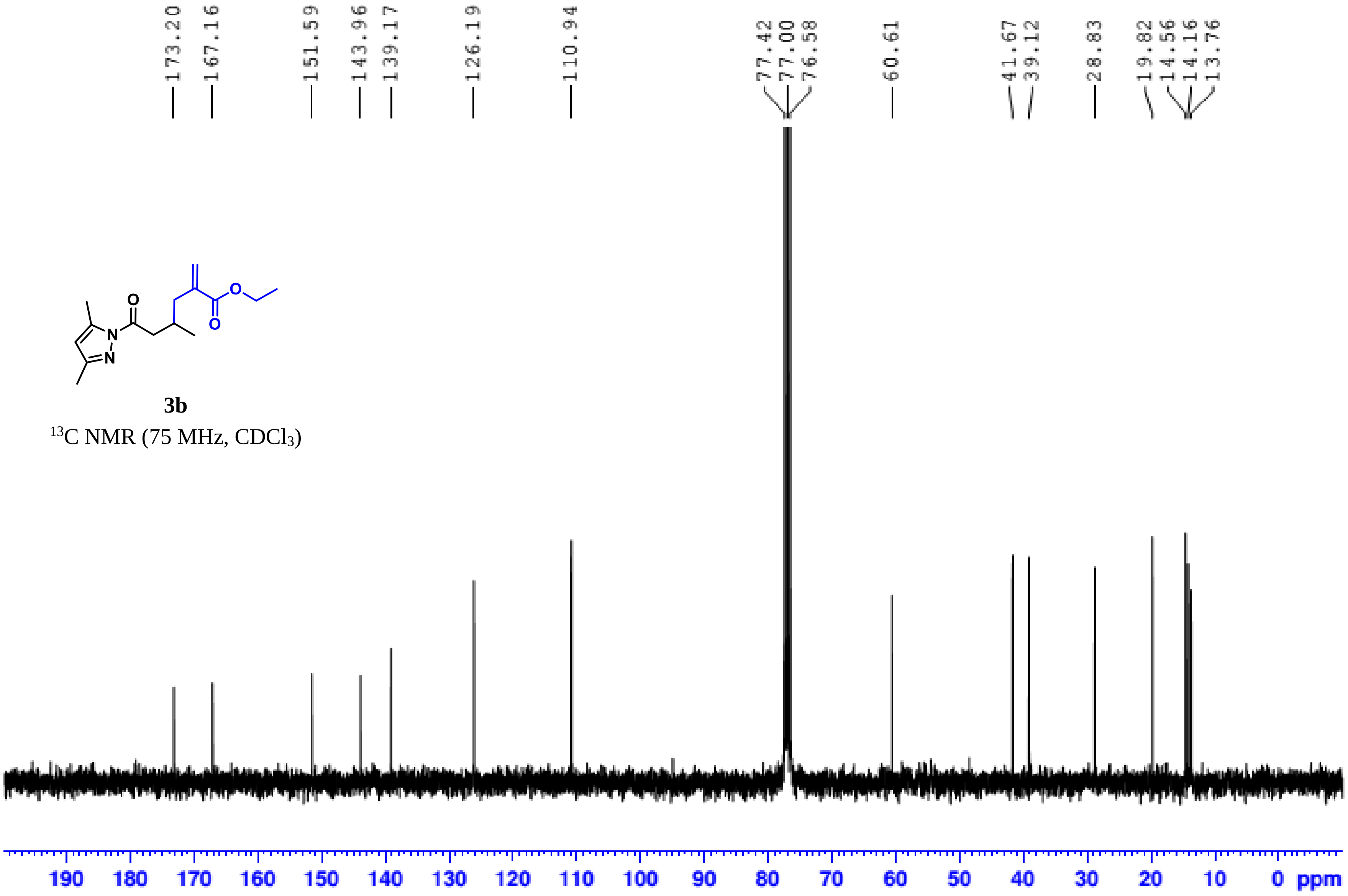

3b

^{13}C NMR (75 MHz, $CDCl_3$)

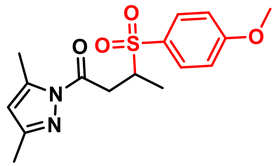

4i

^{1}H NMR (500 MHz, $CDCl_3$)

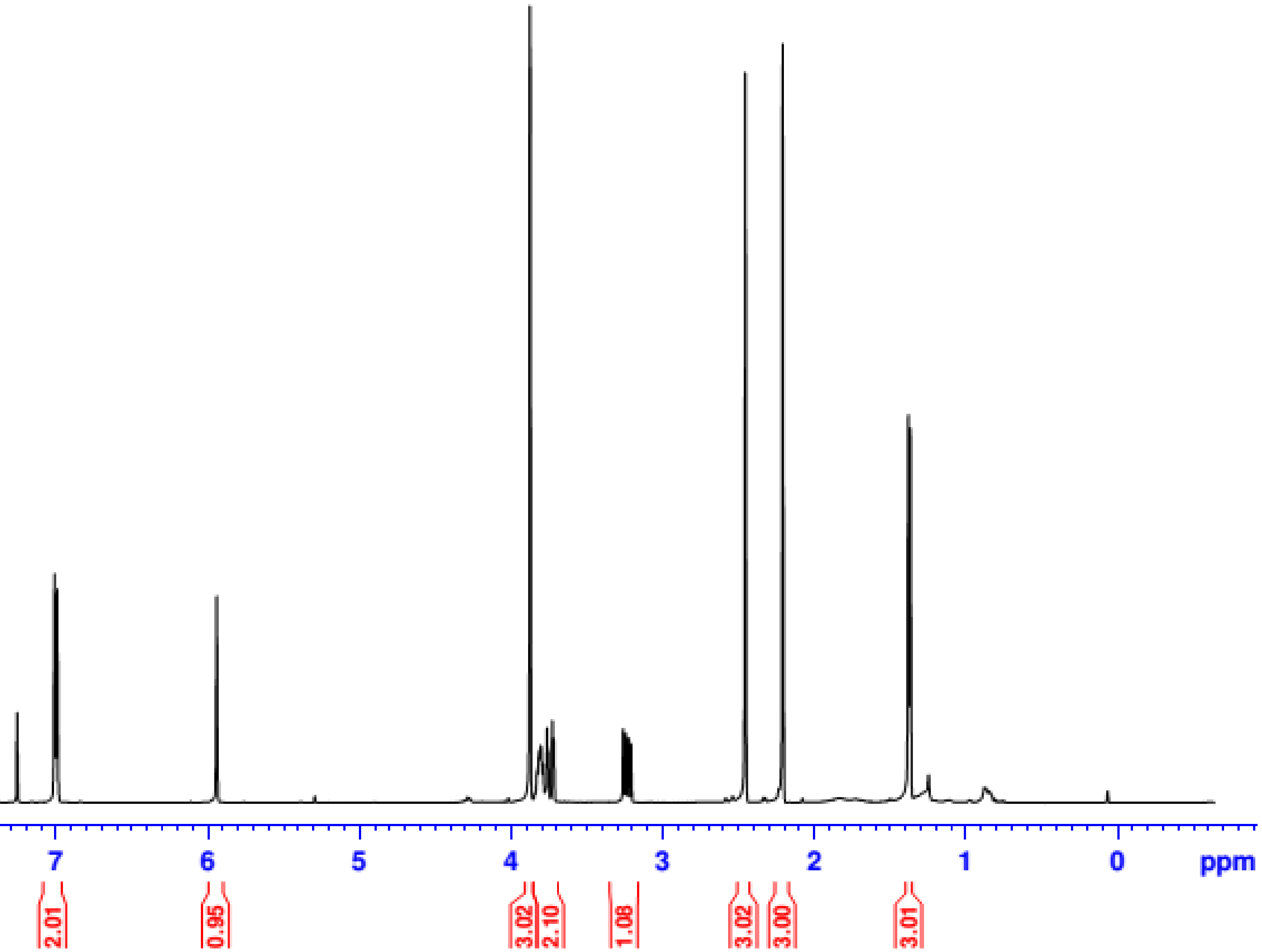

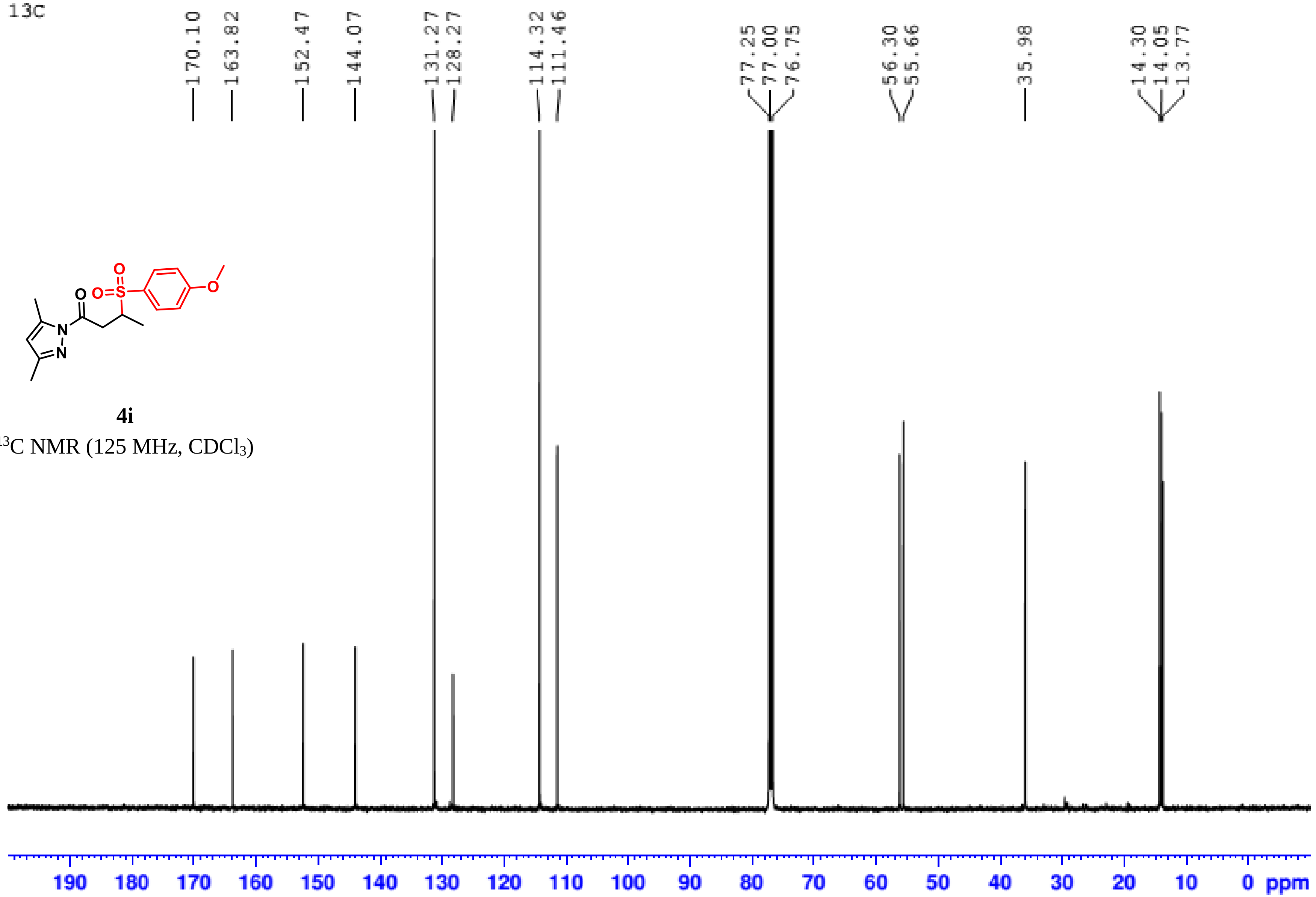

4i

^{13}C NMR (125 MHz, $CDCl_3$)

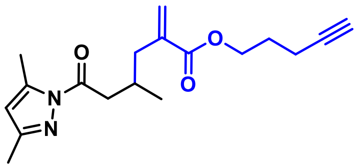

3c

^{1}H NMR (500 MHz, $CDCl_3$)

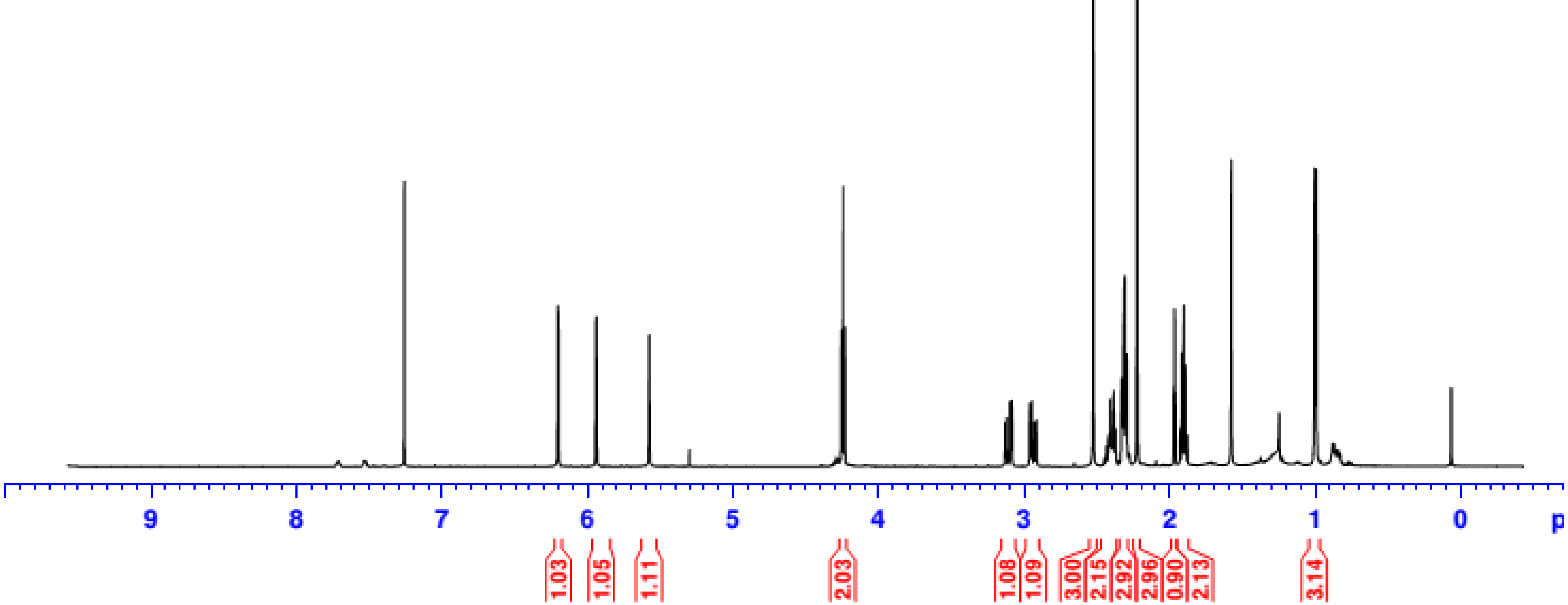

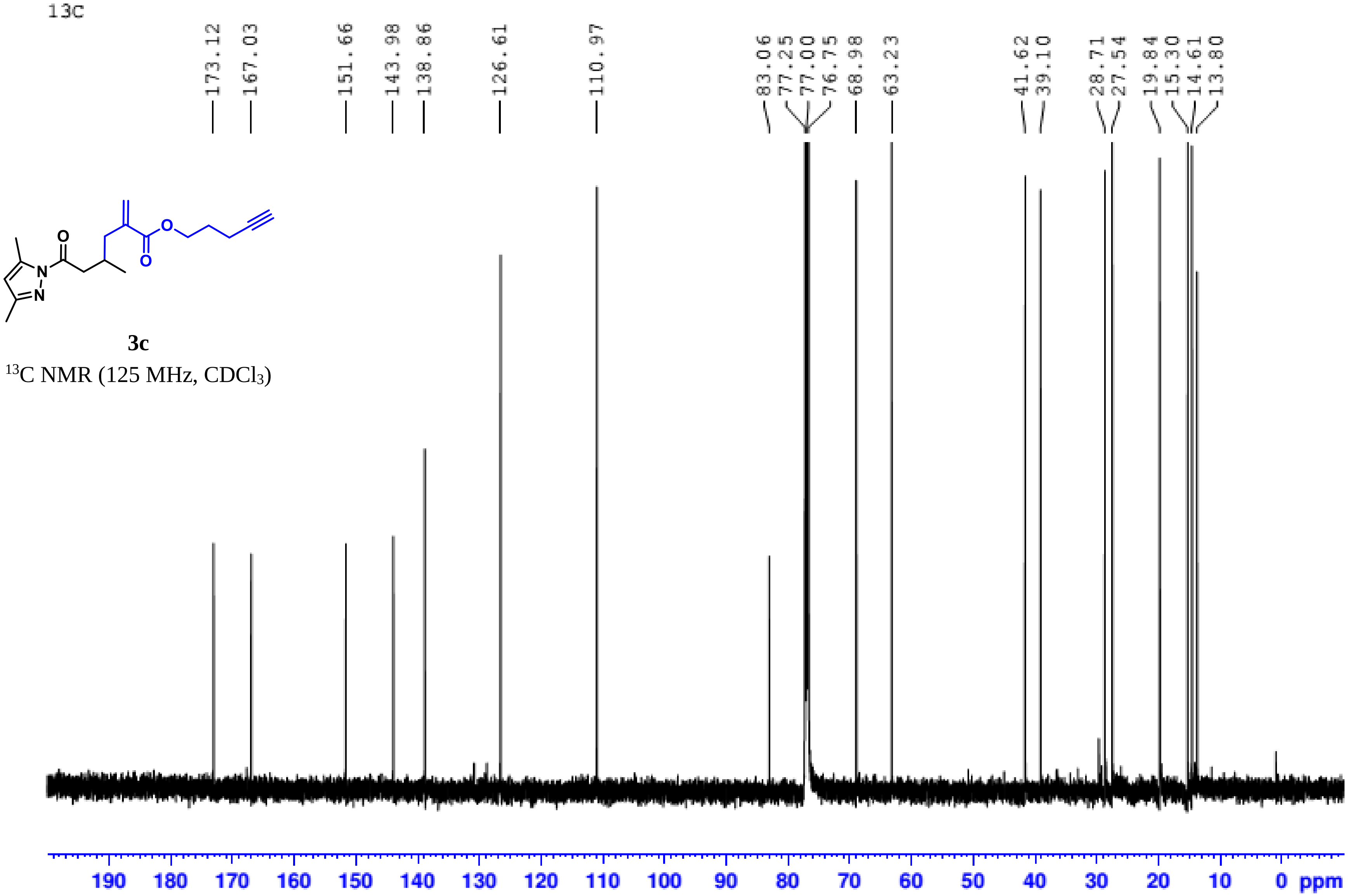

3c

^{13}C NMR (125 MHz, $CDCl_3$)

3d

^{1}H NMR (500 MHz, $CDCl_3$)

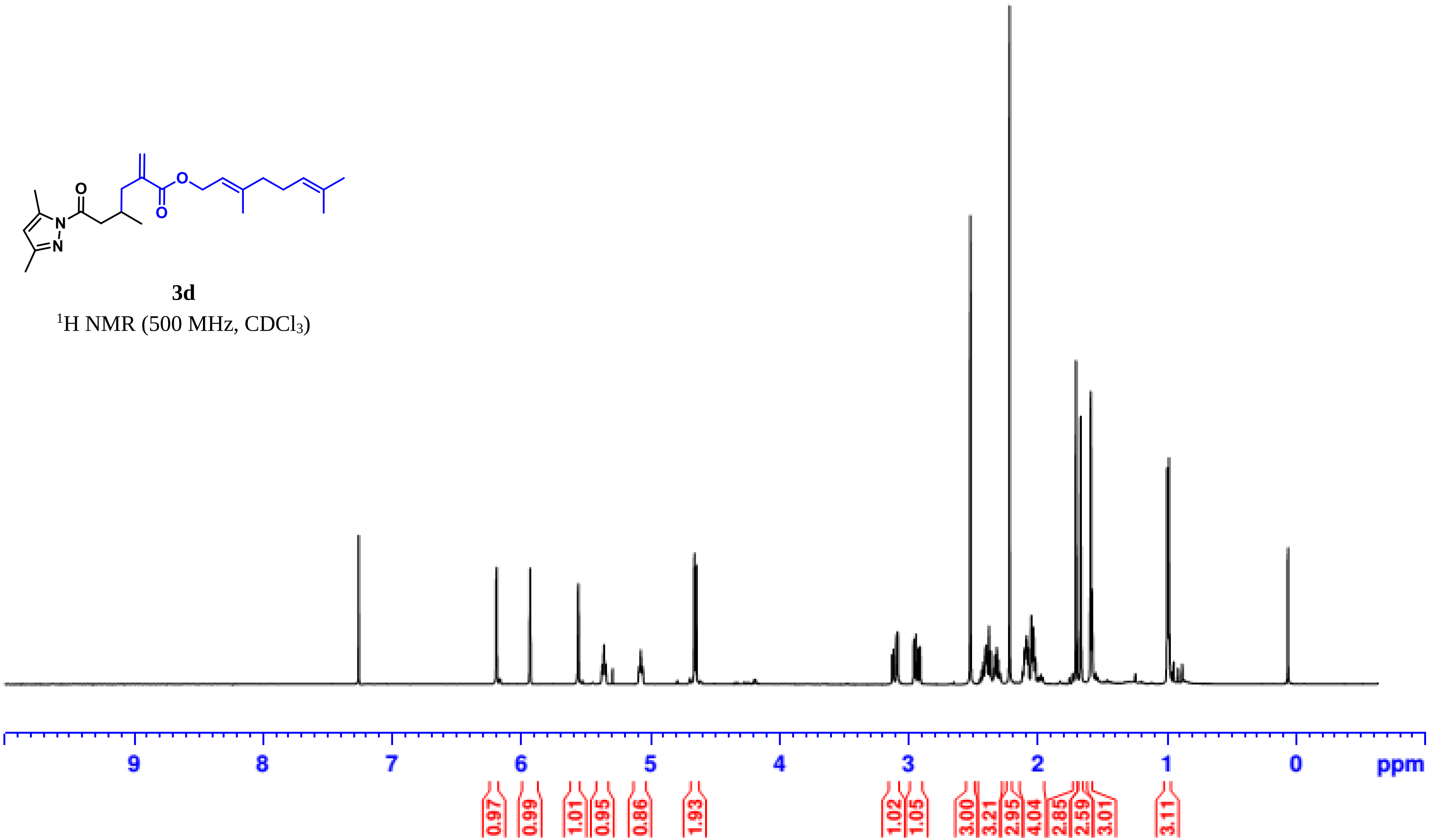

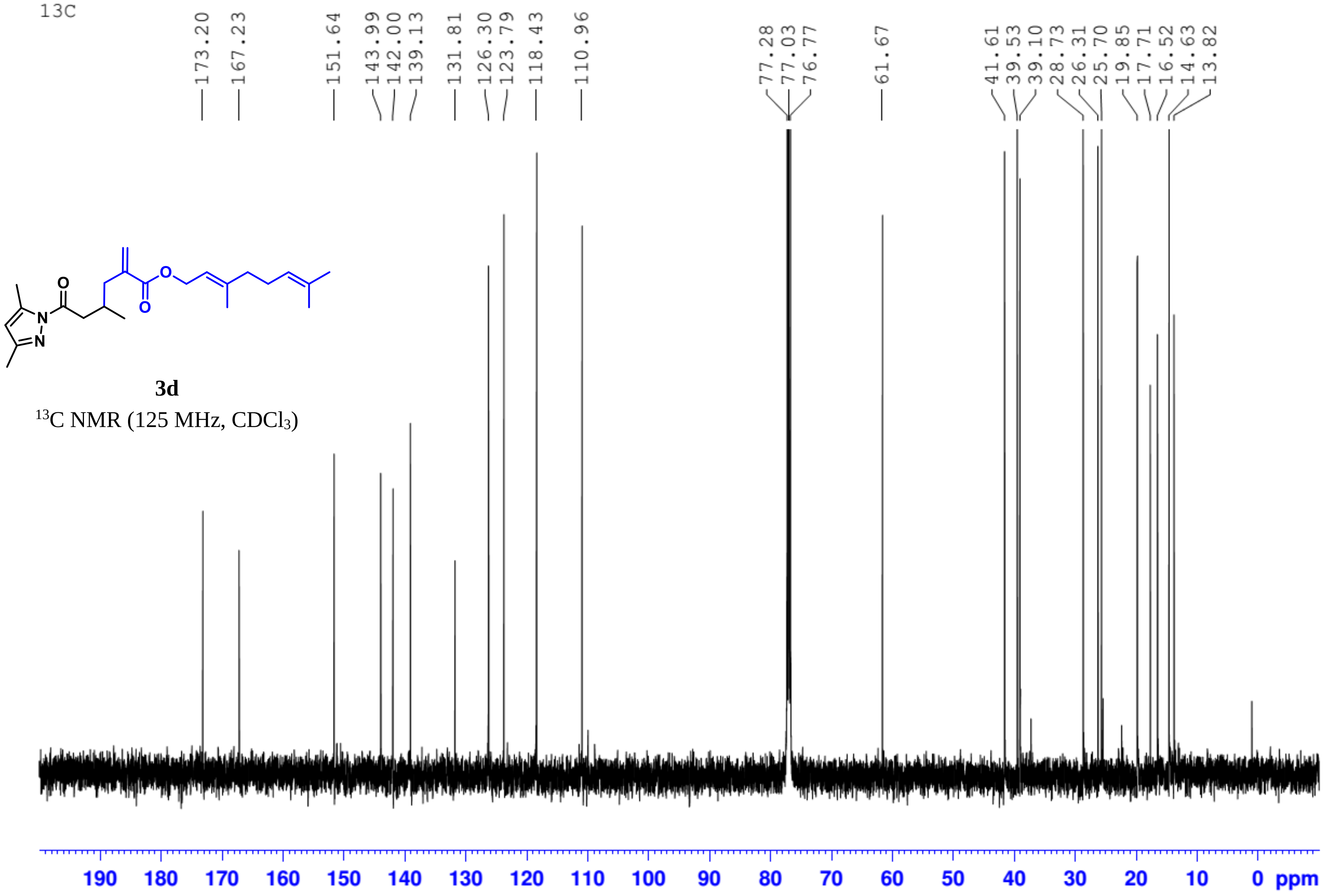

3d

^{13}C NMR (125 MHz, $CDCl_3$)

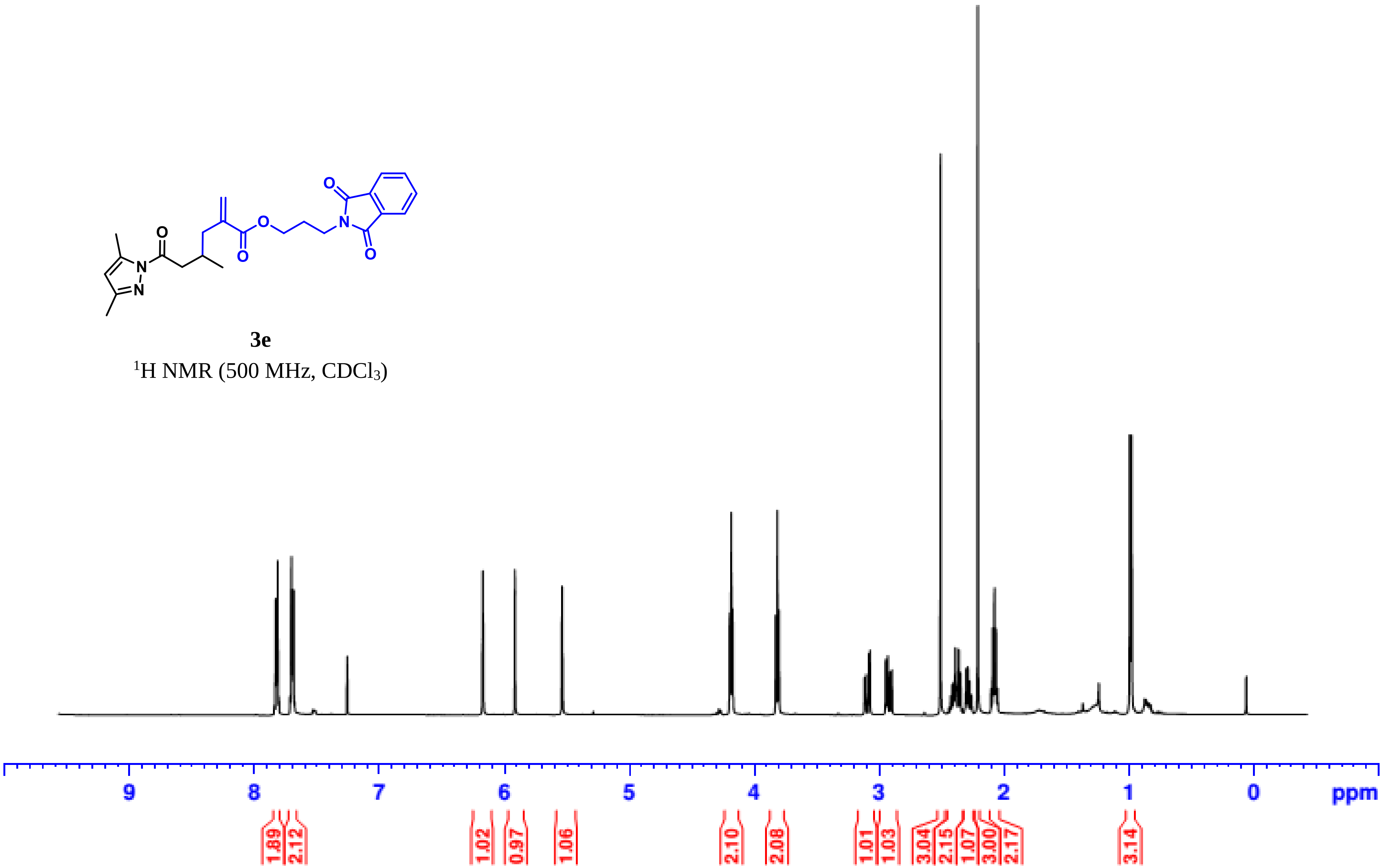

3e

^{1}H NMR (500 MHz, $CDCl_3$)

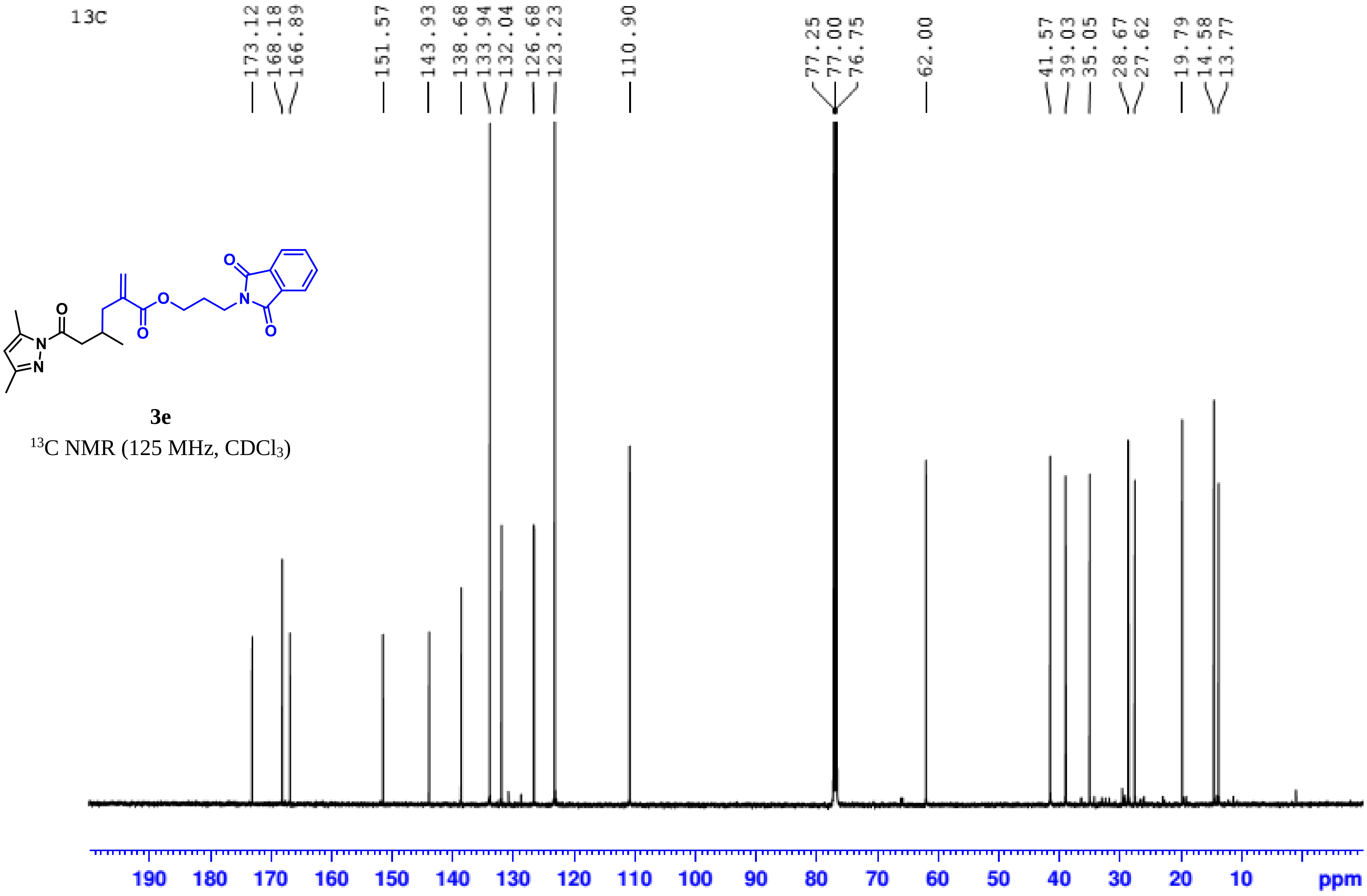

3e

^{13}C NMR (125 MHz, $CDCl_3$)

1H

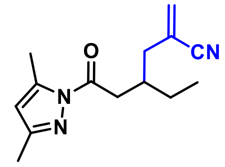

3f

^{1}H NMR (500 MHz, $CDCl_3$)

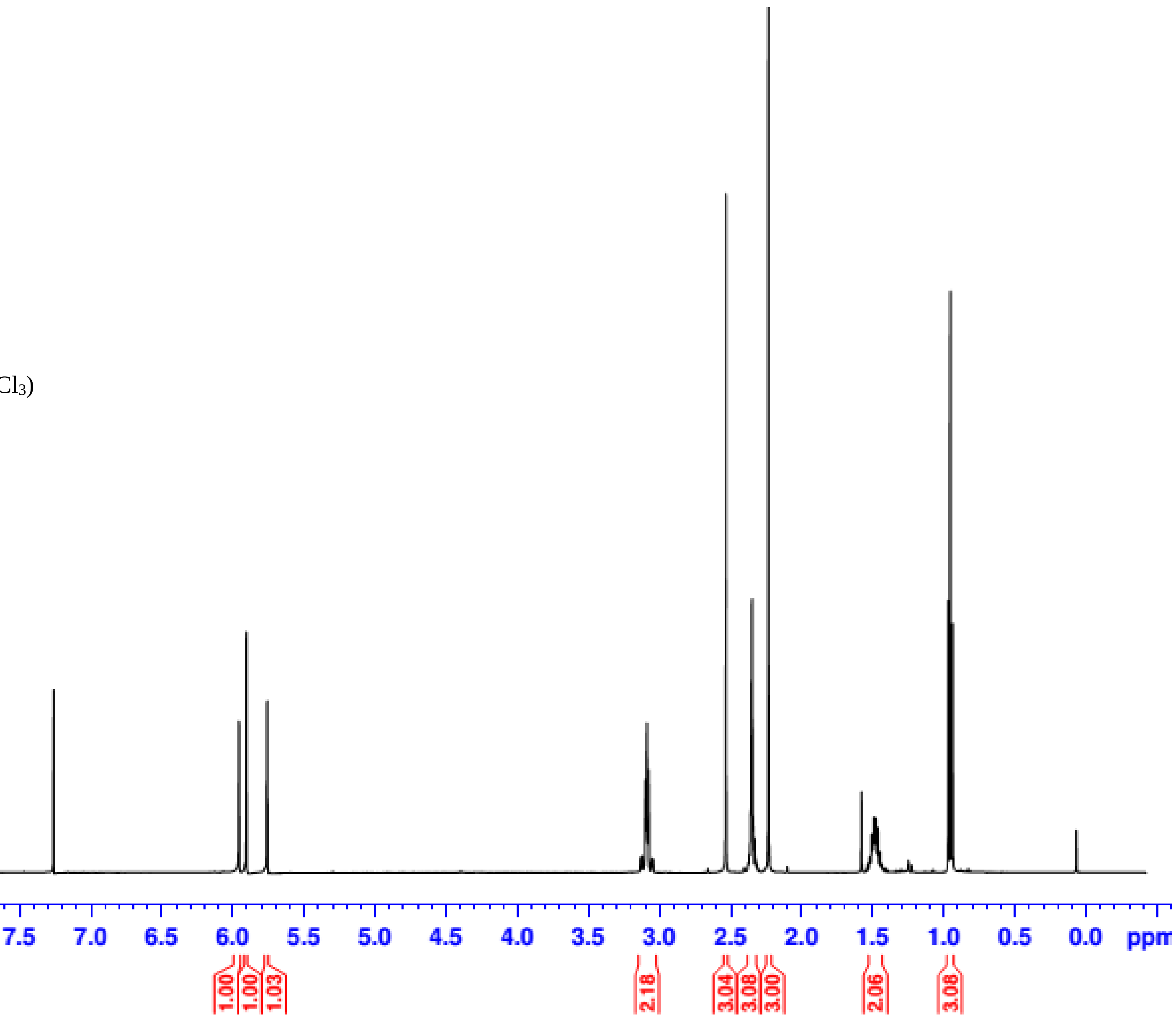

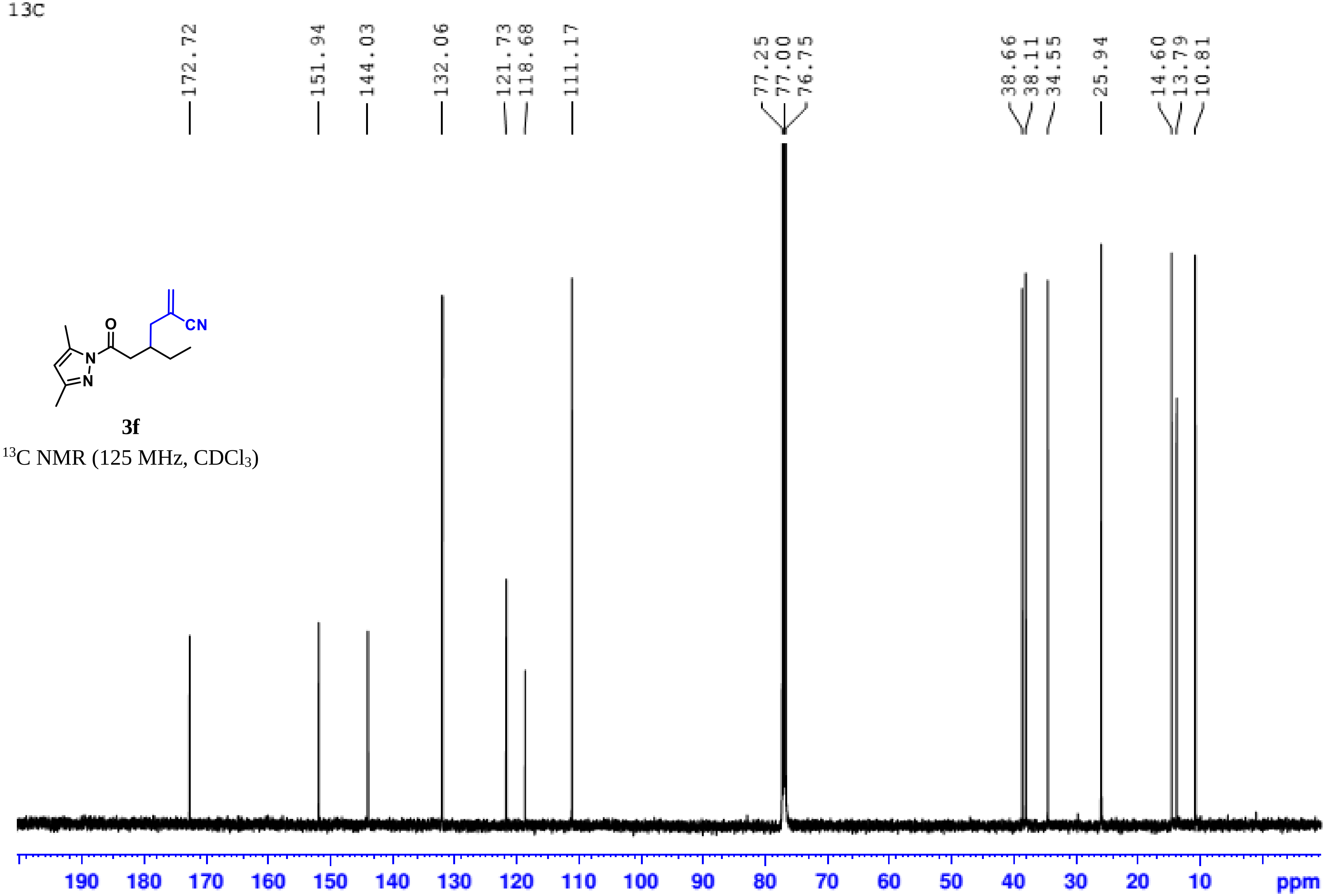

3f

^{13}C NMR (125 MHz, $CDCl_3$)

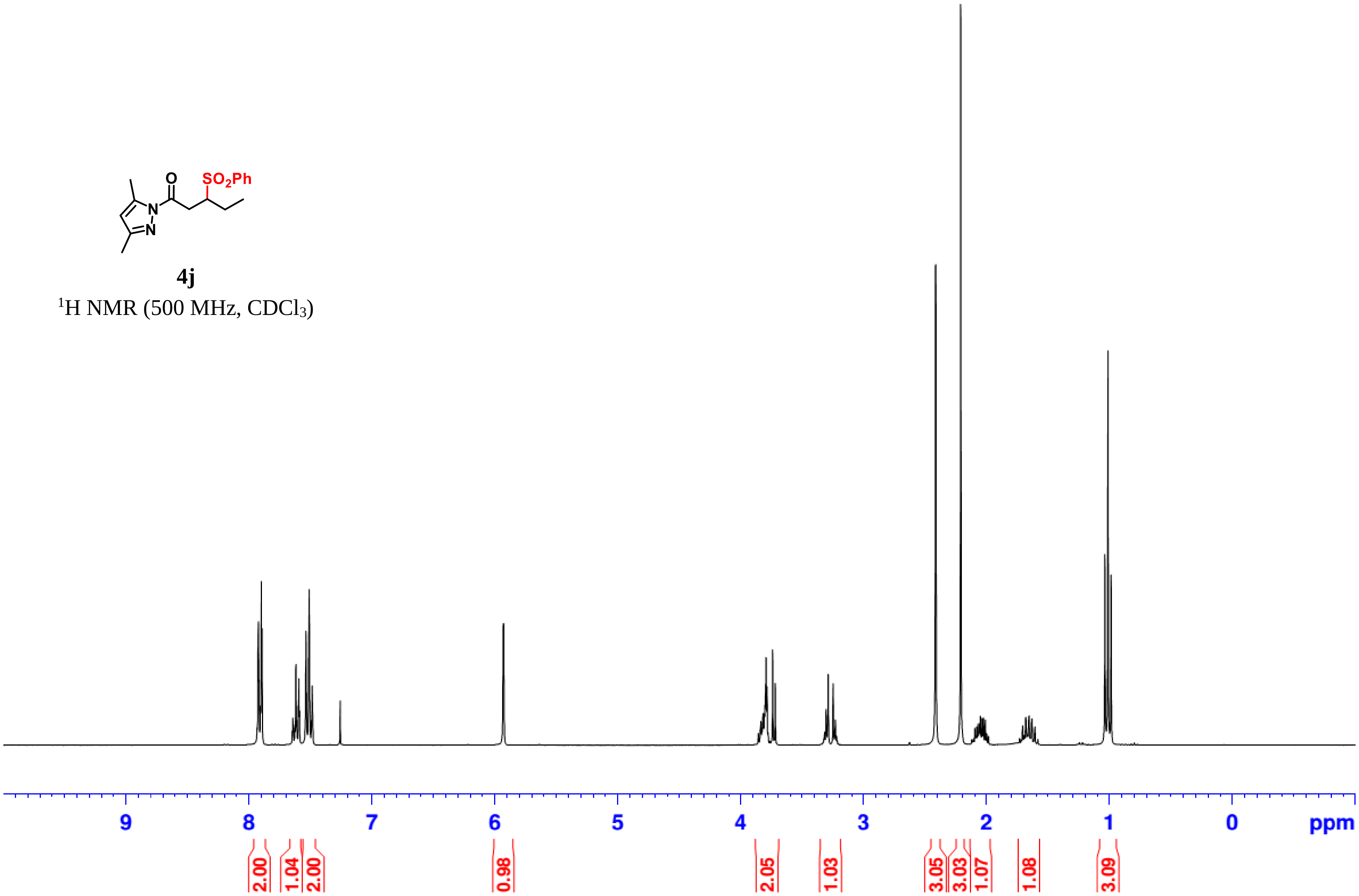

4j

^{1}H NMR (500 MHz, $CDCl_3$)

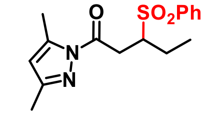

4j

^{13}C NMR (125 MHz, $CDCl_3$)

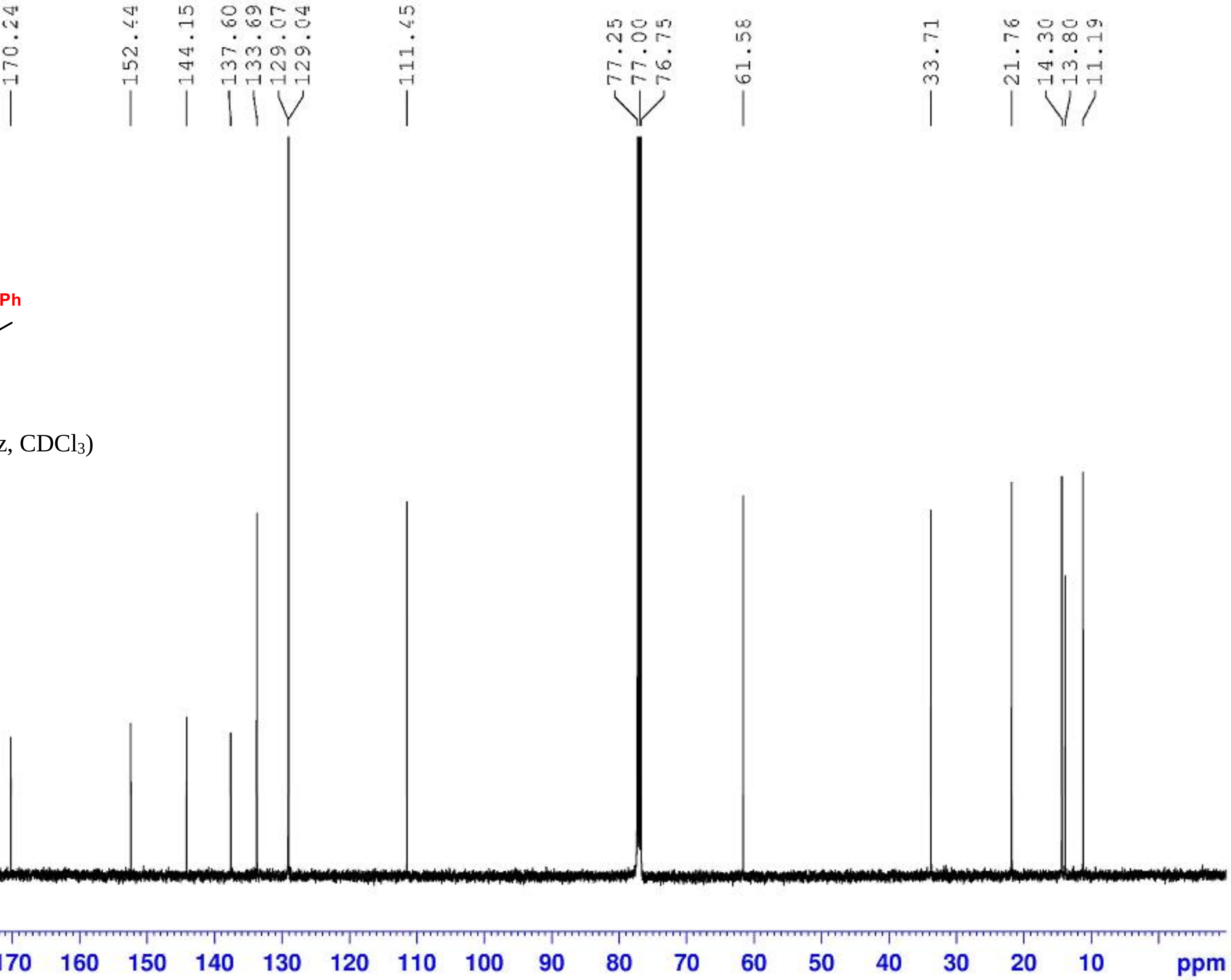

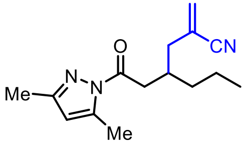

3g

^{1}H NMR (300 MHz, $CDCl_3$)

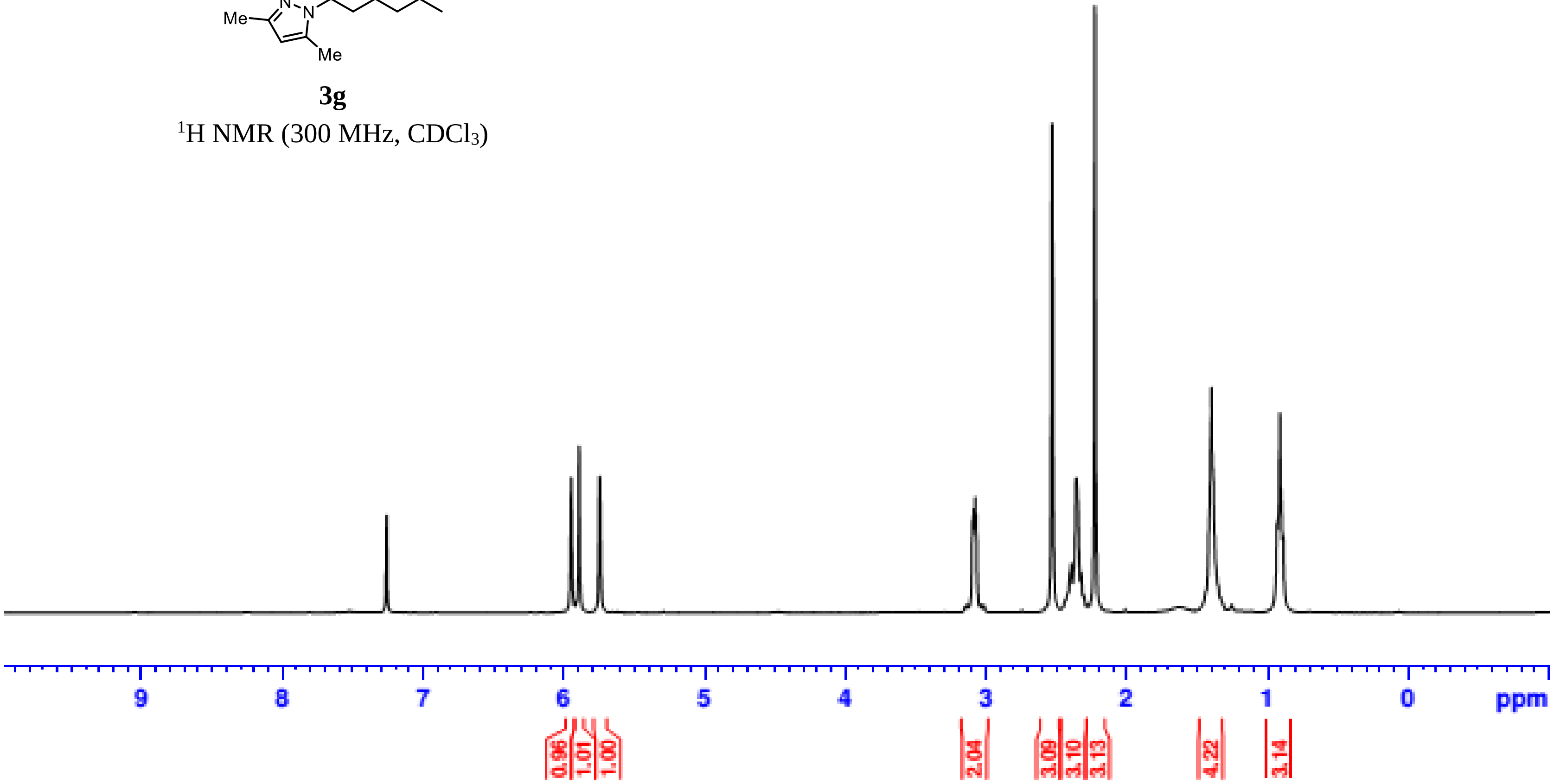

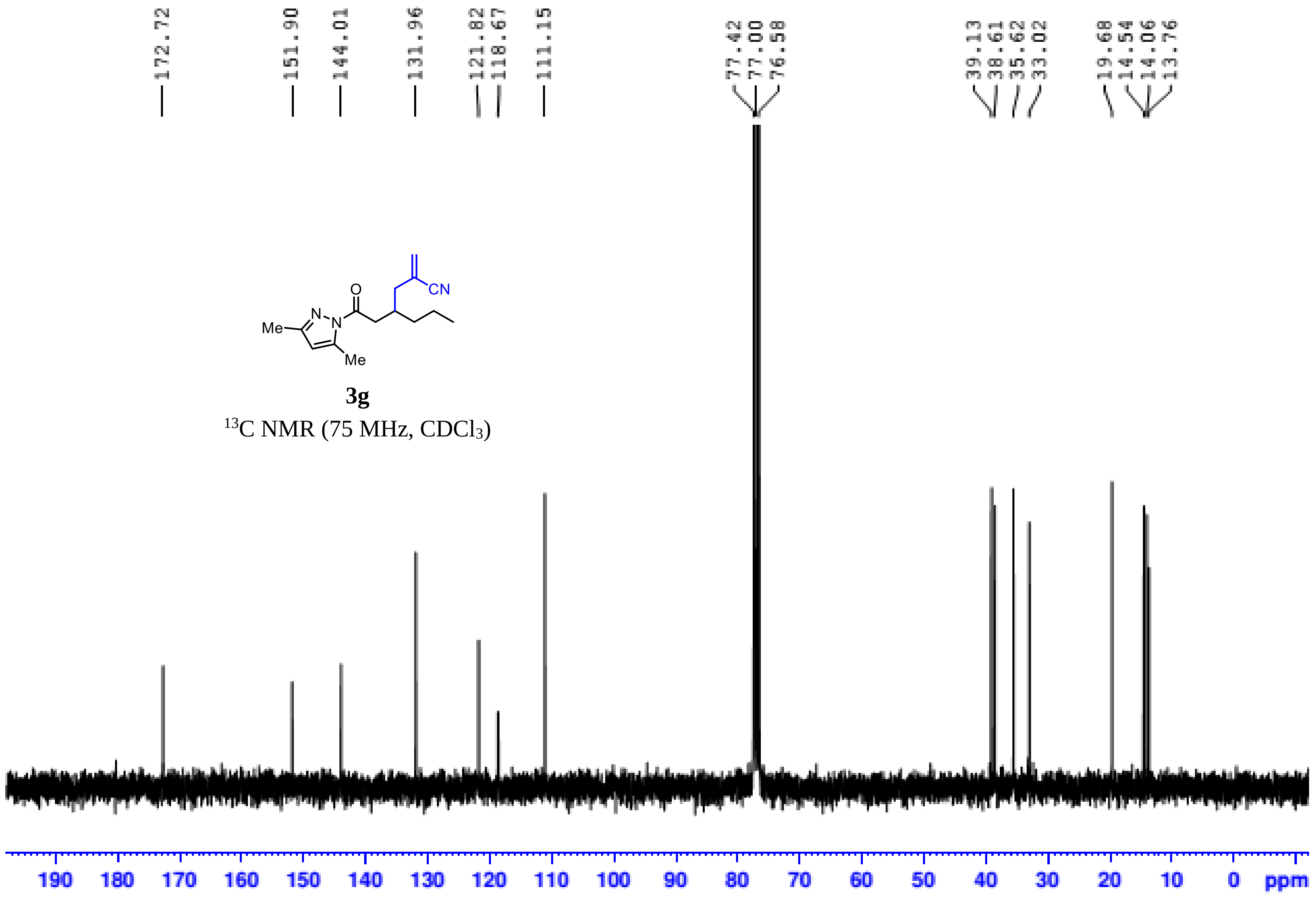

3g

^{13}C NMR (75 MHz, $CDCl_3$)

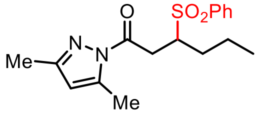

4k

^{1}H NMR (300 MHz, $CDCl_3$)

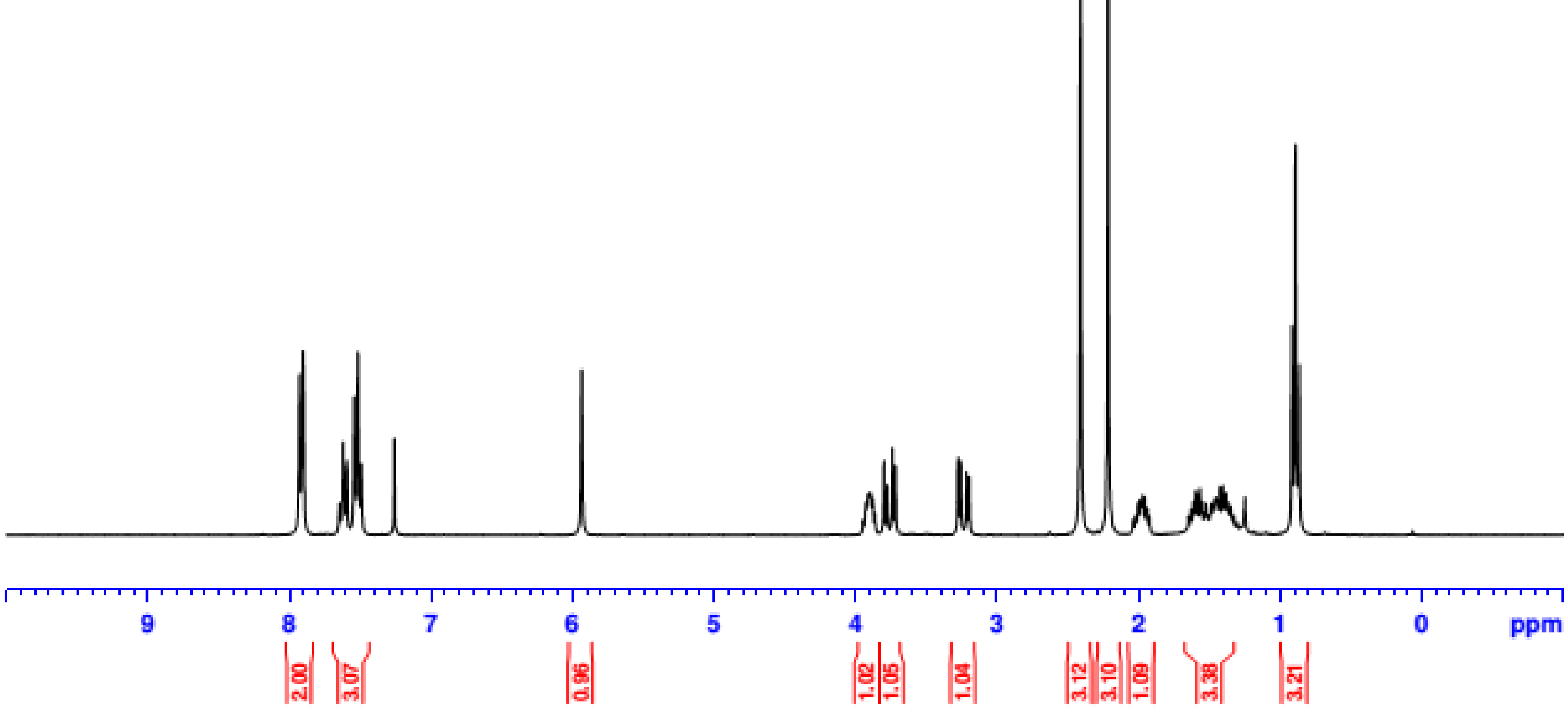

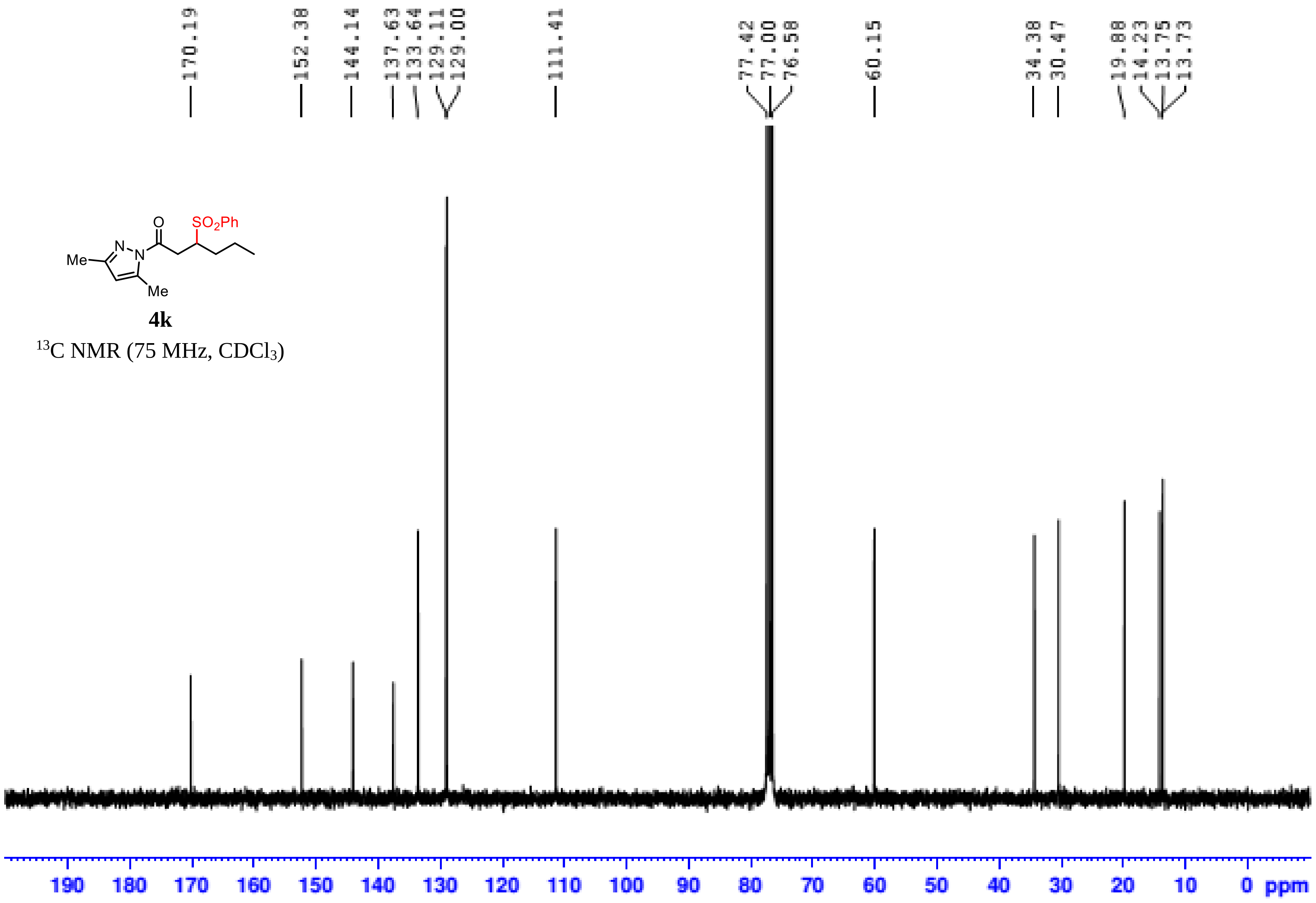

4k

^{13}C NMR (75 MHz, $CDCl_3$)

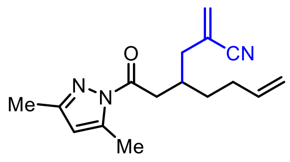

3h

^1H NMR (300 MHz, $CDCl_3$)

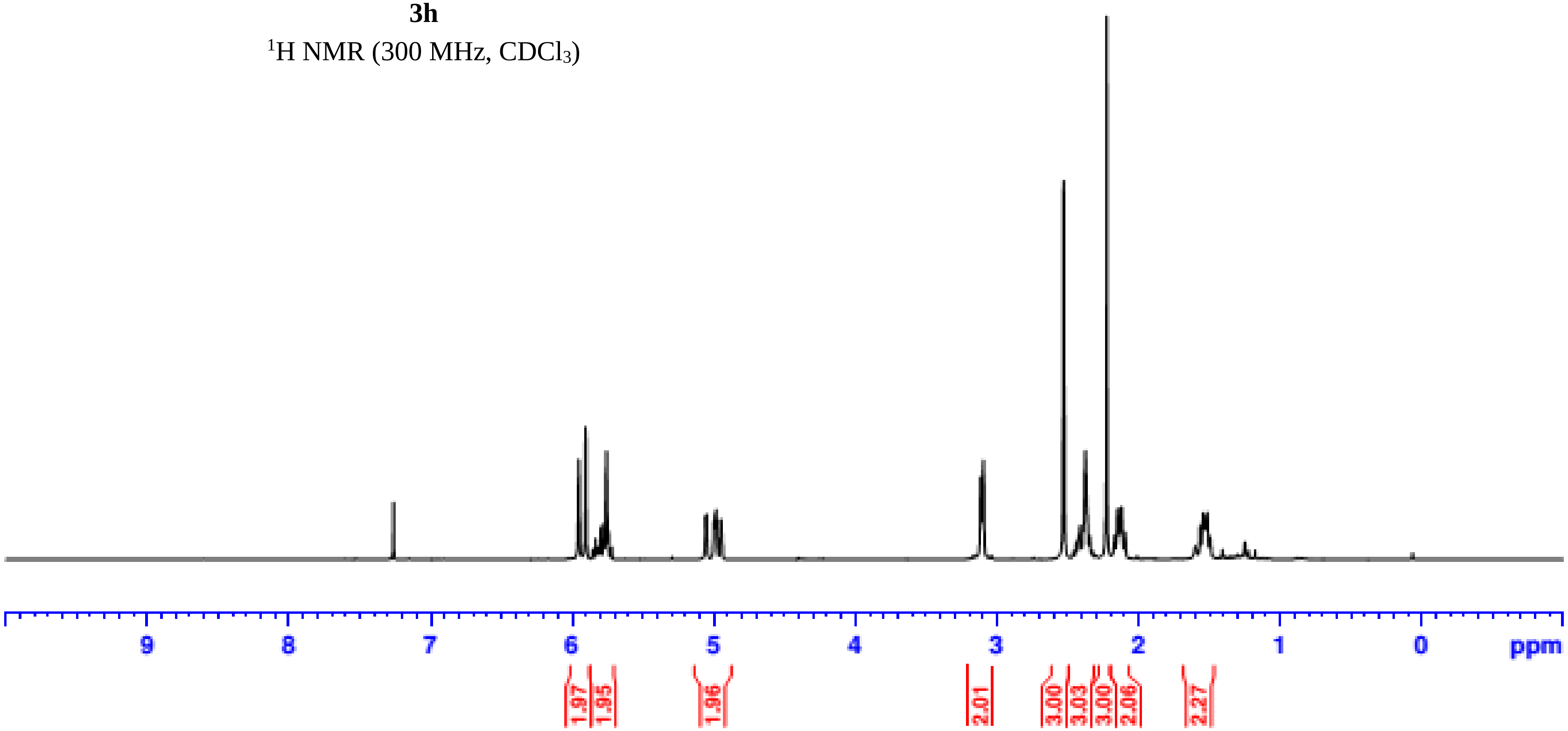

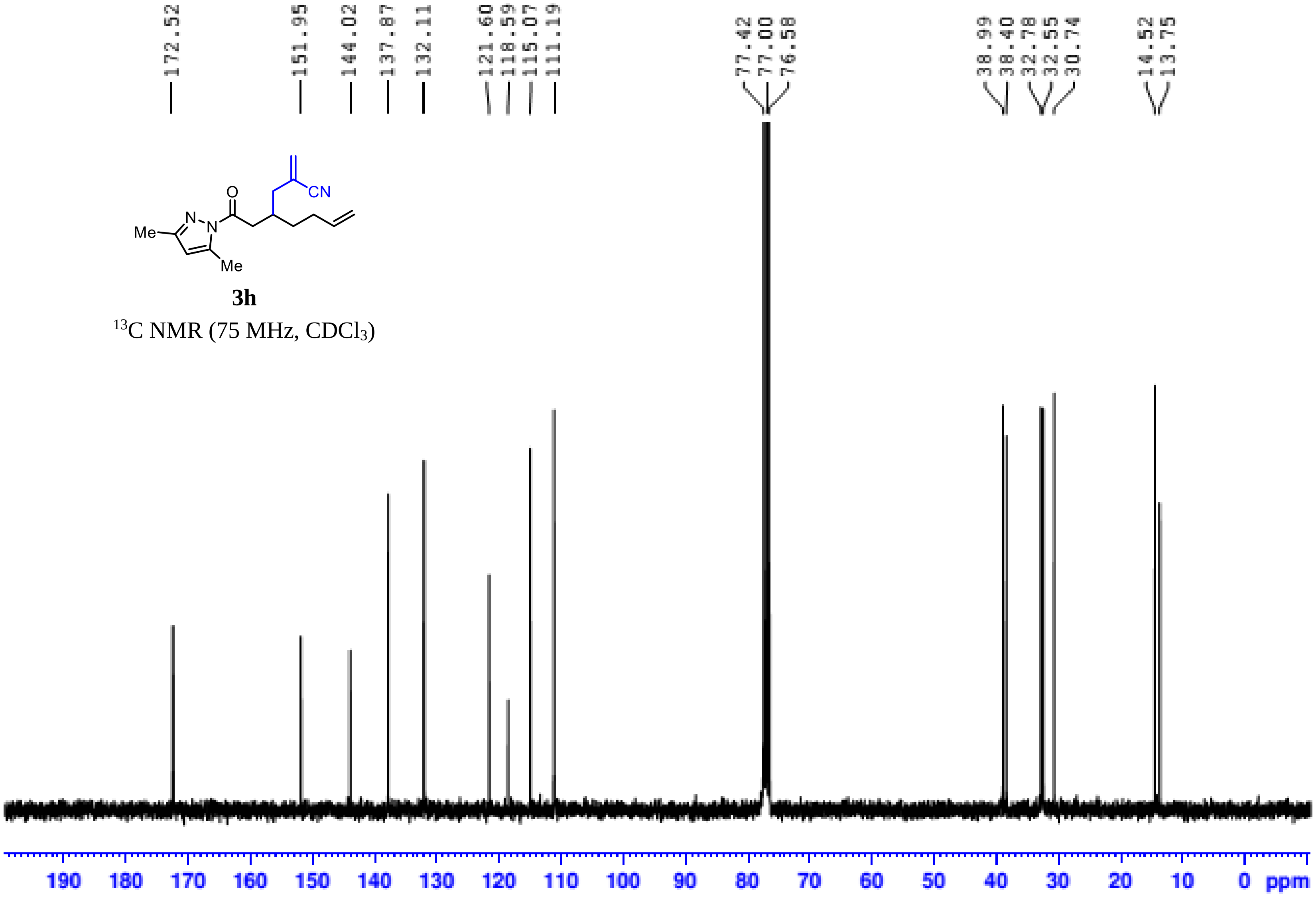

3h

^{13}C NMR (75 MHz, $CDCl_3$)

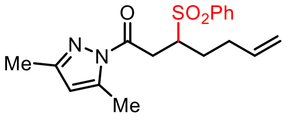

4l

^{1}H NMR (300 MHz, $CDCl_3$)

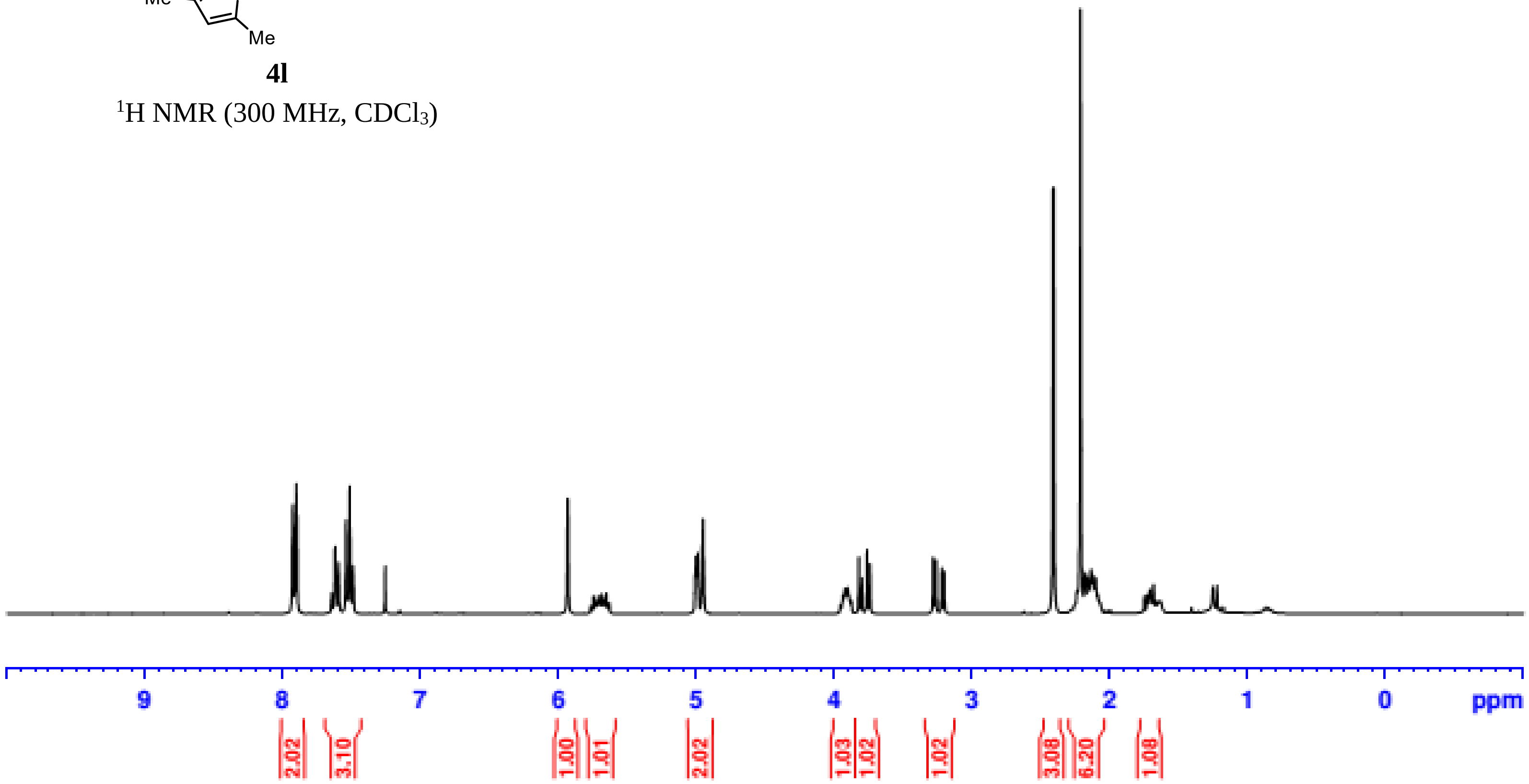

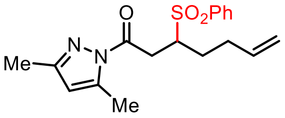

4l

^{13}C NMR (75 MHz, $CDCl_3$)

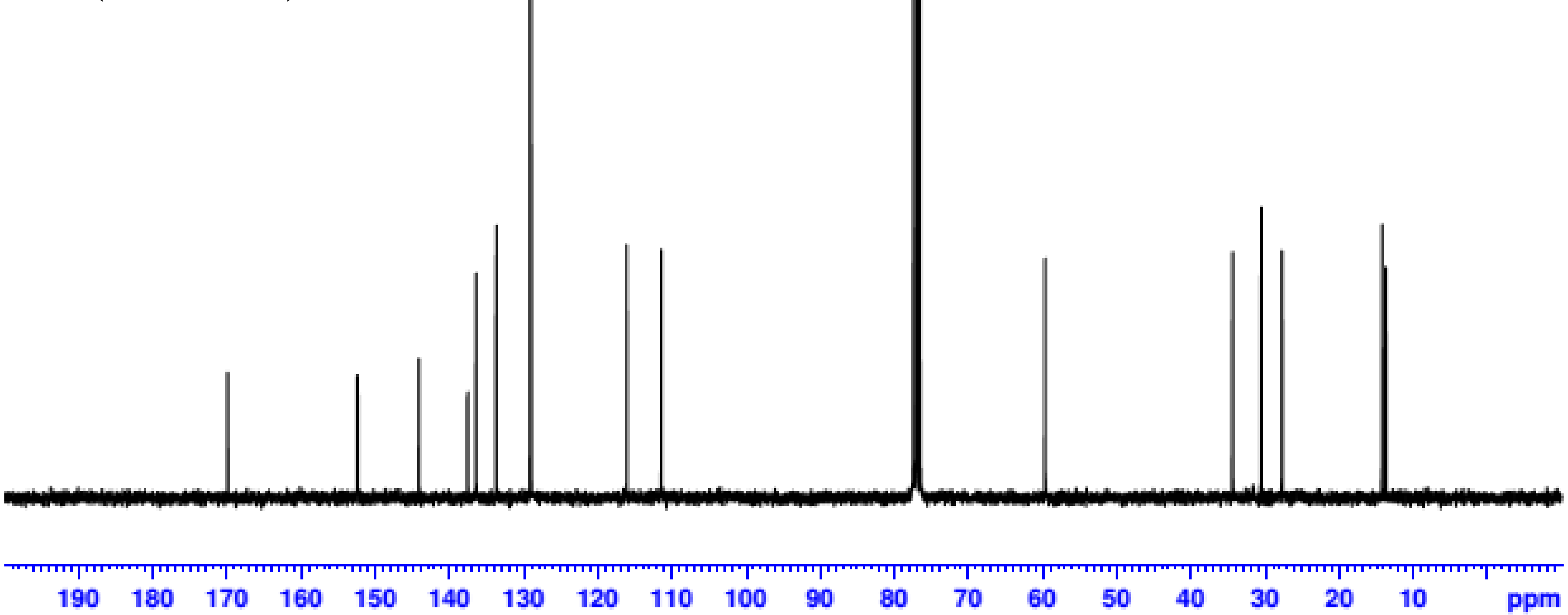

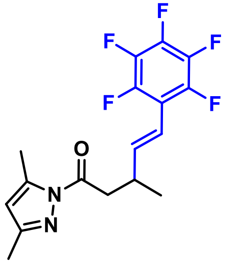

6

^{1}H NMR (500 MHz, $CDCl_3$)

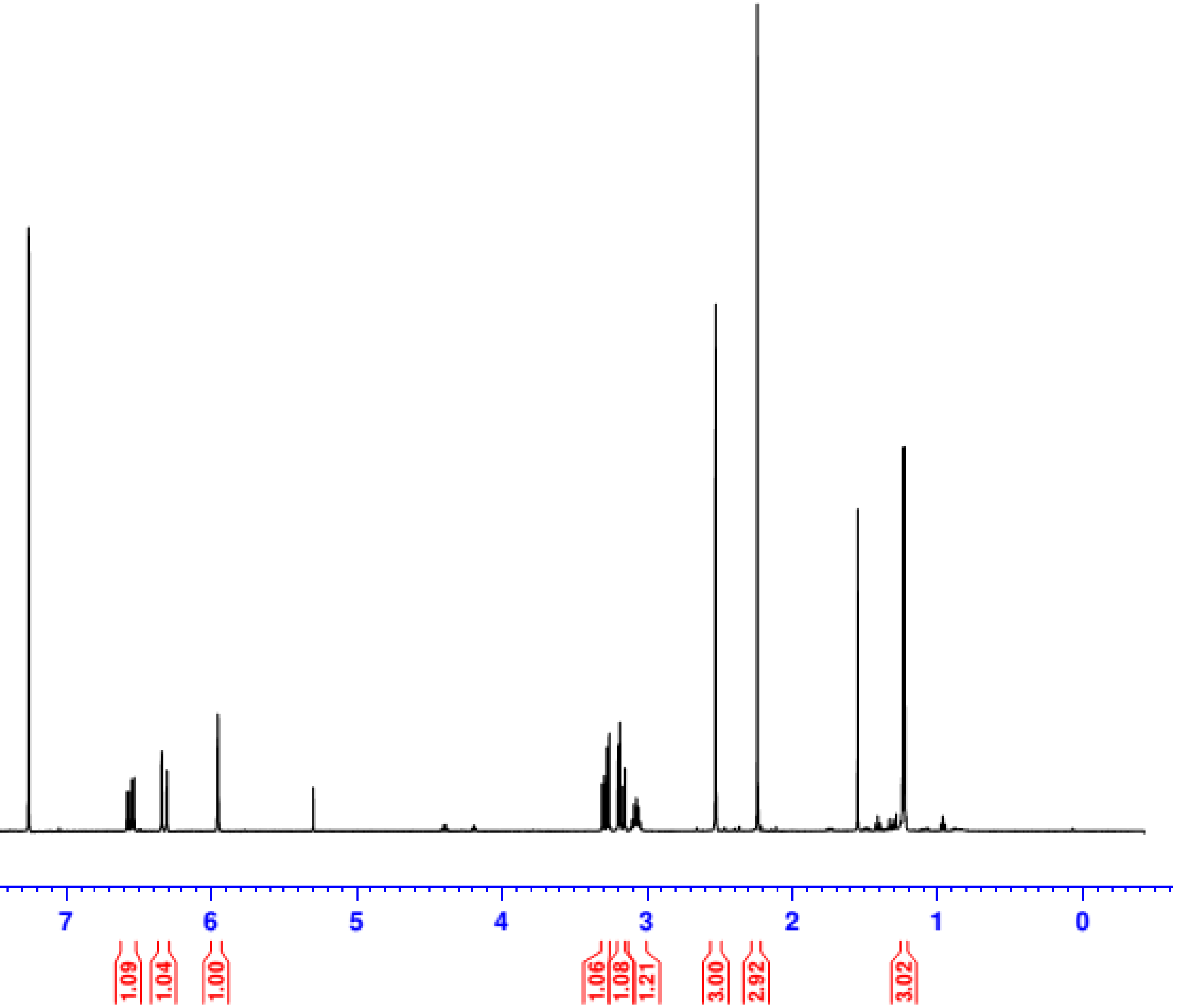

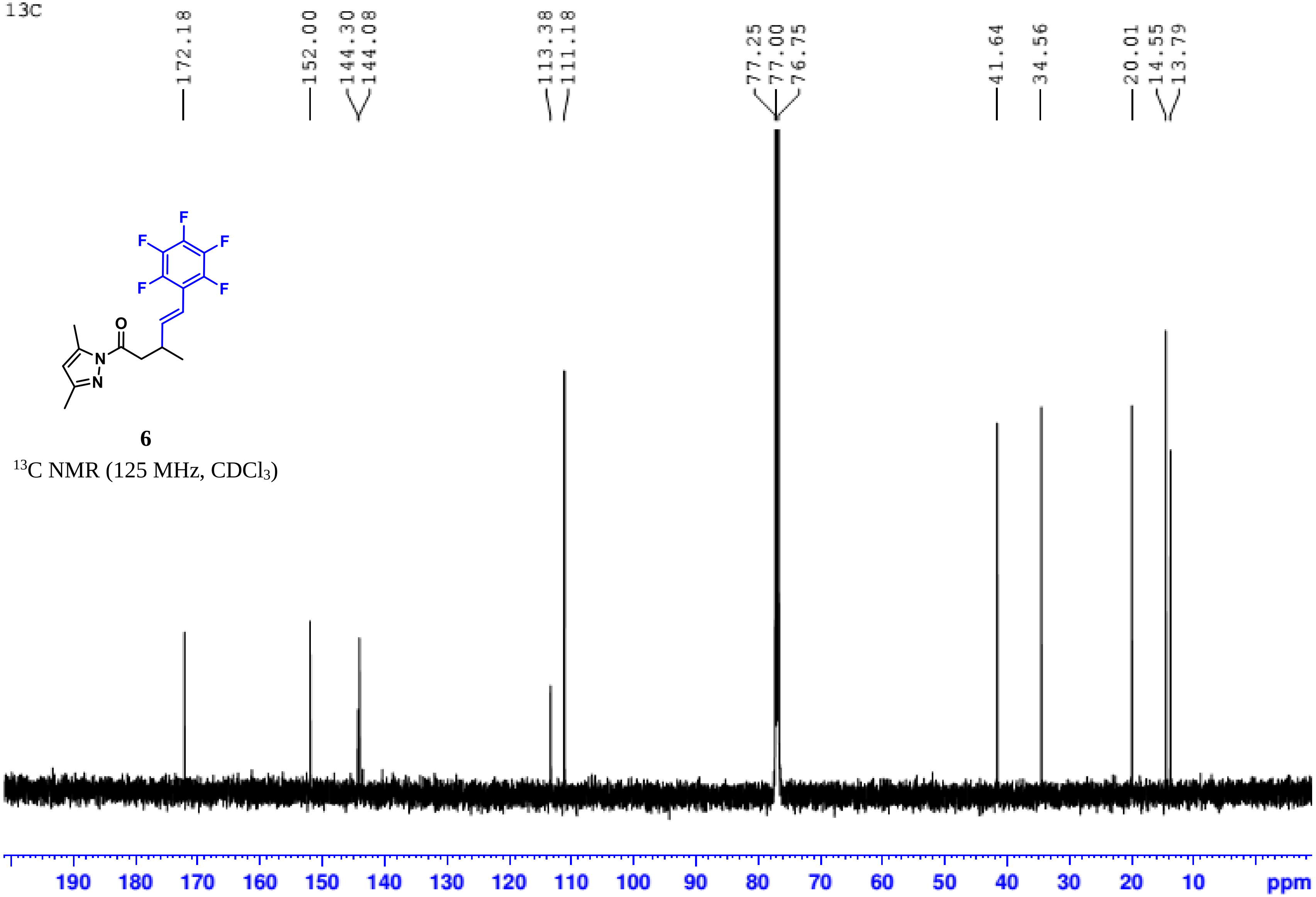

13C
172.18
152.00
144.30
144.08
113.38
111.18
77.25
77.00
76.75
41.64
34.56
20.01
14.55
13.79
F
O
N
N
6
^{13}C NMR (125 MHz, $CDCl_3$)
190 180 170 160 150 140 130 120 110 100 90 80 70 60 50 40 30 20 10 ppm

161115_37654_1sp117-1

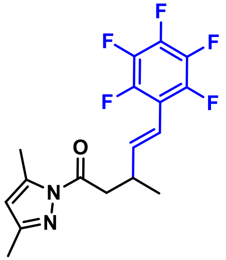

6

^{19}F NMR (282 MHz, CDCl$_3$)

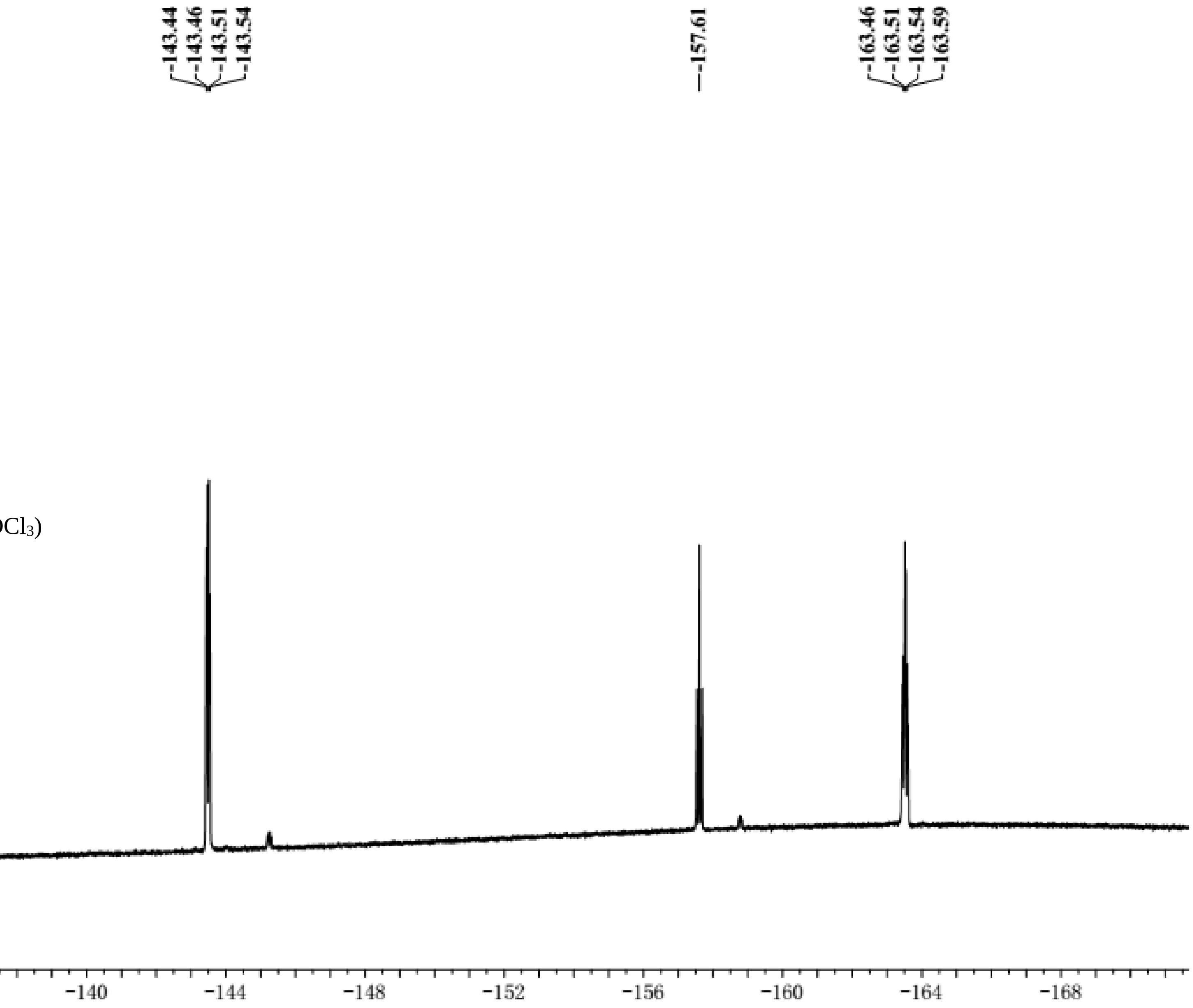

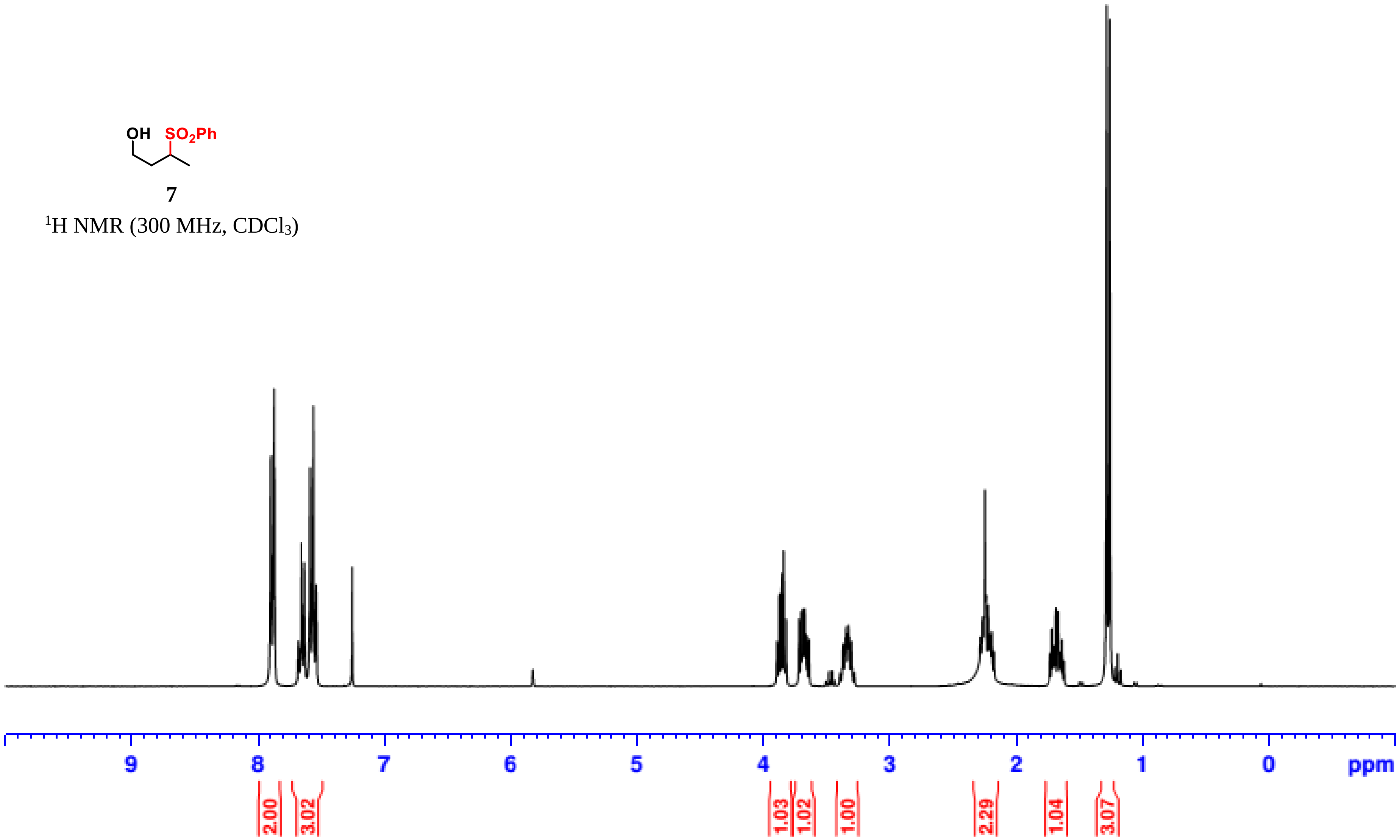

7

^{1}H NMR (300 MHz, $CDCl_3$)

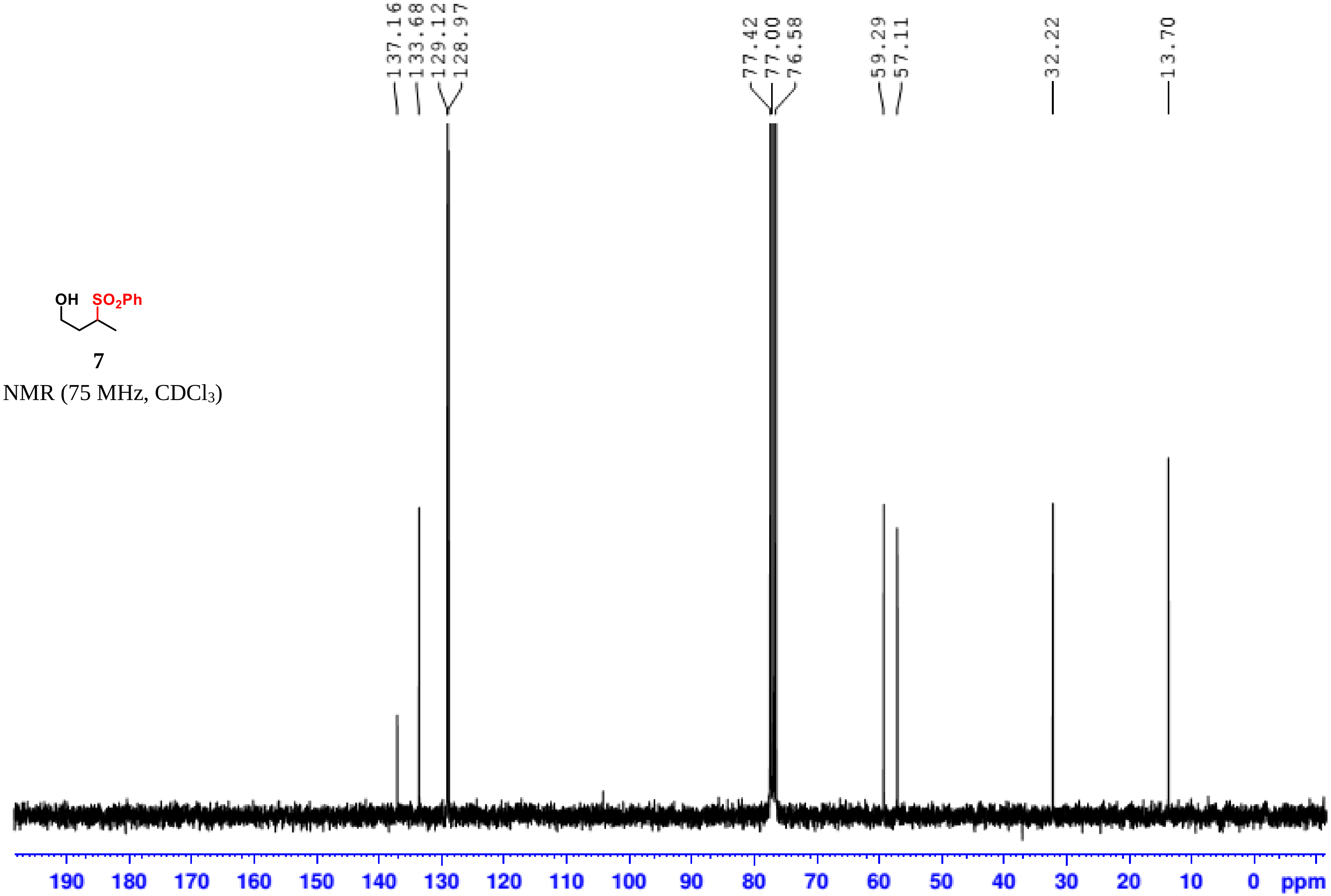

7

^{1}C NMR (75 MHz, $CDCl_3$)

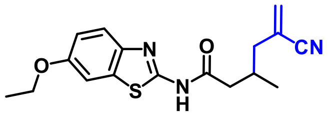

8

^{1}H NMR (300 MHz, $CDCl_3$)

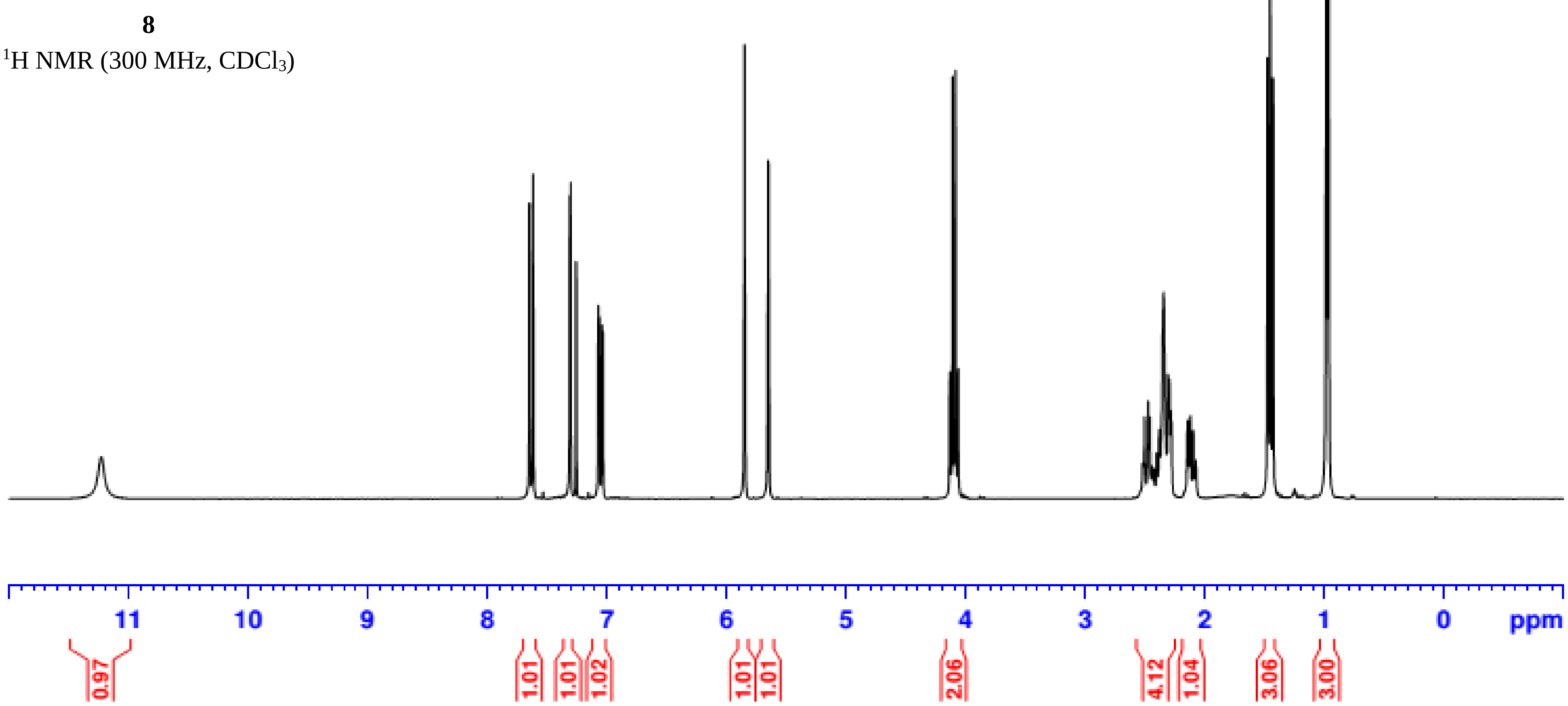

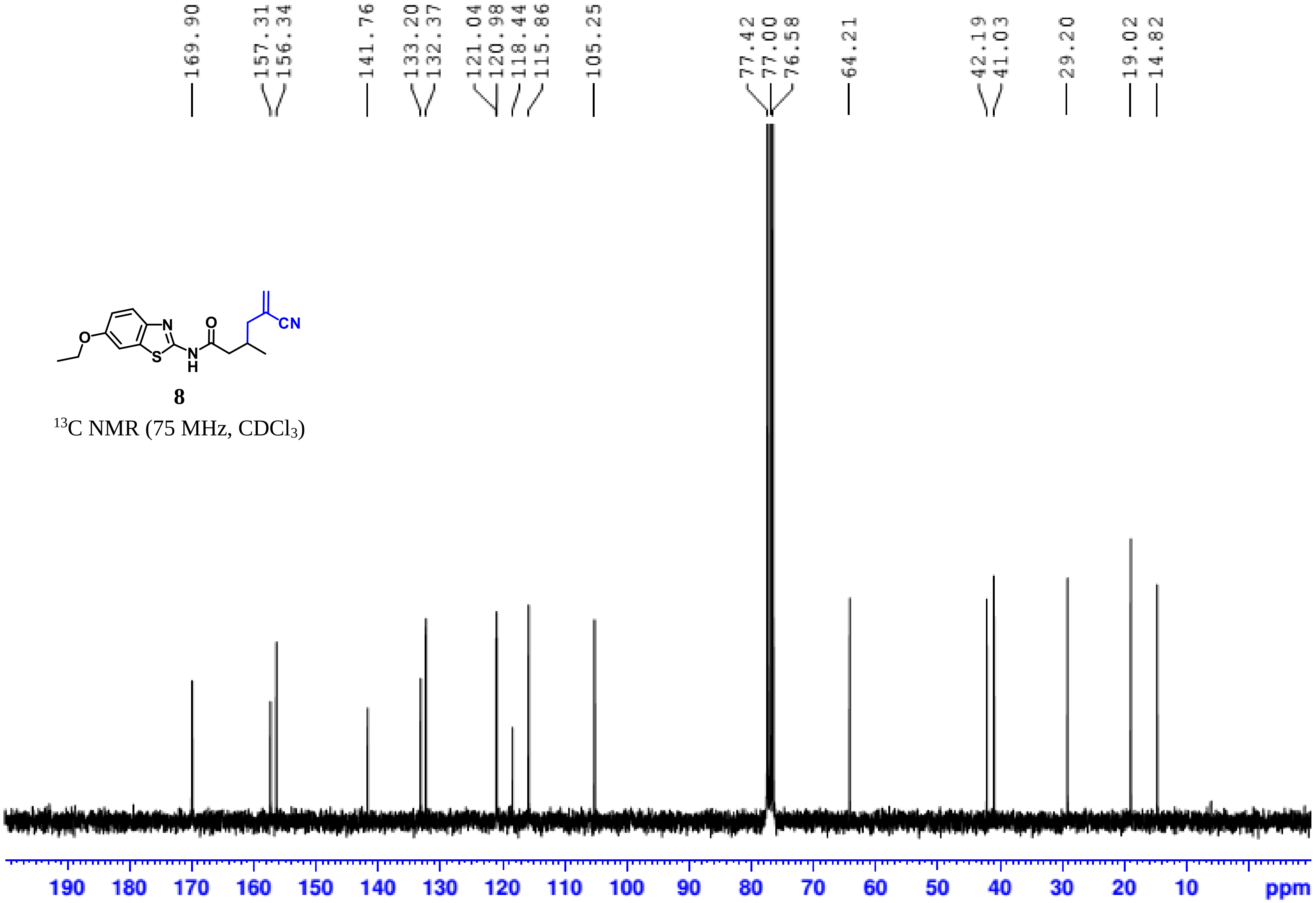

8

^{13}C NMR (75 MHz, $CDCl_3$)

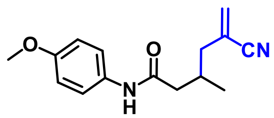

9

^{1}H NMR (300 MHz, $CDCl_3$)

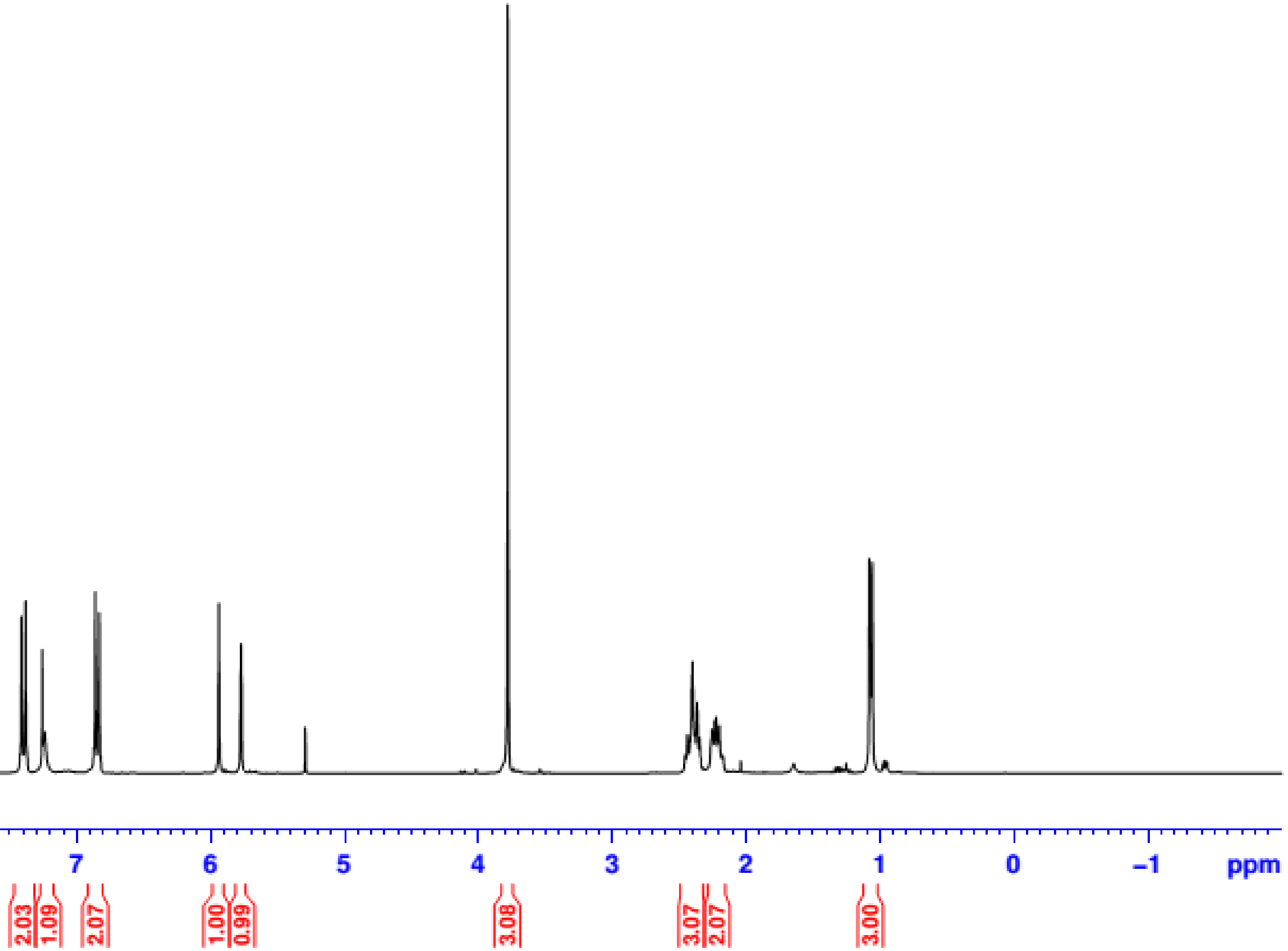

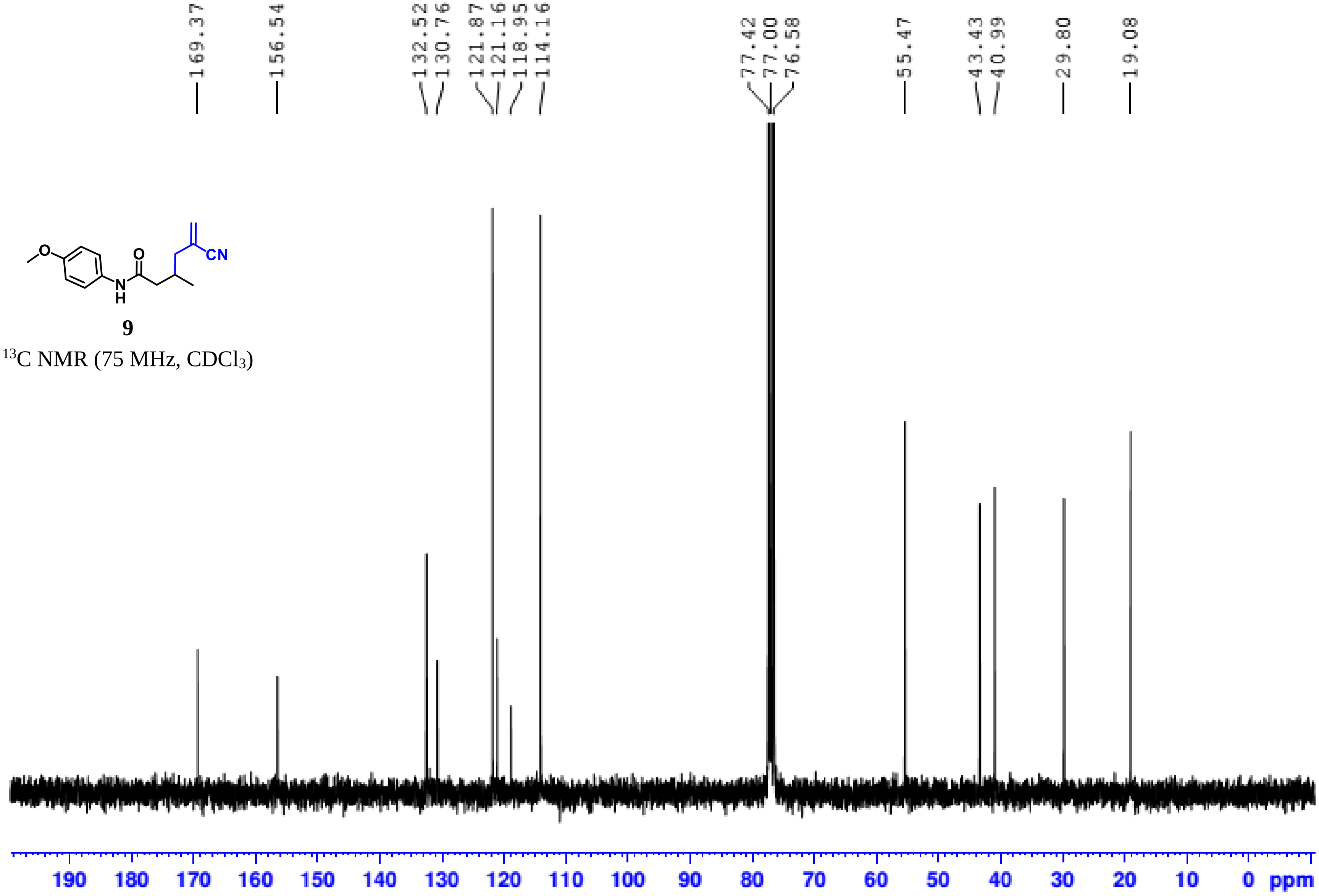

9

^{13}C NMR (75 MHz, $CDCl_3$)

13. References

(S1) X. Shen, H. Huo, C. Wang, B. Zhang, K. Harms and E. Meggers, *Chem.-Eur. J.*, 2015, **21**, 9720.

(S2) J. Ma, X. Shen, K. Harms and E. Meggers, *Dalton Trans.*, 2016, **45**, 8320.

(S3) C. Wang, L.-A. Chen, H. Huo, X. Shen, K. Harms, L. Gong and E. Meggers, *Chem. Sci.*, 2015, **6**, 1094.

(S4) A. L. Fuentes de Arriba, F. Urbitsch and D. J. Dixon, *Chem. Commun.*, 2016, **52**, 14434.

(S5) M. P. Sibi and K. Itoh, *J. Am. Chem. Soc.*, 2007, **129**, 8064.

(S6) H. T. Abdel-Mohsen, J. Conrad and U. Beifuss, *Green Chem.*, 2012, **14**, 2686.

(S7) Y. Zheng, K. Harms, L. Zhang and E. Meggers, *Chem.-Eur. J.*, 2016, **22**, 11977.

(S8) C. Kashima, H. Harada, I. Kita, I. Fukuchi and A. Hosomi, *Synthesis*, 1994, 61.

(S9) X. Huang, T. R. Quinn, K. Harms, R. D. Webster, L. Zhang, O. Wiest and E. Meggers, *J. Am. Chem. Soc.*, 2017, **139**, 9120; and ref. S3.

(S10) H. Huo, K. Harms and E. Meggers, *J. Am. Chem. Soc.*, 2016, **138**, 6936.

(S11) J. Y. Lee, Y. T. Hong and S. Kim, *Angew. Chem. Int. Ed.*, 2006, **45**, 6182.

(S12) H. Wang, Q. Lu, C.-W. Chiang, Y. Luo, J. Zhou, G. Wang and A. Lei, *Angew. Chem. Int. Ed.*, 2017, **56**, 595.

(S13) (a) U. Megerle, R. Lechner, B. Konig and E. Riedle, *Photochem. Photobiol. Sci.*, 2010, **9**, 1400; (b) T. Ghosh, T. Slanina and B. König, *Chem. Sci.*, 2015, **6**, 2027.

(S14) J. Zhang, Y. Li, F. Zhang, C. Hu and Y. Chen, *Angew. Chem. Int. Ed.*, 2016, **55**, 1872.

(S15) *APEX3*, Bruker AXS Inc., Madison, Wisconsin, USA, 2015.

(S16) *SAINT*, Bruker AXS Inc., Madison, Wisconsin, USA, 2015.

(S17) *SADABS. Bruker AXS area detector scaling and absorption correction*, Bruker AXS Inc., Madison, Wisconsin, USA, 2014.

(S18) G. M. Sheldrick, *Acta Crystallogr. A, Found. Adv.*, 2015, **71**, 3.

(S19) G. M. Sheldrick, *Acta crystallogr. Sec. C, Struct. Chem.*, 2015, **71**, 3.

(S20) K. Brandenburg, *Diamond - Crystal and Molecular Structure Visualization*, Crystal Impact - Dr. Putz, H. & Dr. Brandenburg K. GbR, Bonn, Germany, 2014.

(S21) C. B. Hübschle, G. M. Sheldrick and B. Dittrich, *J. Appl. Crystallog.*, 2011, **44**, 1281.

(S22) S. Parsons, H. D. Flack and T. Wagner, *Acta Crystallogr., Sect. B: Struct. Sci., Cryst. Eng. Mater.*, 2013, **69**, 249.
